# Thorium-nitrogen multiple bonds provide evidence for pushing-from-below for early actinides

**DOI:** 10.1038/s41467-019-12206-5

**Published:** 2019-09-13

**Authors:** Jingzhen Du, Carlos Alvarez-Lamsfus, Elizabeth P. Wildman, Ashley J. Wooles, Laurent Maron, Stephen T. Liddle

**Affiliations:** 1 Department of Chemistry, The University of Manchester, Oxford Road, Manchester, M13 9PL UK; 2 LPCNO, CNRS & INSA, Université Paul Sabatier, 135 Avenue de Rangueil, 31077 Toulouse, France

**Keywords:** Coordination chemistry, Chemical bonding

## Abstract

Although the chemistry of uranium-ligand multiple bonding is burgeoning, analogous complexes involving other actinides such as thorium remain rare and there are not yet any terminal thorium nitrides outside of cryogenic matrix isolation conditions. Here, we report evidence that reduction of a thorium-azide produces a transient Th≡N triple bond, but this activates C-H bonds to produce isolable parent imido derivatives or it can be trapped in an N-heterocycle amine. Computational studies on these thorium-nitrogen multiple bonds consistently evidences a σ > π energy ordering. This suggests pushing-from-below for thorium, where 6p-orbitals principally interact with filled f-orbitals raising the σ-bond energy. Previously this was dismissed for thorium, being the preserve of uranium-nitrides or the uranyl dication. Recognising that pushing-from-below perhaps occurs with thorium as well as uranium, and with imido ligands as well as nitrides, suggests this phenomenon may be more widespread than previously thought.

## Abstract (web summary)

Despite the burgeoning nature of uranium–ligand multiple bonding, analogous thorium complexes remain incredibly rare. Here the authors report evidence for a transient thorium–nitride species, which, together with data on parent imido derivatives, suggests that the pushing-from-below phenomenon may be more widespread than previously thought.

## Introduction

In recent years there has been intense interest in early actinide-nitrides, [An≡N]_n_ (An = uranium or thorium), in molecular and materials contexts^1,2^. From the patent for the Haber Bosch chemical process for ammonia synthesis in the early 1900s it has been known that uranium-nitrides are implicated in the formation of ammonia, where uranium was noted to be the best promoter for this heterogeneous process^3^. Binary nitride materials also have great potential as accident tolerant fuels, and although much focus has been on uranium-nitrides it is being increasingly recognised that proliferation-resistant thorium-nitride offers potential in next generation reactors due to a range of favourable physical properties^4,5^. It is thus important to study such materials, but these binary systems can be ill-defined and difficult to probe, and so there has been interest in studying well-defined molecular congeners^6^ where the nature of the chemical bonding and electronic structure of actinides can be probed^7^. This addresses one of the longstanding challenges of actinide chemistry, which is the study of covalency, since this could benefit recycling and clean up of nuclear fuels and wastes. Although binary materials are unlikely to contain much actinide-element multiple bonding, the study of discrete molecular linkages is important because they usually exhibit covalency^8,9^, and only by generating such linkages can the chemistry of those bonds be benchmarked in isolation, as well as perhaps generating molecular precursors that could be decomposed to binary systems.

The chemistry of uranium-ligand multiple bonds is now well developed^10^, but in comparison that of thorium is sparse. Apart from a few carbene^11–18^, pnictidene^19–33^ and chalcogenido^34–39^ complexes, thorium-ligand multiple bonding remains overall quite rare. A transient zero-valent thorium synthon produced a thorium-amide from dinitrogen^40^, but whether this forms via a nitride or a biomimetic-type sequence of separate protonation and reduction steps is unknown, and only a few elegant species such as ThN, F_3_ThN, NThN, Th(N)_2_Th, and NThO have been trapped under cryogenic matrix isolation conditions^21,41,42^. In contrast, molecular uranium-nitrides^43^ were first prepared under matrix isolation conditions^42,44–49^, then a range of bridging nitrides were isolated and characterised^50–65^, or inferred from the products of transient species^66^, and then terminal U≡N triply bonded species were finally secured^67–71^. During publication of this work a bridging thorium-nitride was reported^72^, but isolation of a terminal molecular Th≡N triple bond under normal experimental conditions remains an unmet challenge.

Recently, utilising the triamidoamine ligand {N(CH_2_CH_2_NSiMe_2_Bu^t^)_3_}^3−^ (Tren^DMBS^), we reported attempts to prepare bridging diactinide-nitrides^73^. Although diuranium-nitrides [{U(Tren^DMBS^)}_2_(μ-N)]^0/1−^ in two different charge states could be prepared and isolated and were found to be robust, the corresponding dithorium-nitride [{Th(Tren^DMBS^)}_2_(μ-N)]^1−^ proved elusive and bridging parent imido complexes, formed from C–H bond activation reactions, were the only isolable species. Given that these diactinide-nitride species derive from azide precursors and are produced in concerted reactivity the formation of any Th≡N triple bond seems quite unlikely. An interesting feature of these bridging [{An(Tren^DMBS^)}_2_(μ-N)]^1−^ and terminal uranium-nitrides is that the σ-component of their An-N multiple bonds is higher in energy than the π-bonds, and this is also found for the uranyl dication but not bis(imido) analogues or other actinide-ligand multiple bonds more generally^74,75^. This phenomenon can be partly explained by anti-bonding interactions raising the energy of the σ-bond at short actinide-nitrogen distances. However, the bridging nitrides do not fit with that model. An alternative, more complete explanation may be the pushing-from-below phenomenon where the pseudo-core 6p-orbitals are engaged in repulsive interactions, principally with f-orbitals^76^. To date, pushing-from-below occurs with hard, charge-dense anions, such as O^2−^ and N^3−^, but it is the preserve of high oxidation state uranium or actinyl complexes so whether it is a specific or more periodic in nature phenomenon has remained an open question. Thus, we focussed our attention on the more sterically demanding ligand {N(CH_2_CH_2_NSiPr^i^_3_)_3_}^3−^ (Tren^TIPS^) to extend our search for a thorium-nitride since this ligand is the only ligand so far to support isolable terminal nitride linkages at uranium.

Here, we report findings that suggest that reduction of a thorium-azide supported by Tren^TIPS^ generates a transient Th≡N triple bond, but this is highly reactive and activates C–H bonds of aromatic solvents to give rare thorium parent imido complexes. However, we were able to effect nitride atom-transfer by incorporation into an organic amine heterocycle. Thus, we find that the Th≡N triple bond is inherently destabilised compared to U≡N triple bonds, which can be related to the greater covalency of uranium chemical bonding. The formation of parent imido complexes from a nitride with expulsion of alkyl during C–H activation presents some similarities to established actinide-nitride reactivity, but also introduces a contrasting mode of reactivity outcome since all prior related terminal uranium-nitride reactivity has resulted in 1,1-insertion to give an alkyl-amide or 1,2-addition to form an imido-alkyl combination. Calculations suggest that for the Th≡N bond the energy ordering of the Th–N bond components is *σ* > *π*, but for Th = NR it is generally *σ* < *π* with Th = NH potentially being either depending on the Th = NH geometry. This permits us to recognise that thorium as well as uranium might engage in pushing-from-below, suggesting that this phenomenon may play a wider and more common periodic role in actinide chemical bonding than previously thought.

## Results

### Synthesis and characterisation

**Fig. 1:**
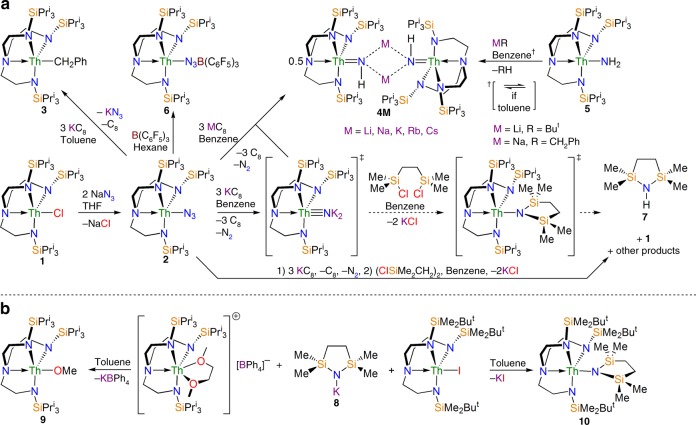
Synthesis of the compounds reported in this study. **a** Treatment of **1** with sodium azide produces the thorium-azide **2**, which when reduced in toluene with potassium graphite gives the known thorium-benzyl **3**. When the same reduction is conducted in benzene the thorium-imidos **4M** are isolated, and these complexes can alternatively be prepared by deprotonation of the thorium-amide **5**. Notably, when the deprotonation of **5** is conducted in toluene an imido/toluene-amide-benzyl equilibrium is established. Attempts to trap the thorium-nitride intermediate resulted in the isolation of the cyclic amine **7**. **b** Treatment of the potassium-amide **8** with a thorium separated ion pair gives the thorium-methoxide **9** whereas use of a less sterically demanding thorium-iodide gives the isolable thorium-amide **10**

Treatment of the thorium-chloride complex [Th(Tren^TIPS^)(Cl)] (**1**) with excess NaN_3_ affords the colourless thorium-azide complex [Th(Tren^TIPS^)(N_3_)] (**2**) in good (80%) isolated yield, Fig. 1a (see Supplementary Methods). The use of 1-^15^N-NaN_3_ gives the ^15^N-isotopologue (**2**^**15**^**N**) where the ^15^N-label is distributed Th-^15^N^14^N^14^N:Th-^14^N^14^N^15^N in a 1:1 ratio. This can be observed in the ATR-IR data of **2** and **2**^**15**^**N** (see Supplementary Fig. 1), which exhibit azide asymmetric stretching absorptions at 2086 and 2082/2070 cm^−1^, respectively, which compare well to the azide stretch of 2086 cm^−1^ for [U(Tren^TIPS^)(N_3_)]^70^ and other thorium-azides generally^77,78^. All other characterisation data for **2** and **2**^**15**^**N** are unremarkable and support for the proposed formulations (see Supplementary Figs. 2–4).

Reduction of **2** with an excess of KC_8_ in toluene gives full consumption of **2** as evidenced by NMR and ATR-IR spectroscopies. However, surprisingly, after work-up the sole thorium-containing product that is repeatedly isolated is the known thorium-benzyl [Th(Tren^TIPS^)(CH_2_Ph)] (**3**) (45% crystalline yield)^79^, Fig. 1a. All characterisation, including a single-crystal cell check, are in accord with published data, and additionally the formation of KN_3_ was evidenced by the presence of an azide asymmetric stretch at 2032 cm^−1^ in the ATR-IR spectrum, which is identical to that found for an authentic sample of KN_3_.

In contrast, when **2** is reduced with an excess of KC_8_ in benzene, the dimeric thorium-parent-imido [{Th(Tren^TIPS^)(μ-NHK)}_2_] (**4K**) is isolated in 50% yield, Fig. 1a. Using **2**^**15**^**N** we also prepared the 50% labelled ^15^N-isotopologue **4K**^**15**^**N**. Complex **4K** is isolated in similar yield when the reaction is conducted under argon, which together with the ^15^N-labelling confirms azide as the source of the imido nitrogen in **4K**. The N–H stretch could not be observed in the ATR-IR spectrum of **4K**, presumably because its asymmetric stretching mode is coupled to other vibrational modes in the molecule, which is a common feature in heavy-atom structures including the uranium congeners [{U(Tren^TIPS^)(μ-NHM)}_2_] (M = Li, Na, K, Rb, Cs)^80^. However, we were able to identify the Th = N stretch of **4K** at 575 cm^−1^, in perfect agreement with a computed value of 575 cm^−1^ from an analytical frequencies calculation, since in the corresponding spectrum of **4K**^**15**^**N** the absorption at 575 cm^−1^ is reduced in intensity and a band at 565 cm^−1^ has grown in, consistent with the presence of a 50% ^15^N-label (see Supplementary Figs. 5 and 6). Once isolated, the dimeric nature of **4K** renders it poorly soluble in non-polar solvents, and it decomposes in polar solvents, which has hampered its characterisation by NMR spectroscopy, but elemental analyses confirm the bulk formulation.

To establish the generality of the reaction that produces **4K**, we treated **2** separately with RbC_8_ and CsC_8_, isolating the corresponding dimeric thorium-parent-imido complexes [{Th(Tren^TIPS^)(μ-NHM)}_2_] (M = Rb, **4Rb**; Cs, **4Cs**) in crystalline yields of 30 and 20%, respectively, Fig. 1a. Like **4K**, **4Rb** and **4Cs** are poorly soluble once isolated which has prevented their full characterisation. In order to complete the **4M** series we targeted the Li and Na analogues. Unfortunately, reductions with these metals are more difficult to control, and intractable product mixtures were obtained. However, using the new thorium-parent-amide [Th(Tren^TIPS^)(NH_2_)] (**5**) – which is only the third example of a structurally authenticated Th–NH_2_ bond^40,72^, prepared here by reaction of NH_3_ with the known cyclometallate [Th{N(CH_2_CH_2_NSiPr^i^_3_)_2_(CH_2_CH_2_SiPr^i^_2_CHMeCH_2_)}]^79^ (see Supplementary Figs. 7–9) – we find that **5** can be deprotonated with strong bases; treating **5** with Bu^t^Li or NaCH_2_Ph in benzene affords [{Th(Tren^TIPS^)(μ-NHM)}_2_] (M = Li, **4Li**; Na, **4Na**) in 56 and 38% isolated yields, respectively, Fig. 1a. Interestingly, if toluene is used as the solvent in the preparation of **4Li** and **4Na**, an equilibrium between **4M** and **5**/MCH_2_Ph is established, suggesting that the **4M** series are capable of activating C–H bonds. Complexes **4Li** and **4Na** also suffer from low solubility once isolated, but in both cases the asymmetric imido N–H stretch with these lighter alkali metals can be observed in their respective ATR-IR spectra (**4Li**, 3393; **4Na**, 3397 cm^−1^). With the deprotonation of **5** established for **4Li** and **4Na**, we find that **4K**, **4Rb** and **4Cs** can also be prepared by deprotonation of **5** with MCH_2_Ph, but the yields are consistently low (av. 16%).

In related uranium chemistry, when [U(η^5^-C_5_Me_5_)_2_{(N(SiMe_3_)_2_}(N_3_)] is photolysed it produces the insertion product [U(η^5^-C_5_Me_5_)(η^5^:κ^1^-C_5_Me_4_CH_2_NH){N(SiMe_3_)_2_}] via a transient nitride^66^. On the other hand, reaction of [N_3_B(C_6_F_5_)_3_][NBu^n^_4_] with [U{NBu^t^C_6_H_3_-3,5-Me_2_}_3_] produced [(C_6_F_5_)_3_BNU{NBu^t^C_6_H_3_-3,5-Me_2_}_3_], which can be formulated as either a borane-capped nitride or an imido-borate^56^. Therefore, we treated **2** with B(C_6_F_5_)_3_ in hexane and isolated [Th(Tren^TIPS^)(μ-N_3_)B(C_6_F_5_)_3_] (**6**) in 62% isolated yield, Fig. 1a. The characterisation data for **6** (see Supplementary Figs. 10–14) support its formulation, with a diagnostic azide stretch at 2171 cm^−1^ observed in its ATR-IR spectrum, which compares very well to an azide stretch of 2178 cm^−1^ reported for [U(η^5^-C_5_Me_5_)_2_{(N(SiMe_3_)_2_}(μ-N_3_)B(C_6_F_5_)_3_]^66^. However, **6** does not liberate N_2_ when reduced by KC_8_, with or without crown ethers or 2,2,2-cryptand, and instead borane-centred decomposition occurs, presumably involving fluoride extraction which is common for fluorinated-boranes. Treating **2** with excess KC_8_ in the presence of B(C_6_F_5_)_3_ to generate a nitride and then trap it results in the same outcome. Further, complex **6** does not react under photolytic conditions (125 W Hg-lamp) – which efficiently converts [U(Tren^TIPS^)(N)] to the nitride-insertion product [U{N(CH_2_CH_2_NSiPr^i^_3_)_2_(CH_2_CH_2_SiPr^i^_2_CMe_2_NH)}]^68^ – most likely reflecting the fact that **6** does not contain any localised metal valence electrons to transfer to the azide π*-orbitals. Seeking to provide an electron source, we photolysed **6** in the presence of KC_8_ but again borane-centred decomposition occurred. Thus, the borane capping would seem to deactivate the azide linkage in **2**, as it does also for [U(η^5^-C_5_Me_5_)_2_{(N(SiMe_3_)_2_}(μ-N_3_)B(C_6_F_5_)_3_]^66^.

The isolation of the **4M** series suggests that [Th(Tren^TIPS^)(NM_2_)] is initially formed, which is credible given the prior synthesis of the uranium-arsenido complex [{U(Tren^TIPS^)(AsK_2_)}_4_]^81^. For [{U(Tren^TIPS^)(AsK_2_)}_4_], abstraction of K ions results in K–H exchange to form arsinidiide, U = AsHK, linkages, presumably because the K ions perform a key, charge-stabilising role. It can be surmised that the same process occurs with [Th(Tren^TIPS^)(NM_2_)], since Th is generally expected to bond more ionically than uranium giving a more polar, charge-rich and reactive nitride, which then spontaneously exchanges K for H to give the imido complexes **4M**. We therefore sought to trap [Th(Tren^TIPS^)(NK_2_)] using (ClSiMe_2_CH_2_)_2_, to eliminate KCl and produce [Th(Tren^TIPS^){N(SiMe_2_CH_2_)_2_}]. However, stepwise or one-pot reduction of **2** and treatment with (ClSiMe_2_CH_2_)_2_ resulted in the formation of **1** and HN(SiMe_2_CH_2_)_2_ (**7**), Fig. 1a, both identified by comparison of reaction mixture NMR data with characterisation data on authentic pure samples (see Supplementary Figs. 15 and 16). Nonetheless, the formation of **7** suggests that [Th(Tren^TIPS^)(NK_2_)] forms and then converts to [Th(Tren^TIPS^){N(SiMe_2_CH_2_)_2_}], but then the latter decomposes effecting, overall, nitride atom-transfer into an organic heterocycle. We suggest that the bulky Tren^TIPS^ ligand would sterically clash with the four silyl methyl groups of the heterocycle in the putative [Th(Tren^TIPS^){N(SiMe_2_CH_2_)_2_}] excessively straining and thence rupturing the Th–N_heterocycle_ bond. Supporting evidence for this is provided by the observation that the known cyclometallate [Th{N(CH_2_CH_2_NSiPr^i^_3_)_2_(CH_2_CH_2_SiPr^i^_2_CHMeCH_2_)}]^79^ does not react with HN(SiMe_2_CH_2_)_2_, presumably on steric grounds given NH_3_ reacts with the former to give **5**. Further, we prepared the potassium salt [{KN(SiMe_2_CH_2_)_2_}_2_] (**8**) (see Supplementary Figs. 17–19) and reacted it with [Th(Tren^TIPS^)(DME)][BPh_4_]^31^, which has proven itself an excellent precursor to forming thorium-group 15 element bonds. However, the only thorium-containing product that could be isolated was the methoxide [Th(Tren^TIPS^)(OMe)] (**9**) (see Supplementary Figs. 20–22), Fig. 1b; this indirectly suggests, though not definitively, that [Th(Tren^TIPS^){N(SiMe_2_CH_2_)_2_}] may form but be so reactive as to cleave the DME by-product. Lastly, we find that reaction of **8** with the sterically less encumbered [Th(Tren^DMBS^)I] results in the target amide complex [Th(Tren^DMBS^){N(SiMe_2_CH_2_)_2_}] (**10**) being isolated in 76% yield (see Suppupplementary Figs. 23–25), Fig. 1b, so there is nothing inherently unstable about the Th–N_heterocycle_ linkage in a Tren-ligand environment so long as the Tren ligand is not too sterically demanding. We therefore conclude that reduction of **2** generates the thorium-nitride [Th(Tren^TIPS^)(NK_2_)], but this either degrades to the parent imidos **4M**, or can be converted to the sterically overloaded amide [Th(Tren^TIPS^){N(SiMe_2_CH_2_)_2_}], but this in turn decomposes to **7**.

### Solid state structures

**Fig. 2:**
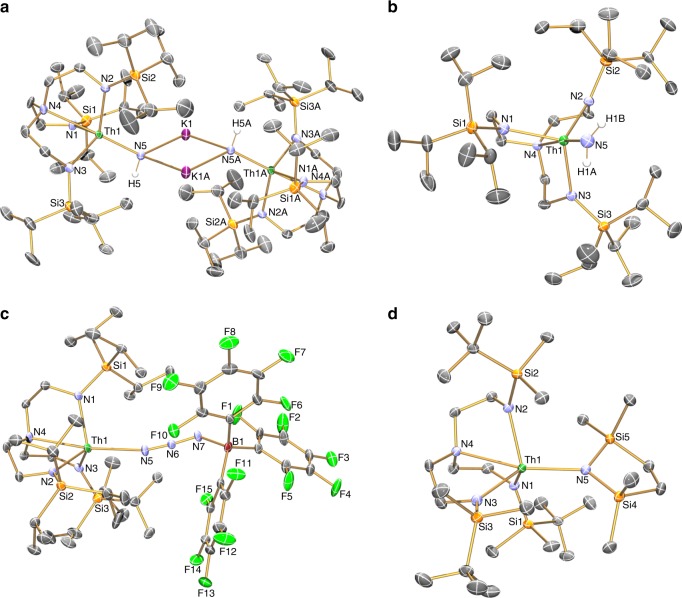
Molecular structures of **4K**, **5**, **6** and **10** with selective labelling. **a** the parent imido dimer **4K**. **b** the parent amide **5**. **c** the borane-capped azide **6**. **d** the amide-heterocycle **10**. The data for these complexes were collected at 150 K, displacement ellipsoids are presented at 40% probability and non-imido hydrogen atoms and minor disorder components are omitted for clarity

We determined the solid state structures of **2**, **4M**, **5**, **6**, and **8**–**10** and **4K** (see Supplementary Figs. 26–36), and **4K**, **5**, **6** and **10** are shown in Fig. 2. All of the thorium complexes contain trigonal bipyramidal thorium ions with a single functional group linkage in the pocket defined by the Tren^TIPS^ ligand. For **2**, the Th–N_azide_ distance of 2.365(3) Å is similar to the three Th–N_amide_ distances (range: 2.301(3)–2.317(3) Å) and the Th–N_amine_ distance is as expected longer at 2.673(3) Å, which for the Tren-related distances are similar to those observed for Th-Tren^TIPS^ complexes with P- and As-donor ligand sets^31,32^. The Nα-Nβ and Nβ-Nγ distances of 1.200(4) and 1.150(4) Å, respectively, are only marginally distinct, suggesting a slight prevalence of the N≡N^+^–N^2−^ resonance form of azide over the ^−^N = N^+^ = N^−^ form induced by coordination to the thorium ion, and hence only weak azide activation.

For the imido series **4M**, as Group 1 is descended from Li to Cs the Th–N_imido_ distance becomes shorter overall, though after Li the heavier alkali metal distances are statistically indistinguishable (**4Li**, 2.209(2); **4Na**, 2.158(5); **4K**, 2.147(4); **4Rb**, 2.149(6); **4Cs**, 2.105(13) Å). This suggests that the donor capacity of the imido with respect to thorium is dictated in part by how charge attracting the alkali metal is, so with the most polarising metal Li the imido experiences the greatest charge depletion and is thus the poorest donor. A similar trend was observed for uranium analogues^80^. According to Pyykkö, the sum of the single and double bond covalent radii of thorium and nitrogen are 2.46 and 2.03 Å^82^, respectively; despite their bridging natures, the Th = N distances above are closer to the double than single bond values suggesting multiple bond interactions, and they compare favourably to terminal Th–N_imido_ distances that span the range 2.034–2.165 Å^19,20,22,23,26,28–30^. The strong donor nature of the imido units in **4M** is also suggested by the Th–N_amine_ bond lengths, which are now ~2.745 Å, some 0.07 Å longer than the analogous distance in **2**. This most likely reflects a *trans*-influence in the N_imido_–Th–N_amine_ linkages, which are all ~175°, and thus close to optimally aligned for a *trans*-influence to exert itself. The Th–N_amide_ distances in the **4M** series now average 2.385 Å, nearly 0.09 Å longer than in **2**, most likely reflecting the sterically congested nature of these dimers coupled to the presence of the strong imido donor ligands.

The parent amide character of **5** versus the imido nature of the **4M** series is reflected by a Th–N distance of 2.290(4) Å in **5**, which is 0.08–0.19 Å longer than the Th-imido distances, though the parent amide is clearly a stronger donor than the azide in **2** since the Th–N_amine_ distance of 2.710(3) Å is between the Th–N_amine_ distances for **2** and the **4M** series.

Of all the complexes reported here, **6** reports perhaps the greatest structural changes. The coordination of the borane to azide clearly renders the azide an extremely poor donor as evidenced by the very long Th–N_azide_ distance of 2.551(6) Å. Likely in response to this, the Th–N_amide_ (2.275(6)–2.286(5) Å) and Th–N_amine_ (2.652(6) Å) distances are much shorter than the analogous distances in **2**, the **4M** series, and **5**. The Nα-Nβ and Nβ-Nγ distances of 1.160(8) and 1.173(8) Å are now statistically indistinguishable from one another (c.f. **2**) suggesting that the ^−^N = N^+^ = N^−^ resonance form now dominates in this bridging azide.

Lastly, the structure of **10** reveals a Th–N_heterocycle_ distance of 2.385(2) Å that is slightly longer than the other three Th–N_amide_ distances (2.310(2)–2.335(2) Å). The N_amine_–Th–N_heterocycle_ angle is 165.68(7)°, distorted ~10° from the ~175° N_amine_–Th–Naxial-_amide/-imido_ angle observed in **2**, **4M**, **5** and **9**. Also, the N-heterocycle is coordinated in a skewed manner to the thorium ion, as evidenced by Si-N_heterocycle_-Th angles of 113.77(10) and 141.23(11)°, with the remaining Si–N_heterocycle_–Si angle of 104.42(11)° completing the trigonal planar geometry of the heterocycle amide. Taking these data together, along with the fact the Tren^TIPS^ analogue has not been isolated, suggests that **10** is close to the limit of steric saturation and thence Tren^TIPS^ would be beyond that limit.

### Computational reaction profiling studies

The reaction of **2** with KC_8_ to give **3** has the appearance of KN_3_ elimination with a thorium(III) intermediate activating toluene, but it stands in contrast to the production of **4K** when the reaction is carried out in benzene. The latter invokes a nitride intermediate, and since C–H bond activation occurs in both reactions we computed possible reaction mechanism profiles in simulated benzene or toluene solvent media at the DFT level (B3PW91) in order to ascertain whether distinct paths are followed or whether there is any commonality to these seemingly unrelated reaction outcomes.

**Fig. 3:**
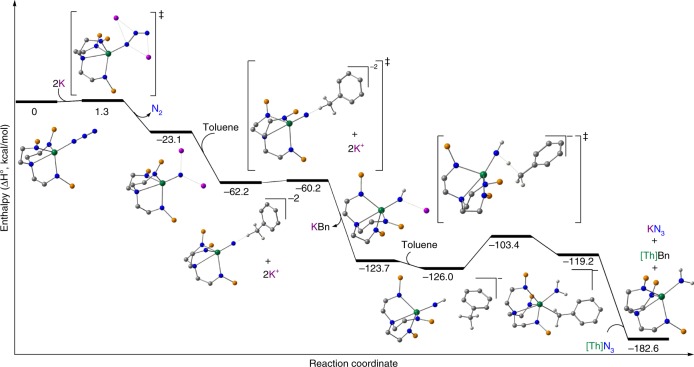
Computed reaction profile for the reduction of **2** by potassium in toluene. This pathway accounts for the reduction of the azide to nitride then subsequent protonation from toluene solvent to give a parent imido which reacts further with toluene to give parent amide **5** along with benzyl **3** and potassium-azide in-line with experimental findings. The iso-propyl groups of the silyl substituents are omitted for clarity. In this reaction scheme potassium rather than potassium graphite was used to give a tractable calculation

**Fig. 4:**
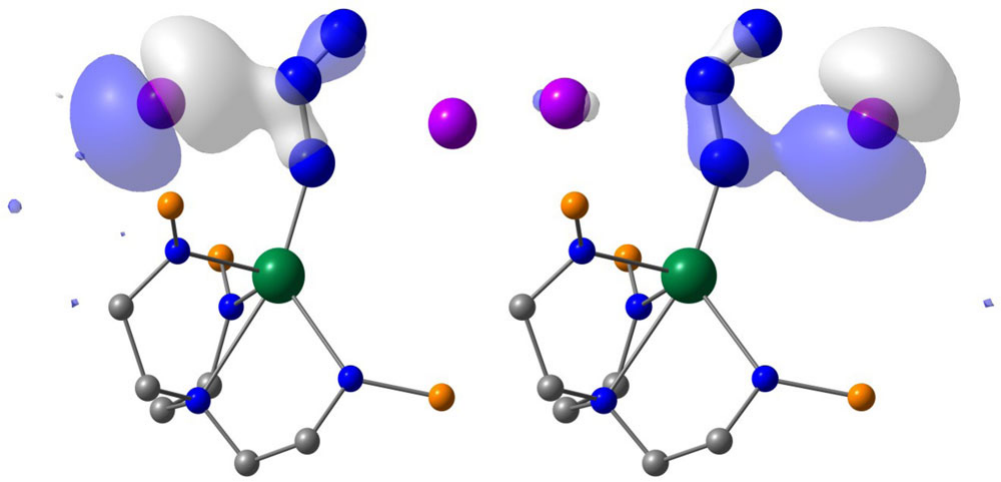
Views of the potassium-azide stabilising interactions. These are both in the first transition state of Figs. 3 and 5 at 1.3 kcal/mol as the azide is activated and extrudes N_2_. These frontier orbitals alternatively highlight the interaction of the azide unit with vacant sp-hybrid orbitals of the two potassium ions following electron transfer from the latter into the π*-orbitals of the former. The iso-propyl groups of the silyl substituents are omitted for clarity

The first step of the computed mechanism for the reaction in toluene, Fig. 3, involves the formation of a nitride through the KC_8_ mediated reduction. Adding the alkali metal leads to a low-lying transition state (TS) with an associated barrier of 1.3 kcal/mol that corresponds to the release of dinitrogen from **2**. The involvement of potassium in this step is crucial. Indeed, when this same step is computed without potassium ions an extremely high energy barrier (85.6 kcal/mol) is found which clearly would impede nitride formation, and this is in-line with previous uranium-azide chemistry that promoted the elimination of N_2_ also with potassium as a reductant^70^ or by photolytic oxidation^66^. Moreover, potassium coordination not only changes the geometry of the coordinated azide ligand from linear to bent, needed to release N_2_, but also empty hybrid sp-orbitals of potassium are involved in the frontier orbitals of the TS, Fig. 4, which assist in stabilising charge accumulation within the azide unit as it is reduced.

Following the reaction coordinate yields a thorium nitride capped by two K^+^ ions as the newly formed N_2_ molecule is expelled, with a global energy gain of 23.1 kcal/mol, Fig. 3. Natural Bond Order (NBO) analysis show the presence of a polarised Th≡N triple bond with one *σ*- and two *π*-bonding orbitals and a Wiberg Bond Index (WBI) of 1.56 (0.57 in the Th–N bond from the initial thorium-azide complex), indicative of a strongly polarised bond. The resulting nitride activates the C–H bond from toluene in an irreversible exothermic step with a 2-kcal/mol barrier. This step is best described as a proton transfer from the CH_3_ group of toluene to the nucleophilic nitride. Indeed, this reaction is an outer-sphere process indicating that only the nucleophilicity of the nitride is needed to achieve the process. Analogously, the resulting benzyl anion does not bind the metal centre, but instead extracts a potassium ion from the nitride, yielding an imido intermediate [Th(Tren^TIPS^)(NHK)]. NBO analysis of this imido complex indicates the presence of a Th = N double bond interaction and the WBI of 1.04 is in line with the results found for the formal polarised single and triple Th–N bonds. Interestingly, the imido complex can activate another C–H bond from a toluene molecule. This process involves a barrier of 22.6 kcal/mol that is ~20 kcal/mol higher than the first proton transfer. This is because the imido is less nucleophilic than the nitride, as evidenced by the geometry of the TS which resembles a classical σ-bond metathesis. In the same way, the final product of this step involves the coordination of the benzyl group to the metal centre rather than the extraction by the second potassium. This step is slightly endothermic by 6.8 kcal/mol and is in line with the experimental observation that the C–H bond activation of toluene is reversible in toluene, establishing an imido(**4K**)/toluene-amide(**5**)/benzyl equilibrium, when preparing **4K** from benzyl potassium, for example. The system can evolve with another instance of the thorium-azide complex **2** that can extract the benzyl ligand to generate thorium-benzyl **3** and liberate the thorium-amide **5** and KN_3_.

**Fig. 5:**
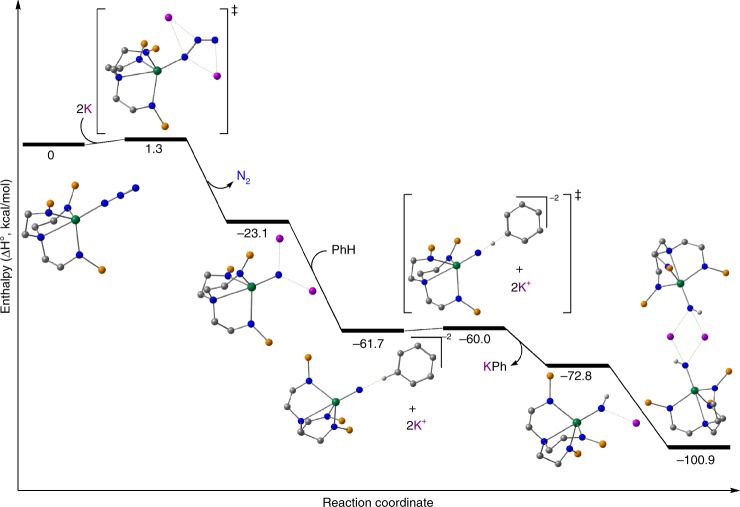
Computed reaction profile for the reduction of **2** by potassium in benzene. This pathway accounts for the reduction of the azide to nitride then subsequent protonation from benzene solvent to give a parent imido, which dimerises in-line with experimental findings. The iso-propyl groups of the silyl substituents are omitted for clarity. In this reaction scheme potassium rather than potassium graphite was used to give a tractable calculation

Modelling the reaction with benzene instead of toluene gives similar results for the initial steps, Fig. 5, including a low activation barrier for the first C-H bond activation of 1.7 kcal/mol. However, the formation of KPh, whilst still exothermic, is not as favourable, with an energy gain of 11.1 kcal/mol as opposed to the 63.5 kcal/mol from the reaction with toluene. Mirroring the mechanism with toluene, a second C–H bond activation from another benzene molecule was studied, to form the thorium-amide **5**, however, the intermediate, which retains the amide and phenyl ligands, is ~100 kcal/mol higher in energy, ruling out this reaction. Therefore, dimerisation of the imido intermediate [Th(Tren^TIPS^)(NHK)] is preferred, yielding the stabilised and experimentally obtained imido dimer **4K**.

The formation of the imido series **4M** partially mimics the known reactivity of terminal uranium-nitrides and a transient bridging dithorium-nitride. When uranium(VI)-nitrides react with C–H bonds, always in an intramolecular fashion with ancillary ligands to date, uranium(IV)-amides are formed where the nitride has undergone a 1,1-insertion with a C–H bond^66,68^. A transient uranium(IV)-nitride also activates ancillary-ligand C–H bonds, but instead of undergoing a 1,1-insertion a parent imido-alkyl is formed by 1,2-addition to avoid the formation, formally, of uranium(II)^83^. Our previous work on [{Th(Tren^DMBS^}_2_(μ-N)]^1−^ showed that the transient nitride is converted to a bridging imido and either potassium alkyl is eliminated or intramolecular Tren-activation occurs depending on the availability of the potassium counter-ion^73^. The chemistry reported here is distinct to the prior uranium and thorium chemistry because although parent imido groups are formed the alkyl component is always eliminated with potassium; certainly thorium(II) is a very difficult oxidation state to access and together with the steric demands of Tren^TIPS^ 1,1-insertion and 1,2-additions would seem to be disfavoured. This suggests that the transient thorium-nitride is a highly polar and thus reactive group, which is confirmed by the following computational studies, and the observed reactivity here complements but also contrasts to established modes of uranium-nitride reactivity introducing an alternative nitride reactivity outcome.

### Computational modelling of the thorium-parent-imido and -nitride bonds

Since the reaction profile calculations suggest the presence of a common Th≡N triple bond intermediate and experimentally the imido series **4M** is isolated we have carried out a detailed examination of the **4M** complexes and the potassium-free dianion intermediate [Th(Tren^TIPS^)(N)]^2−^ (**11**) using DFT calculations. In order for these calculations to be meaningfully consistent with our prior work these calculations were performed in the gas-phase at the BP86 TZP level with an all-electron basis set and ZORA. For completeness, we also provide a comparison to the hypothetical uranium(IV)-nitride [U(Tren^TIPS^)(N)]^2−^ (**12**) along with previously reported [U(Tren^TIPS^)(N)]^1−^ (**13**)^67^ and [U(Tren^TIPS^)(N)]^0^ (**14**)^68^ to enable meaningful benchmarking. Where experimental comparisons are possible, the geometry optimised structures of the computed complexes are in close agreement with the solid state structures, Table 1 and Supplementary Tables 1–38, so we conclude that these models provide qualitatively meaningful pictures of the electronic structure of these compounds.

**Table 1:** Selected computed DFT, NBO, and QTAIM data for 2, 4M, 5, and 11–14

	Bond lengths and indices		MDC atomic charges		NBO σ-component^f^			NBO *π*-component^*f*^			QTAIM parameters^g^			
Entry^a^	An-N^b^	BI^c^	*Q*_*An*_^d^	*q*_N_^e^	%An	%N	An s:p:d:f	%An	%N	An s:p:d:f	ρ(r)	∇^2^ρ(r)	*H*(r)	*ε*(r)
**2**	2.355	0.55	1.48	−0.7	6	94	7:1:48:44	–	–	–	0.07	0.19	−0.06	0.00
**4Li**	2.237	0.87	1.57	−1.02	7	93	2:1:49:48	8	92	1:2:53:44	0.11	0.19	−0.05	0.18
**4Na**	2.191	0.92	1.65	−1.14	8	92	2:1:49:48	10	90	1:1:57:41	0.12	0.22	−0.07	0.16
**4K**	2.183	1.24	1.66	−1.23	8	92	1:2:45:52	10	90	2:1:51:46	0.12	0.22	−0.07	0.17
**4Rb**	2.165	1.26	1.71	−1.26	8	92	1:2:44:53	10	90	2:1:51:56	0.13	0.24	−0.07	0.17
**4Cs**	2.161	1.29	1.72	−1.25	7	93	1:2:44:53	10	90	2:1:52:48	0.13	0.24	−0.07	0.16
**5**	2.311	0.54	1.51	−1.08	0	100	–	8	92	0:0:42:58	0.09	0.21	−0.04	0.33
**11**	1.925	2.88	1.90	−1.29	25	75	12:5:33:50	13	87	0:0:52:48	0.23	0.15	−0.23	0.25
**12**	1.832	2.91	1.89	−1.29	34	64	2:2:16:80	26	74	0:0:24:76	0.28	0.11	−0.34	0.16
**13**	1.810	2.91	3.34	−1.36	32	68	5:4:44:47	26	73	0:0:28:72	0.29	0.24	−0.27	0.01
**14**	1.779	2.92	3.79	−1.35	41	59	1:1:9:89	30	70	0:0:19:81	0.39	0.21	−0.30	0.06

^a^All molecules geometry optimised without symmetry constraints at the BP86 TZP/ZORA level

^b^Calculated An-N distances (Å)

^c^Mayer bond indices

^d^MDC-q charges on An metal

^e^MDC-q charges on nitrogen

^f^Natural Bond Orbital (NBO) analyses

^g^QTAIM topological electron density [*ρ*(r)], Laplacian [∇²*ρ*(r)], electronic energy density [*H*(r)], and ellipticity [*ε(r)*] bond critical point data

For all complexes, the computed Mayer bond orders are ~0.7 and ~0.2 for the Th–N_amide_ and Th–N_amine_ linkages, respectively. In comparison, the Th–N_azide_ and Th–N_amide_ bond orders of 0.55 and 0.54 for **2** and **5** confirm their formal single bond status. As expected, the Th–N_imido_ bond orders are substantially larger, at 0.87–1.29 reflecting their polarised multiple bond interactions. Notably, on moving from lithium to caesium the Th–N_imido_ bond order increases, consistent with the more polarising lithium depleting charge from the imido group as also suggested by the solid state Th–N_imido_ bond lengths. Even though **11** is a charge-rich dianion, a Th–N_nitride_ bond order of 2.88 suggests a Th≡N triple bond. Interestingly, the analogous bond order for **12** of 2.91 is only marginally larger than that of **11**, and on moving from uranium(IV) (**12**) to (V) (**13**) to (VI) (**14**) the U–N_nitride_ bond order only modestly increases to 2.92 for the latter. The computed multipole derived (MDC_q_) charges for the metal and amide/imido/nitride centres are in-line with their formulations, though we note that the metal charges for **11** and **12** are higher than for their imido cousins, suggesting that the nitrides exhibit charge accumulation at the nitride centres, which for **11** is certainly consistent with the experimentally observed reactivity.

**Fig. 6:**
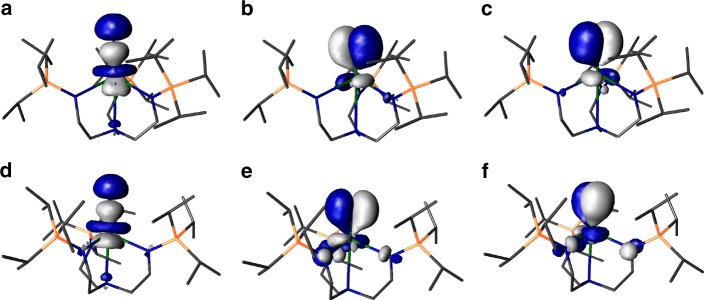
Frontier molecular orbitals of [M(Tren^TIPS^)(N)]^2−^ (M = Th, **11**; M = U, **12**). **a** HOMO of **11** (221, 3.382 eV), **b** HOMO−1 of **11** (220, 2.757 eV), **c** HOMO−2 of **11** (219, 2.746 eV), **d** α-spin HOMO−2 of **12** (221a, 1.679 eV), **e** α-spin HOMO−3 of **12** (220a, 1.215 eV), **f** α-spin HOMO−4 of **12** (219a, 1.200 eV). The HOMO (223a, 3.921 eV) and HOMO−1 (222a, 3.824 eV) in the α-spin manifold of **12** are of essentially pure, non-bonding 5 f orbital single electron character. Hydrogen atoms in these all-electron DFT calculations are omitted from these visualisations for clarity

The Kohn Sham frontier molecular orbitals of these complexes (see Table 1, Supplementary Figs. 37–41, and Fig. 6) reveals several notable phenomena. As expected, **2** and **5** exhibit single covalent Th–N_azide/amide_ *σ*^2^-bonds that are supplemented by weak, dative *π*-interactions from the N-ligands. For **4M**, **11** and **12** σ^2^-*π*^2^-*π*^2^-triple bond interactions are apparent; these are certainly polarised and not optimally aligned due to symmetry constraints for the **4M** series, and although less polarised the triple bonds of **11** and **12** are clearly fairly N-centred consistent with the computed charges and the experimental reactivity. The frontier orbitals of **11** and **12** are visually strikingly similar to each other, and appear more polarised than **13** and **14** where more covalent interactions are present by virtue of the uranium(V) and (VI) ions, respectively, in those complexes.

For computed **11** and **12** the An-N σ-bonds are clearly resolved and higher in energy than the two quasi-degenerate An-N *π*-bonding contributions. The situation is not as clear-cut for the **4M** imidos because the deviation of the Th centres from the M_2_N_2_ rings leads to non-ideal bonding alignment, but in each case the HOMO approximates to a skewed Th–N_imido_ *σ*-bond with more clearly resolved Th–N_imido_ π-MOs lower in energy. The *σ* > *π* phenomenon is known for uranyl^74–76^, **13** and **14**^67,68^, and [{An(Tren^DMBS^)}_2_(μ-N)]^1−^ (An = U, Th)^73^, but it is not found in U = C, mono-U = O, and U = NR complexes generally, including closely related [U(Tren^TIPS^)(NH)][K(15C5)_2_]^80^. To ascertain whether **4M** are outliers, we computed the electronic structures of [Th(Tren^TIPS^)(NR)]^1−^ (R = H, 1-adamantyl, Ph, SiMe_3_) and in all instances the calculations return a *π* > *σ* energy ordering like uranium analogues and other thorium-imidos (see Supplementary Figs. 42–45). For a bond defined as being along a z-axis, a σ > π energy ordering is usually rationalised on the basis that at short U–E (E = N, O) distances the σ-orientated p_z_-orbital is at such close-approach that it experiences anti-bonding interactions with annular 5f- and 6d-orbital lobes thus raising it above the π-bonds. To date this has been the hallmark of high oxidation state uranium-element multiple bonds with high bond orders and short U-E distances. Whilst **11** and **12** contain high bond order Th-/U-N bonds, they are lower (IV) oxidation states than **13** and **14** and have longer nitride bond distances so would not be expected to have this *σ* > *π* energy ordering. It may be that the longer Th–N distances, compared to uranium analogues, may be off-set or even outweighed by the greater radial distribution of thorium 5f- and 6d-orbitals compared to uranium, on a like-for-like basis, due to a lower effective nuclear charge of the former compared to the latter.

An alternative explanation that would account for our data is that these systems are manifesting the pushing-from-below phenomenon where the pseudo-core occupied 6p-orbitals are engaged in repulsive, polarising interactions with occupied f-orbitals or hybrids with f-character^74–76^. However, this has previously been restricted to uranium, and indeed it has been pointed out that its occurrence for thorium has been previously dismissed because of the bent nature of ThO_2_ vs linear uranyl^76^. It is unrealistic to expect thorium 6p-orbital contributions to be localised in any particular molecular orbital due to the delocalised nature of molecular orbital theory and so this aspect of the calculations cannot be extracted directly, but these calculations use an all-electron basis set and so the 6p-electrons are explicitly treated in the calculations. However, in order to probe the involvement, or not, of 6p-orbitals in the observed *σ* > *π* energy ordering we performed two further sets of calculations (see Supplementary Figs. 46–49). Firstly, instead of using exclusively all-electron basis sets we geometry optimised **11** and **12** using a frozen core up to and including the 5d-level for the Th and U ions. This results in virtually no change to valence orbital energies (*Δ*_max_ ≤ 0.01 eV), and thus the *σ*–*π* energy gaps of **11** and **12**, and so this establishes that the frozen core has not perturbed the valence electronic structure. We then geometry optimised the structures of **11** and **12** with Th and U frozen cores up to and including the 6p-level. For both **11** and **12** this results in the metal-nitride distances lengthening (by 0.09 and 0.05 Å for **11** and **12**, respectively), and thus the σ- and π-components of the An≡N triple bonds are destabilised and rise in energy as a group. However, the *σ*-components of the An≡N triple bonds are stabilised relative to the *π*-components, and for **11** the *σ*–*π* gap decreases from 0.621 to 0.439 eV and for **12** it decreases from 0.450 to 0.270 eV. To put this another way, taking the 6p-orbitals out of the frozen core and placing them in the valence region results in shorter An≡N triple bonds with destabilised *σ*-bonds relative to the π-components. These observations, consistent with prior work on {UO_2_}^2+^ and related systems^74–76^, suggest that pushing-from-below involving 6p-orbitals may be operating in **11** and **12**. This may seem strange for **11** at first given the dominance of 6d-orbital character in the bonding of thorium, but in **11** thorium bonds with 5f-6d hybrids (see NBO discussion below). It is interesting to note that the reduction in the *σ*–*π* gaps for **11** and **12** are 71 and 60%, respectively. This suggests that there is slightly greater pushing-from-below for uranium than thorium, and indeed the ~0.18 eV *σ*–*π* energy gap changes of tetravalent **11** and **12** compare to changes of ~1 eV for the σ_u_ HOMO of hexavalent {UO_2_}^2+^ when the 6p-orbitals of uranyl are frozen. This would suggest that the 6p-5f energy gap for Th(IV) and U(IV) might be expected to be larger than that of U(VI), and so whilst still present the pushing-from-below phenomenon would be diminished with the former ions. Indeed, calculations on U(VI), U(V), U(IV), and Th(IV) ions in the gas-phase return 6p-5f energy gaps of 19.0, 21.1, 22.9, and 23.2 eV, respectively, where the 6p-5f energy gap is taken as the difference between the fully occupied β-spin 6p and fully unoccupied 5f-orbitals. Whilst 6p-5d interactions cannot be ruled out from contributing to pushing-from-below, we note that it is the 6p-5f interactions that are most pronounced on the basis that f-p overlap tends to be better than d-p overlap for early actinides, and certainly **12** has more 5f character in its An≡N bond than **11** which has more 6d character^74^. We note, however, that 6p-orbital energies would be raised in anions such as **11** and **12** perhaps favouring 6p-5f interactions. We also note that pushing-from-below tends to occur when hard, polarising anions are coordinated to An-metals, and certainly O^2−^ and N^3−^ are hard, charge-dense anions. PhN^2−^, Me_3_SiN^2−^, and 1-adamantyl-N^2−^ can be considered to be softer donors than nitrides and oxides. For linear HN^2−^ the presence of the H atom along the *σ*-axis would be expected to diminish the 6p-5f interaction that raises the σ-MO energy in the same way as is found for {H_3_P-N = U = N-PH_3_}^4+^^74^. However, this linear arrangement is not found for the **4M** series consistent with the observations above. A similar argument applies for the variation of *σ* and *π* frontier orbital energies for uranyl vs bis(imido) complexes^84^. Looking more widely, we note that certain group 6 nitrides are computed to have σ > π energy ordering^68^, the thorium-oxo [Th(η^5^-C_5_H_2_Bu^t^_3_)_2_(O)] appears to have a weak Th-O σ-amplitude in its HOMO with π-interactions lower in energy^34^, and indeed more broadly this is experimentally determined to be the case for N_2_ also. Interestingly, for molecules like N_2_ the 2s-2p energy gap is ~12 eV, whereas the 6p-5f gaps for the naked actinide ions are computed to be ~22 eV. Of course these 6p-5f energy gaps will change upon complexation, but it is likely that they are still larger for the actinides, which suggests that the respective orbital overlap efficiencies could be a contributing factor. When taken together, noting the above examples span the p-, d-, and f-blocks, this emerging picture suggests that there may be a *σ*/*π* ligand field strength and metal identity/oxidation state energy ordering phenomenon that is more periodic and commonly applicable than currently is appreciated.

From an energetic perspective, we note that **11** contains the highest-lying and thus most destabilised An≡N interactions of **11**–**14**. The σ/2π energies (eV) for **11**–**14** are 3.382/2.757/2.746, 1.679/1.215/1.200, −1.261/−1.547/−1.552, and −5.173/−5.644/−5.668, respectively. Whilst the decreasing energies for **12**–**14** are as would be expected for the decreasing formal charge of these nitrides and the increasing oxidation state of uranium, since nitrides inherently stabilise high oxidation state metal ions, and it follows that they are ill-suited to mid- and low-oxidation state metals, it is notable that for **11** vs **12** the Th–N_nitride_ σ orbital is destabilised by ~1.7 eV compared to the analogous interaction for **12** and the π-bonds are destabilised by ~1.5 eV for **11** compared to **12**. A similar situation was also found for [{Th(Tren^DMBS^)}_2_(μ-N)]^1−^ vs [{U(Tren^DMBS^)}_2_(μ-N)]^1−^ ^74^, though in that case the destabilisation of thorium compared to uranium was not so great. The significant destabilisation of the frontier orbitals of the Th–N_nitiride_ bond in **11** is in-line with the experimental paucity of terminal Th≡N triple bonds generally, noting that there are few examples even in matrix isolation experiments^21,35,36^, the observed C–H activation chemistry^73^, and the generally more covalent nature of uranium bonding compared to thorium.

NBO analysis further supports the polarised bonding picture for the Th–N bonds in **4M** and **11**, but confirms the multiple bond formulations of these complexes. The **4M** imido complexes have highly polar Th = N_imido_ bonds with ~10% thorium contribution to the *σ*- and *π*-bonds, and, perhaps surprisingly considering the often found dominance of 6d over 5f orbital character of thorium bonding, the thorium contributions to these bonds are reasonably balanced with ~1:1 5f:6d contributions making up the vast (>97%) majority of these components with only modest (<3%) 7s and 7p intrusions. For **11**, the thorium now plays a substantially larger (25%) role in the σ-bonding to the nitride, but, mirroring the visualised Kohn Sham orbitals, only 13% in the *π*-bonds suggesting that the latter are quite polar and N-centred. Interestingly, for the nitride **11** thorium 5f character now starts to dominate over 6d in the *σ*-component though they are balanced ~1:1 in the *π*-bonds. This contrasts to the situation for **12** where the anticipated greater covalency of uranium is reflected by greater uranium contributions to the *σ-* and *π*-bonds and within those contributions dominant 5f character of the bonding. As expected, as the uranium oxidation state continues to increase the U–N_nitride_ bonds become more covalent with greater uranium contributions.

DFT and NBO approaches are orbital-based, so to examine the bonding of these complexes further we used QTAIM since this probes the bonding topology, i.e. the electron density, at the bond critical point (BCP) which occurs when a bonding interaction is present^85^. For **4M**, **11**, and **12** An-N_imido_ and An-N_nitride_ 3,−1-BCPs were found in all cases. At a BCP the electron density, *ρ*(r) tends to be >0.2 for a covalent bond, and 0.1–0.2 for a polarised-covalent bond; a lower value tends to represent an ionic interaction. Thus, the BCP *ρ*(r) values for **2** and **5** are consistent with predominantly electrostatic bonds, whereas for the **4M** series, they are polarised-covalent. The nitrides **11**–**14** are all >0.2 indicating covalent bonds in-line with their covalent triple bond formulations. The more covalent a bond is the more negative its electronic energy density term, *H*(r), should be at the BCP. Here, we find moderately negative values for **3** and **5**, with slightly more negative values for **4M**, but substantially negative values for the nitrides. Lastly, the bond ellipticity, *ε*(r), is definitive for determining whether bonds are single, double, or triple in nature, being zero or close to zero for single or triple bonds, which present symmetrical distributions of electron density when viewed down the inter-nuclear axis, or up to ~0.5 for double bonds which are asymmetric. The **4M** series presents *ε*(r) values close to zero that are indicative of triple bonds but the deviation from zero reflects the fact they are not pure triple bonds being distorted by the fact the imido units in those complexes are bridging. Interestingly, the nitrides **11** and **12** deviate from zero, most probably reflecting the fact that whilst of triple bond parentage they are quite polarised and so not of pure triple bond character like the higher valent uranium complexes **13** and **14** which are clearly strong triple bonds.

## Discussion

We have assembled evidence that reduction of a thorium-azide generates a transient thorium-nitride with a Th≡N triple bond, however this linkage is highly reactive and activates C–H bonds of aromatic solvents to give isolable parent thorium-imido complexes that are themselves capable of activating C–H bonds. When the thorium-azide is coordinated to a Lewis acidic borane its reduction chemistry is blocked. Attempts to trap the thorium-nitride intermediate by incorporating it into a thorium-N-heterocycle were unsuccessful, but instead due to steric overload the N-heterocycle is formed and liberated confirming nitride formation and atom-transfer. Calculations consistently suggest that the Th≡N triple bond is more reactive than uranium analogues, and the formation of parent imidos with concomitant expulsion of alkali metal alkyl complements existing uranium(IV/VI)-nitride reactivity but also contrasts by introducing an alternative nitride reactivity outcome. For all the computed Th(IV)/U(IV)/U(V)/U(VI)-nitrides the An-N σ-bonds are found to be higher in energy than the two quasi-degenerate An-N *π*-bonding contributions even though this has previously been recognised only with high oxidation state uranium-element multiple bonds with high bond orders and short U-E distances. Whilst this can be in part accounted for by anti-bonding interactions raising the energy of the σ-bond at short actinide-nitrogen distances, an alternative explanation that would account for our data is that these systems are manifesting the pushing-from-below phenomenon where the pseudo-core 6p-orbitals are engaged in repulsive interactions, principally with 5f-orbitals but also likely to some extent with 6d-orbitals. Whilst recognising that pushing-from-below involving 6p-orbitals is almost certainly not the only factor contributing to *σ* > *π* energy ordering the calculations suggest that the *σ*-components of our computed An≡N triple bonds are stabilised relative to the *π*-components when the 6p-orbitals are frozen. Pushing-from-below occurs with hard, charge-dense and polarising anions and O^2−^ and N^3−^ fit this description, but RN^2−^ is softer, though HN^2−^ may be a special cross-over case. Such arguments also account for the different energy ordering of σ and π bonds in uranyl (*σ* > *π*) vs bis(imido) complexes (*π* > *σ*). Taken together, now recognising that thorium as well as uranium might be subject to pushing-from-below suggests that there may be a *σ*/*π* ligand field and metal identity/oxidation state energy ordering phenomenon that is far more periodic and thus commonly applicable than currently is appreciated.

## Methods

### General

Experiments were carried out under a dry, oxygen-free dinitrogen atmosphere using Schlenk-line and glove-box techniques. All solvents and reagents were rigorously dried and deoxygenated before use. Compounds were characterised by single-crystal X-ray diffraction, multi-nuclear NMR spectroscopy, infra-red spectroscopy, and elemental analyses, and DFT, NBO, QTAIM, and reaction profile theoretical calculations. Further details are provided in the Supplementary Information.

### Preparation of [Th(Tren^TIPS^)(N_3_)] (2)

Cold THF (100 ml, –78 °C) was added to solid **1** (8.80 g, 10.00 mmol) and NaN_3_ (1.30 g, 20.00 mmol). The resulting mixture was stirred at –78 °C for 1 h and then left to warm to room temperature and stirred for an additional 24 h. All volatiles were subsequently removed in vacuo. The remaining solids were extracted into hot hexanes (100 ml) and filtered. All volatiles were removed in vacuo, and the residue was washed with pentane (2 × 10 ml) and dried in vacuo to yield **2** as an analytically pure colourless solid (4.81 g). An additional 2.30 g of colourless crystals were obtained from the combined washings upon cooling to –30 °C. Yield: 7.11 g, 80%. Anal. Calcd for C_33_H_75_N_7_Si_3_Th: C, 44.72; H, 8.53; N, 11.06%. Found: C, 44.50; H, 9.03; N, 10.87%. ^1^H NMR (400 MHz, C_6_D_6,_ 298 K): δ (ppm) 1.20–1.33 (m, 63 H, C*H*(C*H*_3_)_2_), 2.55 (t, 1 H, ^3^*J*_HH_ = 4.9 Hz, 6 H, C*H*_2_CH_2_), 3.52 (t, ^3^*J*_HH_ = 4.9 Hz, 6 H, CH_2_C*H*_2_). ^13^C{^1^H} NMR (101 MHz, C_6_D_6_, 298 K): δ (ppm) 13.12 (*C*H(CH_3_)_3_), 19.48 (CH(*C*H_3_)_3_), 46.69 (*C*H_2_CH_2_), 62.89 (CH_2_*C*H_2_). ^29^Si{^1^H} NMR (79 MHz, C_6_D_6_, 298 K): δ (ppm) 4.15 (*Si*(CH(CH_3_)_2_)_3_). FTIR *v*/cm^−1^: 2938 (br, m), 2861 (br, m), 2086 (vs, N_3_^−^), 1462 (m), 1362 (s), 1269 (w), 1133 (m), 1060 (w), 1009 (w), 987 (s), 921 (s), 880 (w), 819 (vs), 736 (s), 673 (s), 631 (w), 548 (m), 514 (m), 450 (w), 423 (w). The ^15^N-isotopologue [Th(Tren^TIPS^)(^15/14^N^14^N^14/15^N)] was prepared analogously using Na(^15^N^14^N_2_). The asymmetric ν_azide_ stretching frequency for the ^15^N-isotopologue was measured at 2082 and 2070 cm^–1^ by FTIR.

### Reduction of 2 with KC_8_ in Toluene

Toluene (30 ml) was added slowly to a pre-cooled (–78 °C) mixture of **2** (0.89 g, 1.00 mmol) and KC_8_ (0.41 g, 3.00 mmol). The resulting mixture was stirred at –78 °C for 1 h and then left to warm to room temperature with stirring for 24 h, during which time the formation of black graphite was observed. The suspension was filtered to remove graphite and unreacted KC_8_, affording a colourless solution. All volatiles were removed in vacuo, yielding **3** as an off-white solid, which was crystallised from toluene to yield **3** as a colourless crystalline solid. Yield: 0.42 g, 45%. All characterisation data matched those of an authentic sample^79^.

### Preparation of [{Th(µ-NHLi)(Tren^TIPS^)}_2_] (4Li)

Benzene (40 ml) was added slowly to a pre-cooled (–78 °C) mixture of **5** (0.86 g, 1.00 mmol) and ^t^BuLi (0.064 g, 1.00 mmol). The frozen mixture was left to thaw to room temperature and stirred for 2 h, during which time the formation of colourless precipitate was observed. Removal of all volatiles in vacuo yielded a white solid, which was washed with pentane (2 × 10 ml) and dried in vacuo to afford analytically pure **4Li**. Yield: 0.48 g, 56%. Colourless crystals suitable for an X-ray diffraction study were obtained by cooling a saturated solution in hot benzene (80 °C) to room temperature. Anal. Calcd for C_66_H_152_Li_2_N_10_Si_6_Th_2_: C, 45.76; H, 8.84; N, 8.08%. Found: C, 46.05; H, 9.12; N, 7.52%. FTIR *v*/cm^−1^: 3393 (br, w, NH), 2937 (s), 2860 (s), 1460 (m), 1380 (w), 1275 (w), 1252 (w), 1136 (w), 1056 (s), 1009 (w), 927 (vs), 880 (s), 733 (vs), 669 (s), 628 (m), 574 (m), 539 (w), 508 (w), 476 (w). Once isolated as a pure sample, this compound is poorly soluble in non-polar solvents, and rapidly decomposes in the polar solvents THF and pyridine, and is therefore not amenable to NMR spectroscopic analysis.

### Preparation of [{Th(µ-NHNa)(Tren^TIPS^)}_2_] (4Na)

Benzene (80 ml) was added slowly to a pre-cooled (–78 °C) mixture of **5** (0.86 g, 1.00 mmol) and NaCH_2_Ph (0.23 g, 2.00 mmol). The frozen mixture was left to thaw to room temperature and stirred for 24 h, forming an orange suspension. The mixture was heated to 80 °C and filtered, colourless crystals started to form upon cooling the filtrate, which was left to further crystallise at 10 °C for 15 h. After decanting the mother liquor, crystalline **4Na** was dried in vacuo. Yield: 0.34 g, 38%. Anal. Calcd for C_66_H_152_N_10_Na_2_Si_6_Th_2_: C, 44.92; H, 8.68; N, 7.94%. Found: C, 43.84; H, 8.81; N, 7.79%. FTIR *v*/cm^−1^: 3397 (br, w, NH), 2939 (s), 2861 (s), 1461 (m), 1380 (w), 1275 (w), 1252 (w), 1128 (w), 1058 (m), 1010 (w), 928 (vs), 880(s), 736 (vs), 669 (s), 626(m), 562 (m), 539 (w), 508 (w), 438 (w). Once isolated as a pure sample, this compound is poorly soluble in non-polar solvents, and rapidly decomposes in the polar solvents THF and pyridine, and is therefore not amenable to NMR spectroscopic analysis.

### Preparation of [{Th(Tren^TIPS^)(µ-NHK)}_2_] (4K)

Benzene (120 ml) was added slowly to a pre-cooled (–78 °C) mixture of **2** (1.77 g, 2.00 mmol) and KC_8_ (0.81 g, 6.00 mmol). The frozen mixture was left to thaw to room temperature and stirred for 24 h, during which time the formation of black graphite was observed. The mixture was then heated to 90 °C to dissolve the poorly soluble product, and filtered to remove insoluble graphite and unreacted KC_8_. Colourless crystals started to form upon cooling the clear filtrate, which was left to further crystallise at 10 °C for 15 h. After decanting the mother liquor, crystalline **4K** was dried in vacuo. Yield: 0.90 g, 50%. Anal. Calcd for C_66_H_152_K_2_N_10_Si_6_Th_2_: C, 44.12; H, 8.53; N, 7.80%. Found: C, 44.32; H, 8.89; N, 7.48%. FTIR *v*/cm^−1^: 2936 (br, m), 2859 (s), 1458(m), 1378 (w), 1338 (w), 1275 (w), 1253 (w), 1137 (w), 1060 (w), 1023 (s), 1010 (w), 988 (w), 928 (vs), 880 (s), 809 (w), 735 (vs), 668 (s), 626 (m), 575 (w, Th = NH), 563 (w), 539 (w), 507 (w), 463 (w). The N–H stretching resonance was not observed in the IR spectrum (see Rb analogue). Once isolated as a pure sample, this compound is poorly soluble in non-polar solvents, and rapidly decomposes in the polar solvents THF and pyridine, and is therefore not amenable to NMR spectroscopic analysis. The ^15^N-isotopologue [{Th(Tren^TIPS^)(µ-^15^N[H]K)}_2_] (50 atom % ^15^N) was prepared analogously from [Th(Tren^TIPS^)(^15/14^N^14^N^14/15^N)]. The Th = NH asymmetric stretching frequency for [{Th(Tren^TIPS^)(µ-N[H]K)}_2_] was experimentally measured at 575 cm^–1^ by FTIR, which corresponds exactly to the calculated value (575 cm^–1^). For the ^15^N-isotopologue (50 atom% ^15^N), the Th = ^14^NH stretch at 575 cm^–1^ stretch was reduced in intensity, whilst an additional stretch was observed at 565 cm^–1^, corresponding to the isotopically shifted Th = ^15^NH stretch.

### Preparation of [{Th(Tren^TIPS^)(µ-NHRb)}_2_] (4Rb)

Benzene (80 ml) was added slowly to a pre-cooled (–78 °C) mixture of **2** (0.89 g, 1.00 mmol) and KC_8_ (0.41 g, 3.00 mmol). The frozen mixture was left to thaw to room temperature and stirred for 48 h, during which time the formation of black graphite was observed. After filtration to remove graphite and unreacted RbC_8_, a yellow solution was obtained. All volatiles were removed in vacuo yielding a yellow solid, which was washed with pentane (2 × 10 ml) and dried in vacuo to afford analytically pure **4Rb** as a colourless solid. Yield: 0.28 g, 30%. Colourless crystals suitable for an X-ray diffraction study were obtained by slow evaporation of a saturated solution in benzene. Anal. Calcd for C_66_H_152_N_10_Rb_2_Si_6_Th_2_: C, 41.95; H, 8.11; N, 7.41%. Found: C, 42.17; H, 8.44; N, 7.53%. FTIR *v*/cm^−1^: 2937 (s), 2860 (s), 1460 (m), 1400 (w), 1380 (w), 1275 (w), 1251 (w), 1128 (m), 1063 (w), 1010 (w), 989 (w), 930 (s), 879 (s), 809 (w), 736 (vs), 667 (s), 628 (w), 582 (w), 563 (w), 539(w), 507 (w), 459 (w). The N–H stretching resonance was not observed in the IR spectrum. Once isolated as a pure sample, this compound is poorly soluble in non-polar solvents, and rapidly decomposes in the polar solvents THF and pyridine, and is therefore not amenable to NMR spectroscopic analysis.

### Preparation of [{Th(Tren^TIPS^)(µ-NHCs)}_2_] (4Cs)

Benzene (80 ml) was added slowly to a pre-cooled (–78 °C) mixture of **2** (0.89 g, 1.00 mmol) and KC_8_ (0.41 g, 3.00 mmol). The frozen mixture was left to thaw to room temperature and stirred for 48 h, during which time the formation of black graphite was observed. After filtration to remove graphite and unreacted CsC_8_, a yellow solution was obtained. All volatiles were removed *in vacuo* yielding a yellow solid, which was washed with pentane (2 × 10 ml) and dried *in vacuo* to afford analytically pure **4Cs** as a colourless solid. Yield: 0.20 g, 20%. Colourless crystals suitable for an X-ray diffraction study were obtained by slow evaporation of a saturated solution in benzene. Anal. Calcd for C_66_H_152_N_10_Cs_2_Si_6_Th_2_: C, 39.95; H, 7.72; N, 7.06%. Found: C, 39.87; H, 7.85; N, 6.75%. FTIR *v*/cm^−1^: 2935 (s), 2858 (s), 1458 (m), 1386 (w), 1339 (w), 1275 (w), 1253 (w), 1138 (w), 1063 (s), 1024 (w), 1012 (w), 931 (vs), 880 (s), 738 (vs), 669 (s), 628 (m), 580 (s), 539 (w), 508 (w), 439 (w). The N–H stretching resonance was not observed in the IR spectrum. Once isolated as a pure sample, this compound is poorly soluble in non-polar solvents, and rapidly decomposes in the polar solvents THF and pyirdine, and is therefore not amenable to NMR spectroscopic analysis.

### Preparation of [Th(Tren^TIPS^)(NH_2_)] (5)

A NH_3_ solution (50 ml, 20 mmol) in THF (0.4 M) was added into a pre-cooled (–78 °C) colourless suspension of [Th^cyclomet^(Tren^TIPS^)] (8.43 g, 10.00 mmol) in DME (50 ml), and then the mixture was allowed to warm to the room temperature and stirred for 48 h. All volatiles were removed in vacuo yielding a colourless solid, which was extracted with hexanes (80 ml) and filtered. Removal of the volatiles in vacuo afforded a colourless solid, which was then washed with cold pentane (–78 °C, 2 × 10 ml) and dried. Yield: 6.62 g, 77%. Colourless crystals of **5** suitable for an X-ray diffraction study were obtained by slow evaporation of a saturated solution in pentane in the glove box. Anal. Calcd for C_33_H_77_N_5_Si_3_Th: C, 46.07; H, 9.02; N, 8.14%. Found: C, 45.64; H, 9.25; N, 7.92%. ^1^H NMR (400 MHz, C_6_D_6,_ 298 K): δ (ppm) 1.26 (d, ^3^*J*_HH_ = 7.1 Hz, 54 H, CH(C*H*_3_)_2_), 1.33–1.43 (m, 9 H, C*H*(CH_3_)_2_), 2.58 (t, ^3^*J*_HH_ = 4.7 Hz, 6 H, C*H*_2_CH_2_), 3.21 (br, s, 2 H, N*H*_2_), 3.56 (t, ^3^*J*_HH_ = 4.7 Hz, 6 H, CH_2_C*H*_2_). ^13^C{^1^H} NMR (101 MHz, C_6_D_6_, 298 K): δ (ppm) 13.00 (*C*H(CH_3_)_3_), 19.43 (CH(*C*H_3_)_3_), 45.63 (*C*H_2_CH_2_), 63.06 (CH_2_*C*H_2_). ^29^Si{^1^H} NMR (79 MHz, C_6_D_6_, 298 K): δ (ppm) 3.44. FTIR *v*/cm^−1^: 2939 (br, s), 2862 (m), 1512 (w), 1461 (m), 1379 (w), 1341 (w), 1273 (w), 1136 (w), 1052 (s), 1010 (m), 925 (s), 880 (s), 817 (w), 737 (vs), 670 (s), 625 (m), 565 (w), 465 (w), 443 (w). The N–H stretching resonance was not observed in the IR spectrum.

### Preparation of [Th(Tren^TIPS^)(N_3_(B(C_6_F_5_)_3_))] (6)

Hexanes (30 ml) were added slowly to a pre-cooled (–78 °C) mixture of **2** (0.89 g, 1.00 mmol) and B(C_6_F_5_)_3_ (0.52 g, 1.00 mmol). The resulting mixture was left to warm to room temperature and stirred for 24 h, during which time a white precipitate formed. The precipitate was isolated by filtration and washed with pentane (2 × 10 ml) and dried in vacuo to yield **6** as an analytically pure colourless solid. Yield: 0.87 g, 62%. Colourless crystals suitable for an X-ray diffraction study were obtained by cooling a concentrated toluene solution at –30 °C for 2 days. Anal. Calcd C_51_H_75_BF_15_N_7_Si_3_Th·(C_7_H_8_): C, 46.74; H, 5.61; N, 6.58%. Found: C, 46.34; H, 5.66; N, 6.61%. ^1^H NMR (400 MHz, C_6_D_6,_ 298 K): δ (ppm) 0.99–1.07 (m, 63 H, C*H*(C*H*_3_)_2_), 2.48 (t, ^3^*J*_HH_ = 4.9 Hz, 6 H, C*H*_2_CH_2_), 3.41 (t, ^3^*J*_HH_ = 4.9 Hz, 6 H, CH_2_C*H*_2_). ^13^C{^1^H} NMR (101 MHz, C_6_D_6_, 298 K): δ (ppm) 12.48 (*C*H(CH_3_)_3_), 18.93 (CH(*C*H_3_)_3_), 47.13 (*C*H_2_CH_2_), 62.07 (CH_2_*C*H_2_), 135.95 (Ar-*C*), 138.74 (Ar-*C*), 141.50 (Ar-*C*), 147.74 (Ar-*C*), 149.54 (Ar-*C*). ^29^Si{^1^H} NMR (79 MHz, C_6_D_6_, 298 K): δ (ppm) 4.86 (*Si*(CH(CH_3_)_2_)_3_). ^11^B{^1^H} NMR (128 MHz, C_6_D_6_, 298 K): δ (ppm) –5.79 (br, *B*(C_6_F_5_)_3_. ^19^F{^1^H} NMR (376 MHz, C_6_D_6_, 298 K): δ (ppm) –134.24 (dd, ^3^*J*_FF_ = 23.8, ^4^*J*_FF_ = 8.7 Hz, 6 F, *o*-F), –157.49 (t, ^3^*J*_FF_ = 20.6 Hz, 3 F, *p*-F), –163.93 (td, ^3^*J*_FF_ = 23.8, ^4^*J*_FF_ = 8.7 Hz, 6 F, *m*-F). FTIR *v*/cm^−1^: 2946 (br, m), 2863 (br, m), 2171 (vs, N_3_^−^), 1643 (m), 1515 (s), 1458 (vs), 1382 (w), 1284 (m), 1104 (s), 1050 (w), 972 (w), 919 (s), 894 (s), 880 (s), 787 (w), 772 (w), 736 (vs), 673 (vs), 631 (w), 573 (w), 55(w).

### Reduction of 2 with KC_8_ in the Presence of (ClSiMe_2_CH_2_)_2_

C_6_D_6_ (15 ml) was added slowly to a pre-cooled (–78 °C) mixture of **2** (0.27 g, 0.30 mmol), KC_8_ (0.12 g, 0.90 mmol), and (ClSiMe_2_CH_2_)_2_ (0.07 g, 0.30 mmol). The frozen mixture was left to thaw to room temperature and stirred for 24 h, during which time the formation of black graphite was observed. ^1^H NMR analysis of the filtered solution revealed the formation of **1**, along with a small amount of HN(Si(Me)_2_CH_2_)_2_, identified by comparison to an authentic sample (see Supplementary Figs. 15 and 16). Due to the small quantity of HN(Si(Me)_2_CH_2_)_2_ produced, it was not possible to isolate this compound from the mixture.

### Preparation of [{KN(SiMe_2_CH_2_)_2_}_2_] (8)

A solution of HN(SiMe_2_CH_2_)_2_ (0.64 g, 4.00 mmol) in THF (5 ml) was added slowly to a pre-cooled (–78 °C) suspension of PhCH_2_K (0.52 g, 4.00 mmol) in THF (10 ml). The resulting mixture was stirred at –78 °C for 1 h and then left to warm to room temperature and stirred for an additional 24 h. All volatiles were subsequently removed in vacuo. The remaining solids were extracted into toluene (15 ml) and filtered through celite® to afford a pale yellow solution. Toluene was then removed in vacuo, and the resulting off-white solid was washed with pentane (2 × 5 ml) and dried in vacuo to yield KN(Si(Me)_2_CH_2_)_2_ as a colourless solid. Yield: 0.40 g, 50%. Colourless crystals suitable for an X-ray diffraction study were obtained by cooling a saturated toluene solution at –30 °C. Anal. Calcd for C_6_H_16_KNSi_2_: C, 36.49; H, 8.17; N, 7.09%. Found: C, 36.67; H, 8.32; N, 6.89%. ^1^H NMR (400 MHz, C_6_D_6,_ 298 K): δ (ppm) 0.00 (s, 12 H, Si(C*H*_3_)_2_), 1.04 (s, 4 H, C*H*_2_C*H*_2_). ^13^C{^1^H} NMR (101 MHz, C_6_D_6_, 298 K): δ (ppm) 5.14 (Si(*C*H_3_)_2_), 12.75 (*C*H_2_*C*H_2_). ^29^Si{^1^H} NMR (79 MHz, C_6_D_6_, 298 K): δ (ppm) 1.01(*Si*(CH_3_)_2_). FTIR *v*/cm^−1^: 2933 (w), 2875 (br, w), 1577 (w), 1304 (w), 1232 (vs), 1184 (w), 1124 (w), 980 (vs), 877 (s), 832 (w), 807 (w), 758 (s), 650 (s), 519 (w), 422 (w).

### Preparation of [Th(Tren^TIPS^)(OMe)] (9)

DME (30 ml) was added slowly to a pre-cooled (–78 °C) mixture of [Th(Tren^TIPS^)(DME)][BPh_4_] (1.25 g, 1.0 mmol) and KN(SiMe_2_CH_2_)_2_ (0.20 g, 1.0 mmol). The resulting mixture was stirred at –78 °C for 1 h and then left to warm to room temperature and stirred for 24 h, forming a colourless solution. All volatiles were removed in vacuo, and the residue was extracted with toluene (30 ml) and filtered, yielding a colourless solution. Then all volatiles were removed in vacuo again, and the oily residue was extracted with pentane (10 ml) and subsequently concentrated to 4 ml. Colourless crystals of **9** suitable for an X-ray diffraction study were obtained by cooling the concentrated pentane solution at –30 °C. Yield: 0.22 g, 24%. Anal. Calcd for C_34_H_78_N_4_OSi_3_Th·0.5(C_5_H_12_): C, 48.05; H, 9.39; N, 6.14%. Found: C, 48.28; H, 9.39; N, 6.18%. ^1^H NMR (400 MHz, C_6_D_6,_ 298 K): δ (ppm) 1.26–1.27 (m, 63 H, C*H*(C*H*_3_)_2_), 2.61 (t, 1 H, ^3^*J*_HH_ = 4.9 Hz, 6 H, C*H*_2_CH_2_), 3.56 (t, ^3^*J*_HH_ = 4.9 Hz, 6 H, CH_2_C*H*_2_), 3.72 (s, 3 H, OC*H*_3_). ^13^C{^1^H} NMR (101 MHz, C_6_D_6_, 298 K): δ (ppm) 12.73 (*C*H(CH_3_)_3_), 19.21 (CH(*C*H_3_)_3_), 45.58 (*C*H_2_CH_2_), 61.23 (O*C*H_3_), 62.58 (CH_2_*C*H_2_). ^29^Si{^1^H} NMR (79 MHz, C_6_D_6_, 298 K): δ (ppm) 3.10 (*Si*(CH(CH_3_)_2_)_3_). FTIR *v*/cm^−1^: 2938 (br, m), 2860 (br, m), 1462 (m), 1271 (w), 1104 (s), 1057 (m), 1008 (w), 925 (s), 880 (s), 739 (vs), 671 (s), 628 (m), 544 (w), 511 (w), 445 (w).

### Preparation of [Th(Tren^DMBS^)(N(Si(Me)_2_CH_2_)_2_)] (10)

Toluene (15 ml) was added slowly to a pre-cooled (–78 °C) mixture of [Th(Tren^DMBS^)(I)] (0.42 g, 0.50 mmol) and KN(SiMe_2_CH_2_)_2_ (0.10 g, 0.50 mmol). The resulting mixture was stirred at –78 °C for 1 h and then left to warm to room temperature and stirred for 24 h, forming a suspension, which was filtered through celite® to remove the KI salt. All volatiles were removed in vacuo, and the residue was extracted with pentane (10 ml) and subsequently concentrated to 2 ml. Colourless crystals of **10** suitable for an X-ray diffraction study were obtained by cooling the concentrated pentane solution at –30 °C. Yield: 0.33 g, 76%. Anal. Calcd for C_30_H_73_N_5_Si_5_Th: C, 41.11; H, 8.40; N, 7.99%. Found: C, 41.65; H, 8.66; N, 7.94%. ^1^H NMR (400 MHz, C_6_D_6,_ 298 K): δ (ppm) 0.37 (s, 18 H, Si((C*H*_3_)_2_(^*t*^Bu)), 0.52 (s, 12 H, Si(C*H*_3_)_3_), 0.89 (s, 4 H, C*H*_2_C*H*_2_), 1.03 (s, 27 H, Si(Me_2_(C(C*H*_3_)_3_))), 2.57 (t, ^3^*J*_HH_ = 5.1 Hz, 6 H, C*H*_2_CH_2_), 3.34 (t, ^3^*J*_HH_ = 5.1 Hz, 6 H, CH_2_C*H*_2_). ^13^C{^1^H} NMR (101 MHz, C_6_D_6_, 298 K): δ (ppm) –3.47 (Si((*C*H_3_)_2_(^*t*^Bu)), 2.81 (Si((*C*H_3_)_2_), 11.09 (*C*H_2_*C*H_2_), 20.59 (SiMe_2_(*C*(CH_3_)_3_))), 27.83 (Si(Me_2_(C(*C*H_3_)_3_)), 46.51 (*C*H_2_CH_2_), 66.96 (CH_2_*C*H_2_). ^29^Si{^1^H} NMR (79 MHz, C_6_D_6_, 283 K): δ (ppm) 4.12 (*Si*Me_2_), –2.31 (*Si*Me_2_^*t*^Bu). FTIR *v*/cm^−1^: 2951 (m), 2926 (m), 2882 (m), 2851 (m), 1467 (m), 1345 (w), 1247 (s), 1066 (s), 1025 (w), 924 (s), 875 (s), 801 (s), 771 (vs), 743 (vs), 712 (s), 654 (m), 566 (m), 548 (m), 443 (w).

## Supplementary information

Supplementary Information

## Data Availability

The X-ray crystallographic coordinates for the structures reported in this Article have been deposited at the Cambridge Crystallographic Data Centre (CCDC) under deposition nos. 1869060-1869070. These data can be obtained free of charge from The Cambridge Crystallographic Data Centre (www.ccdc.cam.ac.uk/data_requst/cif). All other data can be obtained from the authors on request.
