# Supplementary material for: Thorium-nitrogen multiple bonds provide evidence for pushing-from-below for early actinides
Source: Nat Commun. 2019 Sep 13;10:4203. doi: 10.1038/s41467-019-12206-5 (PMC6744569; doi:10.1038/s41467-019-12206-5)
Supplement: Supplementary file 1 — Supplementary Information [file 41467_2019_12206_MOESM1_ESM.pdf]

Thorium-Nitrogen Multiple Bonds Provide Evidence for Pushing-from-Below for Early Actinides

Du et al.

Supplementary Figures

Characterisation Data

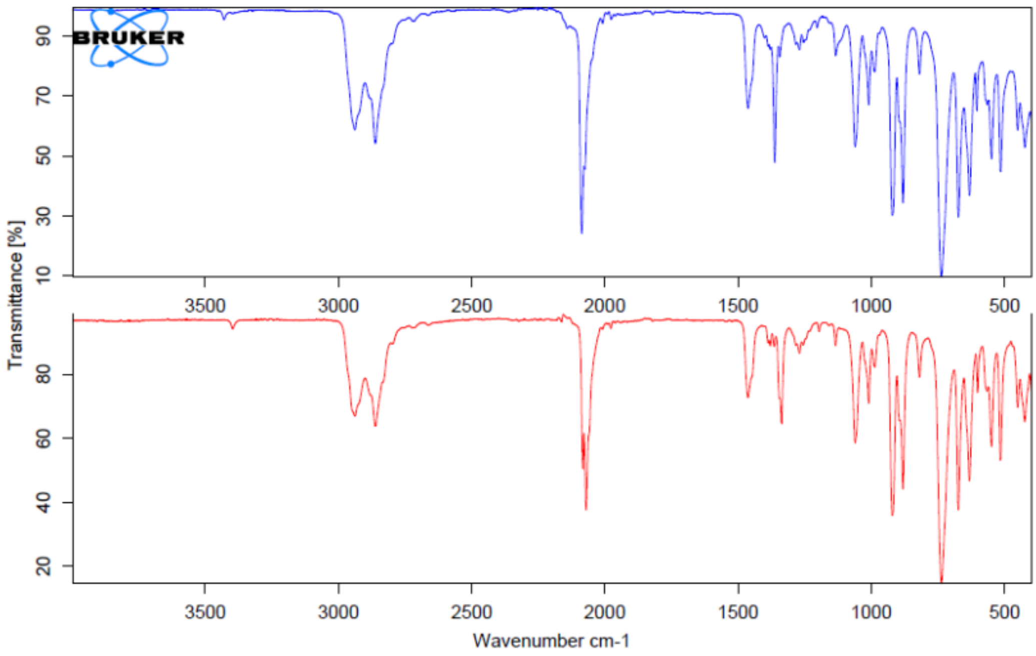

Supplementary Figure 1. IR spectra of **2** (Blue) and the corresponding ^{15}N-isotopologue (Red).

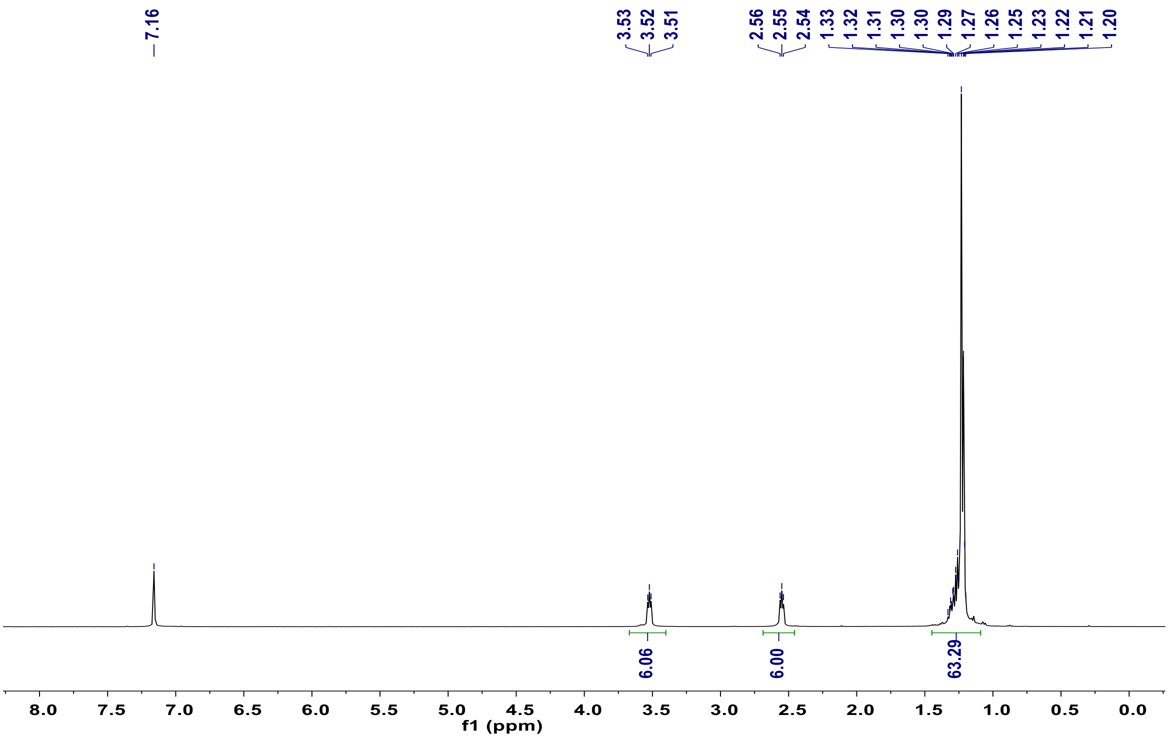

Supplementary Figure 2. ^{1}H NMR (400 MHz, C_6D_6, 298 K) of **2**.

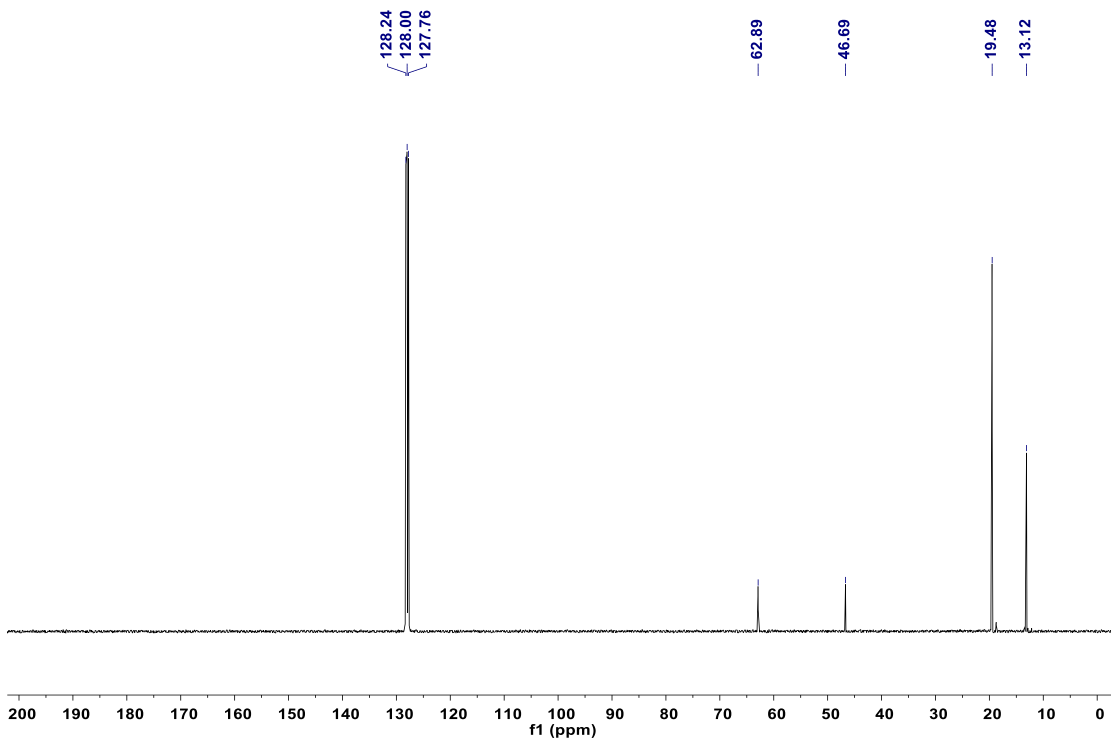

Supplementary Figure 3. $^{13}C\{^{1}H\}$ NMR (101 MHz, C_6D_6, 298 K) of **2**.

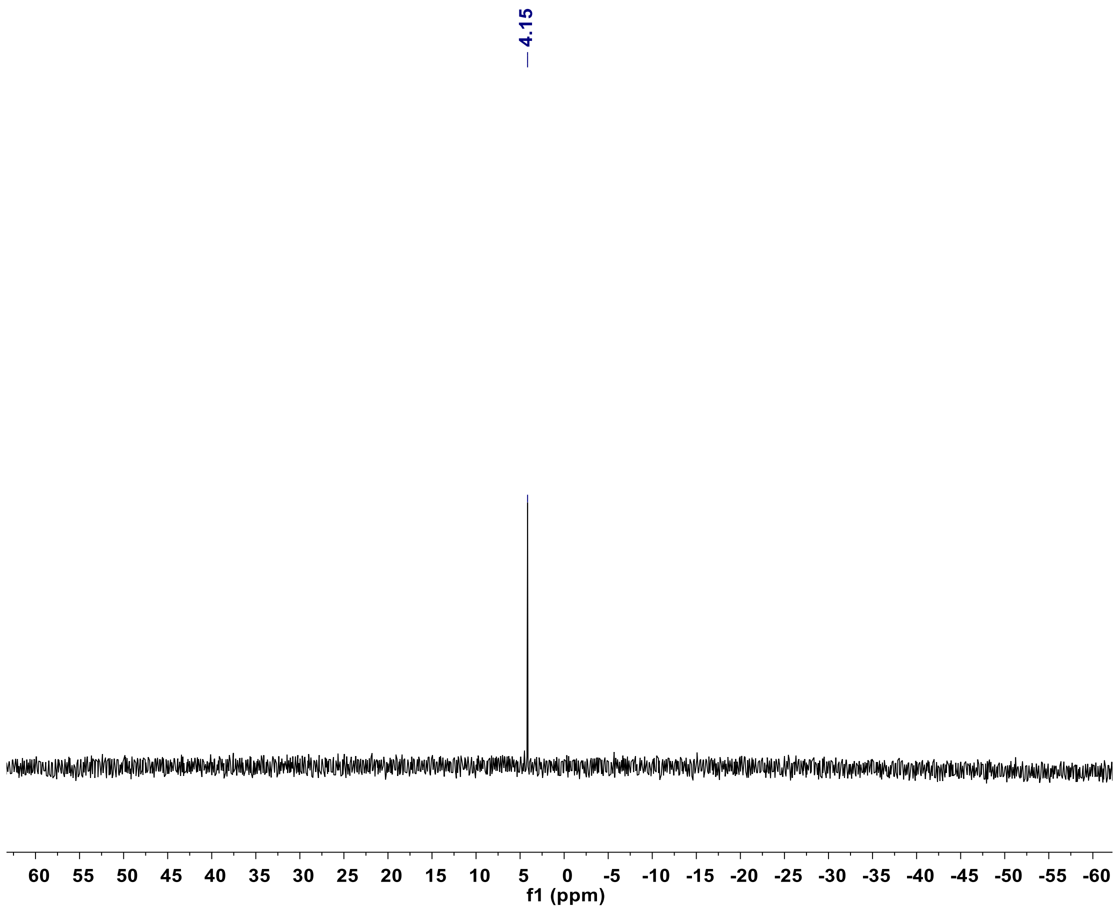

Supplementary Figure 4. $^{29}Si\{^{1}H\}$ NMR (79 MHz, C_6D_6, 298 K) of **2**.

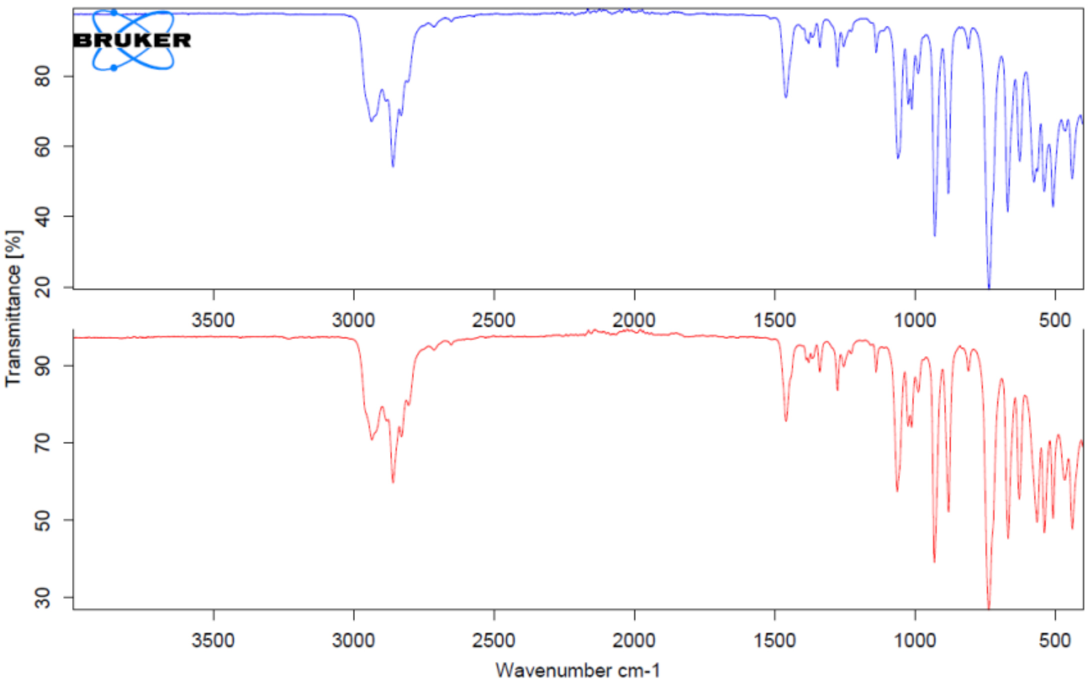

Supplementary Figure 5. IR spectra of **4K** (Blue) and the corresponding ^{15}N-isotopologue (Red).

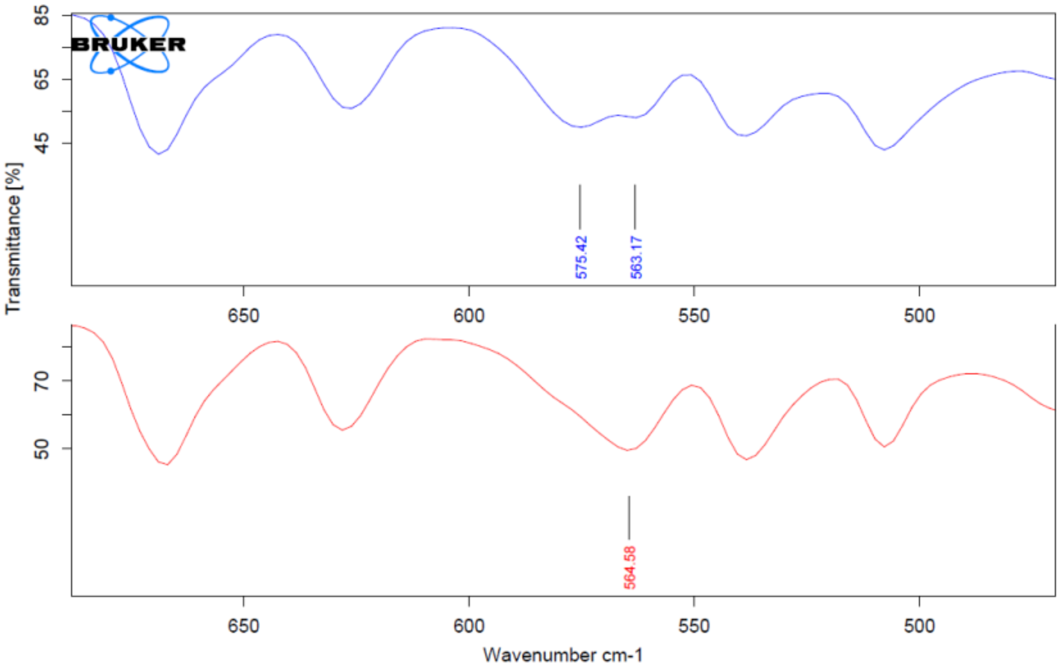

Supplementary Figure 6. IR spectra of **4K** (Blue) and the corresponding ^{15}N-isotopologue (Red) between 500 to 650 cm^{-1}.

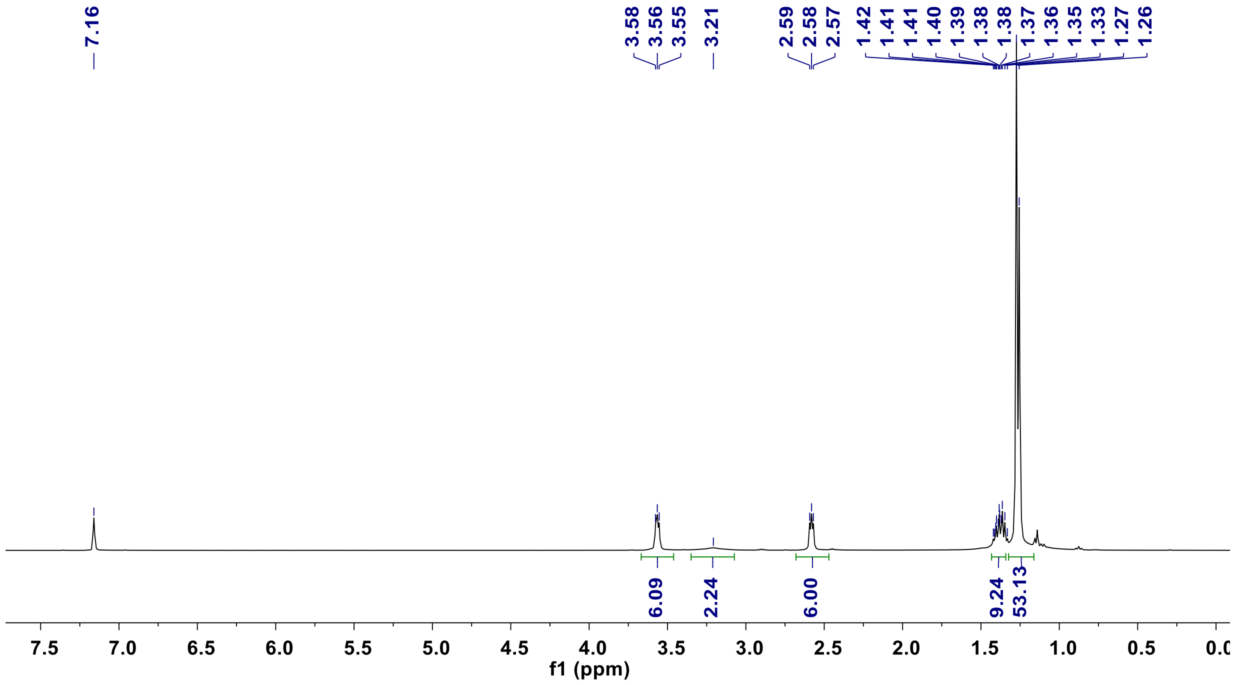

Supplementary Figure 7. ^{1}H NMR (400 MHz, C_6D_6, 298 K) of **5**.

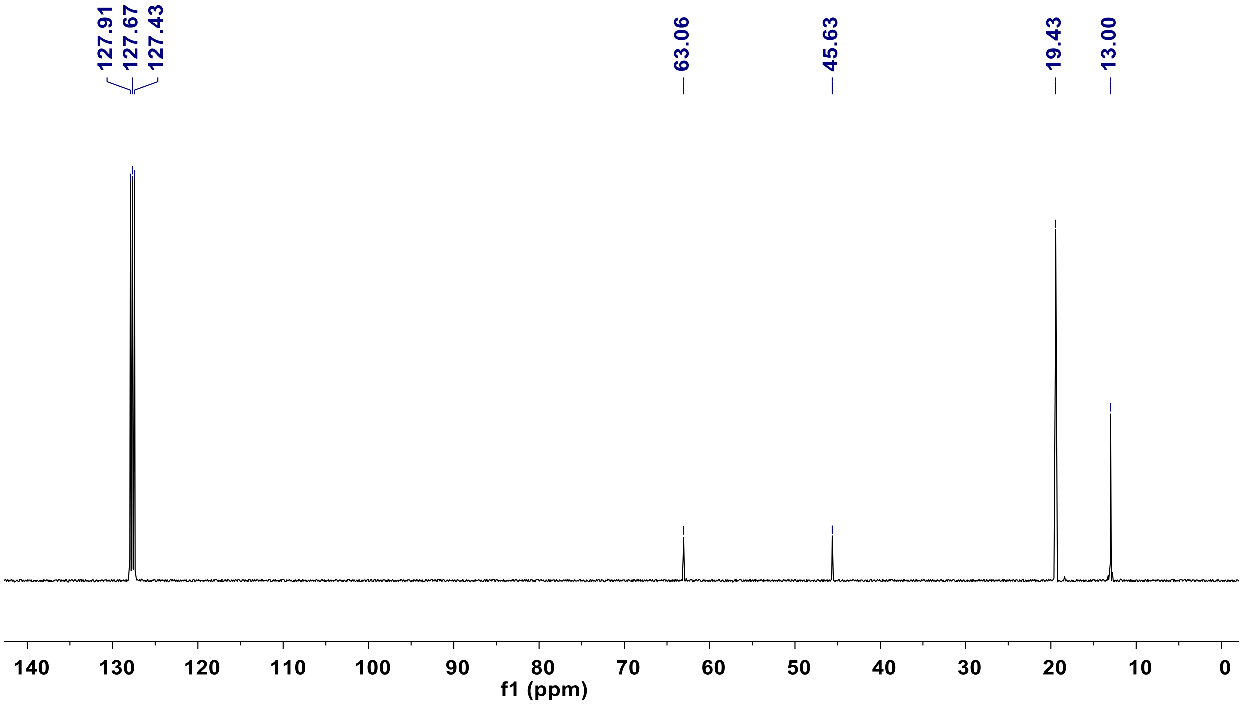

Supplementary Figure 8. ^{13}C{^{1}H} NMR (101 MHz, C_6D_6, 298 K) of **5**.

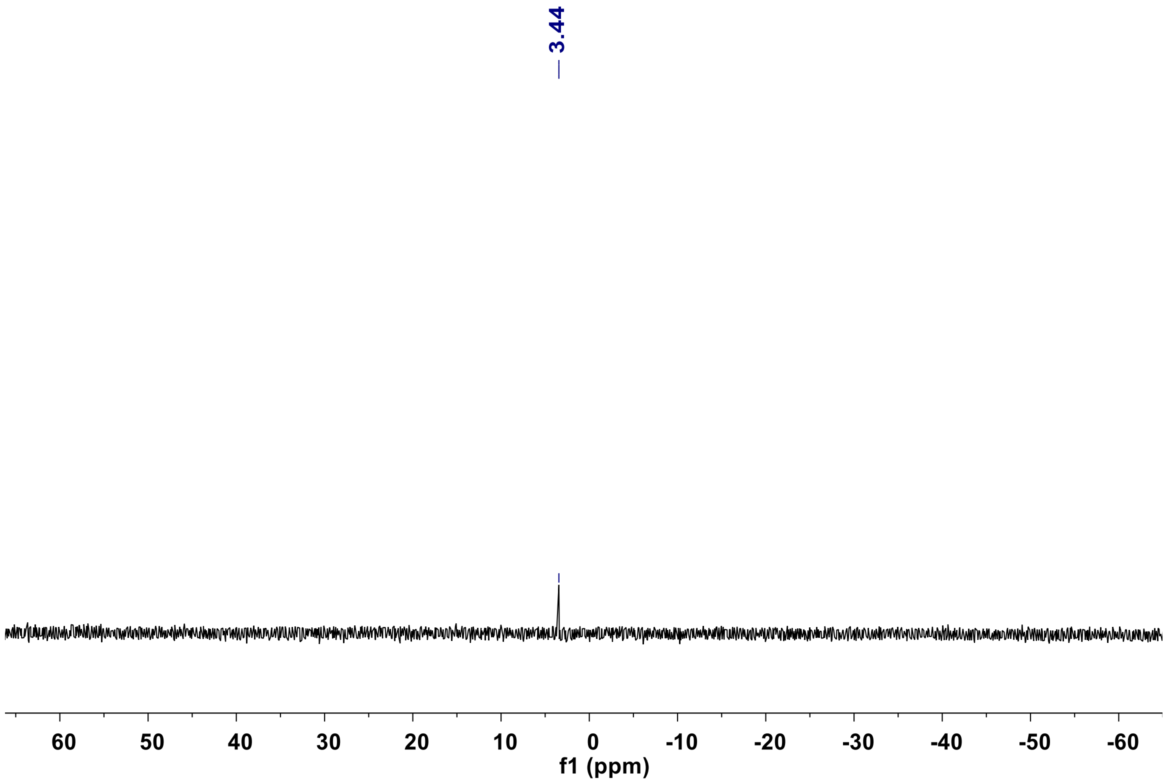

Supplementary Figure 9. $^{29}Si\{^{1}H\}$ NMR (79 MHz, C_6D_6, 298 K) of **5**.

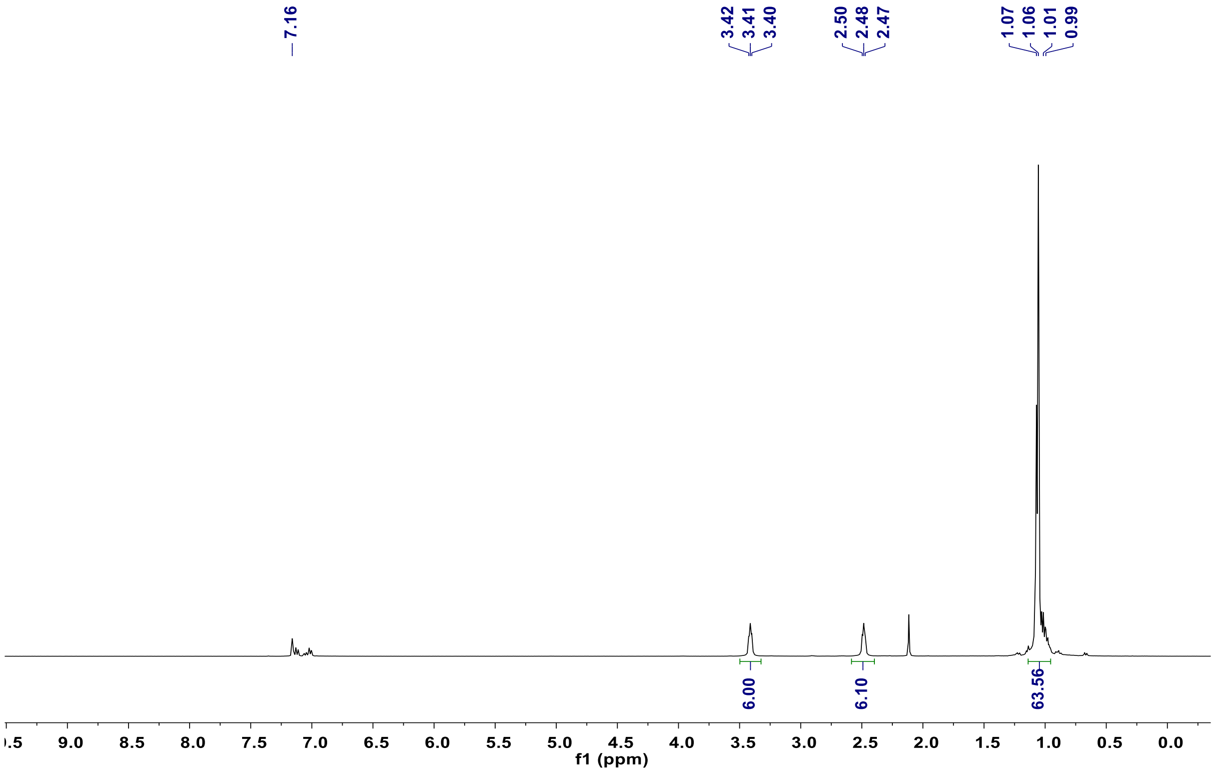

Supplementary Figure 10. ^{1}H NMR (400 MHz, C_6D_6, 298 K) of **6**.

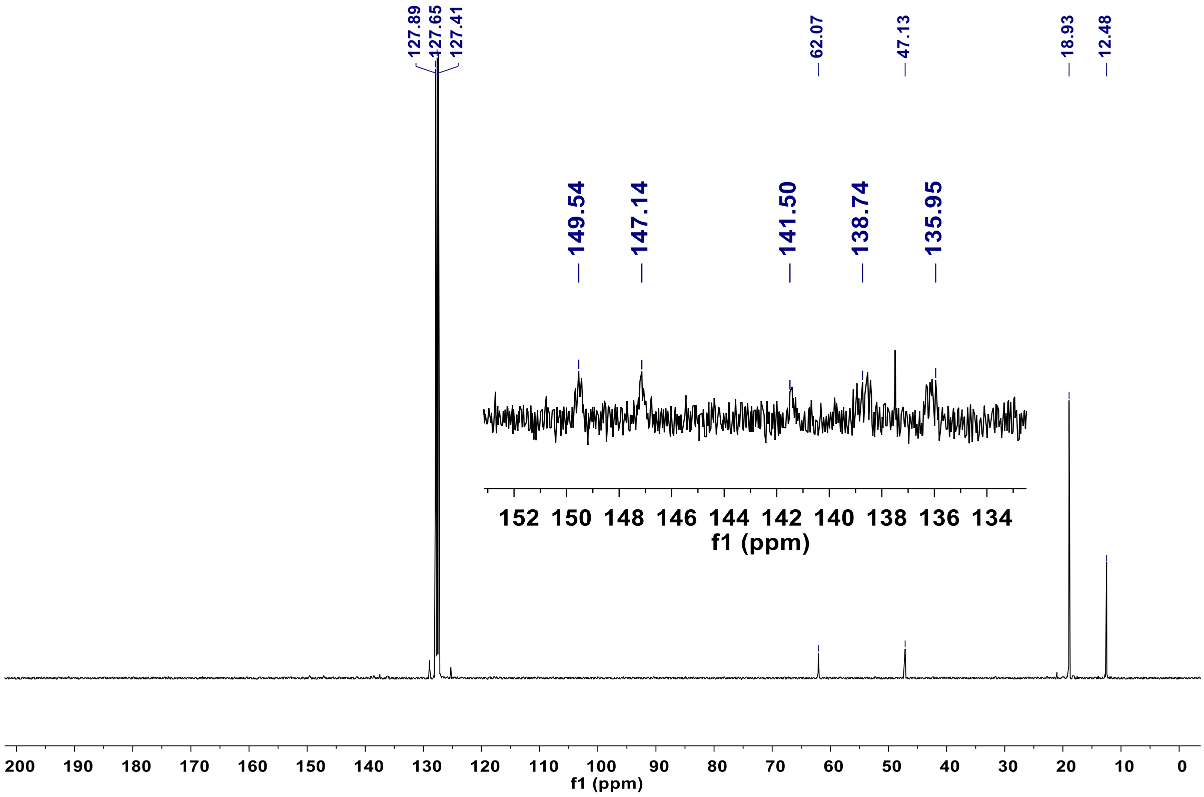

Supplementary Figure 11. $^{13}C\{^{1}H\}$ NMR (101 MHz, C_6D_6, 298 K) of **6**.

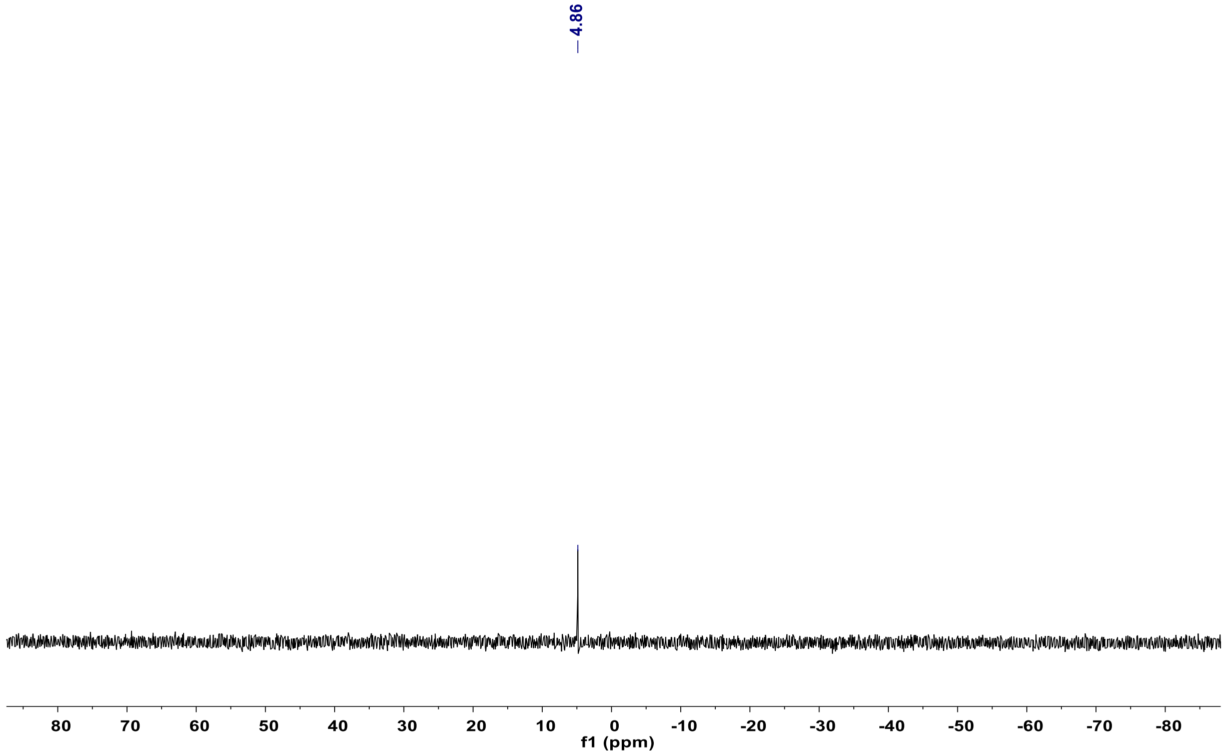

Supplementary Figure 12. $^{29}Si\{^{1}H\}$ NMR (79 MHz, C_6D_6, 298 K) of **6**.

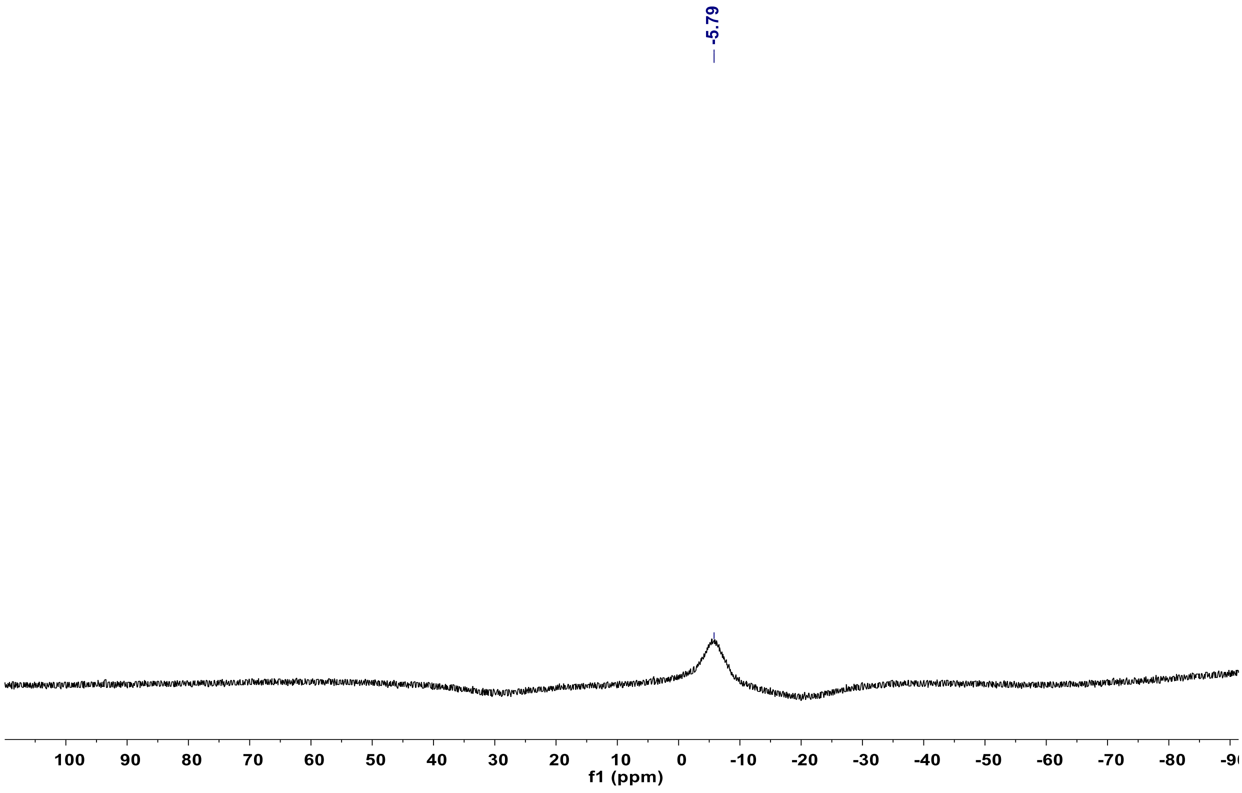

Supplementary Figure 13. $^{11}B\{^{1}H\}$ NMR (128 MHz, C_6D_6, 298 K) of **6**.

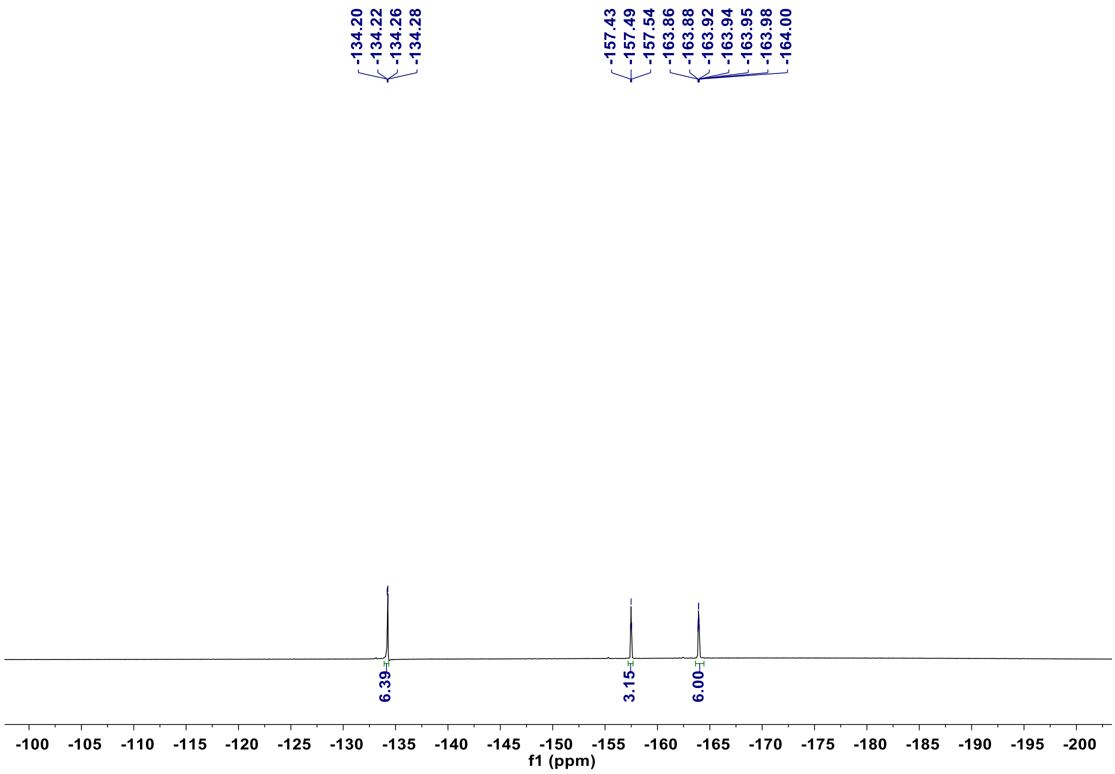

Supplementary Figure 14. $^{19}F\{^{1}H\}$ NMR (376 MHz, C_6D_6, 298 K) of **6**.

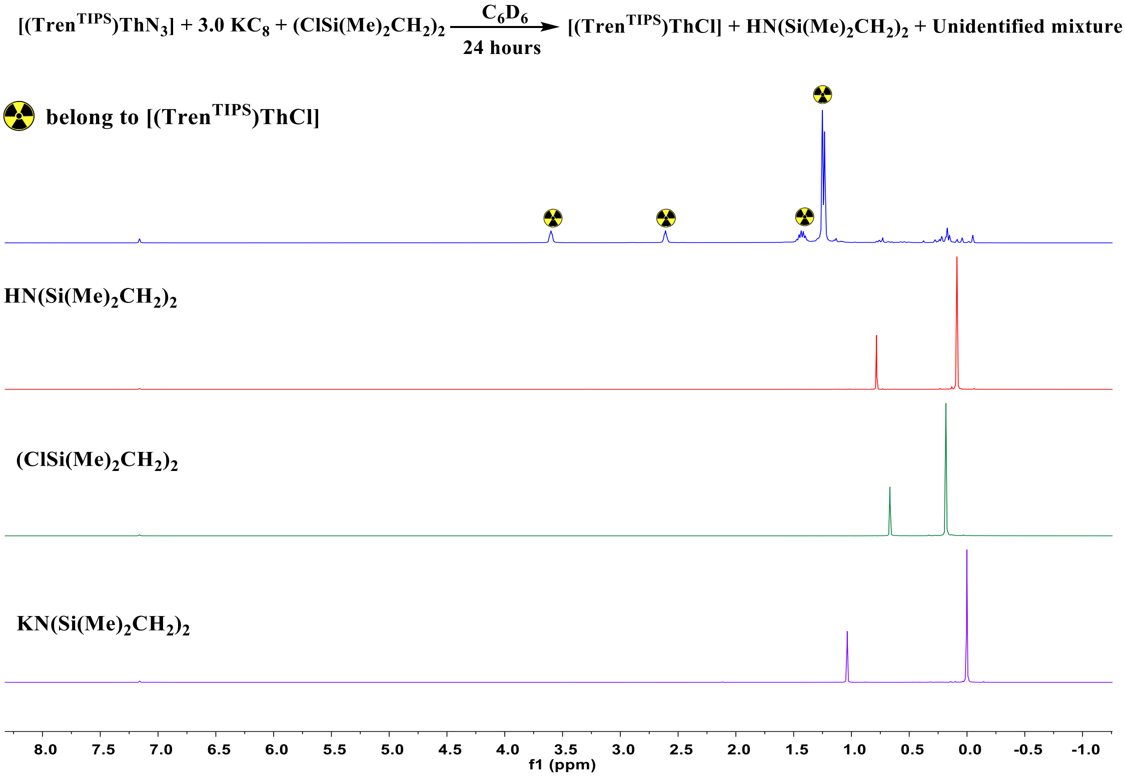

Supplementary Figure 15. Comparison of ^{1}H NMR spectra of **8**, the chlorosilane, the free *N*-heterocyclic amine, and the mixture from **2** reduced with KC_8 in the presence of the chlorosilane.

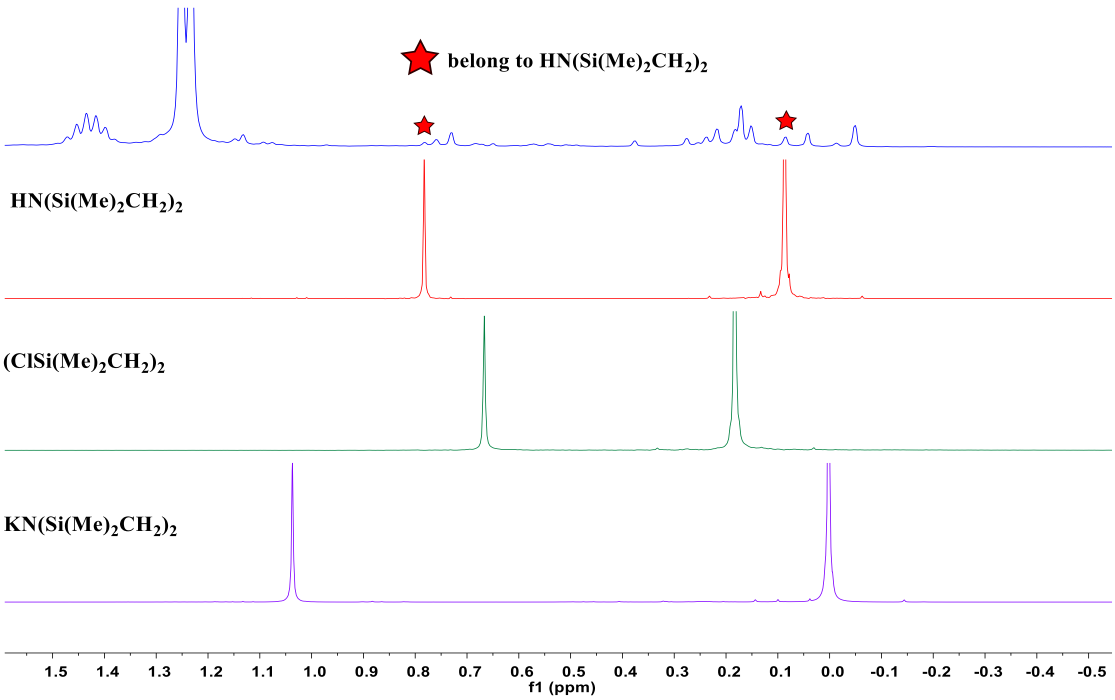

Supplementary Figure 16. Zoom-in of the spectra in Figure S18.

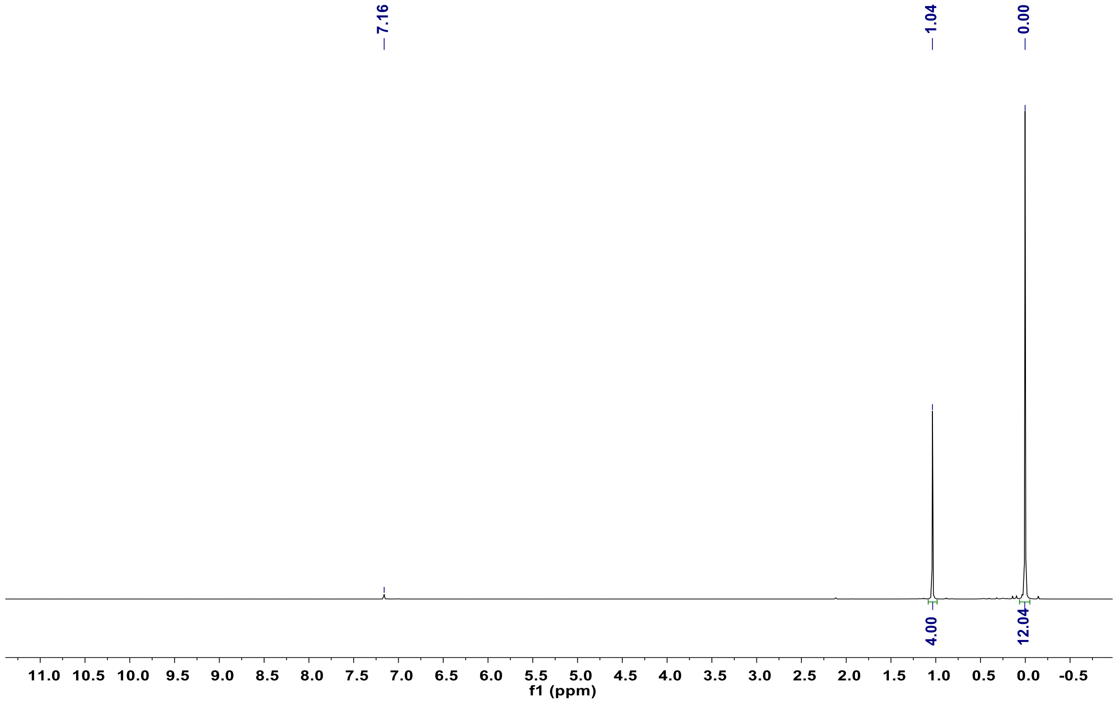

Supplementary Figure 17. ^{1}H NMR (400 MHz, C_6D_6, 298 K) of **8**.

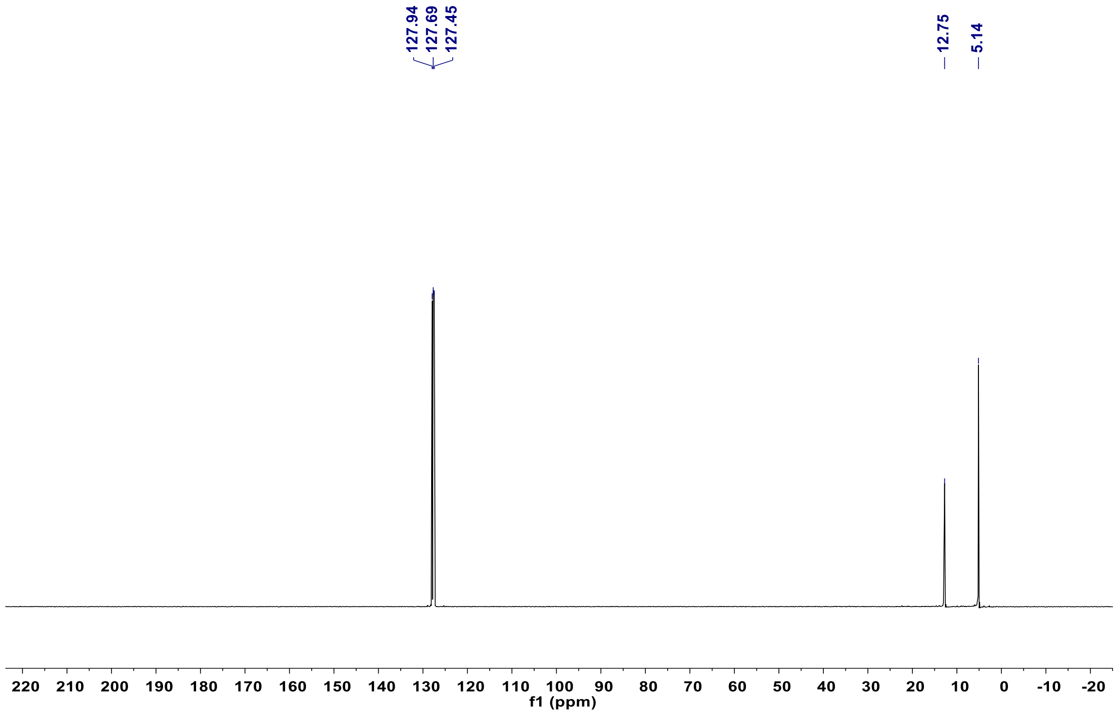

Supplementary Figure 18. ^{13}C{^{1}H} NMR (101 MHz, C_6D_6, 298 K) of **8**.

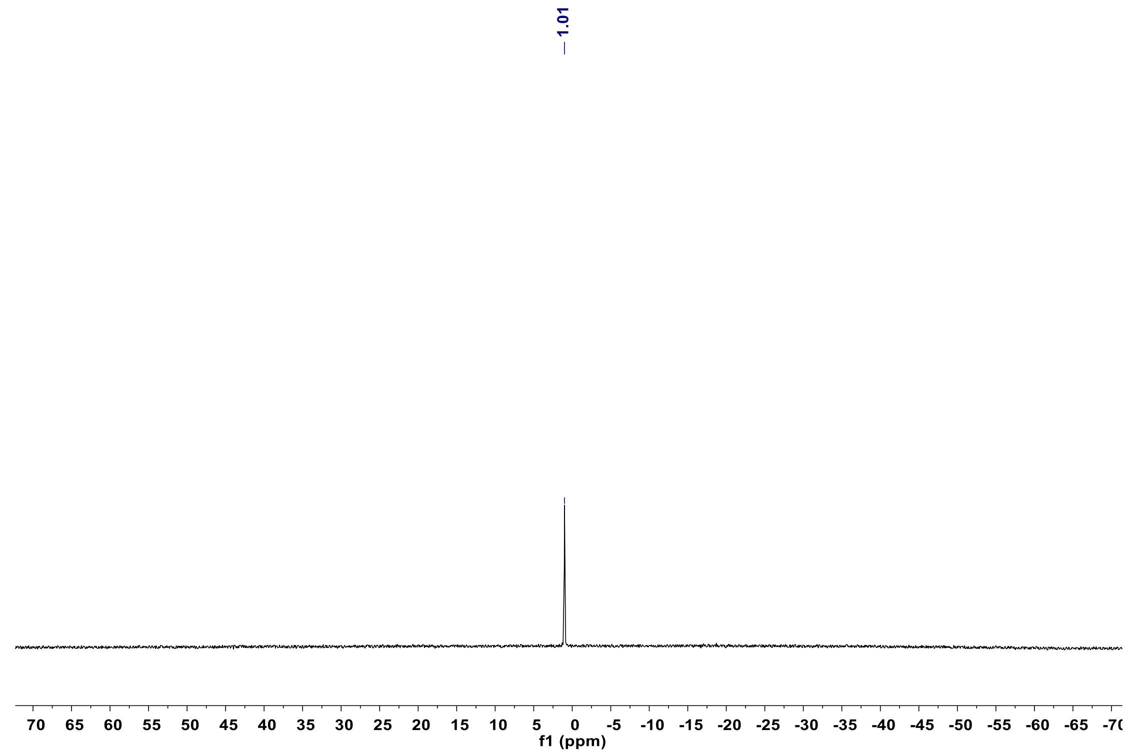

Supplementary Figure 19. $^{29}Si\{^{1}H\}$ NMR (79 MHz, C_6D_6, 298 K) of **8**.

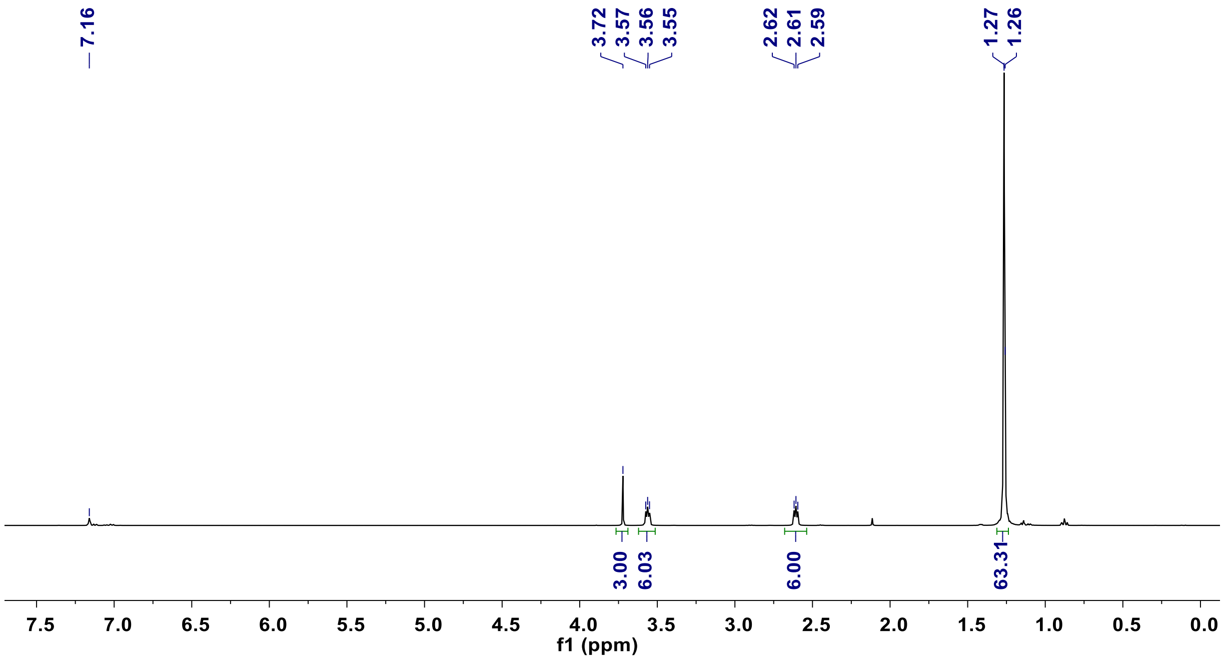

Supplementary Figure 20. ^{1}H NMR (400 MHz, C_6D_6, 298 K) of **9.**

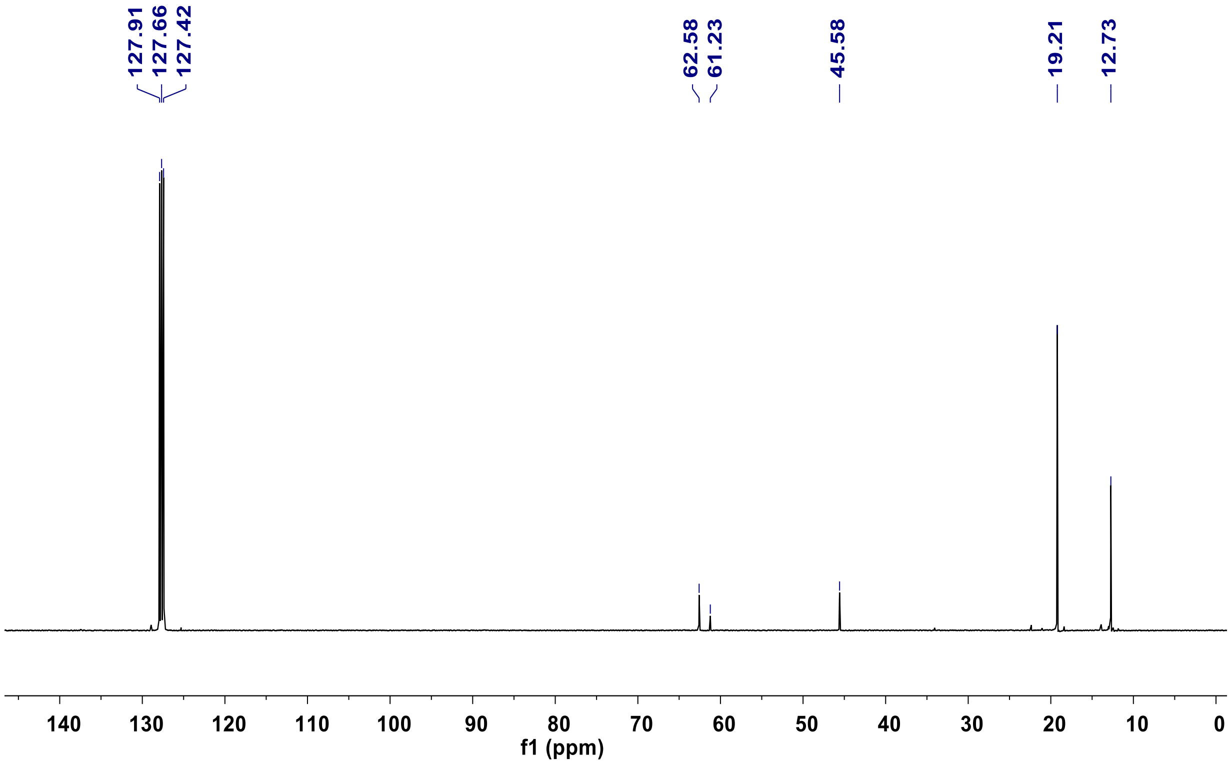

Supplementary Figure 21. $^{13}C\{^{1}H\}$ NMR (101 MHz, C_6D_6, 298 K) of **9.**

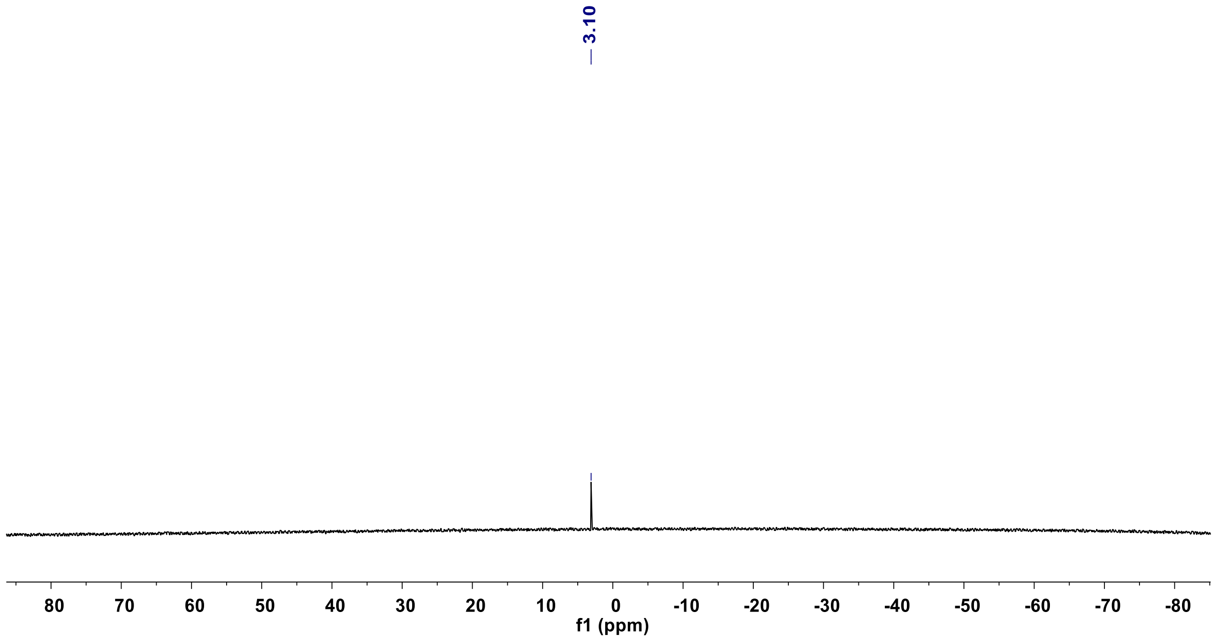

Supplementary Figure 22. $^{29}Si\{^{1}H\}$ NMR (79 MHz, C_6D_6, 298 K) of **9.**

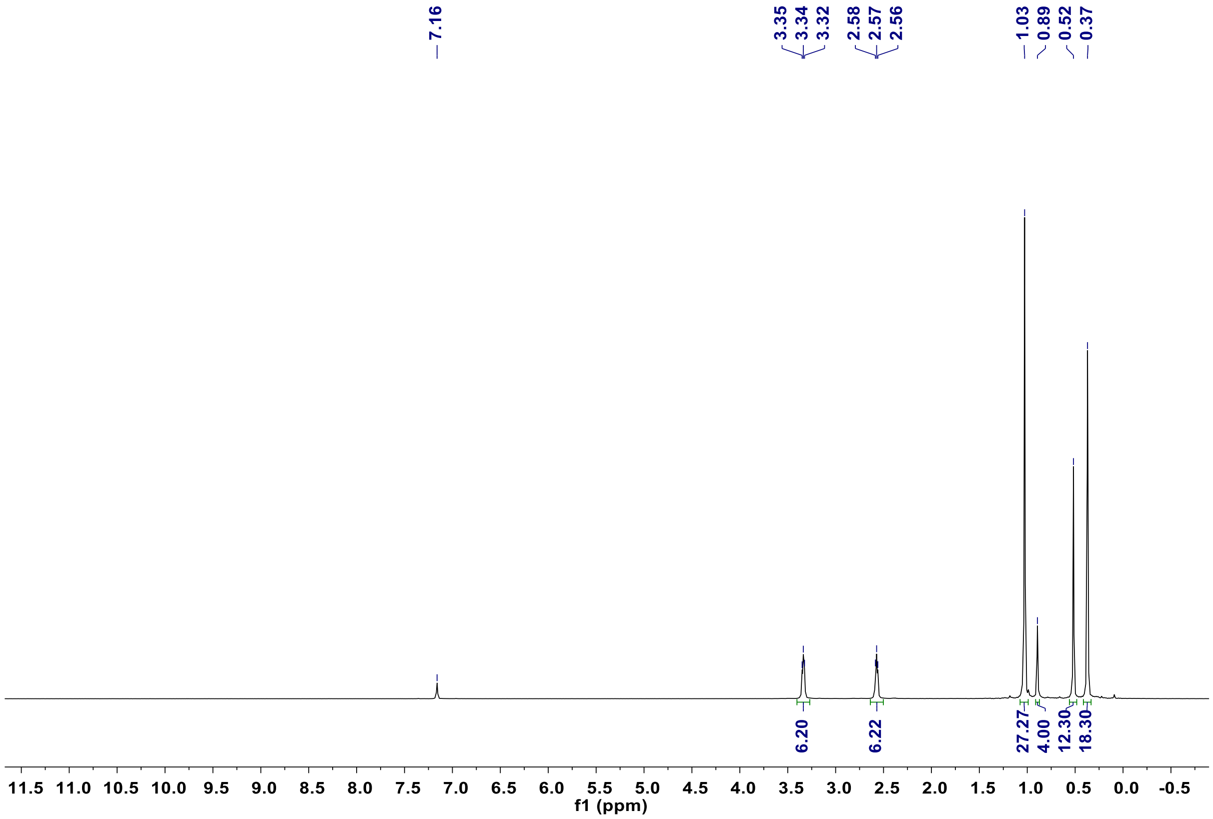

Supplementary Figure 23. ^{1}H NMR (400 MHz, C_6D_6, 298 K) of **10**.

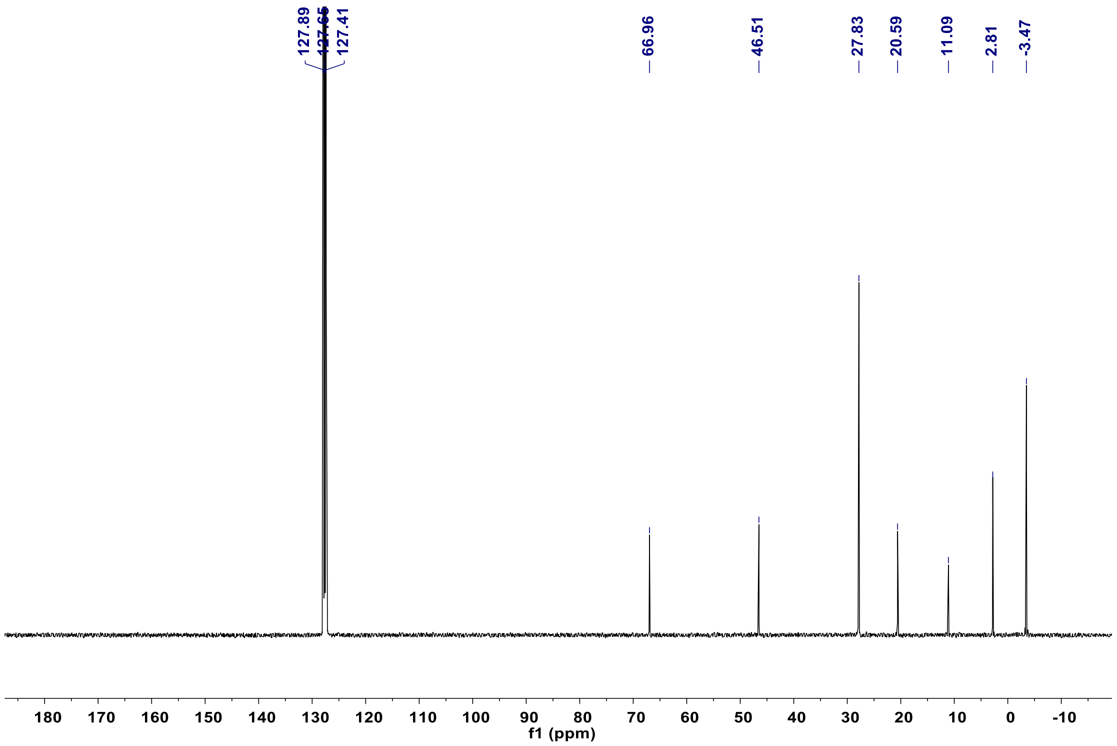

Supplementary Figure 24. $^{13}C\{^{1}H\}$ NMR (101 MHz, C_6D_6, 298 K) of **10**.

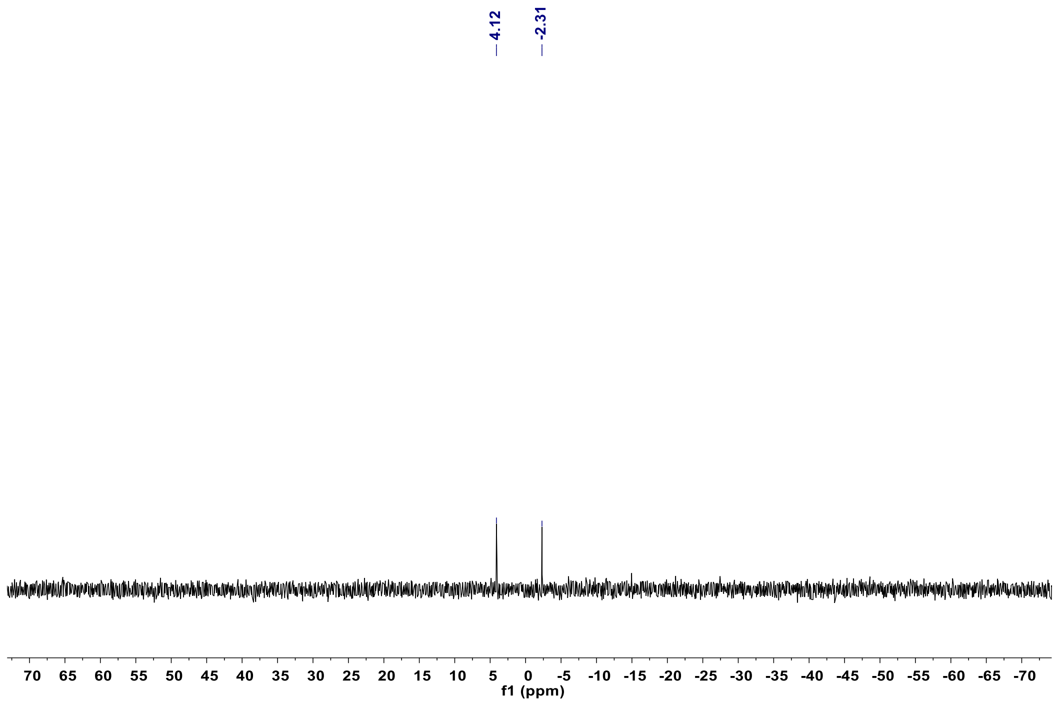

Supplementary Figure 25. ^{29}Si NMR{^{1}H} (79 MHz, C_6D_6, 298 K) of **10**.

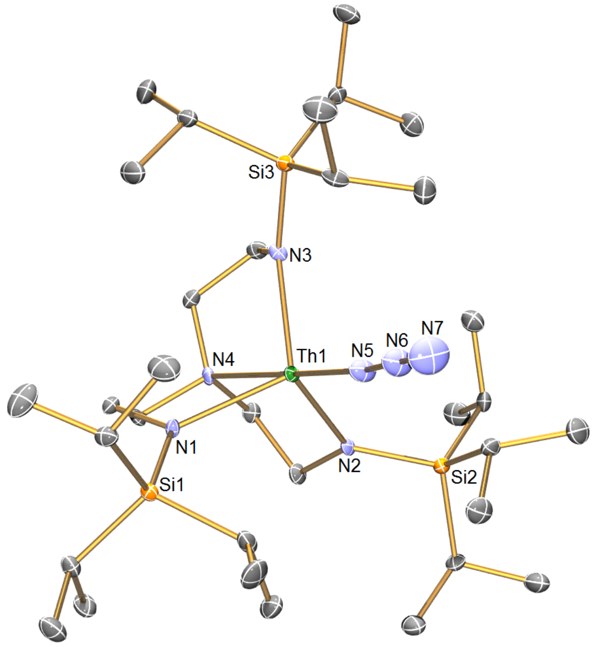

Supplementary Figure 26. Molecular structure of **2** with displacement ellipsoids at 40%. Hydrogen atoms, minor disorder components, and any lattice solvent omitted for clarity.

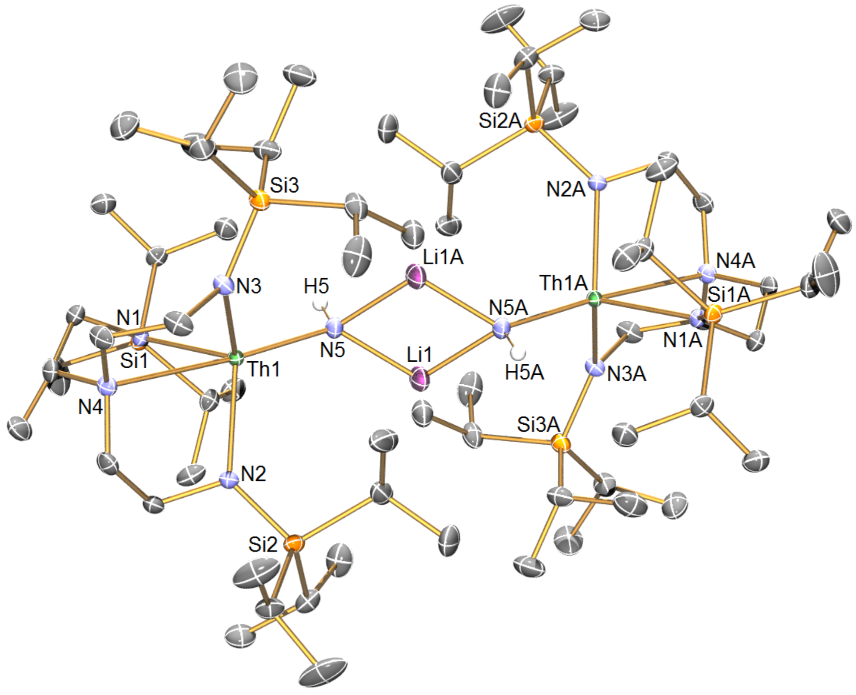

Supplementary Figure 27. Molecular structure of **4Li** with displacement ellipsoids at 40%. Non-imido hydrogen atoms, minor disorder components, and any lattice solvent omitted for clarity.

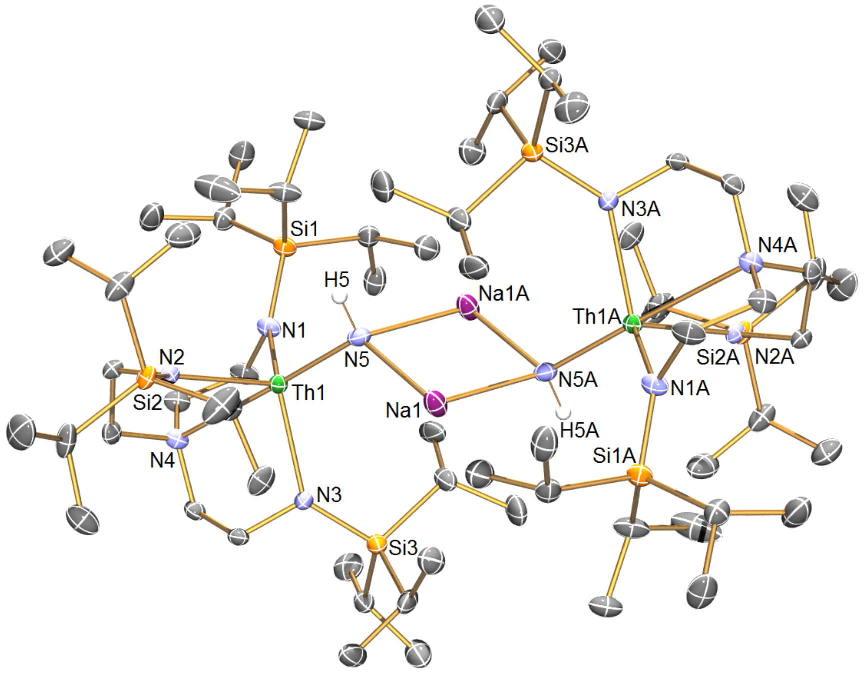

Supplementary Figure 28. Molecular structure of **4Na** with displacement ellipsoids at 40%. Non-imido hydrogen atoms, minor disorder components, and any lattice solvent omitted for clarity.

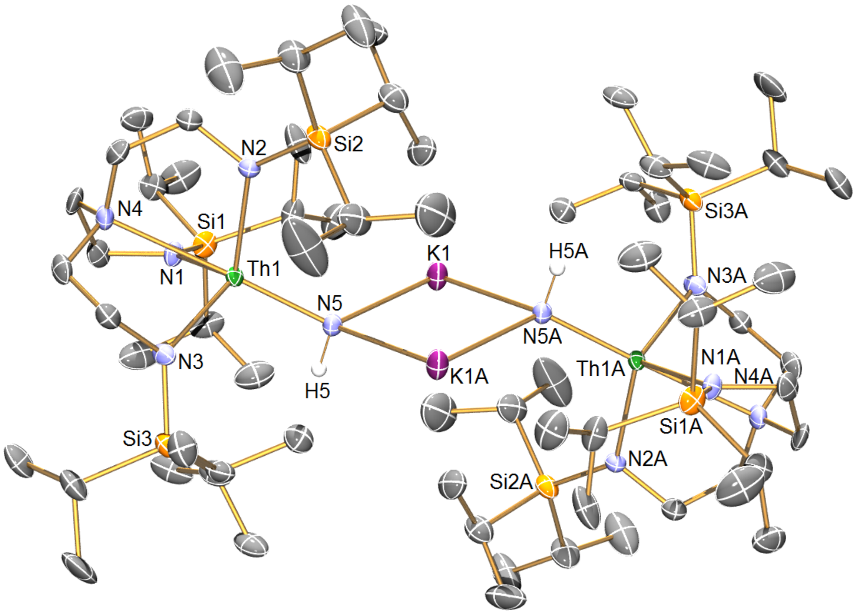

Supplementary Figure 29. Molecular structure of **4K** with displacement ellipsoids at 40%. Non-imido hydrogen atoms, minor disorder components, and any lattice solvent omitted for clarity.

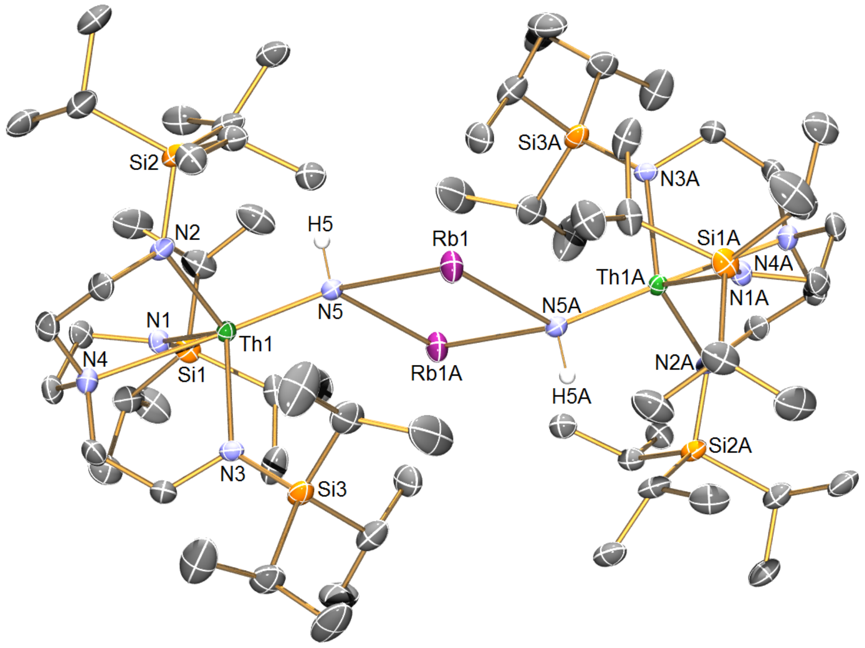

Supplementary Figure 30. Molecular structure of **4Rb** with displacement ellipsoids at 40%. Non-imido hydrogen atoms, minor disorder components, and any lattice solvent omitted for clarity.

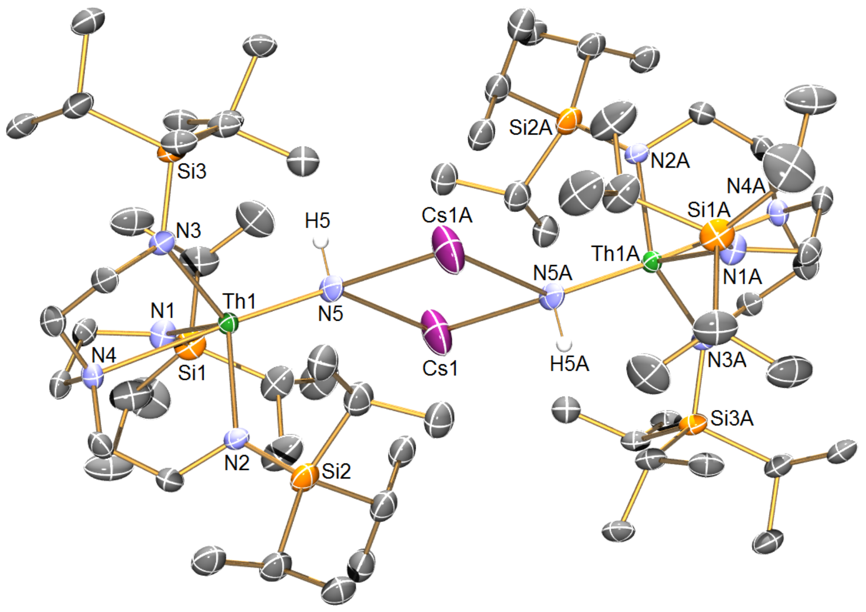

Supplementary Figure 31. Molecular structure of **4Cs** with displacement ellipsoids at 40%. Non-imido hydrogen atoms, minor disorder components, and any lattice solvent omitted for clarity.

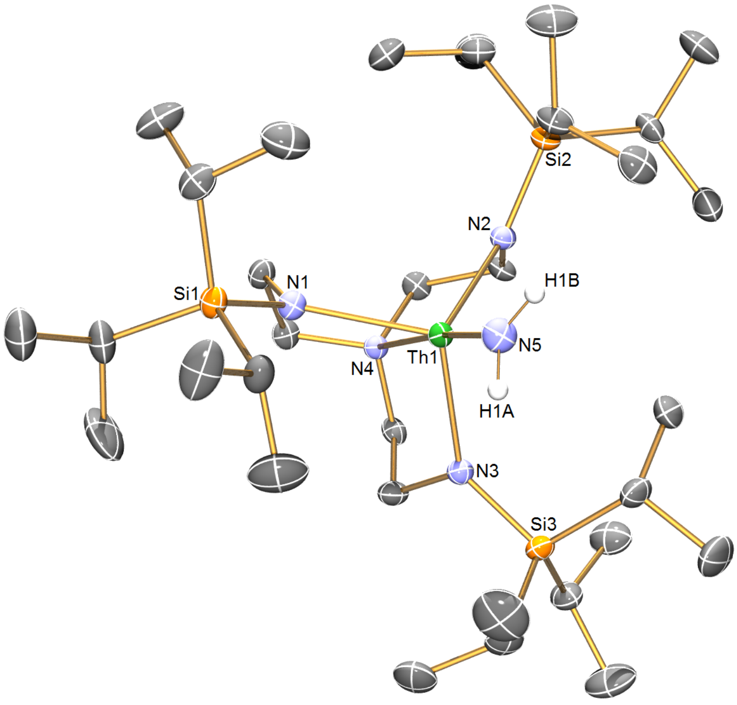

Supplementary Figure 32. Molecular structure of **5** with displacement ellipsoids at 40%. Non-amide hydrogen atoms, minor disorder components, and any lattice solvent omitted for clarity.

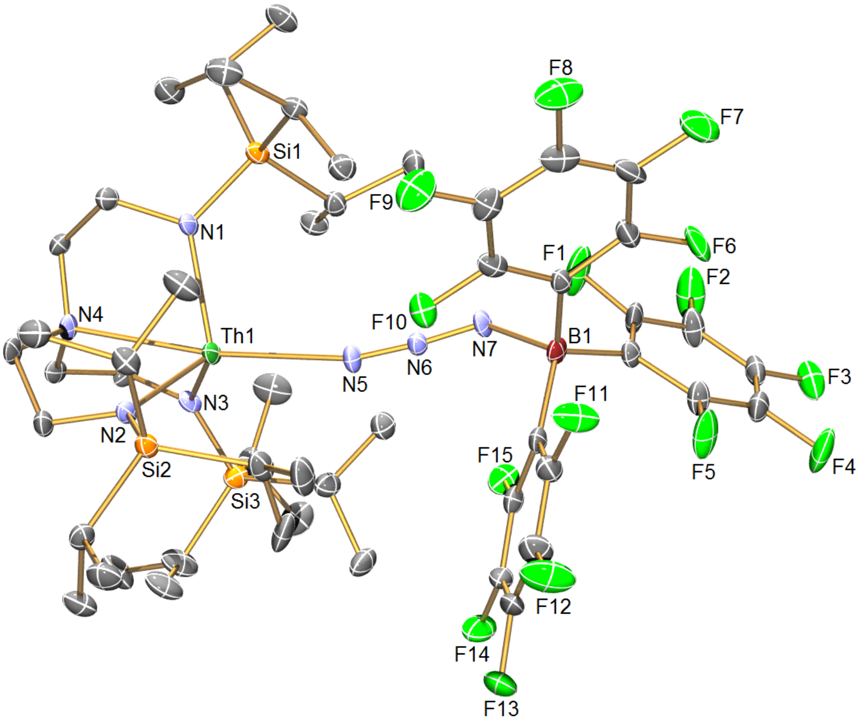

Supplementary Figure 33. Molecular structure of **6** with displacement ellipsoids at 40%. Hydrogen atoms, minor disorder components, and any lattice solvent omitted for clarity.

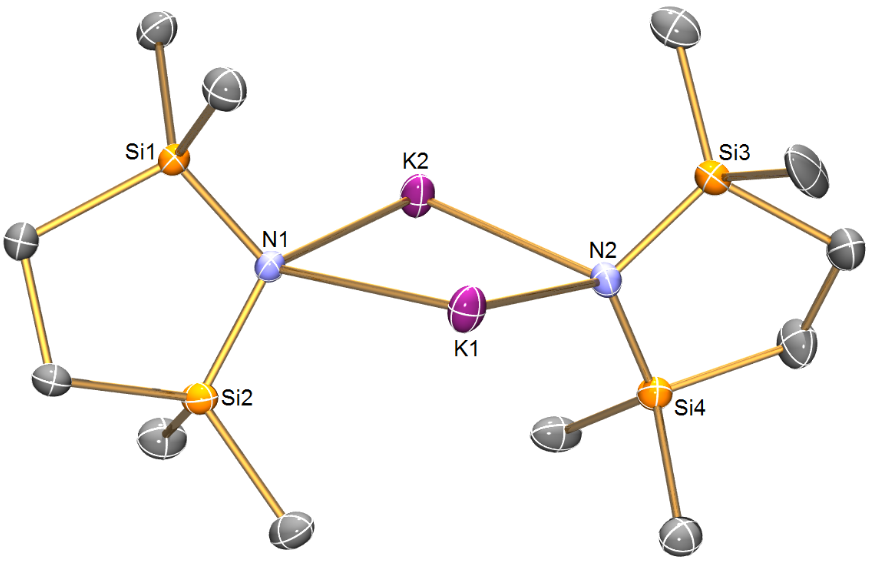

Supplementary Figure 34. Molecular structure of **8** with displacement ellipsoids at 40%. Hydrogen atoms, minor disorder components, and any lattice solvent omitted for clarity.

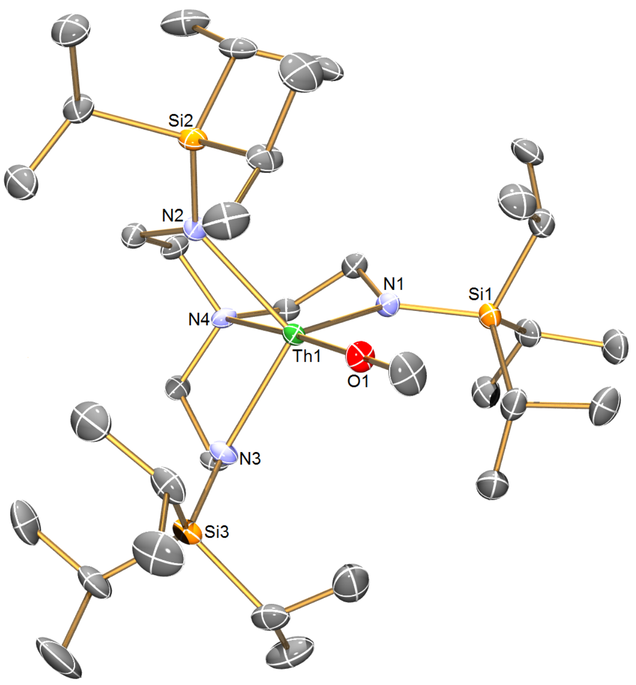

Supplementary Figure 35. Molecular structure of **9** with displacement ellipsoids at 40%. Hydrogen atoms, minor disorder components, and any lattice solvent omitted for clarity.

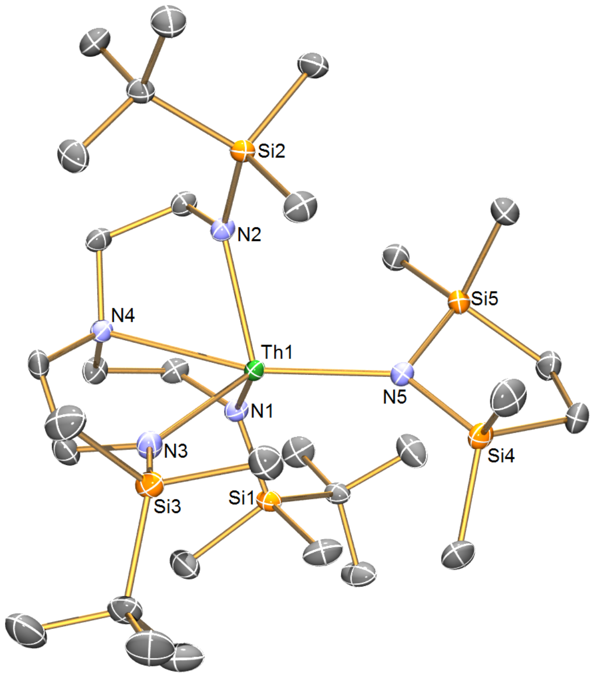

Supplementary Figure 36. Molecular structure of **10** with displacement ellipsoids at 40%. Hydrogen atoms, minor disorder components, and any lattice solvent omitted for clarity.

Computational Figures

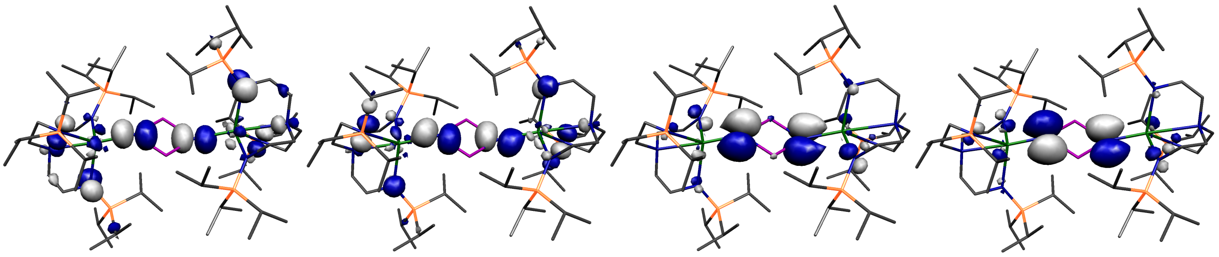

Supplementary Figure 37. Left to right: HOMO (−4.984 eV), HOMO−6 (−5.492 eV), HOMO−7 (−5.593 eV), and HOMO−8 (−5.699 eV) of **4Li** in the gas-phase (all-electron basis sets). Hydrogen atoms are omitted for clarity.

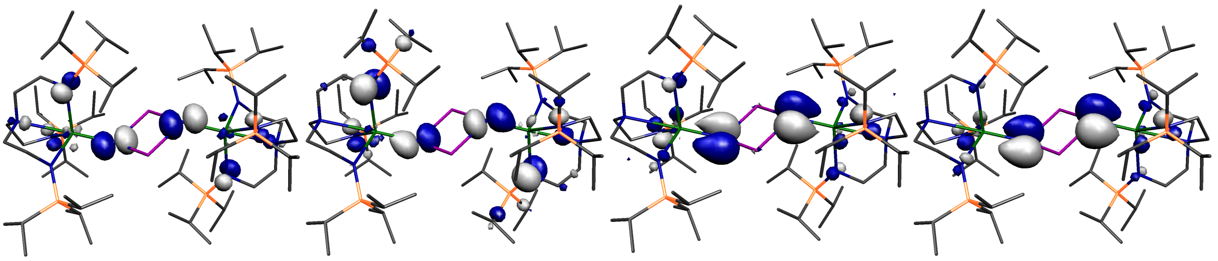

Supplementary Figure 38. Left to right: HOMO (−4.843 eV), HOMO−6 (−5.414 eV), HOMO−7 (−5.433 eV), and HOMO−8 (−5.538 eV) of **4Na** in the gas-phase (all-electron basis sets). Hydrogen atoms are omitted for clarity.

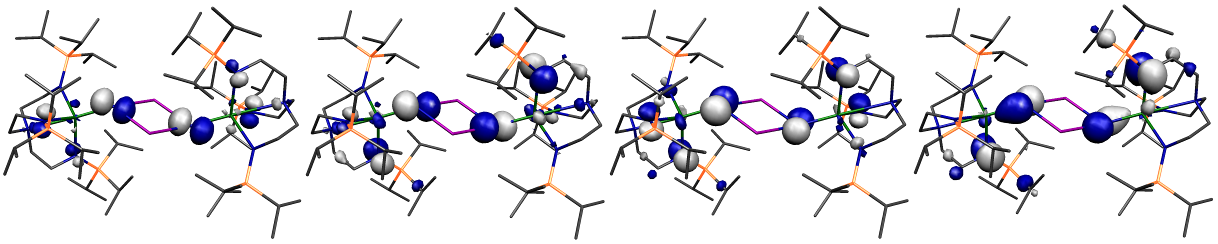

Supplementary Figure 39. Left to right: HOMO (−4.769 eV), HOMO−2 (−4.879 eV), HOMO−3 (−4.914 eV), and HOMO−8 (−5.315 eV) of **4K** in the gas-phase (all-electron basis sets). Hydrogen atoms are omitted for clarity.

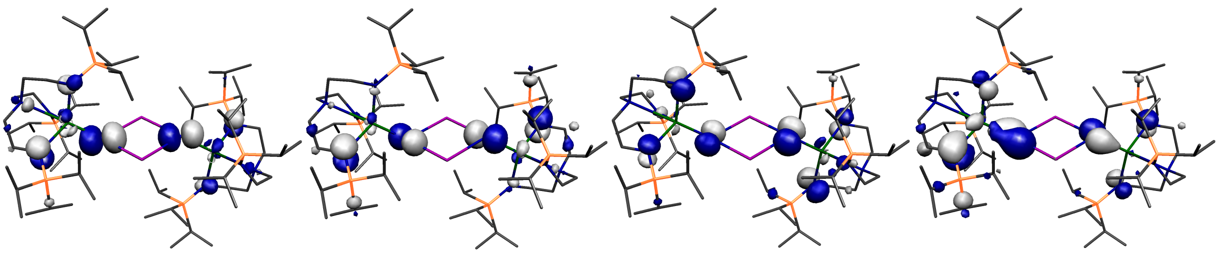

Supplementary Figure 40. Left to right: HOMO (−4.773 eV), HOMO−1 (−4.822 eV), HOMO−3 (−4.865 eV), and HOMO−6 (−5.064 eV) of **4Rb** in the gas-phase (all-electron basis sets). Hydrogen atoms are omitted for clarity.

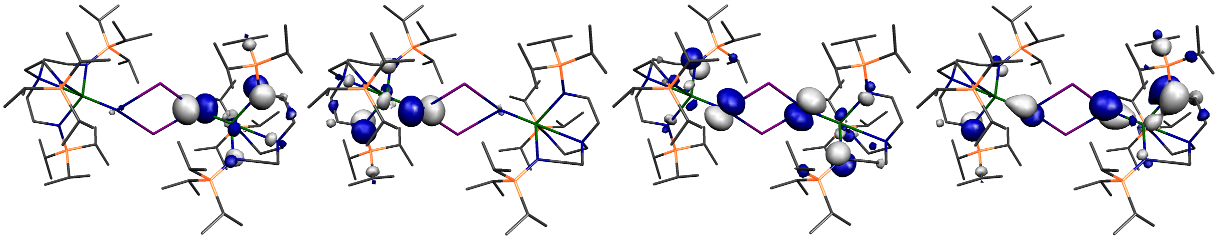

Supplementary Figure 41. Left to right: HOMO (−4.744 eV), HOMO−1 (−4.757 eV), HOMO−2 (−4.808 eV), and HOMO−6 (−5.172 eV) of **4Cs** in the gas-phase (all-electron basis sets). Hydrogen atoms are omitted for clarity. Hydrogen atoms are omitted for clarity.

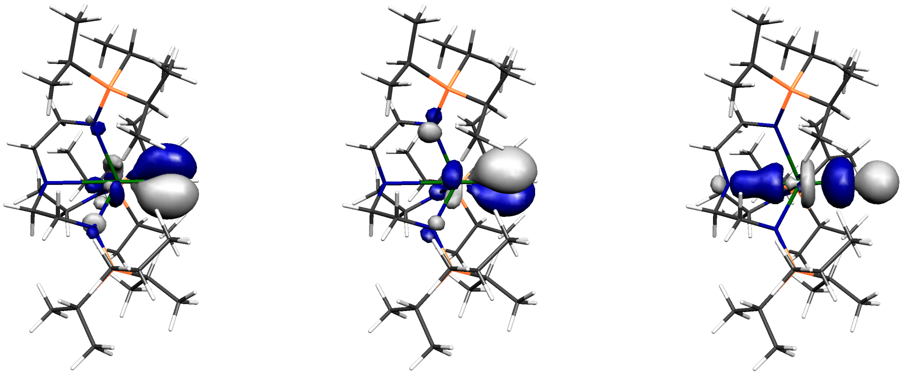

Supplementary Figure 42. Left to right: HOMO (−1.531 eV), HOMO−1 (−1.545 eV), and HOMO−15 (−4.880 eV) of [Th(Tren^{TIPS})(NH)]$^{-}$ in the gas-phase (all-electron basis sets).

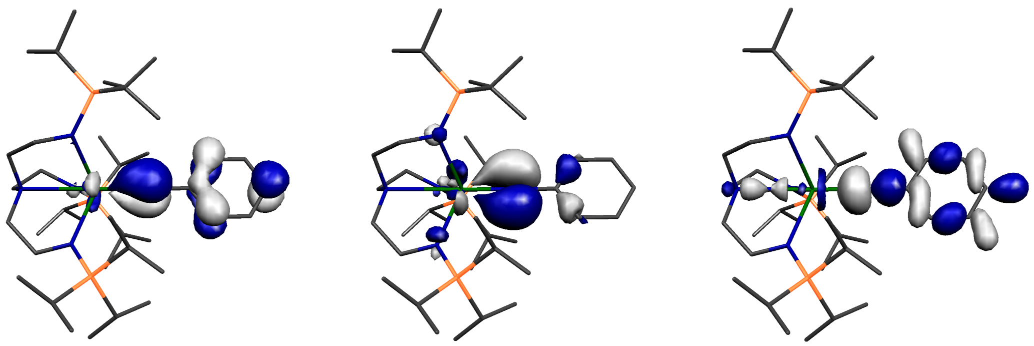

Supplementary Figure 43. Left to right: HOMO (−1.424 eV), HOMO−1 (−1.756 eV), and HOMO−18 (−5.196 eV) of [Th(Tren^{TIPS})(NPh)]$^{-}$ in the gas-phase (all-electron basis sets). Hydrogen atoms are omitted for clarity.

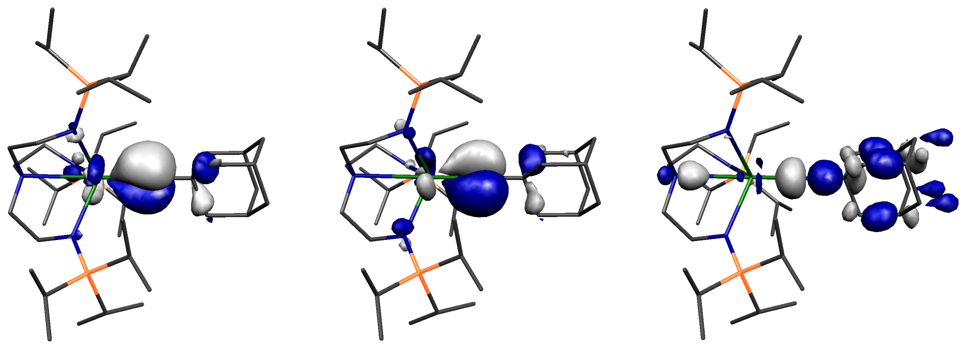

Supplementary Figure 44. Left to right: HOMO (−1.421 eV), HOMO−1 (−1.436 eV), and HOMO−15 (−4.323 eV) of [Th(Tren^{TIPS})(NAd)]$^{-}$ in the gas-phase (all-electron basis sets). Hydrogen atoms are omitted for clarity.

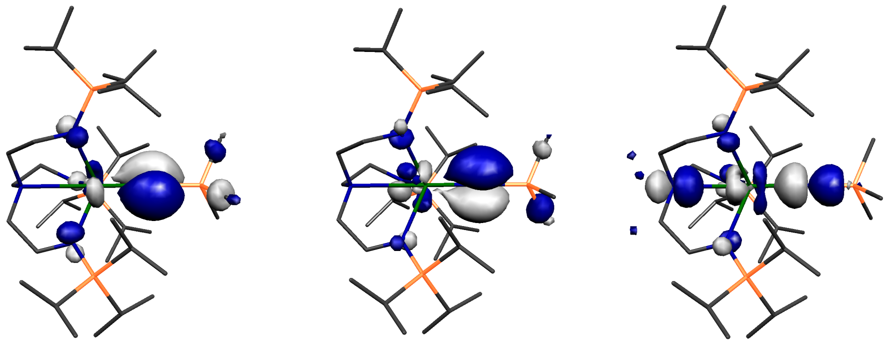

Supplementary Figure 45. Left to right: HOMO (−1.943 eV), HOMO−1 (−1.946 eV), and HOMO−5 (−3.203 eV) of [Th(Tren^{TIPS})(NSiMe$_3$)]$^{-}$ in the gas-phase (all-electron basis sets). Hydrogen atoms are omitted for clarity.

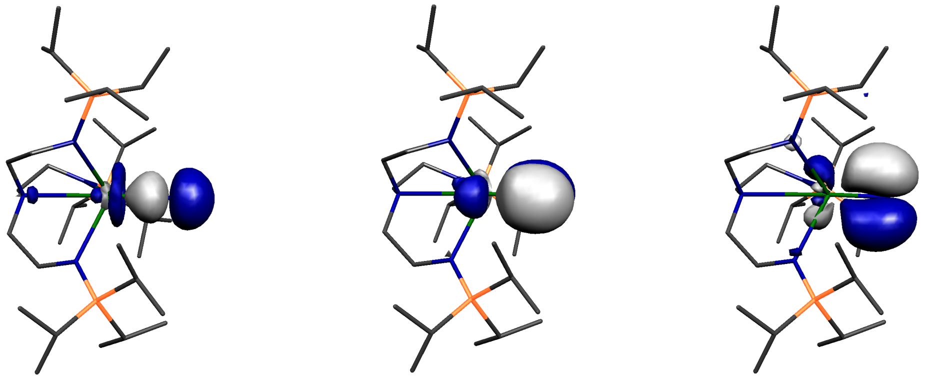

Supplementary Figure 46. Left to right: HOMO (3.539 eV), HOMO−1 (3.100 eV), and HOMO−2 (3.090 eV) of $[Th(Tren^{TIPS})(N)]^{2-}$ (**11**) in the gas-phase (up to 6p in frozen-core for Th, all other atoms all-electron). Hydrogen atoms are omitted for clarity.

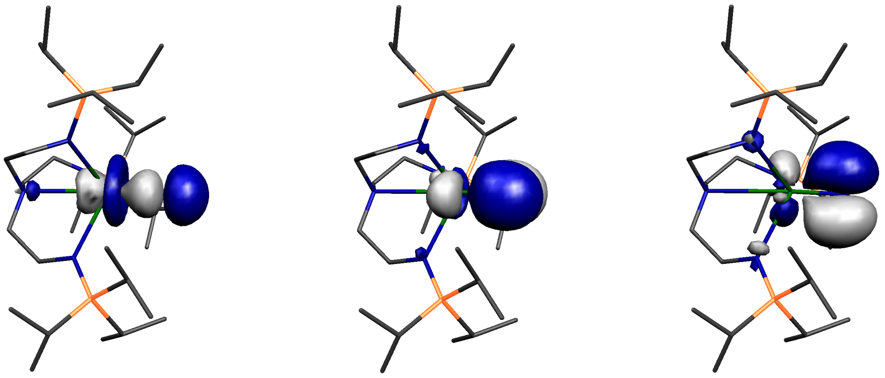

Supplementary Figure 47. Left to right: HOMO (3.286 eV), HOMO−1 (2.665 eV), and HOMO−2 (2.653 eV) of $[Th(Tren^{TIPS})(N)]^{2-}$ (**11**) in the gas-phase (up to 5d in frozen-core for Th, all other atoms all-electron). Hydrogen atoms are omitted for clarity.

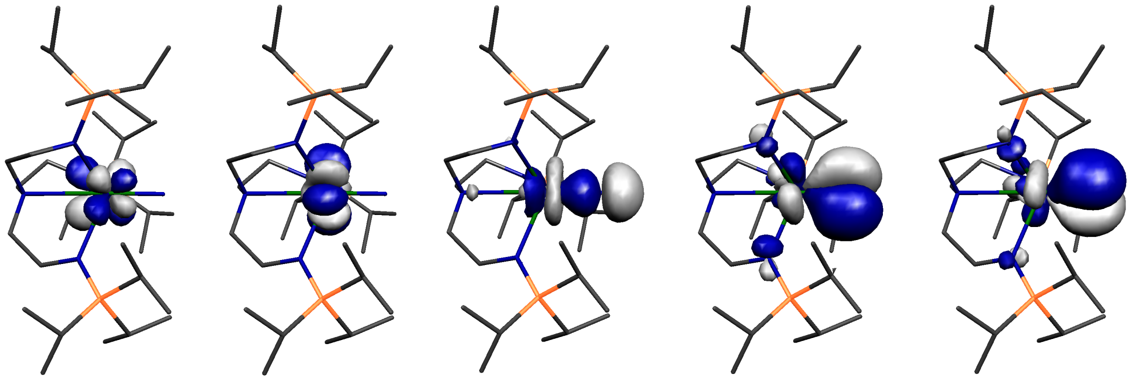

Supplementary Figure 48. Left to right: α-spin HOMO (3.988 eV), HOMO−1 (3.841 eV), HOMO−2 (1.904 eV), HOMO−3 (1.634 eV), and HOMO−4 (1.626 eV) of $[U(Tren^{TIPS})(N)]^{2-}$ (**12**) in the gas-phase (up to 6p in frozen-core for U, all other atoms all-electron). Hydrogen atoms are omitted for clarity.

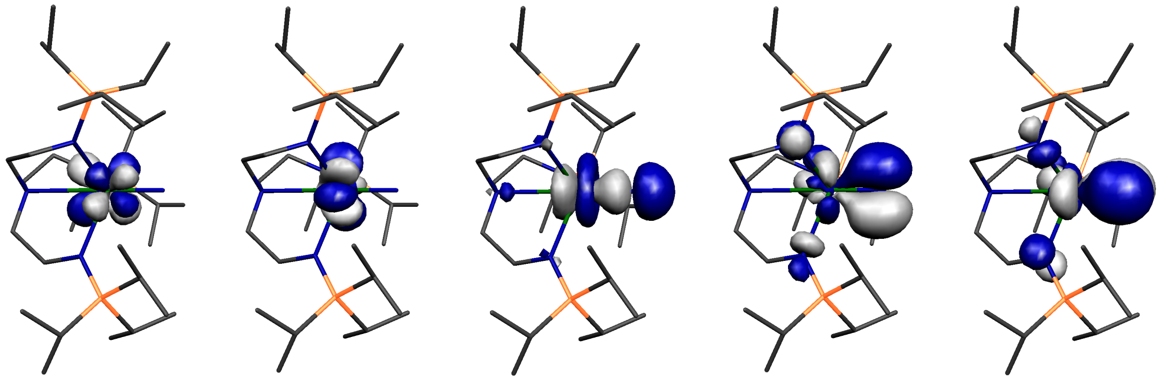

Supplementary Figure 49. Left to right: α-spin HOMO (3.955 eV), HOMO−1 (3.853 eV), HOMO−2 (1.688 eV), HOMO−3 (1.238 eV), and HOMO−4 (1.222 eV) of $[U(Tren^{TIPS})(N)]^{2-}$ (**12**) in the gas-phase (up to 5d in frozen-core for U, all other atoms all-electron). Hydrogen atoms are omitted for clarity.

Supplementary Tables

Supplementary Table 1 - Complex 2

1.C	-0.394683	-2.926632	-4.770826
2.C	0.822365	3.095419	-4.198288
3.C	0.978749	-0.868175	-4.265225
4.C	0.843245	-2.382329	-4.032677
5.C	0.798353	5.483757	-3.353117
6.C	0.167941	4.083445	-3.215125
7.C	1.744931	-5.534829	-2.755737
8.C	3.801181	-2.416411	-2.488215
9.C	-1.793756	1.639948	-2.212010
10.C	0.647831	-4.725616	-2.033516
11.C	-2.665060	-1.365267	-1.735839
12.C	2.631754	-2.244984	-1.496887
13.C	-2.981823	1.060254	-1.449959
14.C	-2.379622	5.096510	-1.151047
15.C	2.869307	2.756931	-1.337244
16.C	-1.685952	-2.462188	-1.317881
17.C	0.419056	-5.269486	-0.611545
18.C	-1.048920	4.751468	-0.454972
19.C	1.758877	3.421793	-0.498271
20.C	3.008640	-2.864302	-0.136037
21.C	2.251642	4.780776	0.039877
22.C	-3.450872	-0.462795	0.418016
23.C	-1.305495	4.331566	1.002702
24.C	-2.870158	0.263191	1.631293
25.C	0.227366	-3.166340	2.835945
26.C	0.550487	-1.733715	3.304426
27.C	-3.167754	-2.641831	3.668428
28.C	0.535683	1.772984	3.880541
29.C	-2.450795	-1.507765	4.424898
30.C	1.480441	-1.775752	4.534077
31.C	-0.551030	0.917928	4.555376
32.C	-1.766104	1.795042	4.914634
33.C	-2.015683	-1.994778	5.824473
34.H	-0.353428	-2.674423	-5.843404
35.H	0.677673	3.427751	-5.239499
36.H	0.973164	-0.624261	-5.339989
37.H	-0.482611	-4.019644	-4.696037
38.H	0.716085	5.847855	-4.390359
39.H	1.729818	-2.868692	-4.480505
40.H	-1.326345	-2.489782	-4.377799
41.H	1.905936	3.017357	-4.031168
42.H	0.404943	2.083644	-4.110046
43.H	1.898512	-5.205492	-3.793281
44.H	1.907201	-0.457579	-3.842149
45.H	0.140909	-0.323212	-3.801713
46.H	-0.889308	4.184635	-3.524326
47.H	3.622894	-1.910831	-3.446850
48.H	1.869907	5.468866	-3.102404
49.H	1.484701	-6.605462	-2.785721
50.H	0.316185	6.229277	-2.704957
51.H	-1.538431	0.970902	-3.056337
52.H	3.991462	-3.478047	-2.704383
53.H	-2.137136	2.575371	-2.685915
54.H	-0.290510	-4.897336	-2.594470
55.H	-2.372575	-0.994118	-2.727861
56.H	2.711285	-5.454513	-2.235379
57.H	-2.235246	5.465753	-2.176044
58.H	4.728356	-2.000796	-2.062480
59.H	3.179641	3.409335	-2.166608

60.H	-3.857234	0.909691	-2.111703
61.H	-1.787505	-3.286335	-2.045483
62.H	-3.700778	-1.750437	-1.811332
63.H	2.554233	1.798591	-1.775402
64.H	-3.050234	4.224738	-1.201671
65.H	2.525846	-1.152317	-1.329161
66.H	-2.920327	5.879777	-0.594203
67.H	2.422028	5.501855	-0.772956
68.H	0.134752	-6.334182	-0.643769
69.H	3.761870	2.563556	-0.722620
70.H	-3.272946	1.771600	-0.664341
71.H	-0.447326	5.678662	-0.430434
72.H	-2.012120	-2.889628	-0.350233
73.H	3.248032	-3.932294	-0.244407
74.H	-0.377657	-4.727864	-0.083688
75.H	1.326826	-5.195175	0.003014
76.H	-4.499535	-0.167042	0.220746
77.H	3.898109	-2.371530	0.286012
78.H	3.209001	4.656892	0.570598
79.H	1.597540	2.775445	0.387002
80.H	1.542931	5.237258	0.743931
81.H	2.200926	-2.781434	0.604692
82.H	-3.437212	-1.542010	0.621916
83.H	-1.877498	3.391008	1.047061
84.H	-1.889587	5.094930	1.542280
85.H	-0.373681	4.174568	1.565343
86.H	-2.947312	1.357197	1.478314
87.H	-0.422332	-3.182991	1.948873
88.H	-3.523679	0.047170	2.494425
89.H	1.149293	-3.713034	2.582160
90.H	1.142886	-1.249671	2.500386
91.H	-3.538660	-2.321484	2.685309
92.H	0.166692	2.193035	2.930827
93.H	-2.507106	-3.503798	3.503019
94.H	-0.276000	-3.739836	3.627960
95.H	1.448682	1.203960	3.657648
96.H	-4.034893	-3.003071	4.245442
97.H	-2.262400	2.182744	4.011203
98.H	2.383042	-2.367340	4.312505
99.H	0.824149	2.624488	4.517989
100.H	-3.198515	-0.709774	4.589941
101.H	1.812467	-0.776060	4.843832
102.H	0.987469	-2.245592	5.398203
103.H	-1.455438	2.669024	5.510169
104.H	-0.129714	0.527357	5.499853
105.H	-2.518954	1.250881	5.502346
106.H	-1.297722	-2.825748	5.754735
107.H	-2.882755	-2.365888	6.394587
108.H	-1.546133	-1.200206	6.421179
109.N	-0.625398	1.843098	-1.318091
110.N	-0.298619	-1.943563	-1.227548
111.N	-2.607326	-0.218082	-0.785404
112.N	2.148911	0.306354	0.863413
113.N	3.261543	0.489676	1.310725
114.N	4.324448	0.671931	1.738791
115.N	-1.469481	-0.158063	1.853868
116.Si	0.920631	-2.822708	-2.157702
117.Si	0.059430	3.476821	-1.388450
118.Si	-1.008700	-0.622279	3.496627
119.Th	-0.031500	0.013676	0.024028

Energy: −648.96843002 eV

Supplementary Table 2 - Complex 4Li

1.C	-0.369433	-1.825713	-7.510542
2.C	-2.723063	-0.939159	-7.312975
3.C	-1.614313	-1.739355	-6.602813
4.C	0.379442	1.120582	-5.479104
5.C	-2.023318	1.624557	-4.909031
6.C	-0.834462	0.685895	-4.635263
7.C	-4.916874	-2.194456	-4.224135
8.C	0.119938	-3.679043	-4.291429
9.C	0.354755	-2.156521	-4.292712
10.C	-3.470694	-2.667106	-4.048206
11.C	-4.774683	4.468946	-3.290228
12.C	-5.713265	0.804254	-3.242850
13.C	-6.419401	-0.552060	-3.148438
14.C	3.552827	1.421897	-3.111520
15.C	5.742538	2.671757	-2.934744
16.C	4.215893	2.758827	-2.740838
17.C	0.938233	-1.710340	-2.942072
18.C	-5.982724	-2.720803	-2.066833
19.C	4.183456	6.066799	-1.997053
20.C	1.097237	3.997734	-2.101520
21.C	-4.356584	4.099903	-1.853620
22.C	-2.831710	3.915668	-1.782402
23.C	5.670389	-1.407976	-1.478289
24.C	4.460251	-3.616726	-1.459098
25.C	-7.180560	3.073031	-1.266335
26.C	-8.216391	1.975295	-0.961502
27.C	4.432843	5.138289	-0.790555
28.C	1.786650	3.296865	-0.914596
29.C	-5.737305	-2.384935	-0.594138
30.C	-4.048672	-5.857796	-0.516697
31.C	-7.506108	4.335450	-0.438354
32.C	4.728288	-2.319442	-0.668563
33.C	-1.169987	-3.776127	-0.410614
34.C	7.506523	-4.335393	0.438294
35.C	4.048467	5.857401	0.516630
36.C	5.737403	2.385114	0.594216
37.C	1.169915	3.776156	0.410468
38.C	8.216507	-1.975290	0.962041
39.C	-4.432876	-5.138651	0.790511
40.C	-4.728131	2.319341	0.668811
41.C	7.180748	-3.073203	1.266569
42.C	-1.786696	-3.296967	0.914527
43.C	-5.670454	1.408076	1.478736
44.C	-4.459791	3.616543	1.459100
45.C	-4.183358	-6.067309	1.996963
46.C	4.356736	-4.100049	1.853888
47.C	2.831916	-3.915919	1.782203
48.C	5.982819	2.720846	2.066846
49.C	-1.097086	-3.997726	2.101515
50.C	-4.216044	-2.759290	2.741006
51.C	-5.742763	-2.672286	2.934934
52.C	6.419601	0.552142	3.148423
53.C	-0.938168	1.710629	2.940752
54.C	4.774445	-4.468715	3.290678
55.C	5.713475	-0.804184	3.242949
56.C	-3.553144	-1.422419	3.111945
57.C	3.471184	2.667288	4.048520
58.C	4.917189	2.194488	4.224169
59.C	-0.119701	3.678972	4.290831
60.C	-0.354408	2.156334	4.291487
61.C	0.834555	-0.685756	4.634807

62.C	2.023400	-1.624246	4.909184
63.C	-0.379729	-1.120476	5.478833
64.C	1.614343	1.739630	6.601931
65.C	2.723317	0.939754	7.311882
66.C	0.369239	1.825278	7.510057
67.H	-0.644001	-2.222660	-8.501809
68.H	-3.013604	-1.426059	-8.258268
69.H	0.088547	-0.839635	-7.675301
70.H	-2.380909	0.074716	-7.567605
71.H	0.406951	-2.485627	-7.100152
72.H	-3.629189	-0.835172	-6.699845
73.H	-1.985215	-2.776116	-6.499077
74.H	0.145284	1.087524	-6.553525
75.H	-2.350348	1.567274	-5.956698
76.H	1.263821	0.488511	-5.316289
77.H	0.667333	2.159234	-5.248463
78.H	-0.204573	-4.047785	-5.275113
79.H	-4.927609	-1.384693	-4.966729
80.H	-3.172191	-3.182973	-4.976320
81.H	1.112919	-1.932055	-5.063414
82.H	-5.572089	-3.006406	-4.598244
83.H	-1.748890	2.672896	-4.710753
84.H	-7.045485	-0.749196	-4.041288
85.H	-2.896996	1.388077	-4.283495
86.H	-5.062535	0.802996	-4.139932
87.H	5.991986	2.389341	-3.971024
88.H	-4.568673	3.645333	-3.990524
89.H	3.905169	1.057989	-4.091216
90.H	1.038597	-4.225879	-4.022821
91.H	-4.213631	5.347171	-3.650196
92.H	-6.483714	1.566877	-3.454169
93.H	-5.843763	4.712652	-3.366042
94.H	-0.566211	0.821336	-3.567069
95.H	3.837518	3.523116	-3.444141
96.H	-0.653600	-3.960026	-3.560990
97.H	-3.450424	-3.454865	-3.268483
98.H	2.458662	1.513765	-3.176471
99.H	4.527869	5.629073	-2.944013
100.H	6.246500	3.626930	-2.726200
101.H	1.491086	3.668510	-3.072964
102.H	1.287068	-0.664324	-2.989996
103.H	-7.082063	-0.534045	-2.273462
104.H	-7.059137	-2.881086	-2.274627
105.H	1.806474	-2.320675	-2.646491
106.H	-7.317254	3.352550	-2.327635
107.H	-5.456225	-3.655491	-2.301678
108.H	4.082283	-3.385679	-2.468847
109.H	5.239959	-1.168679	-2.463548
110.H	6.186132	1.909545	-2.277279
111.H	3.784169	0.641989	-2.370048
112.H	4.713247	7.025058	-1.866353
113.H	-2.508249	3.060077	-2.394505
114.H	-2.305910	4.806428	-2.164055
115.H	3.115482	6.305226	-2.112064
116.H	1.227479	5.088510	-2.047502
117.H	0.013563	3.803176	-2.097889
118.H	0.179767	-1.828021	-2.148272
119.H	-8.094612	1.094553	-1.604542
120.H	6.635169	-1.902627	-1.663742
121.H	5.380294	-4.204166	-1.591234
122.H	-9.238865	2.356563	-1.120197
123.H	-4.618062	4.952736	-1.201042

124.H	-4.161333	-5.212084	-1.399076
125.H	5.525964	4.969740	-0.750854
126.H	5.877493	-0.457748	-0.965380
127.H	3.723589	-4.268291	-0.971137
128.H	-1.639627	-3.315888	-1.294669
129.H	-8.529581	4.685525	-0.650623
130.H	7.457507	-4.128863	-0.641645
131.H	-4.673045	-6.752806	-0.672752
132.H	-6.823328	5.170487	-0.648036
133.H	1.565726	2.212564	-1.002413
134.H	-2.476515	3.742673	-0.756083
135.H	-3.002801	-6.197893	-0.492363
136.H	3.753857	-1.792512	-0.579471
137.H	-6.259464	-1.436356	-0.357904
138.H	-1.275854	-4.863535	-0.528605
139.H	-1.502506	1.346009	-0.611385
140.H	-8.157303	1.632153	0.080806
141.H	8.157457	-1.631945	-0.080206
142.H	-0.091704	-3.552865	-0.450610
143.H	-6.256248	-3.149883	0.009419
144.H	6.255862	3.150458	-0.009362
145.H	8.530145	-4.685199	0.650325
146.H	6.824021	-5.170682	0.647986
147.H	4.673028	6.752283	0.672979
148.H	6.259715	1.436810	0.357626
149.H	-7.457377	4.129075	0.641649
150.H	3.002702	6.197772	0.492009
151.H	9.239005	-2.356420	1.120798
152.H	0.091496	3.553398	0.450216
153.H	1.276259	4.863474	0.528656
154.H	-5.526002	-4.970168	0.750967
155.H	-3.753862	1.792090	0.579703
156.H	2.476967	-3.743149	0.755747
157.H	1.501348	-1.345185	0.610273
158.H	-5.877898	0.457898	0.965764
159.H	-3.722850	4.267831	0.971101
160.H	4.160699	5.211548	1.398929
161.H	4.618499	-4.953010	1.201523
162.H	-1.565839	-2.212642	1.002321
163.H	8.094474	-1.094662	1.605233
164.H	1.639178	3.315592	1.294583
165.H	-6.634986	1.902984	1.664305
166.H	-5.379661	4.204377	1.591052
167.H	-4.713676	-7.025296	1.866561
168.H	-3.115408	-6.306193	2.111489
169.H	7.317452	-3.352966	2.327818
170.H	7.059300	2.881043	2.274548
171.H	-1.227325	-5.088487	2.047444
172.H	-6.186343	-1.910033	2.277544
173.H	2.306011	-4.806590	2.163943
174.H	5.456481	3.655624	2.301867
175.H	-0.013466	-3.803034	2.097839
176.H	7.082277	0.534114	2.273446
177.H	-5.239855	1.168608	2.463803
178.H	-0.179795	1.827600	2.146243
179.H	-4.081974	3.385658	2.469008
180.H	2.508237	-3.060147	2.394001
181.H	-3.784359	-0.642395	2.370464
182.H	-6.246726	-3.627439	2.726285
183.H	-4.527133	-5.629387	2.944074
184.H	-1.805552	2.322617	2.646600
185.H	-1.490987	-3.668507	3.072897

186.H	5.843527	-4.712407	3.366865
187.H	-1.289028	0.665478	2.989382
188.H	6.483982	-1.566672	3.454449
189.H	3.450557	3.455404	3.269449
190.H	-2.458982	-1.514200	3.177094
191.H	-3.837712	-3.523706	3.444235
192.H	4.213319	-5.346883	3.650742
193.H	0.653909	3.960396	3.560636
194.H	-5.992137	-2.390009	3.971222
195.H	4.568240	-3.644936	3.990694
196.H	7.045720	0.749247	4.041237
197.H	0.566529	-0.821085	3.566886
198.H	-1.038385	4.225722	4.022479
199.H	5.062780	-0.802786	4.140046
200.H	-3.905705	-1.058564	4.091588
201.H	2.897836	-1.386386	4.285531
202.H	5.572401	3.006389	4.598163
203.H	1.749834	-2.672529	4.708822
204.H	4.928183	1.384685	4.966810
205.H	3.172359	3.182046	4.977099
206.H	-1.112770	1.931848	5.061832
207.H	0.204679	4.047124	5.274764
208.H	-0.668469	-2.158477	5.246797
209.H	-1.263413	-0.487471	5.316990
210.H	2.348450	-1.568524	5.957496
211.H	1.984643	2.776510	6.498258
212.H	-0.145025	-1.089028	6.552976
213.H	3.629105	0.835458	6.698422
214.H	-0.407656	2.484221	7.099366
215.H	2.381287	-0.073971	7.567226
216.H	-0.087491	0.838917	7.675160
217.H	3.014492	1.427130	8.256951
218.H	0.643848	2.222899	8.500862
219.Li	0.461293	-0.042597	-1.061024
220.Li	-0.463464	0.042337	1.061326
221.N	-2.577772	-1.536685	-3.697651
222.N	-5.438793	-1.650197	-2.944361
223.N	-4.941647	1.092603	-2.009526
224.N	-1.451988	0.307921	-0.644488
225.N	-4.285829	-2.293980	-0.289000
226.N	4.285784	2.293811	0.289069
227.N	1.450494	-0.307244	0.645108
228.N	4.941862	-1.092752	2.009693
229.N	5.438973	1.650228	2.944302
230.N	2.577791	1.536904	3.697064
231.Si	-1.245222	-1.188117	-4.783348
232.Si	-5.311484	2.578951	-1.150663
233.Si	3.706835	3.354616	-0.978781
234.Si	-3.706787	-3.355012	0.978956
235.Si	5.311690	-2.579056	1.150976
236.Si	1.245510	1.188280	4.782705
237.Th	-3.272968	-0.566517	-1.605872
238.Th	3.272708	0.566490	1.605926

Energy: −1279.46096334 eV

Supplementary Table 3 - Complex 4Na

1.C	3.649414	-2.930770	-5.339240
2.C	-7.136471	2.022314	-4.791452
3.C	2.335576	-5.032271	-4.880510
4.C	2.890615	3.164443	-4.904912
5.C	-3.371878	1.782105	-4.655961
6.C	3.198729	-3.921946	-4.247510

7.C	-5.530649	-0.806065	-4.046989
8.C	-6.818152	-1.623729	-3.824149
9.C	-4.303240	-1.732454	-4.024768
10.C	1.443544	-0.625522	-3.869638
11.C	5.285983	5.666218	-3.413600
12.C	-6.830659	1.888543	-3.285974
13.C	-0.209013	-2.519062	-3.701728
14.C	2.614791	3.659395	-3.472532
15.C	5.245106	0.929383	-3.190971
16.C	5.587989	4.227873	-2.946200
17.C	-3.632852	1.458282	-3.171464
18.C	1.251330	3.135776	-2.987219
19.C	-6.725327	3.286618	-2.646922
20.C	1.024988	-1.860905	-3.054699
21.C	6.535934	0.335762	-2.612516
22.C	6.822419	4.228892	-2.023465
23.C	6.079282	-2.074863	-2.306480
24.C	-3.273145	2.679389	-2.309119
25.C	4.980813	-2.893074	-1.618685
26.C	1.633999	-4.539152	-1.660373
27.C	2.660111	-5.584680	-1.182651
28.C	-6.750933	-0.138979	-0.608605
29.C	7.195099	-0.795574	-0.515230
30.C	-4.516536	-3.661205	-0.614521
31.C	2.986035	5.314176	-0.502750
32.C	3.486806	3.855495	-0.521211
33.C	0.805161	-4.038757	-0.465109
34.C	-7.193336	0.800907	0.517062
35.C	-2.993367	-5.321178	0.509114
36.C	6.752004	0.141907	0.612218
37.C	-0.801092	4.024583	0.454327
38.C	4.505122	3.645590	0.613430
39.C	-3.498032	-3.863663	0.521408
40.C	-2.643054	5.583980	1.176015
41.C	-4.976534	2.897609	1.620502
42.C	-1.621839	4.532896	1.651945
43.C	-6.839339	-4.221982	2.017063
44.C	-6.076185	2.080538	2.307711
45.C	6.717743	-3.290844	2.647706
46.C	3.266070	-2.670492	2.310354
47.C	-6.535012	-0.329690	2.614823
48.C	-5.606768	-4.223708	2.942239
49.C	-5.311323	-5.662167	3.413231
50.C	6.829226	-1.893137	3.286402
51.C	-1.267597	-3.140708	2.987060
52.C	-5.244566	-0.926832	3.189927
53.C	3.632275	-1.453316	3.175514
54.C	-1.023322	1.854122	3.051159
55.C	-2.630733	-3.664190	3.473199
56.C	6.827787	1.618254	3.828905
57.C	0.218749	2.506125	3.688201
58.C	5.538696	0.803558	4.052679
59.C	4.313878	1.733839	4.036752
60.C	-1.446370	0.625419	3.874243
61.C	-3.184173	3.931207	4.242481
62.C	7.139458	-2.027861	4.790793
63.C	3.374459	-1.781303	4.659771
64.C	-2.310805	5.035799	4.872454
65.C	-2.907338	-3.166442	4.904400
66.C	-3.642808	2.946570	5.336531
67.H	4.297688	-3.428395	-6.078910
68.H	2.786174	-2.531249	-5.892015

69.H	2.866604	-5.500643	-5.725905
70.H	2.072502	3.444815	-5.589353
71.H	-6.298890	2.479312	-5.339377
72.H	-7.350346	1.054192	-5.264031
73.H	1.388751	-4.635891	-5.277317
74.H	-8.016257	2.667813	-4.952919
75.H	3.819377	3.585050	-5.317284
76.H	-3.575123	0.928474	-5.316950
77.H	-5.593524	-0.365091	-5.058978
78.H	-3.997168	2.620380	-4.998079
79.H	2.980782	2.068437	-4.936711
80.H	4.203932	-2.073168	-4.932574
81.H	-6.913963	-2.420664	-4.580230
82.H	1.740964	-0.897249	-4.891852
83.H	-4.443589	-2.592208	-4.701211
84.H	-2.322426	2.080050	-4.816618
85.H	0.013552	-2.865696	-4.721679
86.H	2.086282	-5.831213	-4.169058
87.H	6.182298	6.121916	-3.866393
88.H	-7.726087	-1.005436	-3.884390
89.H	-3.386757	-1.215333	-4.342617
90.H	4.484737	5.707323	-4.164151
91.H	5.870190	3.661671	-3.853728
92.H	5.533876	1.642122	-3.983545
93.H	4.114110	-4.416318	-3.871020
94.H	0.604407	0.084330	-3.967788
95.H	4.699253	0.123916	-3.722499
96.H	-1.041879	-1.801955	-3.783312
97.H	7.205425	-0.034074	-3.414502
98.H	2.545025	4.761402	-3.507024
99.H	0.449688	3.377742	-3.705138
100.H	5.781396	-1.906823	-3.350077
101.H	2.291369	-0.081057	-3.424293
102.H	-5.968594	3.901309	-3.157265
103.H	-7.713045	1.398415	-2.831561
104.H	-6.813177	-2.112971	-2.838526
105.H	-7.681142	3.830364	-2.723771
106.H	7.719621	4.569328	-2.565715
107.H	-4.124594	-2.135461	-3.017844
108.H	-0.576106	-3.384718	-3.133948
109.H	4.986858	6.312498	-2.575043
110.H	1.286865	2.037946	-2.894253
111.H	-2.940513	0.643550	-2.880819
112.H	-3.885650	3.554889	-2.565505
113.H	7.046336	-2.615526	-2.316296
114.H	7.070190	1.128966	-2.072845
115.H	0.937966	-5.045354	-2.353067
116.H	-2.221522	2.976815	-2.467551
117.H	7.039954	3.235582	-1.607479
118.H	3.236828	-6.014740	-2.014467
119.H	5.005459	-3.913221	-2.041726
120.H	0.969823	3.571226	-2.016201
121.H	0.714870	-1.496934	-2.052881
122.H	-6.450660	3.243323	-1.582787
123.H	-7.549111	-0.141569	-1.372172
124.H	6.682678	4.914507	-1.174842
125.H	-4.070657	-3.924174	-1.586902
126.H	2.230478	5.517006	-1.274733
127.H	-3.408132	2.512757	-1.227229
128.H	8.217623	-0.554486	-0.868002
129.H	-1.627091	-0.970731	-1.118503
130.H	2.163318	-6.420295	-0.662023

131.H	3.813710	6.021381	-0.661127
132.H	-4.875871	-2.623079	-0.689864
133.H	5.258979	-3.026013	-0.553700
134.H	-0.029905	-3.394468	-0.784123
135.H	-5.401180	-4.300182	-0.483661
136.H	3.377247	-5.141456	-0.475765
137.H	-2.545631	-5.571950	-0.466304
138.H	-6.733115	-1.175861	-0.216277
139.H	7.211819	-1.821534	-0.122148
140.H	2.619691	3.202582	-0.295036
141.H	-7.208782	1.826259	0.122485
142.H	-1.449698	3.467440	-0.241691
143.H	-0.361140	4.860507	-0.113529
144.H	6.733640	1.179593	0.222097
145.H	0.368321	-4.878822	0.099303
146.H	5.394168	4.278508	0.482215
147.H	1.446539	-3.479186	0.236025
148.H	-2.632416	-3.209558	0.293161
149.H	2.537327	5.561464	0.473101
150.H	-3.366081	5.143751	0.473369
151.H	-3.818801	-6.029894	0.672377
152.H	-8.216159	0.561459	0.870201
153.H	-5.254788	3.032237	0.555492
154.H	-2.142839	6.414879	0.650927
155.H	4.858134	2.605168	0.688135
156.H	0.030599	3.375600	0.772692
157.H	-6.700854	-4.910363	1.170460
158.H	-2.235931	-5.518354	1.280673
159.H	7.550570	0.143351	1.375549
160.H	1.622399	0.972651	1.112094
161.H	3.398257	-2.501537	1.228459
162.H	6.441257	-3.246753	1.584231
163.H	4.061926	3.910592	1.586452
164.H	-7.052030	-3.228788	1.597965
165.H	-3.213145	6.020142	2.009079
166.H	-4.999410	3.917063	2.044789
167.H	-7.042614	2.622276	2.316894
168.H	-7.072781	-1.121400	2.076410
169.H	-5.014782	-6.311741	2.576398
170.H	-7.738955	-4.557259	2.558442
171.H	-0.984139	-3.580580	2.018546
172.H	7.672039	-3.837894	2.722757
173.H	-0.919638	5.036137	2.340883
174.H	-0.722138	1.483173	2.049113
175.H	2.213712	-2.965011	2.471016
176.H	3.876291	-3.548824	2.562281
177.H	7.711813	-1.405739	2.829144
178.H	6.821257	2.110951	2.845075
179.H	5.960186	-3.902892	3.159870
180.H	2.941830	-0.635264	2.889805
181.H	4.134310	2.141081	3.031548
182.H	-1.305828	-2.043450	2.887560
183.H	-5.778974	1.912384	3.351367
184.H	-7.201460	0.041967	3.418267
185.H	0.588799	3.366811	3.114701
186.H	-6.209971	-6.112880	3.866722
187.H	-2.561052	-4.766295	3.509704
188.H	-2.299030	0.084027	3.434792
189.H	-4.510783	-5.705041	4.164282
190.H	7.734269	0.997112	3.884107
191.H	-5.888551	-3.654344	3.847852
192.H	-4.694421	-0.122066	3.718456

193.H	-4.095273	4.432955	3.865299
194.H	-0.466613	-3.377269	3.707404
195.H	-5.532627	-1.637197	3.984821
196.H	1.047043	1.783618	3.768368
197.H	-2.055715	5.831043	4.158950
198.H	-0.611433	-0.089151	3.973099
199.H	6.928352	2.412032	4.587519
200.H	3.396725	1.219018	4.355161
201.H	8.018271	-2.674933	4.949566
202.H	4.459182	2.590895	4.715706
203.H	0.004390	2.858754	4.707863
204.H	-4.205780	2.093499	4.931986
205.H	2.324916	-2.078335	4.822062
206.H	7.356547	-1.060192	5.263055
207.H	3.999377	-2.621485	4.997754
208.H	5.602830	0.359812	5.063416
209.H	-1.738381	0.904540	4.896051
210.H	-2.996070	-2.070097	4.934034
211.H	6.302371	-2.483180	5.341360
212.H	-3.836901	-3.584988	5.316783
213.H	3.580481	-0.929967	5.322668
214.H	-1.367108	4.632329	5.269087
215.H	-2.837287	5.510390	5.717203
216.H	-2.090095	-3.446344	5.590132
217.H	-4.285743	3.451633	6.075957
218.H	-2.782894	2.539897	5.889221
219.N	4.402480	1.542524	-2.136032
220.N	6.229604	-0.751023	-1.645867
221.N	3.654370	-2.238841	-1.746454
222.N	-5.426702	0.250721	-1.150925
223.N	-1.795055	-0.363342	-0.295428
224.N	1.794188	0.366837	0.288773
225.N	5.428254	-0.249485	1.154319
226.N	-6.227820	0.756450	1.647502
227.N	-3.651345	2.240857	1.747298
228.N	-4.406750	-1.544338	2.133406
229.Na	-0.096863	1.209353	-0.891265
230.Na	0.099057	-1.206818	0.887609
231.Si	-5.369936	0.693963	-2.844781
232.Si	2.443691	-3.100022	-2.664846
233.Si	4.063148	3.255941	-2.254196
234.Si	-4.076197	-3.259364	2.252169
235.Si	-2.436513	3.100411	2.661507
236.Si	5.371466	-0.693809	2.847878
237.Th	3.751200	-0.145694	-0.553395
238.Th	-3.749690	0.146224	0.555416

Energy: −1277.56325620 eV

Supplementary Table 4 - Complex 4K

1.C	1.330966	2.816854	-6.697138
2.C	3.809371	2.503168	-6.327767
3.C	2.420059	1.854146	-6.175155
4.C	0.269279	-0.776260	-5.848057
5.C	4.042493	-0.707224	-4.826601
6.C	5.525257	-0.417335	-4.573062
7.C	0.258370	0.252378	-4.699590
8.C	4.588056	-5.736505	-3.919482
9.C	6.322980	-2.353341	-3.283590
10.C	-4.758156	-0.705471	-3.476459
11.C	-0.248268	-0.411044	-3.409217
12.C	1.557094	-3.886772	-3.276791
13.C	-8.108217	-2.036556	-2.539895

14.C	2.951979	3.400373	-2.956953
15.C	1.769986	2.415211	-3.036621
16.C	0.433688	3.180433	-3.022720
17.C	7.011916	-0.084671	-2.638654
18.C	4.443124	-5.958660	-2.401137
19.C	3.737195	-7.304551	-2.129464
20.C	-5.722861	1.255529	-2.213888
21.C	5.985710	-3.047980	-1.962644
22.C	-5.014922	-0.107085	-2.077927
23.C	6.421225	1.103676	-1.876712
24.C	1.837359	-4.112481	-1.778651
25.C	-3.184073	4.011230	-1.416353
26.C	-7.782599	-1.496429	-1.130384
27.C	-8.658580	-0.266277	-0.821393
28.C	-5.245069	5.372556	-0.883898
29.C	-5.128637	-3.060171	-1.062361
30.C	0.822865	-5.110880	-1.187823
31.C	-3.598064	-3.063795	-0.895919
32.C	-3.799162	5.045551	-0.457588
33.C	-5.765135	-4.107351	-0.126855
34.C	5.767901	4.105881	0.125464
35.C	3.794201	-5.037588	0.467310
36.C	8.659430	0.263889	0.822760
37.C	5.238229	-5.364286	0.899540
38.C	3.600537	3.062662	0.893993
39.C	5.130689	3.060473	1.062549
40.C	7.784698	1.495537	1.129822
41.C	-0.821276	5.110032	1.192916
42.C	3.178220	-3.999988	1.421322
43.C	-1.836657	4.110262	1.779422
44.C	-6.420333	-1.104494	1.876911
45.C	-3.731174	7.302762	2.147696
46.C	-5.985719	3.048481	1.970281
47.C	5.015727	0.107903	2.077940
48.C	-4.439108	5.957153	2.415375
49.C	5.724091	-1.254226	2.214848
50.C	8.112253	2.038548	2.537746
51.C	-7.010781	0.082835	2.640825
52.C	-2.943839	-3.406883	2.955894
53.C	-0.426501	-3.185183	3.022525
54.C	-1.763157	-2.419978	3.034919
55.C	-6.321175	2.350589	3.290166
56.C	-1.551413	3.875677	3.275559
57.C	4.757850	0.706639	3.475949
58.C	0.244110	0.412492	3.399067
59.C	-4.583512	5.730377	3.932665
60.C	-5.521588	0.412397	4.574420
61.C	-0.257631	-0.248359	4.692365
62.C	-4.038428	0.701403	4.825936
63.C	-0.274795	0.785632	5.836239
64.C	-2.412438	-1.857756	6.171905
65.C	-3.802031	-2.506425	6.327686
66.C	-1.322317	-2.821285	6.690142
67.H	1.541469	3.115782	-7.738443
68.H	3.988402	2.790344	-7.378305
69.H	2.406502	0.974337	-6.847630
70.H	0.326849	2.368870	-6.682314
71.H	0.518460	-0.316967	-6.815398
72.H	1.291222	3.742234	-6.100819
73.H	4.624786	1.830593	-6.030538
74.H	3.902834	3.417722	-5.723977
75.H	-0.716486	-1.259772	-5.960575

76.H	3.823643	-0.415491	-5.868364
77.H	6.166989	-0.845247	-5.370356
78.H	1.003724	-1.575440	-5.659689
79.H	-0.457186	1.051972	-4.966873
80.H	3.893348	-1.804590	-4.804341
81.H	5.664670	0.671952	-4.579291
82.H	5.154393	-6.562396	-4.383065
83.H	3.609476	-5.696861	-4.420595
84.H	5.113473	-4.802378	-4.158288
85.H	5.735479	-2.830091	-4.079266
86.H	-4.174889	-0.006038	-4.099259
87.H	-5.699577	-0.902417	-4.011769
88.H	2.947488	4.088508	-3.815709
89.H	7.395304	-2.465647	-3.542607
90.H	0.325998	3.814702	-3.915220
91.H	1.662610	-4.822716	-3.844730
92.H	-7.872045	-1.295102	-3.319456
93.H	7.732195	0.247938	-3.413349
94.H	2.244887	-3.153156	-3.721722
95.H	-1.238270	-0.876384	-3.557723
96.H	-4.203211	-1.653706	-3.438669
97.H	-9.183602	-2.267593	-2.631518
98.H	0.529265	-3.525692	-3.451233
99.H	-7.554208	-2.956325	-2.778630
100.H	4.274708	-8.134059	-2.620671
101.H	-0.441520	2.515430	-2.998228
102.H	3.919715	2.879867	-2.952639
103.H	-5.124273	1.956814	-2.817089
104.H	0.456769	-1.196192	-3.091589
105.H	-6.695711	1.149074	-2.717233
106.H	2.709841	-7.309262	-2.527609
107.H	5.890921	1.753933	-2.600491
108.H	-3.283479	4.330322	-2.467993
109.H	-0.334358	0.312056	-2.582521
110.H	5.469059	-6.059346	-1.996669
111.H	6.360688	-4.083562	-2.034978
112.H	7.558536	-0.711162	-1.921734
113.H	2.903320	4.021342	-2.047834
114.H	-5.285505	5.692269	-1.939214
115.H	-5.349487	-3.362196	-2.103185
116.H	0.361404	3.860045	-2.154486
117.H	1.809033	1.782947	-2.120087
118.H	-9.729581	-0.517003	-0.910454
119.H	7.270317	1.713229	-1.521511
120.H	-8.461058	0.560982	-1.518622
121.H	-4.021206	0.078031	-1.618168
122.H	0.916194	-6.102367	-1.656626
123.H	3.678926	-7.541187	-1.057142
124.H	-3.683388	3.035740	-1.321607
125.H	-5.906888	1.730092	-1.238935
126.H	6.587052	-2.583342	-1.157322
127.H	-3.108715	-2.473120	-1.686457
128.H	-2.113730	3.848548	-1.224365
129.H	-0.220617	-4.787568	-1.367263
130.H	1.697380	-3.139069	-1.255728
131.H	5.595988	3.852031	-0.931840
132.H	-5.897000	4.489996	-0.788460
133.H	-8.085402	-2.287015	-0.416550
134.H	-3.206936	5.976142	-0.543119
135.H	-3.191320	-4.089550	-0.938542
136.H	-5.686785	6.179133	-0.280430
137.H	-1.940169	0.788042	-0.786311

138.H	-6.851540	-4.189995	-0.274641
139.H	-5.334724	-5.109515	-0.297679
140.H	8.489334	-0.127080	-0.189046
141.H	-8.494345	0.119161	0.193410
142.H	3.324566	2.621351	-0.078340
143.H	6.854115	4.189774	0.274810
144.H	0.949324	-5.252212	-0.105777
145.H	-0.957216	5.269152	0.114170
146.H	5.336766	5.108096	0.293685
147.H	5.681941	-6.174034	0.301311
148.H	-3.319829	-2.628732	0.078391
149.H	8.087726	2.283839	0.413820
150.H	3.200394	-5.967846	0.554368
151.H	5.890506	-4.482470	0.802836
152.H	3.192564	4.088037	0.929994
153.H	-5.591786	-3.856279	0.931032
154.H	9.730648	0.515677	0.904135
155.H	-3.672976	7.543383	1.076239
156.H	1.920055	-0.787081	0.777526
157.H	8.466463	-0.559858	1.525407
158.H	-6.588713	2.585833	1.164651
159.H	0.221347	4.776441	1.356581
160.H	-7.269456	-1.713452	1.521144
161.H	-1.699928	3.139262	1.251278
162.H	5.909364	-1.728901	1.240277
163.H	2.109258	-3.833585	1.224315
164.H	-0.902678	6.094807	1.677643
165.H	3.681313	-3.026539	1.327374
166.H	3.110133	2.474324	1.685690
167.H	4.022165	-0.077605	1.617665
168.H	5.274797	-5.680013	1.956405
169.H	-7.557572	0.710144	1.925233
170.H	-5.465639	6.060617	2.011860
171.H	-6.361066	4.084117	2.045558
172.H	5.350751	3.363700	2.103145
173.H	-2.894171	-4.028060	2.046545
174.H	-0.356925	-3.866230	2.155180
175.H	-4.266843	8.131601	2.642220
176.H	-2.703589	7.304134	2.545207
177.H	-1.801736	-1.788887	2.118046
178.H	9.187828	2.269616	2.627565
179.H	-5.889404	-1.755835	2.599457
180.H	3.271779	-4.317798	2.474168
181.H	6.696469	-1.146652	2.718970
182.H	7.558721	2.958897	2.775458
183.H	0.334311	-0.311572	2.574118
184.H	5.125587	-1.955737	2.818043
185.H	-3.912637	-2.888177	2.951496
186.H	7.877072	1.298632	3.319166
187.H	-7.730433	-0.250999	3.415608
188.H	-0.464242	1.193775	3.078987
189.H	0.448673	-2.520749	2.997233
190.H	-7.393300	2.462385	3.551195
191.H	4.202061	1.654511	3.437404
192.H	-0.521574	3.517995	3.445137
193.H	-2.234873	3.136178	3.717529
194.H	-1.659796	4.807358	3.849942
195.H	1.232313	0.883086	3.544350
196.H	-2.937898	-4.094865	3.814338
197.H	-0.320400	-3.818591	3.915957
198.H	5.698779	0.904788	4.011686
199.H	-5.112614	4.797497	4.168815

200.H	-5.732516	2.825386	4.085790
201.H	-5.146251	6.556848	4.399893
202.H	4.174730	0.007178	4.098737
203.H	-3.604712	5.684935	4.433077
204.H	-5.661270	-0.677026	4.579043
205.H	-3.888672	1.798536	4.804844
206.H	0.462457	-1.042628	4.962921
207.H	-6.161749	0.839206	5.373322
208.H	-1.014604	1.578567	5.644301
209.H	-3.898500	-3.418798	5.721292
210.H	-3.817935	0.408160	5.866742
211.H	0.707730	1.276294	5.946062
212.H	-4.617759	-1.832049	6.035737
213.H	-1.281752	-3.744503	6.090425
214.H	-0.318667	-2.372141	6.676274
215.H	-0.520669	0.329045	6.805674
216.H	-2.397162	-0.978515	6.845139
217.H	-3.977120	-2.796621	7.377849
218.H	-1.531895	-3.124298	7.730444
219.K	-0.529942	-1.686139	-0.407508
220.K	0.510314	1.690973	0.384577
221.N	3.183562	-0.018218	-3.841022
222.N	5.946126	-0.919471	-3.242241
223.N	4.537737	-2.975148	-1.674324
224.N	5.529945	0.660618	-0.782808
225.N	2.018447	-0.549194	-0.224594
226.N	-2.025992	0.550064	0.216250
227.N	-5.529701	-0.659622	0.783172
228.N	-4.538788	2.976298	1.679453
229.N	-5.944529	0.916825	3.245007
230.N	-3.181268	0.012704	3.837795
231.Si	1.981307	1.098590	-4.440786
232.Si	3.696357	-4.484036	-1.381488
233.Si	-5.876741	-1.287439	-0.819831
234.Si	5.878377	1.287749	0.820295
235.Si	-3.696388	4.485229	1.389580
236.Si	-1.976964	-1.102359	4.437381
237.Th	3.723187	-0.711517	-1.578447
238.Th	-3.724500	0.713067	1.578749

Energy: −1277.25837385 eV

Supplementary Table 5 - Complex 4Rb

1.C	3.961981	-0.154914	-6.309735
2.C	4.735510	0.366416	-5.080324
3.C	6.082603	-0.371390	-4.962614
4.C	-4.393744	-3.946972	-4.795413
5.C	-6.665161	3.663797	-4.419038
6.C	-4.418146	0.366058	-4.520610
7.C	2.533410	2.917414	-4.508885
8.C	-3.138207	2.528473	-4.392956
9.C	-7.719267	1.443741	-3.849584
10.C	-2.306041	-2.802328	-3.959016
11.C	-3.226079	-4.023156	-3.792006
12.C	2.120118	1.524758	-3.995132
13.C	-6.881007	2.616239	-3.306692
14.C	1.771308	-1.809888	-3.723866
15.C	-3.893826	1.462832	-3.574226
16.C	4.070068	-2.354131	-2.842125
17.C	2.976042	-1.273904	-2.930835
18.C	-4.046897	-7.101891	-2.499189
19.C	1.097709	1.672117	-2.857103
20.C	5.638280	1.994336	-2.335980

21.C	-4.847210	-5.892969	-1.972474
22.C	-6.229295	-2.882400	-1.717298
23.C	6.918433	1.466523	-1.677130
24.C	-4.600799	3.817368	-1.658315
25.C	-1.096430	-5.128262	-1.388159
26.C	-5.623461	4.509304	-0.736880
27.C	-5.536307	-6.257679	-0.644379
28.C	-2.274742	-4.295376	-0.851819
29.C	-3.257001	3.669294	-0.924559
30.C	-7.076128	-2.690621	-0.455648
31.C	-7.556383	-0.277985	-0.388581
32.C	2.565158	4.695002	-0.614876
33.C	-6.763414	1.033092	-0.368294
34.C	7.555771	0.281158	0.388967
35.C	6.764832	-1.031069	0.370709
36.C	7.073630	2.693380	0.452080
37.C	5.527633	6.258796	0.630586
38.C	5.633442	-4.506374	0.738219
39.C	-2.572223	-4.685168	0.607622
40.C	3.264451	-3.673758	0.927922
41.C	2.269536	4.289970	0.840721
42.C	1.082124	5.107095	1.382473
43.C	-6.922270	-1.467373	1.676009
44.C	4.609165	-3.818470	1.660850
45.C	6.226614	2.887972	1.713108
46.C	4.836051	5.897398	1.958097
47.C	4.029858	7.105537	2.477877
48.C	-5.643305	-1.996412	2.335793
49.C	-4.067814	2.351959	2.841103
50.C	-1.100416	-1.676841	2.857299
51.C	-2.976132	1.269262	2.928766
52.C	6.885344	-2.611003	3.310636
53.C	3.896057	-1.464943	3.576868
54.C	7.722764	-1.435808	3.849324
55.C	3.222444	4.028460	3.784336
56.C	-1.769838	1.803146	3.722423
57.C	-2.123398	-1.529320	3.994847
58.C	2.312624	2.801106	3.958413
59.C	6.670178	-3.654575	4.426838
60.C	3.143273	-2.532184	4.396518
61.C	-2.538324	-2.921553	4.508493
62.C	4.417968	-0.366464	4.522840
63.C	4.391863	3.965564	4.786663
64.C	-6.084036	0.373295	4.959633
65.C	-4.738701	-0.367547	5.078979
66.C	-3.965949	0.151584	6.309758
67.H	4.574627	-0.063399	-7.221769
68.H	3.026577	0.393692	-6.488004
69.H	3.707932	-1.220626	-6.203314
70.H	6.682928	-0.228679	-5.876513
71.H	-4.022148	-3.822606	-5.826081
72.H	-7.631533	4.004809	-4.826068
73.H	4.972426	1.427303	-5.288032
74.H	-6.094850	3.243849	-5.261410
75.H	3.176518	2.858967	-5.398019
76.H	-5.095924	0.788180	-5.277047
77.H	-5.017863	-4.851827	-4.780327
78.H	-3.803653	3.037668	-5.105304
79.H	-3.590965	-0.115824	-5.065953
80.H	-2.000674	-2.674753	-5.011009
81.H	5.943427	-1.453892	-4.835526
82.H	-8.690442	1.802886	-4.229410

83.H	-2.332186	2.063529	-4.984750
84.H	-7.215173	0.933828	-4.682356
85.H	-5.047158	-3.087475	-4.581817
86.H	2.036157	-2.002383	-4.773680
87.H	1.650825	3.518670	-4.783506
88.H	1.618369	0.993122	-4.824134
89.H	6.685128	-0.018158	-4.115710
90.H	-6.126632	4.554116	-4.065464
91.H	-2.634246	-4.924191	-4.038204
92.H	-4.968681	-0.421314	-3.985494
93.H	4.399974	-2.660338	-3.845278
94.H	-2.813260	-1.876900	-3.649997
95.H	3.082628	3.478134	-3.737104
96.H	-2.680182	3.305869	-3.765944
97.H	-3.591008	-6.912568	-3.480844
98.H	0.919952	-1.115204	-3.733525
99.H	-7.924307	0.686541	-3.081897
100.H	5.857721	2.141759	-3.408320
101.H	-1.387613	-2.885385	-3.359703
102.H	1.417147	-2.774360	-3.317177
103.H	-7.494539	3.109134	-2.529526
104.H	0.238138	2.289794	-3.171149
105.H	-5.652603	-5.710293	-2.708811
106.H	-3.157662	0.990713	-2.891470
107.H	-4.699245	-7.984465	-2.604700
108.H	3.701066	-3.262439	-2.337915
109.H	-6.486833	-2.082531	-2.440740
110.H	-4.429801	4.482180	-2.524683
111.H	-6.568345	-3.818018	-2.196991
112.H	0.703304	0.698536	-2.525950
113.H	-0.787843	-4.832050	-2.400396
114.H	7.099233	0.447563	-2.046008
115.H	4.954604	-2.002850	-2.292859
116.H	7.798848	2.084396	-1.946089
117.H	-3.239611	-7.384499	-1.806109
118.H	5.438309	3.013080	-1.946853
119.H	2.637956	-1.062502	-1.892564
120.H	1.568362	2.154424	-1.985523
121.H	-1.512336	-0.232621	-1.613492
122.H	-7.767777	-0.536698	-1.434855
123.H	-6.567913	4.728549	-1.254505
124.H	-1.347825	-6.197960	-1.428135
125.H	-2.465255	3.308584	-1.599267
126.H	1.696807	4.519441	-1.272806
127.H	-6.207742	-7.122449	-0.776890
128.H	-8.159508	-2.766208	-0.677584
129.H	-7.437602	1.824732	-0.742290
130.H	3.417759	4.143450	-1.036177
131.H	-1.975972	-3.223930	-0.860160
132.H	-0.212093	-5.050856	-0.728735
133.H	2.799034	5.767311	-0.686025
134.H	6.567831	-1.307016	-0.685661
135.H	-6.137026	-5.430635	-0.244122
136.H	-5.230778	5.467282	-0.356311
137.H	-2.926698	4.633104	-0.499946
138.H	8.527590	0.174091	-0.133405
139.H	6.822126	3.491938	-0.259263
140.H	4.798225	6.531249	-0.145674
141.H	-8.528041	-0.170261	0.133743
142.H	-5.860341	3.885854	0.138711
143.H	5.867411	-3.881772	-0.137307
144.H	-4.805286	-6.533489	0.129184

145.H	-6.824533	-3.490673	0.253902
146.H	-3.353200	2.952085	-0.092417
147.H	6.130266	5.431282	0.234260
148.H	3.356325	-2.951426	0.100614
149.H	5.243690	-5.465563	0.357504
150.H	6.197752	7.124712	0.761847
151.H	8.157051	2.769704	0.673964
152.H	2.937323	-4.637528	0.499945
153.H	-6.566121	1.307108	0.688456
154.H	7.440248	-1.821038	0.745799
155.H	-2.820650	-5.753285	0.688769
156.H	0.200338	5.028686	0.719353
157.H	1.980027	3.215931	0.840556
158.H	-3.415350	-4.117923	1.027210
159.H	6.579097	-4.722681	1.254892
160.H	7.767075	0.541667	1.434853
161.H	-1.698920	-4.516422	1.260657
162.H	1.323924	6.178713	1.432611
163.H	2.471522	-3.320418	1.605437
164.H	3.223247	7.382050	1.781651
165.H	-7.803336	-2.085325	1.943171
166.H	-7.103446	-0.448853	2.045999
167.H	-5.443925	-3.015364	1.946996
168.H	4.678540	7.991000	2.581255
169.H	6.564064	3.825603	2.189954
170.H	1.514596	0.230427	1.626106
171.H	-2.637810	1.057055	1.890662
172.H	-1.572629	-2.156778	1.984401
173.H	-4.953321	2.002975	2.291953
174.H	6.484906	2.090718	2.439009
175.H	4.440784	-4.484423	2.526783
176.H	5.639952	5.720251	2.697344
177.H	0.774024	4.799185	2.391260
178.H	7.499356	-3.106185	2.535317
179.H	-3.697022	3.259759	2.337507
180.H	-0.706587	-0.703938	2.525158
181.H	7.929635	-0.682803	3.078071
182.H	3.158755	-0.994849	2.894387
183.H	3.572492	6.918456	3.459244
184.H	-0.241297	-2.295471	3.170407
185.H	-5.863506	-2.143397	3.408029
186.H	-1.419527	2.770121	3.319560
187.H	1.393364	2.872594	3.358952
188.H	-4.396827	2.657967	3.844713
189.H	-3.087311	-3.481985	3.736365
190.H	-0.917206	1.110402	3.727311
191.H	2.688160	-3.311640	3.770141
192.H	2.827577	1.878355	3.654450
193.H	6.131142	-4.546056	4.076839
194.H	4.966209	0.422167	3.987311
195.H	8.692886	-1.793243	4.233048
196.H	2.624006	4.926145	4.027226
197.H	-6.685350	0.023120	4.110729
198.H	7.216871	-0.921356	4.678244
199.H	5.051547	3.110070	4.576002
200.H	5.009222	4.874953	4.767121
201.H	-5.942268	1.455748	4.835078
202.H	-1.656623	-3.523413	4.784241
203.H	7.636909	-3.994467	4.834083
204.H	-2.033210	1.989841	4.773736
205.H	-1.621864	-0.998025	4.824320
206.H	3.810048	-3.038426	5.109691

207.H	2.335614	-2.069095	4.987303
208.H	6.100967	-3.231576	5.268243
209.H	5.097252	-0.786588	5.279102
210.H	3.589727	0.113218	5.068371
211.H	2.008643	2.676873	5.011253
212.H	-3.182325	-2.862107	5.396933
213.H	-4.978211	-1.427999	5.285744
214.H	4.022464	3.842665	5.818272
215.H	-6.686783	0.230114	5.871994
216.H	-3.709237	1.216739	6.204046
217.H	-3.032051	-0.399327	6.489191
218.H	-4.580139	0.061180	7.220754
219.N	4.494073	1.085071	-2.098731
220.N	-4.782123	-2.885582	-1.401350
221.N	-5.506385	0.927575	-1.147401
222.N	-2.031503	-0.384214	-0.730530
223.N	6.767698	1.393538	-0.200427
224.N	-6.769870	-1.392261	0.199622
225.N	2.032415	0.383201	0.742447
226.N	5.507618	-0.926436	1.149856
227.N	4.779598	2.888010	1.396532
228.N	-4.498054	-1.088322	2.099139
229.Rb	0.454940	-1.900579	-0.196202
230.Rb	-0.443014	1.899415	0.203574
231.Si	3.646977	0.451215	-3.482385
232.Si	-5.245798	2.139708	-2.383841
233.Si	-3.841848	-4.239473	-1.976043
234.Si	3.835536	4.241284	1.967058
235.Si	5.249530	-2.138809	2.386788
236.Si	-3.649334	-0.454306	3.481716
237.Th	-4.102162	-0.841069	-0.292125
238.Th	4.101401	0.840801	0.294613

Energy: −1277.01124739 eV

Supplementary Table 6 - Complex 4Cs

1.C	2.914609	2.861964	-6.306386
2.C	5.136349	3.158385	-5.143479
3.C	3.962098	2.192929	-5.391563
4.C	-6.676039	-3.568239	-4.668000
5.C	2.475838	-0.866888	-5.549353
6.C	-3.290669	-1.953769	-4.588862
7.C	1.873098	0.122811	-4.534284
8.C	-4.774851	-0.040199	-3.907966
9.C	-7.831944	-1.701277	-3.422724
10.C	-6.852215	-2.883007	-3.297259
11.C	5.580764	0.077662	-3.396438
12.C	-4.047802	-1.304729	-3.412304
13.C	-5.764813	3.959158	-2.902086
14.C	0.794531	2.975300	-3.205726
15.C	6.829243	0.659861	-2.724927
16.C	1.160851	-0.651345	-3.413633
17.C	-3.371435	3.245880	-2.546267
18.C	2.210301	2.619745	-2.719864
19.C	-4.264977	-4.200332	-2.303297
20.C	6.848905	-4.795901	-1.790874
21.C	3.471782	-3.771145	-2.362327
22.C	-4.529723	4.090135	-1.989580
23.C	2.993839	3.909855	-2.412895
24.C	-5.076711	-5.299392	-1.592045
25.C	-2.880514	-4.066422	-1.649503
26.C	7.522738	-1.047613	-1.090815
27.C	6.271932	-4.968137	-0.373180

28.C	3.288782	-3.836574	-0.835264
29.C	7.529803	1.333728	-0.459925
30.C	5.832888	-6.430598	-0.158655
31.C	2.407056	-5.044369	-0.465332
32.C	-6.933996	1.750819	-0.135283
33.C	-6.492448	-2.386290	0.000017
34.C	-5.843658	6.418532	0.167530
35.C	6.504017	2.375298	-0.000413
36.C	6.924375	-1.765998	0.122707
37.C	-7.525318	-1.350393	0.455207
38.C	-6.285909	4.955446	0.377256
39.C	-2.419482	5.018701	0.476396
40.C	-3.305821	3.816660	0.849499
41.C	-7.532499	1.032364	1.077867
42.C	5.102194	5.297057	1.606063
43.C	-6.865420	4.781705	1.793445
44.C	2.897413	4.082653	1.667611
45.C	4.527099	-4.094249	1.998081
46.C	-2.993385	-3.903728	2.439640
47.C	-3.490654	3.753215	2.376802
48.C	4.286313	4.198962	2.314841
49.C	-6.831086	-0.664900	2.718065
50.C	3.370241	-3.248066	2.554954
51.C	-2.209419	-2.610739	2.730336
52.C	5.764916	-3.961208	2.906455
53.C	7.845160	1.686420	3.418215
54.C	6.867434	2.870114	3.297037
55.C	-1.170530	0.668884	3.399088
56.C	-5.585967	-0.074541	3.387609
57.C	-0.786872	-2.951462	3.208942
58.C	4.057472	1.301890	3.413357
59.C	4.781876	0.035657	3.908009
60.C	-1.878088	-0.101388	4.525066
61.C	6.694885	3.552948	4.669371
62.C	3.303021	1.952086	4.590939
63.C	-2.477149	0.892052	5.538844
64.C	-5.133428	-3.139675	5.158836
65.C	-3.962044	-2.168625	5.397787
66.C	-2.911891	-2.827636	6.316698
67.H	3.377676	3.194297	-7.250486
68.H	5.596336	3.461382	-6.099227
69.H	2.087312	2.187863	-6.568164
70.H	4.371229	1.336861	-5.960383
71.H	2.899223	-0.357536	-6.426073
72.H	2.479236	3.756058	-5.834318
73.H	-7.651356	-3.878563	-5.078736
74.H	-6.223608	-2.885062	-5.402902
75.H	-3.980922	-2.292726	-5.375845
76.H	5.925175	2.710596	-4.526619
77.H	1.712236	-1.571560	-5.919410
78.H	4.808536	4.077926	-4.638843
79.H	-2.605316	-1.227579	-5.056963
80.H	-6.043064	-4.464696	-4.615265
81.H	3.277610	-1.468003	-5.092825
82.H	-5.483742	-0.280773	-4.713934
83.H	1.110488	0.723203	-5.062939
84.H	5.655720	0.297787	-4.476663
85.H	-8.827667	-2.049170	-3.744369
86.H	-2.692690	-2.824368	-4.286862
87.H	-4.059944	0.690806	-4.318228
88.H	-7.489347	-0.969754	-4.168190
89.H	-5.514392	4.210887	-3.946379

90.H	0.825826	3.515213	-4.163412
91.H	-3.194775	3.465223	-3.612550
92.H	7.755678	0.346440	-3.246830
93.H	-4.107205	-4.526429	-3.347324
94.H	5.629453	-1.028005	-3.329749
95.H	0.153327	2.095764	-3.355429
96.H	-7.336937	-3.626340	-2.636512
97.H	-6.151681	2.928813	-2.901631
98.H	0.387074	-1.324435	-3.822094
99.H	-5.342115	0.460613	-3.110432
100.H	3.084266	4.537156	-3.311323
101.H	6.768147	1.755611	-2.779191
102.H	-6.585329	4.622950	-2.593679
103.H	6.104356	-5.042915	-2.562902
104.H	-7.957403	-1.161407	-2.474618
105.H	3.942131	-4.688889	-2.744163
106.H	-3.290996	-0.979134	-2.669253
107.H	7.708699	-5.468331	-1.946971
108.H	-6.050535	-5.474832	-2.070858
109.H	-3.583497	2.169586	-2.462324
110.H	2.503184	-3.674427	-2.882217
111.H	1.887654	-1.263373	-2.856253
112.H	0.280823	3.650230	-2.497294
113.H	4.101569	-2.924383	-2.669685
114.H	7.188959	-3.771834	-1.984141
115.H	-4.212463	5.149138	-2.009562
116.H	7.317589	-1.652622	-1.984723
117.H	-2.425742	3.430958	-2.015786
118.H	0.679595	0.021285	-2.686852
119.H	-2.239907	-3.370041	-2.212496
120.H	-4.533247	-6.259641	-1.597069
121.H	4.008683	3.702194	-2.046838
122.H	2.121051	2.049506	-1.768888
123.H	-2.360482	-5.038633	-1.598695
124.H	2.479803	4.521955	-1.652033
125.H	8.622621	-0.942180	-0.999736
126.H	8.365583	1.807366	-1.013075
127.H	-5.483786	6.609037	-0.853538
128.H	-7.121464	1.134609	-1.037670
129.H	-1.765091	0.299170	-1.220500
130.H	6.672846	-7.120451	-0.344946
131.H	-5.265022	-5.041069	-0.539344
132.H	5.026861	-6.716600	-0.849711
133.H	1.442672	-5.021806	-1.004630
134.H	6.086393	2.875150	-0.898270
135.H	-7.046199	-3.184439	-0.527028
136.H	2.888964	-5.991198	-0.748150
137.H	-7.951348	-0.872151	-0.436055
138.H	-2.978057	-3.682470	-0.620851
139.H	-2.210672	5.076938	-0.600608
140.H	-7.524088	2.671049	-0.293850
141.H	-7.116864	4.779320	-0.331053
142.H	2.757614	-2.911415	-0.523287
143.H	7.105620	-4.789130	0.332148
144.H	7.512596	-2.688124	0.277390
145.H	-6.682516	7.108922	0.353696
146.H	7.952855	0.849665	0.429874
147.H	-5.038649	6.700710	0.862225
148.H	7.064153	3.168107	0.527892
149.H	5.476816	-6.619645	0.864769
150.H	-2.779440	2.888812	0.535644
151.H	2.186621	-5.104995	0.609755

152.H	-8.357973	-1.827715	1.010021
153.H	5.285814	5.042632	0.551701
154.H	-2.892497	5.968683	0.763898
155.H	-8.631497	0.919912	0.983604
156.H	-6.072231	-2.880600	0.899593
157.H	2.985180	3.716521	0.632408
158.H	7.117432	-1.152288	1.025803
159.H	-1.450607	4.989325	1.007122
160.H	4.564187	6.260211	1.617489
161.H	1.797323	-0.287542	1.229538
162.H	2.387115	5.060299	1.632081
163.H	-7.334063	1.641008	1.970829
164.H	-2.494766	-4.512774	1.666517
165.H	-7.723788	5.455839	1.949542
166.H	-7.212436	3.756204	1.980899
167.H	-4.016910	-3.699657	2.095559
168.H	-2.128887	-2.051458	1.772306
169.H	6.078447	5.464670	2.082780
170.H	4.209627	-5.152975	2.021264
171.H	7.969056	1.149172	2.468609
172.H	2.251018	3.385481	2.222892
173.H	2.425261	-3.429900	2.021562
174.H	-6.123510	5.020609	2.567097
175.H	6.584431	-4.625670	2.596928
176.H	-4.134569	2.916381	2.682800
177.H	-0.712601	-0.004203	2.657633
178.H	7.352909	3.614030	2.637337
179.H	-3.948004	4.678325	2.756642
180.H	-6.764873	-1.760177	2.776647
181.H	-2.525557	3.640627	2.900406
182.H	-0.281272	-3.645224	2.513125
183.H	3.585405	-2.172858	2.474374
184.H	6.151825	-2.930915	2.902544
185.H	-1.894323	1.299446	2.858349
186.H	3.298984	0.978944	2.670627
187.H	-7.759522	-0.353824	3.237946
188.H	-3.062451	-4.533039	3.338751
189.H	5.347418	-0.466065	3.109784
190.H	-5.639119	1.030629	3.315880
191.H	4.136471	4.519749	3.361462
192.H	8.841510	2.031851	3.740323
193.H	-0.376294	1.322737	3.799940
194.H	-0.150419	-2.063056	3.322165
195.H	7.501824	0.953447	4.162153
196.H	3.190865	-3.470719	3.620252
197.H	5.517874	-4.210692	3.952179
198.H	-0.802119	-3.461297	4.183163
199.H	-5.660063	-0.290119	4.468973
200.H	4.065985	-0.694060	4.318953
201.H	5.491929	0.274811	4.713222
202.H	6.065944	4.451980	4.619489
203.H	2.706597	2.824414	4.289785
204.H	-3.283338	1.488140	5.084689
205.H	-1.112864	-0.699284	5.052585
206.H	-5.925405	-2.699174	4.540752
207.H	7.672053	3.858241	5.080246
208.H	-4.803230	-4.061742	4.659991
209.H	6.240276	2.869857	5.403372
210.H	2.616736	1.227544	5.059619
211.H	3.994990	2.289512	5.377011
212.H	-1.712704	1.601305	5.899922
213.H	-2.474393	-3.724111	5.851518

214.H	-2.892516	0.386245	6.421233
215.H	-4.372938	-1.309318	5.960442
216.H	-5.590156	-3.437757	6.117749
217.H	-2.086016	-2.149175	6.572709
218.H	-3.373213	-3.153673	7.263950
219.Cs	-0.055262	-2.090726	-0.364065
220.Cs	0.046197	2.096659	0.367166
221.N	4.342329	0.608288	-2.782169
222.N	6.888624	0.281140	-1.289068
223.N	-5.425565	-1.774546	-0.829954
224.N	-5.487946	2.015187	0.052834
225.N	-2.237429	0.101563	-0.321742
226.N	5.476490	-2.026581	-0.060049
227.N	2.242604	-0.104299	0.314126
228.N	5.433877	1.768964	0.829446
229.N	-6.890406	-0.291538	1.281009
230.N	-4.345385	-0.603054	2.775984
231.Si	3.176550	1.367943	-3.828345
232.Si	-5.177545	-2.494211	-2.407181
233.Si	-4.947020	3.661899	-0.153787
234.Si	4.934830	-3.672357	0.159572
235.Si	5.190279	2.488162	2.407737
236.Si	-3.179335	-1.352622	3.827928
237.Th	4.272322	0.053686	-0.411503
238.Th	-4.269247	-0.053670	0.403645

Energy: −1277.13691861 eV

Supplementary Table 7 - Complex 5

1.C	3.280542	-1.353042	-5.502713
2.C	1.062980	-0.159439	-5.274882
3.C	1.975158	-1.251699	-4.687298
4.C	4.815457	0.316280	-2.643139
5.C	2.600995	-4.030739	-2.717823
6.C	2.732502	1.741412	-2.669938
7.C	-1.648492	-1.187268	-2.552200
8.C	-0.323849	-1.931905	-2.390875
9.C	3.275426	-2.710882	-2.295472
10.C	3.325360	0.382763	-2.252964
11.C	-0.125683	5.037729	-1.988085
12.C	-1.876135	1.868176	-1.688507
13.C	-2.836303	0.688672	-1.506936
14.C	3.602049	-2.739516	-0.792130
15.C	0.109577	4.895467	-0.471654
16.C	-2.806180	-1.523978	-0.399071
17.C	1.589780	4.588641	-0.185289
18.C	-2.867384	4.396423	0.187063
19.C	-2.953146	5.891407	0.555886
20.C	-0.066501	-4.611877	0.832131
21.C	-3.969643	3.609275	0.921615
22.C	-2.551335	-1.220409	1.079745
23.C	-2.308260	-4.788948	1.973828
24.C	-0.854768	-4.294295	2.114315
25.C	-0.508823	3.359065	2.138336
26.C	-0.413003	4.670044	2.945999
27.C	-1.299948	2.310662	2.937429
28.C	1.199130	-2.149149	3.057940
29.C	1.944796	-3.381203	3.613893
30.C	1.456179	-0.938658	3.978516
31.C	-1.798753	-2.276451	4.223338
32.C	-2.079816	-0.835306	4.682975
33.C	-1.331298	-3.144099	5.409816
34.H	3.061208	-1.561448	-6.562911

35.H	0.882310	-0.336464	-6.348562
36.H	3.850681	-0.413515	-5.471882
37.H	3.941605	-2.153963	-5.141474
38.H	0.085079	-0.123127	-4.775749
39.H	1.450018	-2.215315	-4.826200
40.H	1.512174	0.839937	-5.182093
41.H	4.956907	0.422536	-3.728821
42.H	2.410848	-4.078859	-3.799657
43.H	2.821809	1.907276	-3.753749
44.H	-0.087809	-2.384240	-3.370140
45.H	-1.503978	-0.379068	-3.281704
46.H	4.233480	-2.641107	-2.842961
47.H	-2.444639	-1.852158	-2.940516
48.H	3.233612	-4.895991	-2.458246
49.H	5.377321	1.134878	-2.164010
50.H	5.289828	-0.626848	-2.339810
51.H	0.527321	5.812991	-2.420461
52.H	-1.163539	5.312649	-2.227650
53.H	-1.247630	1.687633	-2.582087
54.H	0.097964	4.095195	-2.511249
55.H	-3.511306	0.570164	-2.377558
56.H	1.663044	1.835312	-2.419376
57.H	1.638568	-4.171601	-2.202687
58.H	3.263385	2.570400	-2.174323
59.H	-2.479573	2.756236	-1.939946
60.H	-0.473270	-2.790483	-1.708319
61.H	3.282494	0.341645	-1.146491
62.H	-3.098208	4.336353	-0.893114
63.H	-3.459634	0.880032	-0.623239
64.H	4.197170	-3.629938	-0.529469
65.H	4.167535	-1.854627	-0.467080
66.H	-3.888538	-1.509327	-0.636297
67.H	-2.429567	-2.532780	-0.616646
68.H	2.249049	5.323606	-0.677165
69.H	1.873149	3.597944	-0.571938
70.H	2.678004	-2.779818	-0.192903
71.H	-0.120265	5.870397	-0.004856
72.H	-3.969937	6.275639	0.371258
73.H	-2.258934	6.508679	-0.030588
74.H	-0.476605	-4.060636	-0.028007
75.H	-4.971220	3.959668	0.622535
76.H	-0.118710	-5.685568	0.587231
77.H	-3.920811	2.529682	0.721495
78.H	0.997032	-4.344550	0.909544
79.H	1.819837	4.609701	0.889671
80.H	-2.840059	-4.267000	1.163489
81.H	-2.332478	-5.863847	1.728360
82.H	-2.732271	6.064087	1.619090
83.H	-2.955313	-0.215076	1.312002
84.H	2.607269	1.750375	0.941606
85.H	2.720258	0.368949	1.810132
86.H	-3.895456	3.743379	2.010830
87.H	-3.166830	-1.918865	1.673956
88.H	0.521723	2.966769	2.032951
89.H	1.663402	-1.925857	2.073506
90.H	0.163057	5.447926	2.427106
91.H	-1.340220	1.326540	2.441087
92.H	-2.891551	-4.653639	2.895340
93.H	-0.396480	-4.876154	2.935725
94.H	1.884477	-4.251469	2.949639
95.H	-1.412042	5.083084	3.155641
96.H	-2.339318	2.622137	3.106134

97.H	0.989995	-0.013886	3.609223
98.H	0.070909	4.492673	3.919673
99.H	3.011957	-3.150833	3.762460
100.H	-0.843969	2.147619	3.927362
101.H	-2.483818	-0.217215	3.872233
102.H	-2.768150	-2.693602	3.891532
103.H	2.538050	-0.744876	4.077037
104.H	1.540653	-3.682608	4.592888
105.H	-1.171106	-0.338361	5.052539
106.H	1.074374	-1.127311	4.991136
107.H	-1.181844	-4.197955	5.130691
108.H	-2.812611	-0.827095	5.507214
109.H	-0.383785	-2.775747	5.830087
110.H	-2.073198	-3.122854	6.225753
111.N	0.748307	-1.029902	-1.902019
112.N	-2.070024	-0.565063	-1.268757
113.N	-1.044916	2.055985	-0.475099
114.N	2.149632	0.846167	1.104906
115.N	-1.105997	-1.303199	1.411601
116.Si	2.276684	-1.134145	-2.780906
117.Si	-1.097547	3.625562	0.329929
118.Si	-0.667308	-2.452280	2.669213
119.Th	0.201551	0.140462	0.081719

Energy: −641.10299325 eV

Supplementary Table 8 - Complex 11 (all-electron)

1.H	-3.454845	-2.300756	-2.806089
2.H	-4.548963	-0.905131	-2.693262
3.H	-3.603109	1.108658	-2.260032
4.H	-4.515499	2.637896	-2.216930
5.C	-4.188026	-1.793557	-2.153961
6.H	-5.044679	-2.480002	-2.040434
7.C	-4.125178	1.809911	-1.594351
8.H	-5.955411	0.680963	-1.526245
9.H	-3.364788	2.230927	-0.922423
10.H	-2.653375	-0.827358	-0.970320
11.H	-7.092963	-2.018229	-0.891793
12.C	-5.253951	1.133026	-0.798172
13.H	-6.418213	2.995378	-0.656633
14.H	-7.679265	-0.380677	-0.548434
15.C	-3.555607	-1.450301	-0.791954
16.H	-2.260608	-3.207187	-0.748527
17.H	0.730282	4.863193	-0.574209
18.H	-1.376658	3.431997	-0.536102
19.C	-7.395096	-1.335007	-0.081576
20.C	-6.031481	2.194466	0.002021
21.C	-3.036248	-2.734667	-0.124122
22.H	-8.307957	-1.763202	0.377349
23.H	-3.840220	-3.472317	0.032540
24.H	0.107119	-5.182554	0.192560
25.Si	-4.616896	-0.319507	0.340105
26.H	-6.890904	1.769444	0.544838
27.C	0.701440	4.930474	0.528046
28.H	-5.378217	2.674021	0.746656
29.H	-0.101087	-3.442222	0.536373
30.C	-1.418477	3.579374	0.555635
31.H	1.505556	-4.154825	0.581783
32.N	-0.049820	-0.170294	0.382652
33.H	0.162606	5.860352	0.778410
34.H	-2.018843	4.482940	0.752324
35.H	0.536694	2.786753	0.784767
36.C	0.452824	-4.347226	0.830966

37.H	-2.574490	-2.534637	0.854078
38.H	-5.806247	-3.303062	1.005953
39.H	1.737821	5.047636	0.878348
40.C	-6.274473	-1.162662	0.959435
41.H	-1.973451	2.724312	0.970214
42.C	0.003139	3.696578	1.130869
43.C	-6.041354	-2.487753	1.707848
44.H	2.864533	3.150783	1.548323
45.H	-6.650239	-0.439620	1.710362
46.H	-1.333267	-6.132217	1.889383
47.N	-3.655625	0.160898	1.673943
48.H	1.746654	-1.860954	1.767938
49.H	-6.938422	-2.797124	2.276598
50.H	3.094436	-4.043439	1.974002
51.H	-5.200224	-2.415677	2.411768
52.H	4.122415	-2.594433	1.982811
53.H	-1.918328	-4.500835	2.281213
54.Th	-1.084397	-0.009534	1.998536
55.C	0.256538	-4.678698	2.324399
56.H	2.685107	1.865749	2.740525
57.H	-2.383397	5.596987	2.634891
58.C	-1.157742	-5.252818	2.537405
59.C	2.940295	2.928419	2.621866
60.H	0.988382	-5.463847	2.604148
61.H	-5.392124	0.921724	2.729915
62.C	3.331840	-3.129747	2.538686
63.C	-4.287233	0.822536	2.814168
64.H	3.999215	3.055561	2.917671
65.C	2.105668	-2.224687	2.754460
66.H	3.298688	-0.410252	2.994483
67.H	-0.060077	6.786719	2.963586
68.H	-3.926360	1.868879	2.932793
69.Si	0.131813	3.544302	3.041076
70.H	2.250008	4.875292	3.234373
71.H	-1.341314	-5.563428	3.576685
72.C	-2.266869	5.122408	3.621715
73.C	2.014127	3.818781	3.469002
74.H	-2.763803	4.143172	3.569291
75.Si	0.570754	-3.133525	3.472135
76.N	-0.737039	-2.032936	3.544282
77.N	-0.506635	2.024622	3.501320
78.H	3.774341	-3.444874	3.500681
79.C	2.527413	-0.975244	3.545471
80.H	-2.799845	-2.549293	3.821651
81.H	-2.818349	5.747032	4.349775
82.C	-0.105210	6.371230	3.983300
83.C	-0.781098	4.988734	4.001268
84.H	-4.343967	-0.954996	4.018115
85.H	0.925080	6.334959	4.366799
86.H	2.954082	-1.236830	4.529303
87.C	-4.034262	0.091984	4.146627
88.H	1.684705	-0.289798	3.718450
89.C	-1.885358	-2.296114	4.405271
90.N	-2.612364	0.100058	4.534339
91.H	-0.663615	7.099926	4.602991
92.H	0.571233	-5.989249	4.899015
93.H	-1.749148	-3.162730	5.089294
94.H	-4.662342	0.530286	4.960670
95.C	2.304594	3.597615	4.965307
96.H	-0.754735	4.637687	5.051912
97.H	3.370258	3.780105	5.201211
98.H	-2.852574	2.161317	4.813368

99.C	-0.750289	1.735694	4.912791
100.H	2.199230	-4.097394	5.038176
101.H	2.080970	2.559607	5.254887
102.C	1.126398	-3.876576	5.207510
103.C	-2.224362	-1.094757	5.305888
104.C	-2.214375	1.353977	5.199822
105.H	-0.109557	0.901831	5.277181
106.H	1.704721	4.256320	5.613985
107.C	0.478312	-5.199158	5.658139
108.H	-0.595140	-5.071558	5.870908
109.H	-0.519655	2.581174	5.598586
110.H	-3.025182	-1.362603	6.038024
111.H	-1.320431	-0.843784	5.879263
112.H	1.516105	-1.894594	6.093911
113.H	-2.394599	1.283782	6.300448
114.H	0.945386	-5.575234	6.589699
115.C	1.050987	-2.853757	6.356409
116.H	0.005187	-2.639122	6.623407
117.H	1.548547	-3.235237	7.269090

Energy: −631.08043007 eV

Supplementary Table 9 - Complex 11 (frozen core up to and including 6p, all other atoms all-electron)

1.H	-3.443805	-2.311846	-2.799830
2.H	-4.528628	-0.907599	-2.694978
3.H	-3.594479	1.121052	-2.242862
4.H	-4.514158	2.647868	-2.208951
5.C	-4.179221	-1.799271	-2.152862
6.H	-5.043193	-2.478330	-2.046179
7.C	-4.129253	1.818811	-1.582386
8.H	-5.958848	0.681928	-1.537569
9.H	-3.378329	2.240062	-0.899334
10.H	-2.646645	-0.839957	-0.961543
11.H	-7.101032	-2.045914	-0.868523
12.C	-5.264392	1.133612	-0.801391
13.H	-6.439018	2.992309	-0.649094
14.H	-7.693649	-0.403788	-0.551397
15.C	-3.555053	-1.457731	-0.785316
16.H	-2.259766	-3.216292	-0.726558
17.H	0.738046	4.833547	-0.585802
18.H	-1.392144	3.421372	-0.528722
19.C	-7.406732	-1.350580	-0.068470
20.C	-6.051828	2.185579	0.003767
21.C	-3.045466	-2.746270	-0.113344
22.H	-8.320163	-1.774359	0.396106
23.H	-3.852316	-3.485082	0.027465
24.H	0.097518	-5.197342	0.197506
25.Si	-4.626905	-0.317795	0.338474
26.H	-6.912129	1.751255	0.538459
27.C	0.711576	4.911958	0.516599
28.H	-5.403279	2.659202	0.757028
29.H	-0.105600	-3.455472	0.543260
30.C	-1.429441	3.592241	0.559335
31.H	1.498640	-4.171267	0.586093
32.N	0.009599	-0.149842	0.268651
33.H	0.188029	5.853006	0.760050
34.H	-2.013156	4.510584	0.739401
35.H	0.516774	2.772682	0.786086
36.C	0.445813	-4.363112	0.837686
37.H	-2.600802	-2.556369	0.874267
38.H	-5.803768	-3.293713	1.068054
39.H	1.750572	5.015073	0.864242
40.C	-6.287653	-1.156265	0.973381

41.H	-2.004412	2.758791	0.990099
42.C	-0.003756	3.693096	1.131880
43.C	-6.054438	-2.465969	1.751205
44.H	2.861667	3.122704	1.562570
45.H	-6.668815	-0.418454	1.708471
46.H	-1.342878	-6.149285	1.907631
47.N	-3.670514	0.190941	1.671394
48.H	1.750239	-1.862909	1.777269
49.H	-6.957592	-2.771505	2.315210
50.H	3.095827	-4.044660	1.978691
51.H	-5.222795	-2.374908	2.464160
52.H	4.123718	-2.595451	1.995046
53.H	-1.921406	-4.514083	2.298714
54.Th	-1.070705	-0.003003	1.976950
55.C	0.253163	-4.695057	2.332809
56.H	2.674628	1.853357	2.768939
57.H	-2.415206	5.569926	2.673267
58.C	-1.161950	-5.268002	2.553002
59.C	2.937063	2.913408	2.639412
60.H	0.986743	-5.479650	2.611827
61.H	-5.419750	0.881321	2.744839
62.C	3.332549	-3.133739	2.548450
63.C	-4.312737	0.816119	2.824271
64.H	3.998581	3.036235	2.932447
65.C	2.104245	-2.230271	2.766223
66.H	3.295320	-0.413461	3.012153
67.H	-0.089081	6.792862	2.975768
68.H	-3.986411	1.871220	2.955278
69.Si	0.131135	3.551566	3.048345
70.H	2.259868	4.873134	3.234031
71.H	-1.341393	-5.575277	3.594282
72.C	-2.280657	5.105412	3.663694
73.C	2.015651	3.819519	3.476376
74.H	-2.769473	4.121451	3.625998
75.Si	0.567443	-3.148882	3.480844
76.N	-0.752058	-2.057693	3.545369
77.N	-0.491777	2.025900	3.523956
78.H	3.773650	-3.453797	3.510215
79.C	2.526030	-0.981806	3.562103
80.H	-2.809978	-2.570089	3.861892
81.H	-2.828694	5.732133	4.394457
82.C	-0.122055	6.377650	3.997314
83.C	-0.786782	4.987776	4.023235
84.H	-4.341601	-0.968503	4.015895
85.H	0.913068	6.349184	4.371237
86.H	2.953930	-1.245865	4.545070
87.C	-4.043324	0.080833	4.152536
88.H	1.685542	-0.295855	3.741427
89.C	-1.888060	-2.301993	4.425767
90.N	-2.626297	0.100993	4.549968
91.H	-0.680096	7.103337	4.623188
92.H	0.576115	-6.003333	4.922380
93.H	-1.738625	-3.154105	5.123528
94.H	-4.684278	0.506901	4.965921
95.C	2.292808	3.606046	4.977141
96.H	-0.742746	4.636872	5.074450
97.H	3.357505	3.786038	5.223975
98.H	-2.868107	2.163057	4.801634
99.C	-0.766960	1.752301	4.931080
100.H	2.196409	-4.105849	5.049916
101.H	2.061276	2.569766	5.268294
102.C	1.122631	-3.885256	5.221686

103.C	-2.223793	-1.090210	5.316517
104.C	-2.232730	1.360540	5.202453
105.H	-0.125039	0.934208	5.325026
106.H	1.687963	4.269412	5.616726
107.C	0.477377	-5.209391	5.678317
108.H	-0.598523	-5.086725	5.887560
109.H	-0.562341	2.611323	5.607057
110.H	-3.014392	-1.361930	6.061078
111.H	-1.313755	-0.835609	5.878691
112.H	1.518882	-1.899286	6.096696
113.H	-2.420987	1.305717	6.304649
114.H	0.944428	-5.580394	6.613281
115.C	1.052105	-2.855570	6.366985
116.H	0.007336	-2.635435	6.636555
117.H	1.552281	-3.232075	7.281568

Energy: −629.57156754 eV

Supplementary Table 10 - Complex 11 (frozen core up to and including 5d, all other atoms all-electron)

1.H	-3.438129	-2.313237	-2.809360
2.H	-4.524667	-0.910365	-2.699025
3.H	-3.580460	1.117482	-2.250072
4.H	-4.499120	2.644699	-2.217893
5.C	-4.169364	-1.800257	-2.157703
6.H	-5.031215	-2.480698	-2.043907
7.C	-4.112372	1.817694	-1.589629
8.H	-5.939602	0.679002	-1.537922
9.H	-3.359706	2.241807	-0.910165
10.H	-2.635295	-0.831349	-0.977147
11.H	-7.092681	-2.034499	-0.882861
12.C	-5.245174	1.134389	-0.803747
13.H	-6.422876	2.991663	-0.659516
14.H	-7.678161	-0.392070	-0.553441
15.C	-3.536775	-1.454754	-0.795123
16.H	-2.239770	-3.213510	-0.746340
17.H	0.744070	4.842790	-0.590973
18.H	-1.376433	3.419918	-0.548308
19.C	-7.394315	-1.343339	-0.077531
20.C	-6.034159	2.188411	-0.003020
21.C	-3.015832	-2.738750	-0.124007
22.H	-8.309041	-1.766673	0.385121
23.H	-3.819633	-3.477312	0.033717
24.H	0.111704	-5.185674	0.185002
25.Si	-4.608927	-0.319587	0.336749
26.H	-6.893690	1.754380	0.533076
27.C	0.715887	4.912231	0.512176
28.H	-5.386922	2.666719	0.748479
29.H	-0.101257	-3.445924	0.531170
30.C	-1.415154	3.570725	0.542945
31.H	1.507948	-4.155103	0.578057
32.N	-0.064834	-0.153280	0.370798
33.H	0.187506	5.848787	0.761739
34.H	-2.013949	4.476114	0.737988
35.H	0.539444	2.772260	0.776299
36.C	0.454676	-4.350047	0.826087
37.H	-2.554695	-2.540032	0.854178
38.H	-5.798380	-3.301644	1.040584
39.H	1.753983	5.018853	0.861824
40.C	-6.273230	-1.161299	0.964618
41.H	-1.973167	2.719248	0.960158
42.C	0.007606	3.685177	1.119043
43.C	-6.045053	-2.478575	1.730927
44.H	2.867320	3.119575	1.550698

45.H	-6.651383	-0.428453	1.706261
46.H	-1.330479	-6.142577	1.889863
47.N	-3.647951	0.179239	1.672866
48.H	1.758943	-1.860744	1.764052
49.H	-6.949701	-2.785217	2.292144
50.H	3.099666	-4.043923	1.969690
51.H	-5.213194	-2.397539	2.444959
52.H	4.132283	-2.597889	1.984626
53.H	-1.916852	-4.510385	2.282181
54.Th	-1.092979	-0.003174	1.998149
55.C	0.258629	-4.682121	2.320556
56.H	2.687481	1.844298	2.754489
57.H	-2.402375	5.578098	2.659731
58.C	-1.154333	-5.261308	2.536843
59.C	2.944130	2.905959	2.626763
60.H	0.994981	-5.463493	2.601752
61.H	-5.397093	0.902141	2.729949
62.C	3.338047	-3.132358	2.537697
63.C	-4.291656	0.821373	2.816525
64.H	4.005412	3.033308	2.919111
65.C	2.112194	-2.224415	2.752447
66.H	3.312764	-0.412626	2.998530
67.H	-0.072758	6.789573	2.964782
68.H	-3.949514	1.871797	2.944142
69.Si	0.131999	3.540053	3.037905
70.H	2.258296	4.860905	3.221835
71.H	-1.333922	-5.570700	3.577406
72.C	-2.271478	5.112425	3.650238
73.C	2.019658	3.806304	3.466394
74.H	-2.766524	4.131533	3.612435
75.Si	0.569955	-3.134729	3.470579
76.N	-0.746188	-2.034687	3.530861
77.N	-0.504241	2.013988	3.503166
78.H	3.777086	-3.451702	3.500345
79.C	2.536253	-0.975013	3.545134
80.H	-2.805286	-2.553153	3.832275
81.H	-2.815880	5.743070	4.380505
82.C	-0.107673	6.373003	3.985906
83.C	-0.778748	4.985734	4.010577
84.H	-4.339955	-0.960192	4.010229
85.H	0.927168	6.340875	4.360636
86.H	2.957417	-1.238331	4.531094
87.C	-4.036414	0.087539	4.146833
88.H	1.697100	-0.285340	3.715513
89.C	-1.885482	-2.295100	4.404195
90.N	-2.619904	0.100878	4.545040
91.H	-0.663398	7.100521	4.612029
92.H	0.581417	-5.995254	4.908202
93.H	-1.739798	-3.157363	5.090430
94.H	-4.674962	0.518495	4.958593
95.C	2.305006	3.598237	4.966334
96.H	-0.737203	4.634926	5.062021
97.H	3.370419	3.784041	5.207027
98.H	-2.854780	2.162682	4.806682
99.C	-0.756411	1.738561	4.915046
100.H	2.202496	-4.099414	5.031731
101.H	2.079946	2.562080	5.263211
102.C	1.129962	-3.877434	5.210136
103.C	-2.221527	-1.094199	5.306851
104.C	-2.220184	1.356406	5.200869
105.H	-0.116003	0.912514	5.294723
106.H	1.700738	4.261721	5.606380

107.C	0.485852	-5.202557	5.665961
108.H	-0.589111	-5.080184	5.880281
109.H	-0.534288	2.592492	5.592074
110.H	-3.014544	-1.368583	6.047123
111.H	-1.312141	-0.844311	5.872003
112.H	1.536659	-1.897009	6.093472
113.H	-2.401589	1.296449	6.303421
114.H	0.957008	-5.575612	6.598278
115.C	1.067996	-2.853333	6.360645
116.H	0.025105	-2.631873	6.636869
117.H	1.571717	-3.236577	7.270747

Energy: −631.00835994 eV

Supplementary Table 11 - Complex 12 (all electron)

1.H	-3.335047	-2.329257	-2.802954
2.H	-4.404502	-0.909987	-2.715529
3.H	-3.437653	1.120317	-2.224531
4.H	-4.377241	2.634496	-2.238271
5.C	-4.061070	-1.796913	-2.160523
6.H	-4.931335	-2.464023	-2.037173
7.C	-3.989580	1.826410	-1.586964
8.H	-5.813783	0.687625	-1.545807
9.H	-3.258251	2.275950	-0.904016
10.H	-2.535367	-0.807337	-0.988986
11.H	-6.983948	-2.076722	-0.896820
12.C	-5.126713	1.146212	-0.806786
13.H	-6.312891	2.996618	-0.689883
14.H	-7.566380	-0.423006	-0.602100
15.C	-3.426791	-1.438505	-0.802536
16.H	-2.161725	-3.218738	-0.751135
17.H	0.739356	4.755309	-0.647705
18.H	-1.339556	3.350212	-0.615336
19.C	-7.283840	-1.366404	-0.106877
20.C	-5.927305	2.202657	-0.020654
21.C	-2.902497	-2.708816	-0.113870
22.H	-8.199201	-1.776733	0.366108
23.H	-3.708683	-3.427963	0.098974
24.H	0.086432	-5.065745	0.092276
25.Si	-4.515898	-0.317786	0.319263
26.H	-6.791990	1.767962	0.507580
27.C	0.725982	4.815094	0.456590
28.H	-5.292261	2.693516	0.733238
29.H	-0.168702	-3.344216	0.473506
30.C	-1.381692	3.433340	0.483345
31.H	1.462976	-4.011515	0.490244
32.N	-0.238252	-0.103841	0.541856
33.H	0.194065	5.744814	0.722018
34.H	-2.022456	4.294649	0.729709
35.H	0.585052	2.676046	0.727113
36.C	0.416639	-4.234395	0.744006
37.H	-2.398503	-2.478333	0.837136
38.H	-5.692230	-3.306295	1.045718
39.H	1.768337	4.925234	0.794797
40.C	-6.166413	-1.166151	0.936742
41.H	-1.891041	2.526753	0.848277
42.C	0.034711	3.577394	1.060530
43.C	-5.930976	-2.470404	1.723546
44.H	2.855121	2.997150	1.495818
45.H	-6.555853	-0.426980	1.666567
46.H	-1.325930	-6.079475	1.808951
47.N	-3.565907	0.174153	1.678433
48.H	1.732624	-1.805387	1.670428

49.H	-6.832199	-2.764199	2.297173
50.H	3.084572	-3.967424	1.868885
51.H	-5.093234	-2.379143	2.430932
52.H	4.114950	-2.517607	1.898513
53.H	-1.940437	-4.462354	2.219018
54.U	-1.172331	0.001757	2.114300
55.C	0.241030	-4.596182	2.232727
56.H	2.695545	1.742094	2.727652
57.H	-2.407250	5.498841	2.588825
58.C	-1.159966	-5.198973	2.459517
59.C	2.944013	2.800611	2.574595
60.H	0.990759	-5.372154	2.490569
61.H	-5.351350	0.910592	2.665068
62.C	3.318863	-3.058045	2.443220
63.C	-4.251079	0.839872	2.789181
64.H	4.007121	2.937744	2.853653
65.C	2.089430	-2.154583	2.658153
66.H	3.309186	-0.359912	2.921096
67.H	-0.088798	6.717738	2.882859
68.H	-3.914216	1.890962	2.906663
69.Si	0.146089	3.461587	2.978100
70.H	2.262618	4.760178	3.150153
71.H	-1.318880	-5.519227	3.500214
72.C	-2.267918	5.020256	3.572218
73.C	2.025094	3.709079	3.409393
74.H	-2.759267	4.036923	3.525700
75.Si	0.566889	-3.075734	3.398127
76.N	-0.757113	-1.970391	3.464509
77.N	-0.494500	1.931208	3.465520
78.H	3.752452	-3.382349	3.405805
79.C	2.500698	-0.899058	3.443640
80.H	-2.798793	-2.544332	3.776437
81.H	-2.811259	5.638010	4.314583
82.C	-0.108094	6.292911	3.901367
83.C	-0.773056	4.902190	3.927743
84.H	-4.334380	-0.931031	3.990466
85.H	0.931355	6.265249	4.266812
86.H	2.875996	-1.147706	4.449802
87.C	-4.015338	0.111757	4.116768
88.H	1.662829	-0.195950	3.564049
89.C	-1.883870	-2.280503	4.347087
90.N	-2.583005	0.108964	4.486741
91.H	-0.662924	7.009696	4.540262
92.H	0.616590	-5.926917	4.823138
93.H	-1.705029	-3.151247	5.015675
94.H	-4.622222	0.556451	4.938818
95.C	2.318140	3.523265	4.910527
96.H	-0.728302	4.558837	4.981837
97.H	3.382596	3.725078	5.140857
98.H	-2.811923	2.169698	4.791894
99.C	-0.724204	1.707822	4.895838
100.H	2.213627	-4.004950	4.933240
101.H	2.107030	2.489678	5.226701
102.C	1.139514	-3.798612	5.123639
103.C	-2.209003	-1.096465	5.262422
104.C	-2.187704	1.354091	5.179885
105.H	-0.088409	0.888060	5.289955
106.H	1.711306	4.192420	5.543127
107.C	0.523097	-5.136194	5.585495
108.H	-0.549645	-5.030782	5.826257
109.H	-0.484598	2.581287	5.537060
110.H	-3.012713	-1.352314	5.991362

111.H	-1.300981	-0.858707	5.833159
112.H	1.510539	-1.804061	6.003068
113.H	-2.382652	1.267535	6.273734
114.H	1.023315	-5.501500	6.505912
115.C	1.080662	-2.777253	6.278224
116.H	0.040372	-2.595520	6.592757
117.H	1.627609	-3.150495	7.167369

Energy: −632.25732867 eV

Supplementary Table 12 - Complex 12 (frozen core up to and including 6p, all other atoms all-electron)

1.H	-3.331019	-2.314045	-2.789894
2.H	-4.398567	-0.893679	-2.705211
3.H	-3.472681	1.124045	-2.225358
4.H	-4.405879	2.642148	-2.227254
5.C	-4.064848	-1.785871	-2.152993
6.H	-4.938691	-2.452084	-2.048461
7.C	-4.020414	1.828046	-1.582213
8.H	-5.849578	0.693657	-1.542101
9.H	-3.284736	2.268873	-0.897617
10.H	-2.543178	-0.824121	-0.949481
11.H	-6.987741	-2.085595	-0.888136
12.C	-5.158355	1.146703	-0.803575
13.H	-6.328729	3.006869	-0.668179
14.H	-7.588843	-0.437549	-0.602800
15.C	-3.448737	-1.442862	-0.781934
16.H	-2.198655	-3.234134	-0.724273
17.H	0.755131	4.756067	-0.628011
18.H	-1.350752	3.378336	-0.595537
19.C	-7.300015	-1.376207	-0.102293
20.C	-5.950138	2.202534	-0.007663
21.C	-2.950599	-2.727760	-0.097304
22.H	-8.214841	-1.794787	0.365432
23.H	-3.766875	-3.443775	0.085848
24.H	0.063183	-5.080080	0.116298
25.Si	-4.540607	-0.311539	0.329178
26.H	-6.818266	1.770534	0.516826
27.C	0.735760	4.821845	0.475676
28.H	-5.309344	2.678462	0.750492
29.H	-0.144451	-3.350233	0.494124
30.C	-1.393643	3.477407	0.501958
31.H	1.467837	-4.062030	0.508688
32.N	-0.179854	-0.124485	0.445518
33.H	0.210092	5.757754	0.732279
34.H	-2.011438	4.359947	0.734144
35.H	0.560914	2.682541	0.743056
36.C	0.416658	-4.256801	0.766155
37.H	-2.468686	-2.517310	0.869508
38.H	-5.713084	-3.301406	1.066320
39.H	1.776641	4.925641	0.819387
40.C	-6.189923	-1.162802	0.946235
41.H	-1.930281	2.592598	0.880060
42.C	0.026591	3.593646	1.081043
43.C	-5.949345	-2.461984	1.740223
44.H	2.871340	3.034409	1.522720
45.H	-6.586400	-0.422563	1.671010
46.H	-1.357374	-6.067646	1.831716
47.N	-3.605327	0.200974	1.684330
48.H	1.729004	-1.793532	1.703722
49.H	-6.846842	-2.754662	2.320571
50.H	3.075584	-3.969545	1.872375
51.H	-5.107299	-2.365266	2.441692
52.H	4.106691	-2.521045	1.900800

53.H	-1.940486	-4.439491	2.245396
54.U	-1.148630	-0.003342	2.072784
55.C	0.236694	-4.611989	2.256860
56.H	2.690194	1.769703	2.741386
57.H	-2.413717	5.498811	2.599587
58.C	-1.174541	-5.191423	2.483718
59.C	2.947193	2.828575	2.600728
60.H	0.974249	-5.398772	2.517898
61.H	-5.385272	0.902658	2.698468
62.C	3.316130	-3.064549	2.450251
63.C	-4.282083	0.843863	2.809074
64.H	4.007920	2.957336	2.892543
65.C	2.090487	-2.160412	2.684982
66.H	3.318188	-0.372373	2.961589
67.H	-0.102694	6.722420	2.890241
68.H	-3.957108	1.898431	2.932543
69.Si	0.144477	3.473437	2.999328
70.H	2.263852	4.788147	3.182611
71.H	-1.339428	-5.509945	3.524003
72.C	-2.276326	5.019673	3.582584
73.C	2.023349	3.735533	3.433135
74.H	-2.757095	4.031712	3.529328
75.Si	0.567590	-3.087449	3.420049
76.N	-0.762193	-1.995383	3.491271
77.N	-0.476514	1.946757	3.502963
78.H	3.759786	-3.395685	3.406161
79.C	2.515693	-0.918527	3.486886
80.H	-2.807900	-2.551254	3.801774
81.H	-2.830021	5.630640	4.322807
82.C	-0.124112	6.306061	3.911971
83.C	-0.782190	4.912547	3.945026
84.H	-4.345388	-0.932881	4.006346
85.H	0.914150	6.286154	4.280623
86.H	2.905452	-1.186051	4.482993
87.C	-4.029723	0.111354	4.134475
88.H	1.681483	-0.215761	3.630358
89.C	-1.892039	-2.285993	4.371610
90.N	-2.599605	0.111746	4.508854
91.H	-0.683851	7.026365	4.543148
92.H	0.627432	-5.940285	4.845942
93.H	-1.721838	-3.151927	5.050879
94.H	-4.642813	0.550888	4.957092
95.C	2.304792	3.536202	4.934773
96.H	-0.739091	4.573726	5.000751
97.H	3.369492	3.725120	5.174710
98.H	-2.826101	2.173710	4.801131
99.C	-0.735525	1.720606	4.923778
100.H	2.220795	-4.015119	4.954819
101.H	2.080398	2.502095	5.238833
102.C	1.145665	-3.810940	5.142470
103.C	-2.220422	-1.093920	5.279680
104.C	-2.203087	1.358878	5.194002
105.H	-0.101958	0.905017	5.330507
106.H	1.699924	4.205333	5.569030
107.C	0.530917	-5.148161	5.606752
108.H	-0.543013	-5.043610	5.844193
109.H	-0.513088	2.595702	5.570673
110.H	-3.022942	-1.356701	6.010092
111.H	-1.313384	-0.855252	5.852259
112.H	1.498499	-1.808592	6.008789
113.H	-2.400417	1.281058	6.289760
114.H	1.029082	-5.512024	6.529264

115.C	1.080298	-2.784116	6.292284
116.H	0.038588	-2.610854	6.606583
117.H	1.632810	-3.146088	7.182823

Energy: −631.06153200 eV

Supplementary Table 13 - Complex 12 (frozen core up to and including 5d, all other atoms all-electron)

1.H	-3.331667	-2.327990	-2.800753
2.H	-4.400610	-0.907915	-2.713591
3.H	-3.439396	1.124152	-2.222156
4.H	-4.381408	2.636800	-2.235166
5.C	-4.058231	-1.795475	-2.158838
6.H	-4.929137	-2.462286	-2.037633
7.C	-3.992379	1.829116	-1.584126
8.H	-5.816154	0.688773	-1.541995
9.H	-3.261655	2.279596	-0.901078
10.H	-2.532224	-0.810380	-0.982947
11.H	-6.979427	-2.076960	-0.895782
12.C	-5.128250	1.146872	-0.803484
13.H	-6.311916	2.998640	-0.683848
14.H	-7.564545	-0.423820	-0.603198
15.C	-3.425952	-1.439477	-0.799134
16.H	-2.167068	-3.225260	-0.747189
17.H	0.742889	4.755727	-0.645166
18.H	-1.338849	3.356804	-0.615620
19.C	-7.281536	-1.366560	-0.106829
20.C	-5.927338	2.203206	-0.015755
21.C	-2.906782	-2.712702	-0.110295
22.H	-8.197496	-1.777611	0.364680
23.H	-3.715866	-3.429272	0.100797
24.H	0.079973	-5.059736	0.094209
25.Si	-4.514750	-0.316919	0.322746
26.H	-6.792477	1.769264	0.512179
27.C	0.728291	4.815659	0.459195
28.H	-5.290975	2.691852	0.738275
29.H	-0.163158	-3.336883	0.478175
30.C	-1.381535	3.435668	0.483749
31.H	1.464166	-4.016042	0.493216
32.N	-0.237566	-0.110461	0.533598
33.H	0.195912	5.745582	0.723235
34.H	-2.022577	4.296305	0.732615
35.H	0.585762	2.676308	0.728262
36.C	0.416202	-4.231742	0.747138
37.H	-2.403114	-2.485491	0.841939
38.H	-5.694428	-3.305627	1.050900
39.H	1.770162	4.926370	0.798823
40.C	-6.166010	-1.165039	0.938362
41.H	-1.891698	2.528233	0.845258
42.C	0.035469	3.577895	1.062187
43.C	-5.931673	-2.468306	1.727363
44.H	2.858739	3.000156	1.500446
45.H	-6.556396	-0.425065	1.666948
46.H	-1.334365	-6.070281	1.809817
47.N	-3.568289	0.176438	1.683143
48.H	1.731722	-1.798398	1.674468
49.H	-6.832754	-2.760495	2.302383
50.H	3.081594	-3.964319	1.869342
51.H	-5.093093	-2.376685	2.433663
52.H	4.112473	-2.514779	1.896348
53.H	-1.941068	-4.450568	2.221226
54.U	-1.170453	-0.000756	2.107190
55.C	0.238997	-4.593836	2.235931
56.H	2.698256	1.744142	2.731487

57.H	-2.408494	5.497348	2.590865
58.C	-1.164666	-5.191078	2.461255
59.C	2.945966	2.803078	2.579415
60.H	0.985784	-5.372501	2.494524
61.H	-5.353930	0.913458	2.669175
62.C	3.317440	-3.054911	2.442950
63.C	-4.253293	0.842645	2.792997
64.H	4.008602	2.941196	2.860152
65.C	2.089040	-2.150591	2.661223
66.H	3.312487	-0.358380	2.927924
67.H	-0.092437	6.717969	2.885298
68.H	-3.916110	1.893794	2.909540
69.Si	0.145213	3.459109	2.980227
70.H	2.260732	4.761669	3.155755
71.H	-1.325486	-5.511481	3.501475
72.C	-2.269199	5.017747	3.573630
73.C	2.024185	3.710041	3.413398
74.H	-2.759259	4.033971	3.525215
75.Si	0.565598	-3.072232	3.401113
76.N	-0.760752	-1.969978	3.468708
77.N	-0.493061	1.928869	3.470263
78.H	3.753526	-3.379412	3.404387
79.C	2.503135	-0.897263	3.449878
80.H	-2.802573	-2.543483	3.779401
81.H	-2.813590	5.634078	4.316644
82.C	-0.111099	6.292433	3.903518
83.C	-0.774199	4.900724	3.929408
84.H	-4.339358	-0.926845	3.995915
85.H	0.928366	6.266699	4.269121
86.H	2.879052	-1.149150	4.455060
87.C	-4.018033	0.115515	4.121406
88.H	1.666615	-0.192857	3.572814
89.C	-1.887618	-2.279825	4.350288
90.N	-2.586402	0.110343	4.492541
91.H	-0.666826	7.008283	4.542946
92.H	0.621754	-5.925685	4.824073
93.H	-1.709815	-3.151137	5.019546
94.H	-4.625745	0.562077	4.942558
95.C	2.315741	3.522077	4.914516
96.H	-0.729719	4.557929	4.983929
97.H	3.380318	3.722110	5.146140
98.H	-2.811862	2.171348	4.796173
99.C	-0.724656	1.708148	4.900114
100.H	2.215119	-4.001414	4.933414
101.H	2.102771	2.488407	5.228728
102.C	1.140935	-3.796252	5.124262
103.C	-2.213215	-1.096106	5.266130
104.C	-2.188541	1.354924	5.184021
105.H	-0.088756	0.889789	5.297026
106.H	1.709297	4.191210	5.547410
107.C	0.526763	-5.134811	5.586443
108.H	-0.546359	-5.031404	5.827647
109.H	-0.487136	2.583133	5.540846
110.H	-3.017380	-1.354202	5.994619
111.H	-1.305626	-0.860146	5.838412
112.H	1.506334	-1.799662	6.001007
113.H	-2.382513	1.270386	6.278793
114.H	1.027999	-5.499856	6.506731
115.C	1.081014	-2.774132	6.278347
116.H	0.040657	-2.596191	6.594677
117.H	1.631215	-3.144545	7.166863

Energy: −632.41823352 eV

Supplementary Table 14 - [Th(Tren^{TIPS})(NH)]$^-$

1.C	0.347954	1.828191	-6.396268
2.C	1.586791	-1.552504	-5.245621
3.C	-2.843229	0.546402	-4.680423
4.C	-1.607141	-0.366696	-4.787969
5.C	0.436587	1.947241	-4.863229
6.C	-1.954144	-1.797693	-4.336654
7.C	1.811336	2.511465	-4.456196
8.C	1.251656	-1.007437	-3.843790
9.C	2.552295	-0.662559	-3.100693
10.C	-1.100828	2.068942	-1.917820
11.C	-3.862378	-1.084697	-1.017604
12.C	-0.267048	3.002341	-1.026770
13.C	4.389189	-2.081668	-0.050586
14.C	5.784621	-0.059968	0.497185
15.C	-3.584597	-1.801800	0.314824
16.C	-4.686536	-2.845612	0.581252
17.C	2.398785	1.868623	0.514791
18.C	-5.017180	1.557737	1.054304
19.C	4.804302	-1.105171	1.062744
20.C	1.251124	2.813452	0.911672
21.C	-1.185418	2.644305	1.228985
22.C	-4.650767	0.604140	2.206823
23.C	-5.926353	-0.036771	2.784944
24.C	-1.413811	1.450122	2.168107
25.C	-2.072514	-2.932585	3.124803
26.C	2.315092	-1.788377	2.788323
27.C	3.164781	-2.992850	3.237914
28.C	4.091118	0.862043	3.280773
29.C	-3.057411	-1.771832	3.355055
30.C	5.062190	0.140993	4.236829
31.C	3.128009	1.751130	4.087702
32.C	1.408290	-1.338096	3.948761
33.C	-2.664713	-0.974270	4.612664
34.H	0.612267	2.785229	-6.881137
35.H	1.040507	1.067192	-6.788179
36.H	-0.662302	1.557694	-6.734585
37.H	-1.322440	-0.415503	-5.855871
38.H	0.691343	-1.840771	-5.814822
39.H	2.131532	-0.805233	-5.844768
40.H	-3.692955	0.138936	-5.256189
41.H	-2.647802	1.562504	-5.057192
42.H	2.235632	-2.442317	-5.176643
43.H	-2.879054	-2.153616	-4.824427
44.H	1.963966	3.528347	-4.858584
45.H	2.627755	1.885426	-4.847372
46.H	-1.156209	-2.512579	-4.582570
47.H	-0.315126	2.704119	-4.567811
48.H	-3.173077	0.639561	-3.635224
49.H	-2.109759	-1.852643	-3.249794
50.H	1.932669	2.558967	-3.364576
51.H	0.770023	-1.820330	-3.265902
52.H	3.138064	0.104281	-3.629213
53.H	3.189626	-1.555928	-3.002911
54.H	-1.362182	2.642672	-2.828222
55.H	2.375022	-0.280305	-2.083414
56.H	-3.932604	-1.812000	-1.843020
57.H	0.713307	3.143051	-1.503776
58.H	-2.073995	1.892064	-1.412739
59.H	-3.069325	-0.369187	-1.281680
60.H	-4.812916	-0.530274	-0.994316

61.H	-0.744243	4.002333	-0.932803
62.H	5.266443	-2.610867	-0.463742
63.H	3.910290	-1.541887	-0.877867
64.H	2.535792	1.936909	-0.586193
65.H	-4.744050	-3.569899	-0.249473
66.H	0.146561	-3.449112	-0.465621
67.H	5.289054	0.590219	-0.240840
68.H	6.630173	-0.547411	-0.020471
69.H	-5.594903	1.036851	0.276326
70.H	3.666078	-2.837429	0.286989
71.H	-2.626424	-2.347779	0.188192
72.H	-4.127384	1.979992	0.566681
73.H	-5.679158	-2.374112	0.663530
74.H	-5.639206	2.397878	1.410334
75.H	3.327332	2.302371	0.937871
76.H	1.489707	3.865404	0.644214
77.H	-2.088668	2.773127	0.616829
78.H	6.209480	0.587214	1.278870
79.H	-4.516641	-3.417046	1.504815
80.H	5.345512	-1.685202	1.834345
81.H	-6.424706	-0.680931	2.044281
82.H	-1.032375	3.583153	1.803433
83.H	-2.418495	-3.621052	2.341194
84.H	1.657697	-2.153040	1.969152
85.H	1.136846	2.766906	2.003159
86.H	3.761122	-3.416405	2.418762
87.H	-6.657379	0.736522	3.082633
88.H	-1.086576	-2.565912	2.803250
89.H	4.705030	1.546309	2.663677
90.H	-4.223157	1.230970	3.013051
91.H	-0.495940	1.318592	2.780547
92.H	-2.192896	1.756973	2.893103
93.H	5.825520	-0.440709	3.700532
94.H	-5.719543	-0.653493	3.671118
95.H	-1.928095	-3.521929	4.047985
96.H	2.516278	-3.798573	3.622425
97.H	-4.057651	-2.209116	3.535342
98.H	2.468510	2.339656	3.437015
99.H	3.859979	-2.723193	4.049152
100.H	0.766985	-0.489580	3.669508
101.H	4.526755	-0.553661	4.902542
102.H	0.748130	-2.157654	4.275687
103.H	5.590114	0.863755	4.884363
104.H	2.483136	1.159548	4.753050
105.H	3.687758	2.461572	4.722623
106.H	-1.680401	-0.499413	4.485451
107.H	2.002137	-1.033090	4.824838
108.H	-3.389237	-0.179348	4.848178
109.H	-2.596192	-1.631152	5.497662
110.N	-0.400525	0.804826	-2.196930
111.N	0.122832	-2.430149	-0.338283
112.N	-0.042743	2.407964	0.312237
113.N	2.146207	0.486513	0.946506
114.N	-1.752265	0.225904	1.425937
115.Si	-0.084580	0.371461	-3.844590
116.Si	-3.210256	-0.636421	1.796522
117.Si	3.277619	-0.331653	1.972847
118.Th	0.075378	-0.386928	-0.083333

Energy: −638.21100499 eV

Supplementary Table 15 - [Th(Tren^{TIPS})(NPh)]$^{-}$

1.H	-0.721224	0.217536	-6.179969

2.H	0.654459	1.313391	-5.935151
3.H	1.510080	4.952479	-5.503597
4.H	0.018142	3.988866	-5.554282
5.C	-0.078882	0.681746	-5.411381
6.C	0.759452	4.437810	-4.878090
7.H	-1.407286	2.305667	-4.935979
8.H	0.473968	-0.133681	-4.919664
9.H	1.947126	2.678870	-4.626181
10.H	-2.636888	0.128253	-4.617267
11.H	0.243552	5.213775	-4.291819
12.C	-0.919972	1.473839	-4.392938
13.C	1.427129	3.394586	-3.960879
14.H	-0.912425	-2.415736	-3.949181
15.H	3.266571	4.565077	-3.742104
16.C	-2.030571	0.581336	-3.813774
17.H	0.226256	-3.749457	-3.699323
18.C	-0.222254	-2.866245	-3.217894
19.H	-1.756581	4.865841	-3.199651
20.H	2.688720	1.069085	-3.271055
21.C	2.513557	4.069680	-3.103893
22.H	-2.714781	1.124498	-3.148494
23.H	-1.606989	-0.239879	-3.217399
24.H	-2.755091	3.399093	-3.242018
25.H	-2.759163	-3.877320	-3.003077
26.H	2.170551	-4.490815	-2.971294
27.H	0.584988	-2.142377	-3.033848
28.Si	0.153635	2.280434	-3.003359
29.H	-1.648811	-5.212262	-2.646625
30.H	2.088636	4.840746	-2.445527
31.H	3.042604	3.352206	-2.463316
32.C	-2.158571	4.045687	-2.584341
33.H	0.368173	-6.308150	-2.359237
34.H	2.214123	-0.492898	-2.625235
35.C	-2.068173	-4.277422	-2.242214
36.C	2.268017	0.586181	-2.368196
37.H	3.380028	-5.422033	-2.069140
38.C	2.599139	-4.647102	-1.971072
39.H	-2.849671	4.496549	-1.853159
40.C	-0.961530	-3.253545	-1.923900
41.H	1.667973	-7.186572	-1.535951
42.H	3.095218	-3.711600	-1.682652
43.N	0.954032	1.123956	-1.979105
44.C	-1.035025	3.275588	-1.863913
45.C	0.893410	-6.411787	-1.397163
46.H	-1.468958	-2.341712	-1.542224
47.H	4.310915	0.435422	-1.567884
48.H	-2.668586	-4.537080	-1.360358
49.H	0.138226	5.073784	-1.416259
50.C	3.298784	0.758670	-1.243136
51.H	-1.529290	2.484682	-1.260497
52.H	3.353377	1.827333	-0.991860
53.C	1.523787	-5.076310	-0.955500
54.H	0.168612	-6.797116	-0.665973
55.H	3.369726	-1.706232	-1.091562
56.C	-0.300471	4.212953	-0.887448
57.Si	0.215942	-3.718620	-0.477118
58.H	0.514269	3.700929	-0.354287
59.H	2.049962	-5.278341	-0.002602
60.H	-0.991336	4.617228	-0.130246
61.C	3.318542	-1.391366	-0.039817
62.N	2.890573	0.029722	-0.016949
63.Th	0.086222	0.019347	0.009056

64.N	-2.030644	-0.004753	0.012039
65.N	0.986369	-2.243030	0.030032
66.H	-1.312855	-5.441536	0.452459
67.H	4.335298	-1.497092	0.394562
68.H	-2.694618	-3.349272	0.533928
69.C	-0.863573	-4.534962	0.900795
70.H	2.742145	-3.310795	0.709974
71.C	2.306364	-2.293729	0.680227
72.H	2.248797	2.555025	0.855041
73.H	4.340444	1.158509	1.081720
74.C	3.327990	0.722582	1.219746
75.C	-2.013889	-3.617944	1.353030
76.H	-1.576940	3.045692	1.621749
77.H	0.741996	-5.721007	1.849537
78.C	2.313923	1.789153	1.656797
79.H	-1.632462	-2.670776	1.761975
80.H	2.256099	-1.978483	1.743917
81.N	0.999894	1.192618	1.953607
82.C	-0.027958	-4.980707	2.115485
83.H	-2.616886	-4.098484	2.142913
84.H	3.392466	-0.031063	2.016833
85.H	0.734821	4.314051	2.302088
86.H	-2.294435	4.293732	2.663591
87.H	0.481514	-4.122578	2.580720
88.H	2.756631	2.324975	2.519043
89.C	-1.852095	3.282004	2.660298
90.H	-0.667184	-5.436042	2.891742
91.H	-2.645339	2.579223	2.945268
92.H	-0.220995	5.345029	3.381614
93.H	-1.524142	0.024366	2.792186
94.C	0.314841	4.381968	3.317325
95.H	0.413413	-1.522876	3.293313
96.C	-0.636911	3.203137	3.599419
97.Si	0.273385	1.495768	3.503580
98.H	1.156451	4.427281	4.024770
99.C	-1.011134	0.090303	3.775293
100.C	-0.352754	-1.276828	4.043461
101.H	-1.100797	-2.085862	4.030503
102.H	3.309805	0.590004	4.035676
103.H	-1.005862	3.312607	4.636890
104.H	2.284669	-0.492492	4.990545
105.H	-2.619911	1.312306	4.672840
106.C	2.689342	0.529237	4.939632
107.H	0.132456	-1.300401	5.032229
108.H	-2.868252	-0.438463	4.799124
109.C	-2.103668	0.355385	4.829364
110.H	2.069603	2.572343	4.808922
111.C	1.575395	1.592300	4.949227
112.H	3.360842	0.659061	5.807657
113.H	-1.687775	0.368491	5.848491
114.C	0.907592	1.633820	6.339146
115.H	0.453698	0.663329	6.591729
116.H	0.116098	2.394318	6.406341
117.H	1.649185	1.856232	7.126542
118.C	-3.395851	-0.038242	0.016562
119.C	-4.174589	0.571094	1.049531
120.C	-4.153301	-0.687089	-1.011288
121.C	-5.567319	0.541547	1.050557
122.H	-3.639622	1.076144	1.855360
123.C	-5.546410	-0.712511	-1.002900
124.H	-3.603927	-1.167320	-1.823739
125.C	-6.279111	-0.098797	0.024322

126.H	-6.111763	1.027627	1.866147
127.H	-6.076215	-1.220222	-1.815392
128.H	-7.370551	-0.118194	0.025763

Energy: −706.06297136 eV

Supplementary Table 16 - [Th(Tren^{TIPS})(NAd)]$^{-}$

1.H	1.282112	-4.063059	-5.189776
2.H	0.587395	1.134115	-4.884950
3.H	-4.244445	0.081741	-4.601694
4.H	-4.815437	3.865243	-4.574465
5.H	2.873834	-5.760335	-4.217406
6.H	-5.330802	2.227359	-4.121846
7.H	-2.856247	2.340146	-4.304708
8.C	0.946158	-3.857199	-4.158219
9.H	-1.682880	0.526664	-4.303332
10.H	0.505416	-2.849632	-4.143201
11.H	-5.159713	-1.298409	-3.956499
12.H	1.692762	-0.721613	-3.993351
13.H	-2.517889	4.737592	-3.977160
14.H	0.137641	-4.570588	-3.936038
15.H	3.115872	0.353151	-3.958404
16.C	-4.777299	3.108894	-3.769882
17.C	-4.313124	-0.618524	-3.754715
18.C	0.261798	1.289502	-3.834164
19.H	-0.047147	2.339202	-3.734201
20.H	-3.398924	-1.230541	-3.756444
21.H	2.924759	-3.354429	-3.519750
22.H	-0.624401	-0.655208	-3.557192
23.H	2.150658	2.977646	-3.427830
24.C	2.643863	-5.446636	-3.183202
25.C	-3.319487	2.775991	-3.398331
26.C	-0.944509	0.405736	-3.487020
27.C	2.277866	-0.049236	-3.349708
28.C	2.105204	-4.002627	-3.154150
29.H	-5.330912	3.523086	-2.913065
30.C	-2.551599	4.076600	-3.093337
31.H	3.564606	-5.559651	-2.593832
32.H	1.909175	-6.163912	-2.784970
33.N	1.387309	1.047221	-2.900266
34.H	-5.429939	0.678990	-2.454338
35.H	-1.515949	3.886228	-2.776933
36.C	2.119435	2.290749	-2.555467
37.C	-4.488994	0.099718	-2.402760
38.H	3.564801	-1.563408	-2.556316
39.H	3.155410	2.022919	-2.303401
40.H	-3.039523	4.645349	-2.286751
41.H	-1.339115	-3.723925	-2.458613
42.C	2.799584	-0.868086	-2.159649
43.N	-1.483631	0.712940	-2.149794
44.Si	-3.077802	1.383702	-2.054943
45.H	-5.421718	-1.682399	-1.529233
46.H	-0.157196	-5.879255	-1.535132
47.H	4.755940	-3.666478	-1.552501
48.H	0.416694	3.149123	-1.566041
49.H	4.138072	4.232991	-1.312194
50.H	3.361132	-0.182682	-1.491586
51.C	-4.643899	-0.939361	-1.278420
52.C	1.485475	2.962846	-1.327639
53.Si	1.702314	-3.276564	-1.391623
54.N	1.708382	-1.544940	-1.433975
55.C	-1.242828	-3.305529	-1.445902
56.H	-1.241734	-2.209215	-1.551849

57.H	1.924395	3.972593	-1.220308
58.H	-3.705527	-1.487827	-1.108665
59.H	4.916703	1.743878	-0.732827
60.H	-2.161199	-3.565697	-0.893917
61.H	-4.929957	3.521163	-0.559709
62.H	5.431215	4.968507	-0.355126
63.C	-0.118947	-5.376881	-0.555081
64.H	-4.927225	-0.478021	-0.321914
65.C	4.415084	4.539902	-0.292663
66.C	-0.003224	-3.847400	-0.712848
67.H	-5.563155	1.877644	-0.342288
68.C	4.451792	-3.510164	-0.505916
69.H	3.044425	-5.132788	-0.426628
70.H	6.307399	2.668539	-0.129152
71.H	3.733027	5.350859	0.001927
72.H	0.723787	-5.809449	0.003359
73.Th	0.215607	0.142851	-0.454536
74.H	4.510689	-2.430259	-0.302787
75.C	3.037282	-4.047125	-0.213927
76.H	-1.044443	-5.648481	-0.019630
77.C	-3.356942	2.018208	-0.262240
78.C	-4.774263	2.573002	-0.020188
79.H	-2.368683	3.984502	-0.261337
80.C	5.328808	2.243780	0.157686
81.H	5.204433	-4.003736	0.133665
82.N	1.623901	2.125041	-0.121977
83.H	-1.277768	2.660747	0.162272
84.C	-2.313924	3.019513	0.263415
85.H	-0.024031	-3.399024	0.299209
86.C	4.383328	3.341318	0.678969
87.H	-3.242976	1.100802	0.346479
88.H	5.517490	1.465224	0.908231
89.H	-4.936357	2.783973	1.050623
90.H	1.360201	4.833075	1.096485
91.Si	2.599838	2.731626	1.171412
92.H	4.799751	3.698942	1.640578
93.H	2.695497	-0.376603	1.094889
94.H	-2.474189	3.219241	1.335785
95.C	2.708768	-3.871500	1.278845
96.N	-0.652648	-0.518845	1.312133
97.H	1.781604	-4.387939	1.564248
98.H	4.303075	0.012533	1.714412
99.H	-2.520679	-2.458316	1.583505
100.H	2.582460	-2.811291	1.540815
101.H	-3.299231	-0.875907	1.739402
102.H	3.519073	-4.274560	1.912128
103.H	3.474053	5.645229	2.186764
104.C	1.745451	4.281446	1.976091
105.C	3.236775	-0.016502	1.983273
106.C	-2.665203	-1.606323	2.268239
107.H	-0.125128	3.161377	2.285364
108.C	2.626766	5.262567	2.772795
109.C	-1.278235	-0.949020	2.532156
110.C	2.733994	1.344369	2.495178
111.H	-0.241853	-2.856492	2.618855
112.H	3.118441	-0.788655	2.760991
113.H	2.037864	6.134650	3.108493
114.C	0.521780	3.872249	2.816805
115.H	1.683127	1.190424	2.803978
116.H	-0.091068	4.749451	3.087773
117.H	-4.324338	-2.524276	3.344249
118.H	-2.132188	0.994670	2.991912

119.C	-0.411660	-1.998158	3.289438
120.H	4.597424	1.938600	3.499350
121.H	3.042543	4.793108	3.678025
122.C	-3.345849	-2.067046	3.574604
123.H	-2.309950	-3.985059	3.637979
124.H	0.575478	-1.551443	3.493156
125.C	-1.512615	0.241885	3.506993
126.C	3.538115	1.755604	3.743064
127.H	0.830760	3.391810	3.758520
128.H	-0.538975	0.715782	3.714612
129.H	-4.209569	-0.112861	4.012572
130.C	-2.448058	-3.101894	4.284445
131.H	3.146492	2.668145	4.214336
132.H	3.516509	0.956889	4.504364
133.C	-3.552783	-0.851519	4.502223
134.C	-1.079850	-2.462334	4.601355
135.C	-2.186258	-0.209574	4.820418
136.H	-2.930501	-3.451306	5.214688
137.H	-0.435753	-3.203801	5.106011
138.H	-4.054044	-1.164821	5.435410
139.H	-2.333799	0.661718	5.482784
140.C	-1.289981	-1.247271	5.528501
141.H	-0.316409	-0.793858	5.780125
142.H	-1.755049	-1.567986	6.477745

Energy: −778.13793247 eV

Supplementary Table 17 - [Th(Tren^{TIPS})(NSiMe$_3$)]$^-$

1.H	-0.676760	0.214932	-6.204817
2.H	0.702823	1.301924	-5.943303
3.H	1.551116	4.951121	-5.521260
4.H	0.058020	3.989930	-5.582369
5.C	-0.042716	0.677598	-5.428401
6.C	0.794780	4.438377	-4.901001
7.H	-1.363883	2.314579	-4.981703
8.H	0.496628	-0.139220	-4.924380
9.H	1.979830	2.679309	-4.635506
10.H	-2.618649	0.151220	-4.676054
11.H	0.275565	5.216127	-4.320113
12.C	-0.892875	1.481510	-4.426362
13.C	1.453612	3.395819	-3.976050
14.H	-0.940803	-2.451243	-3.947832
15.H	3.289103	4.569789	-3.742824
16.C	-2.021561	0.601419	-3.863760
17.H	0.224503	-3.763941	-3.707073
18.C	-0.239185	-2.891063	-3.220832
19.H	-1.702877	4.911081	-3.248249
20.H	2.694919	1.076135	-3.266171
21.C	2.531452	4.073403	-3.110628
22.H	-2.709233	1.161711	-3.215863
23.H	-1.621284	-0.221048	-3.253548
24.H	-2.706636	3.452222	-3.340409
25.H	-2.759788	-3.952562	-2.990935
26.H	2.195498	-4.476018	-2.986541
27.H	0.554888	-2.153037	-3.036559
28.Si	0.169216	2.287379	-3.024371
29.H	-1.612434	-5.264654	-2.662507
30.H	2.099447	4.844481	-2.456732
31.H	3.056211	3.357583	-2.464584
32.C	-2.126705	4.085743	-2.655175
33.H	0.411213	-6.321498	-2.382868
34.H	2.215395	-0.484776	-2.622526
35.C	-2.049983	-4.344664	-2.243509

36.C	2.267621	0.594412	-2.365616
37.H	3.407093	-5.416041	-2.096015
38.H	-4.198441	-0.897608	-2.252662
39.C	2.626012	-4.642325	-1.988655
40.H	-2.837333	4.531123	-1.939423
41.C	-0.966056	-3.297237	-1.925015
42.H	1.716670	-7.190678	-1.558571
43.H	3.122688	-3.709915	-1.691336
44.N	0.950475	1.132658	-1.986328
45.C	-1.028998	3.300848	-1.912182
46.C	0.935500	-6.422440	-1.420018
47.H	-1.492238	-2.397672	-1.541829
48.H	4.309518	0.452370	-1.562351
49.H	-2.633309	-4.631575	-1.358022
50.H	0.152254	5.087602	-1.437874
51.H	-5.622104	-1.109724	-1.204461
52.C	-4.520868	-1.159877	-1.233478
53.C	3.294318	0.766604	-1.236362
54.H	-4.239171	2.088846	-1.289371
55.H	-1.542511	2.515936	-1.317814
56.H	3.341645	1.834313	-0.979439
57.C	1.554065	-5.082423	-0.974885
58.H	-4.230528	-2.206794	-1.055597
59.H	0.212622	-6.815309	-0.691077
60.H	3.358059	-1.699597	-1.104845
61.C	-0.304854	4.229782	-0.919734
62.Si	0.231968	-3.739826	-0.487399
63.H	0.494325	3.708988	-0.371994
64.C	-4.500372	1.724864	-0.283792
65.H	-5.600554	1.696338	-0.214837
66.H	2.082401	-5.283344	-0.022906
67.H	-1.006890	4.639432	-0.175612
68.C	3.312073	-1.391958	-0.050731
69.N	2.890042	0.028938	-0.015954
70.Si	-3.730796	0.007533	0.059039
71.Th	0.070536	0.020805	0.012023
72.N	-2.026961	0.005779	0.030450
73.N	0.979730	-2.252886	0.015235
74.H	-1.244208	-5.507162	0.450286
75.H	-4.136477	2.469299	0.441009
76.H	4.330500	-1.505357	0.378911
77.H	-2.680968	-3.466061	0.550113
78.C	-0.815716	-4.590507	0.898421
79.H	2.740593	-3.312018	0.698009
80.C	2.300216	-2.296911	0.666573
81.H	2.245377	2.545115	0.872688
82.H	4.339296	1.148913	1.092165
83.C	3.326311	0.711539	1.225176
84.C	-1.986157	-3.709386	1.365319
85.H	-5.573892	-0.555270	1.703069
86.H	-1.535581	3.024070	1.561178
87.H	0.827232	-5.735786	1.832505
88.C	2.311860	1.774786	1.670237
89.C	-4.471685	-0.539201	1.735209
90.H	-1.629809	-2.749441	1.766619
91.H	2.247763	-1.981272	1.730084
92.N	0.999224	1.175512	1.965271
93.H	-4.128063	-1.549230	2.006091
94.C	0.039373	-5.017794	2.106039
95.H	-2.566691	-4.207501	2.161752
96.H	3.390216	-0.047960	2.016968
97.H	0.683098	4.317709	2.339041

98.H	-2.400043	4.154586	2.624990
99.H	0.527829	-4.147594	2.571325
100.H	2.755765	2.305885	2.534730
101.H	-4.172037	0.140876	2.547321
102.C	-1.877101	3.182518	2.594527
103.H	-0.582751	-5.493246	2.884328
104.H	-2.618783	2.399590	2.802461
105.H	-0.353352	5.312665	3.376964
106.H	-1.526574	-0.010607	2.824874
107.C	0.218899	4.369508	3.336099
108.H	0.425016	-1.546436	3.314583
109.C	-0.696928	3.154855	3.581170
110.Si	0.265865	1.477242	3.511068
111.H	1.028590	4.445309	4.077433
112.C	-1.002171	0.061524	3.801106
113.C	-0.338603	-1.302908	4.067864
114.H	-1.084392	-2.114308	4.058923
115.H	3.283282	0.567846	4.049832
116.H	-1.109201	3.247640	4.604013
117.H	2.272584	-0.469369	5.067906
118.H	-2.603944	1.286277	4.705688
119.C	2.679695	0.547812	4.966913
120.H	0.150574	-1.324250	5.054733
121.H	-2.843662	-0.464149	4.856456
122.C	-2.081747	0.332960	4.866706
123.H	2.063454	2.588674	4.783803
124.C	1.567998	1.613422	4.952786
125.H	3.366834	0.710907	5.817080
126.H	-1.652604	0.360184	5.880070
127.C	0.906531	1.692442	6.343421
128.H	0.450774	0.729783	6.622114
129.H	0.117376	2.456765	6.394802
130.H	1.651985	1.931643	7.122475

Energy: −700.82317168 eV

Supplementary Table 18 - Thorium azide complex

Th -0.009425 -0.018906 0.032005
Si -1.009125 -0.609631 3.540721
Si 0.133311 3.478273 -1.313095
Si 0.902623 -2.833396 -2.227651
N -1.433771 -0.201710 1.856539
N 4.438398 0.408126 1.445912
N 3.335593 0.311926 1.139115
N 2.177141 0.211591 0.816646
N -2.568722 -0.207464 -0.785677
N -0.288175 -1.927049 -1.261326
N -0.578614 1.842609 -1.246784
C -2.094931 -2.013940 5.822396
C -1.974829 1.797880 4.857919
C -0.705016 0.980578 4.588159
C 1.428603 -1.610160 4.791835
C -2.450055 -1.581184 4.392024
C 0.373766 1.864082 3.951601
C -3.009536 -2.767957 3.598410
C 0.644592 -1.594299 3.471536
C 0.488619 -3.021709 2.929810
C -2.828158 0.209265 1.634120
C -1.231053 4.370220 1.058637
C -3.401097 -0.483178 0.403552
C 2.300110 4.800311 0.117772
C 3.117627 -2.848120 -0.356687
C 1.832731 3.433778 -0.407596

C -0.976366 4.770486 -0.398217
C 0.464949 -5.196951 -0.551133
C -1.667739 -2.432706 -1.373941
C 2.949792 2.787487 -1.240396
C -2.299932 5.112400 -1.094112
C -2.930645 1.084309 -1.403761
C 2.654038 -2.234579 -1.685721
C -2.631973 -1.317155 -1.761116
C 0.633419 -4.733315 -2.002423
C -1.741859 1.687039 -2.139247
C 3.744890 -2.420426 -2.751546
C 1.691650 -5.577308 -2.728264
C 0.261747 4.062956 -3.150201
C 0.918189 5.443382 -3.298182
C 0.725987 -2.480983 -4.121882
C 0.816671 -0.984884 -4.439483
C 0.904555 3.051121 -4.106404
C -0.512076 -3.089874 -4.792276
H -1.721677 -1.185119 6.433066
H -2.973469 -2.427014 6.335458
H -1.328508 -2.796837 5.824823
H -2.736530 1.222273 5.394277
H -0.323646 0.630834 5.559259
H -1.750377 2.683491 5.467330
H 0.882824 -2.134600 5.583660
H 1.651891 -0.603827 5.157641
H -3.262074 -0.843062 4.482305
H 0.585379 2.746054 4.570514
H 2.387131 -2.129227 4.664253
H -2.426862 2.160093 3.927195
H -3.899026 -3.182944 4.091296
H 1.320481 1.334544 3.802188
H -0.035567 -3.665068 3.645251
H -2.281494 -3.580585 3.514929
H 0.044875 2.231940 2.972096
H -3.298455 -2.489235 2.580897
H 1.265375 -1.036537 2.751256
H 1.468011 -3.481946 2.746724
H -3.485661 -0.036427 2.480054
H -0.072464 -3.056088 1.989042
H -2.917062 1.301828 1.512864
H -0.302574 4.225343 1.620985
H -1.816383 5.133130 1.588986
H -1.794582 3.430785 1.114841
H -3.386522 -1.562205 0.580737
H 2.368890 -2.755549 0.435947
H 1.596484 5.243890 0.828494
H 1.676310 2.791705 0.474455
H 3.264381 4.703004 0.632774
H 4.035151 -2.364362 -0.000308
H -4.447591 -0.189757 0.217878
H 1.394718 -5.097565 0.017121
H -0.301638 -4.622473 -0.022745
H 3.339014 -3.914440 -0.475161
H -2.002752 -2.894594 -0.430953
H -0.368290 5.688251 -0.390444
H -3.221872 1.770258 -0.601931
H 3.843528 2.614043 -0.628891
H 0.173779 -6.255144 -0.510435
H 2.441951 5.519741 -0.696079
H -2.823064 5.920503 -0.565271
H 2.555760 -1.147344 -1.522168

H -2.983180 4.254961 -1.109119
H 2.655077 1.823745 -1.669103
H -3.666788 -1.686138 -1.857358
H -1.772199 -3.225362 -2.128877
H -3.801339 0.962498 -2.069672
H 3.249235 3.436802 -2.070780
H 4.706135 -2.036466 -2.386960
H -2.158828 5.441560 -2.128871
H 2.678204 -5.474324 -2.262200
H -2.327737 -0.926679 -2.736923
H -0.324916 -4.924076 -2.510465
H -2.084092 2.639837 -2.567677
H 3.894270 -3.478315 -2.994846
H -1.496484 1.060510 -3.012117
H 0.442046 6.205532 -2.671780
H 1.433881 -6.644131 -2.693766
H 1.980411 5.413372 -3.030590
H 3.518977 -1.897453 -3.685299
H -0.786737 4.177073 -3.466508
H -0.056468 -0.449495 -4.048844
H 1.705783 -0.513069 -4.007016
H 1.796953 -5.302881 -3.783700
H 0.463502 2.055095 -4.008959
H 1.979079 2.949376 -3.925392
H -1.439443 -2.649988 -4.406620
H 1.606645 -2.969204 -4.566631
H 0.861785 5.794756 -4.337029
H -0.576570 -4.173604 -4.651145
H 0.846895 -0.805154 -5.522260
H 0.784019 3.368618 -5.150770
H -0.502545 -2.904640 -5.874793
Energy: −1733.254104 au

Supplementary Table 19 - Thorium azide with 2K added
Th 5.075400 -1.350444 0.875196
K 6.023593 2.475765 -0.883674
K 5.944280 0.276018 4.202961
Si 1.759141 0.000000 2.346892
Si 5.268212 -2.162931 -2.909608
Si 8.159209 -2.502473 2.954271
N 2.787453 -1.259613 1.652333
N 5.803637 0.620928 1.179189
N 6.365128 1.707091 2.037536
N 6.673545 2.812200 1.641332
N 4.143811 -3.863911 1.284492
N 5.254280 -2.633742 -1.218252
N 6.413476 -2.432798 2.643438
C 0.000000 0.000000 0.000000
C 0.000000 0.000000 1.533719
C 1.430856 -0.313670 4.243441
C 0.577638 -1.546600 4.564502
C -0.916151 1.109037 2.070611
C 2.631955 1.712497 2.207596
C 2.543757 2.314875 0.798964
C 2.195488 2.760590 3.241567
C 2.061420 -2.523746 1.435619
C 2.832260 -3.738985 1.944357
C 4.019394 -4.486008 -0.048889
C 5.148131 -4.085234 -0.999267
C 3.492276 -2.110644 -3.681003
C 2.548533 -1.256375 -2.828115
C 5.131843 -4.546456 2.135777

C 5.715231 -3.611427 3.198093
C 8.937236 -4.097045 2.171413
C 8.784232 -4.190095 0.647682
C 8.635578 -2.654840 4.846984
C 8.139792 -1.516017 5.744405
C 8.976969 -0.868292 2.329619
C 9.129239 -0.791870 0.804645
C 8.343057 -3.993571 5.540913
C 5.943730 -0.352734 -2.979865
C 5.480199 0.490734 -4.173762
C 6.301113 -3.371883 -4.013979
C 6.395869 -2.905022 -5.473024
C 7.467587 -0.273905 -2.835651
C 2.735071 -0.367790 5.044714
C 7.686588 -3.732568 -3.464250
C 2.874637 -3.493670 -3.924209
C 10.325374 -0.529347 2.984540
C 10.397272 -4.366724 2.562287
H 1.869830 -3.407974 -4.360237
H -1.016237 -0.146738 -0.390836
H 1.547320 -1.187472 -3.274282
H 3.473460 -4.104045 -4.608174
H -1.933022 1.008932 1.667244
H 3.599408 -1.621449 -4.661381
H 2.766278 -4.056982 -2.989512
H 0.364651 0.948974 -0.405797
H 0.630988 -0.792200 -0.411907
H 5.412246 -2.711769 -5.914906
H 2.912201 -0.230954 -2.691330
H 2.432169 -1.688953 -1.827481
H -0.445654 -0.956640 1.846823
H 4.391094 0.554948 -4.251368
H -0.556609 2.103133 1.781061
H 6.892727 -3.657923 -6.099763
H -0.999438 1.096355 3.162928
H 1.823003 -2.677991 0.368421
H 5.851243 0.083175 -5.120237
H 1.082337 -2.554354 1.937282
H 5.710191 -4.301046 -4.017614
H 3.073340 -4.149557 -0.484490
H 6.981975 -1.982787 -5.560208
H -0.385530 -1.532979 4.044742
H 5.866530 1.521101 -4.112107
H 1.527864 2.652018 0.562447
H 2.245894 -4.665448 1.812104
H 4.958892 -4.649116 -1.926961
H 3.963558 -5.585088 0.048388
H 2.845823 1.595611 0.030086
H 1.090287 -2.474575 4.286003
H 5.500023 0.081771 -2.063741
H 0.364245 -1.612540 5.640602
H 1.139847 3.032945 3.131400
H 8.137657 -4.549138 -4.044381
H 3.202307 3.191576 0.720229
H 3.002350 -3.609086 3.017906
H 0.872554 0.569388 4.588151
H 6.091723 -4.509914 -0.617025
H 7.965379 -0.624004 -3.746305
H 7.645019 -4.050754 -2.418659
H 8.377644 -2.885373 -3.516975
H 4.684958 -5.441896 2.6040060
H 7.815284 0.759504 -2.6767150

H 2.780016 3.682361 3.121068
H 3.699642 1.496937 2.362655
H 2.332422 2.426376 4.275662
H 7.837179 -0.875436 -2.001323
H 5.951207 -4.886650 1.495749
H 3.368227 -1.192616 4.688347
H 2.553700 -0.531658 6.116197
H 3.287339 0.575969 4.952015
H 4.900570 -3.304215 3.877261
H 8.211102 -1.065550 0.275221
H 6.364916 -4.242045 3.822701
H 9.376457 0.232021 0.498115
H 8.270239 -0.066668 2.590862
H 7.809631 -3.831814 0.301892
H 7.054937 -1.594122 5.906998
H 9.543088 -3.591636 0.134717
H 9.930611 -1.446469 0.445030
H 8.380394 -0.525159 5.337970
H 8.905861 -5.223962 0.298136
H 7.274934 -4.111563 5.757165
H 10.707903 0.422992 2.594980
H 8.595558 -1.561507 6.742639
H 8.329480 -4.908174 2.604712
H 10.262518 -0.427633 4.072685
H 11.086146 -1.289904 2.773217
H 8.867511 -4.058372 6.504340
H 9.730981 -2.557992 4.799862
H 8.653875 -4.857446 4.945670
H 11.061489 -3.584593 2.177213
H 10.747781 -5.317596 2.138325
H 10.547954 -4.421228 3.644686
Energy: −1789.954035 au

Supplementary Table 20 - Thorium nitride complex capped by 2 K^+
Th 3.455259 -1.093599 5.548275
K 5.559812 -3.359115 3.042236
K 7.203894 0.377922 4.675374
Si 4.502678 1.708551 8.090894
Si 3.460621 -4.831477 6.671879
Si 1.994135 0.108882 2.200106
N 3.437639 0.463543 7.500546
N 2.658823 -3.343413 6.231758
N 0.895485 -0.952584 6.649182
N 5.242824 -1.301168 4.608795
N 1.851577 -0.201153 3.914512
C 2.834419 -4.570070 9.533519
C 3.482761 -6.874907 8.753046
C 2.812428 -5.552577 8.355546
C 1.713099 -6.799851 5.379818
C 3.592652 -5.936715 3.975830
C 3.159960 -6.287745 5.402934
C 4.117794 3.626403 5.949306
C 6.505494 3.377730 6.678687
C 5.156215 2.661451 6.534941
C 2.302442 3.494511 8.937449
C 4.580335 4.026045 9.859835
C 3.653428 2.906030 9.364745
C 2.689792 -2.591711 1.419975
C 3.897779 -0.800761 0.139722
C 3.256114 -1.168832 1.482103
C 1.773676 3.007018 2.178118
C 4.094517 2.073531 2.393517

C 2.709330 1.861286 1.772174
C 5.574707 0.219673 10.297646
C 6.946017 0.110577 8.204172
C 6.035870 1.001173 9.058881
C 2.369972 0.015174 8.403724
C 0.990405 0.035483 7.735240
C 5.824257 -3.756591 7.972214
C 6.272126 -5.689375 6.445299
C 5.360807 -4.481798 6.702265
C 1.190062 -3.401669 6.285810
C 0.573509 -2.297371 7.147513
C 0.003801 -0.528311 5.560829
C 0.697589 0.419055 4.579399
C -0.585488 -1.187804 1.654030
C 0.270839 0.035947 1.306363
C 0.361576 0.212153 -0.215584
H 7.327719 -5.382484 6.400309
H 6.197468 -6.430960 7.249020
H 6.043636 -6.216387 5.511784
H 4.548433 -6.733751 8.970065
H 6.853697 -3.389307 7.860855
H 3.406020 -7.638313 7.971481
H 5.812592 -4.421977 8.843119
H 7.690546 -0.412806 8.820756
H 3.028128 -7.294563 9.661262
H 3.795827 -7.119190 5.743493
H 6.429440 -0.197701 10.847976
H 5.495577 -3.751551 5.885541
H 4.681018 -5.800921 3.914691
H 6.378511 -0.652484 7.657481
H 7.514084 0.701897 7.473327
H 5.193944 -2.893519 8.208773
H 3.851519 -4.404840 9.902868
H 2.246036 -4.953601 10.378384
H 3.337702 -6.734141 3.263171
H 4.928622 -0.620061 10.016725
H 6.636195 1.856305 9.404190
H 1.752891 -5.778467 8.157196
H 5.013354 0.845306 10.999303
H 1.610478 -7.670273 4.716296
H 7.300827 2.734422 7.072303
H 1.365230 -7.106609 6.371278
H 3.094730 -5.020544 3.633119
H 2.429200 -3.591104 9.260937
H 1.021802 -6.033572 5.011679
H 5.258889 1.843202 5.800468
H 6.846525 3.796203 5.718360
H 2.551633 -1.009478 8.778770
H 5.531236 3.646736 10.248394
H 6.428776 4.224744 7.368431
H 0.806229 -4.349268 6.699145
H 4.038627 -1.135226 2.262982
H 4.668551 -1.534662 -0.148526
H 3.455713 -3.334056 1.127372
H 0.981863 -2.388994 8.158139
H 2.287037 0.626650 9.315886
H 3.454204 2.260194 10.234092
H 4.816779 1.317724 2.063618
H 0.746288 -3.341105 5.275715
H 4.811440 4.740951 9.060914
H 4.037825 2.007066 3.485980
H 2.251975 -2.906769 2.373570

H 4.381032 0.180448 0.158383
H 4.106215 4.600279 10.667690
H 4.421065 3.992915 4.959477
H 4.500257 3.064293 2.139597
H 3.139945 3.147757 5.841535
H -0.521452 -2.429714 7.227265
H 3.992556 4.506299 6.589619
H 3.166285 -0.786769 -0.675673
H 0.187357 -0.127474 8.477408
H 1.906874 -2.680471 0.659107
H 0.842120 1.030178 7.303367
H 1.605447 2.728084 8.588646
H 2.412962 4.222306 8.127871
H 1.823669 4.019344 9.775820
H -0.298092 -1.423872 5.007941
H 0.979716 1.336269 5.128448
H 2.821624 1.896229 0.677501
H 1.585227 3.004641 3.257833
H -0.915284 -0.067188 5.967037
H -0.083042 0.750247 3.875118
H 2.209886 3.985170 1.930363
H -0.173251 -2.106717 1.223629
H -0.658659 -1.344659 2.733851
H 0.850067 -0.646846 -0.691106
H 0.802751 2.950819 1.674794
H 0.923040 1.108135 -0.501591
H -0.266414 0.916749 1.691189
H -1.606039 -1.081719 1.260689
H -0.637165 0.296560 -0.666233
Energy: -1680.4737981 au

Supplementary Table 21 - Thorium nitride dianion complex
Th -0.789170 -3.226008 5.651107
Si -3.806861 -3.789208 7.893726
Si 2.232336 -4.726986 6.894568
Si -1.048937 -1.452890 2.394669
N -2.075930 -4.295845 4.696116
N -2.235268 -3.015999 7.521270
N 1.381026 -3.231680 6.466998
N -0.273530 -0.904389 6.929346
N -0.908572 -1.382617 4.175096
C 4.183262 -4.128662 4.796207
C 3.258943 -4.062472 9.545947
C -5.272753 -4.722551 10.218826
C -5.421162 -5.982634 6.825601
C -5.290373 -2.374038 5.865899
C -3.068031 -6.565034 7.506665
C -3.984381 -5.461908 6.964012
C -5.389114 -1.346689 8.158401
C -5.288489 -2.658422 7.371070
C -0.520971 -3.508711 0.388384
C -3.645996 -0.169796 2.205104
C 1.241279 -6.222437 4.595426
C 0.952590 -5.065842 9.443359
C 2.339898 -5.035523 8.794776
C 1.425847 -7.522807 6.736313
C 1.160815 -6.137378 6.126610
C -3.665670 -2.682262 2.090084
C -2.869372 -1.415525 1.756776
C -1.385228 -0.703091 7.883879
C -1.827088 -2.012445 8.516380
C -2.775180 -4.675736 10.510244

C -3.971928 -4.012017 9.819004
C 4.822656 -5.936090 6.446044
C 4.042144 -4.630785 6.237089
C 1.972883 -1.934523 6.764746
C 1.024250 -1.077887 7.607847
C -0.226740 0.176787 5.929315
C -1.205634 -0.095636 4.791529
C 1.274473 -3.166622 2.111059
C -0.235274 -3.102812 1.842329
C 1.192549 0.442743 2.221290
C -0.193352 0.121840 1.648870
C -0.142228 0.109636 0.114370
H -1.118592 -0.087411 -0.339978
H 0.206936 1.076619 -0.271904
H 0.554299 -0.650469 -0.256336
H -3.144507 0.766472 1.936609
H -2.763950 -1.361282 0.661390
H -4.642022 -0.146624 1.743617
H -0.034784 -2.834390 -0.324810
H -1.589240 -3.519147 0.153951
H -0.861044 0.950109 1.932282
H -4.683220 -2.619231 1.680737
H -0.134319 -4.516612 0.188030
H -3.800002 -0.167437 3.289984
H 1.551897 1.415491 1.858968
H -3.204386 -3.587063 1.682429
H 1.826280 -2.499426 1.439445
H 1.934641 -0.304124 1.921544
H -3.743786 -2.832287 3.170634
H 1.194093 0.476108 3.314632
H -0.732731 -3.847615 2.485492
H 1.663719 -4.178792 1.939338
H -1.138984 0.747175 4.087536
H 1.538554 -2.878756 3.135865
H -2.235767 -0.051999 5.185019
H -5.281444 -3.289924 5.267352
H -6.172778 -1.790169 5.570269
H -4.404044 -1.798419 5.575535
H 0.783476 0.211101 5.511934
H 1.096744 -5.258349 4.094987
H -6.085322 -5.255638 6.348379
H -3.618531 -5.211039 5.958563
H -5.439286 -6.889644 6.206118
H 0.483660 -6.908823 4.200056
H -0.425138 1.148380 6.410388
H 3.795650 -4.852920 4.072601
H 3.646807 -3.189030 4.636065
H 2.221643 -6.595243 4.279110
H 2.237805 -1.390654 5.843099
H -6.189245 -3.248040 7.600993
H -2.229555 -0.287138 7.325211
H -3.037789 -7.422321 6.820982
H 5.238453 -3.957647 4.545278
H -5.866481 -6.249759 7.790394
H -6.275627 -0.771497 7.857817
H 0.113037 -5.895172 6.390737
H -4.520247 -0.703282 7.974247
H -2.037940 -6.216673 7.642819
H 1.468666 -0.096318 7.838125
H 2.912044 -2.026094 7.329333
H -1.094000 0.037189 8.648543
H -3.415273 -6.942633 8.475668

H 0.747855 -8.269130 6.304464
H -5.458886 -1.507552 9.239460
H 4.441295 -6.737177 5.802625
H 0.847283 -1.595913 8.554412
H 4.505316 -3.871055 6.886735
H -2.631542 -1.746493 9.217497
H 2.448268 -7.858944 6.531810
H -1.012281 -2.389277 9.159932
H -6.161936 -4.256444 9.781358
H 5.883023 -5.803656 6.194437
H -5.264918 -5.773155 9.908364
H 1.283510 -7.539580 7.820300
H -4.041101 -2.987404 10.214890
H 0.439575 -4.105638 9.317609
H 0.301155 -5.833182 9.013132
H 4.776318 -6.293323 7.480497
H -1.831150 -4.176425 10.277077
H -2.668083 -5.724280 10.215127
H 2.881166 -3.033895 9.503359
H 2.780176 -6.040200 8.892137
H -5.403407 -4.713608 11.309648
H 4.280403 -4.057020 9.151582
H 1.018787 -5.262865 10.521178
H -2.897209 -4.660749 11.602216
H 3.323283 -4.329332 10.609010
Energy: −1623.6454727 au

Supplementary Table 22 - Thorium imido dimer LThNH(2K)HNThL
Th -3.733525 0.743258 1.584146
Th 3.727437 -0.729716 -1.610061
K 0.597528 1.673938 0.591931
K -0.561645 -1.637288 -0.544896
Si -1.961384 -0.946640 4.558995
Si -3.782975 4.533802 1.198951
Si 5.989081 1.065116 0.887917
Si -5.832496 -1.435710 -0.770745
Si 3.607454 -4.532730 -1.505387
Si 1.985188 1.294288 -4.374588
N -3.200324 0.076170 3.850602
N -5.965202 0.975275 3.203071
N -4.547977 3.007092 1.627963
N -5.506293 -0.673575 0.785536
N -2.057343 0.645900 0.241229
N 2.085621 -0.624637 -0.226351
N 5.586433 0.503237 -0.734619
N 4.437761 -3.020272 -1.848640
N 5.910108 -0.925157 -3.303061
N 3.193940 0.159601 -3.800675
C -1.425726 -2.681665 6.820956
C -3.862290 -2.467616 6.245545
C -2.503698 -1.756528 6.235675
C -0.568531 1.072989 6.102814
C -4.075204 0.788427 4.798006
C -0.345285 0.035759 4.994722
C -5.552605 0.497279 4.534389
C -4.529395 5.786055 3.763306
C 0.252839 0.715742 3.760753
C 4.923998 0.433818 3.548055
C -1.441263 4.186129 2.884318
C -6.346504 2.397304 3.219534
C -1.492259 -2.255059 3.217100
C -0.111002 -2.901738 3.383363

C -2.559943 -3.343646 3.048795
C -7.004221 0.123509 2.600843
C 8.264517 1.703363 2.573381
C 5.934999 -1.471712 2.270203
C -4.459541 6.017066 2.249420
C 5.173009 -0.144861 2.147554
C -5.992152 3.079559 1.900938
C -3.731284 7.335923 1.950982
C -6.389618 -1.082089 1.891032
C -1.878747 4.275695 1.417649
C 3.336891 -4.069638 1.319544
C -0.993001 5.276318 0.663771
C 7.903481 1.245112 1.151976
C 5.290823 2.839589 1.249211
C 3.764866 2.895596 1.123208
C 5.295647 -5.488453 0.637483
C 8.759090 0.034577 0.760501
C 3.840207 -5.118050 0.323178
C 5.927097 3.928890 0.377129
C -5.649299 -4.193470 0.129260
C -4.075307 5.037696 -0.644215
C -3.529573 -3.173420 -0.749068
C 0.772303 -5.150140 -1.037360
C -5.053610 -3.212880 -0.887821
C -5.547915 5.336948 -0.954929
C -8.633562 -0.503160 -0.836600
C -7.728665 -1.718661 -1.071379
C -3.539455 3.978973 -1.613011
C 1.722042 -4.185006 -1.759397
C 6.464002 0.975778 -1.818163
C -5.013824 -0.349952 -2.135528
C 5.867118 -3.131910 -2.178752
C -5.788783 0.946526 -2.407640
C 3.473141 -7.301201 -2.359075
C 4.248976 -5.997779 -2.601181
C 7.004447 -0.184490 -2.653680
C 0.235448 3.137752 -2.896423
C 1.611816 2.462075 -2.883068
C 2.708023 3.511531 -2.656794
C -8.026370 -2.334887 -2.446444
C 1.320772 -4.054746 -3.233605
C -0.263898 -0.392603 -3.727554
C -4.729438 -1.066945 -3.463139
C 6.210633 -2.359139 -3.450203
C 4.351198 -5.716619 -4.105056
C 0.318069 0.438515 -4.874637
C 5.502317 -0.310860 -4.578694
C 4.007712 -0.498888 -4.835399
C 0.448140 -0.432030 -6.130925
C 2.525723 2.268645 -5.962165
C 3.919818 2.906495 -5.932892
C 1.480575 3.309537 -6.393031
H -1.694031 -3.000260 7.837051
H -4.126652 -2.784362 7.263641
H -2.589505 -0.902341 6.925201
H -0.897518 0.614280 7.040643
H -0.442515 -2.203798 6.879562
H -1.314622 -3.593763 6.223468
H -4.670000 -1.826995 5.883457
H 0.354242 1.627949 6.320861
H -3.877550 0.523148 5.847379
H -3.858330 -3.368056 5.623155

H -1.326882 1.809294 5.811318
H -6.196039 0.932839 5.318715
H 0.388235 -0.697670 5.360910
H -3.921386 1.881017 4.753905
H -5.692195 -0.587074 4.560383
H -3.532037 5.717507 4.209563
H 4.395882 -0.292902 4.180209
H -5.047101 6.615910 4.263398
H -5.779979 2.886364 4.017194
H -5.060047 4.865163 4.018311
H 5.862739 0.673472 4.059768
H -0.054934 -3.491417 4.304379
H -2.580183 -4.006864 3.920168
H 1.154085 1.291341 4.017679
H -1.511697 5.161187 3.379050
H -2.050982 3.482464 3.460712
H -0.390857 3.871332 2.981478
H 4.323854 1.349054 3.533011
H -7.418206 2.510437 3.458822
H 0.703403 -2.172217 3.420622
H -0.479312 1.408538 3.327586
H -7.740719 -0.189638 3.361419
H 8.047530 0.924426 3.313198
H -3.564636 -2.926117 2.928003
H 5.366520 -2.203400 2.858007
H 0.532150 -0.016230 2.993151
H 7.725660 2.606621 2.878157
H 6.893025 -1.329333 2.782394
H 3.489737 -4.395852 2.357516
H -5.853463 -1.691125 2.640386
H 9.337668 1.924167 2.651864
H -1.478365 -1.651879 2.290129
H -2.698224 7.312158 2.316782
H -4.224979 8.179524 2.451909
H 0.106462 -3.596168 2.559217
H -2.355054 -3.979155 2.176436
H 5.543333 3.057881 2.297351
H -6.368407 4.112112 1.965437
H -5.494548 6.144851 1.896976
H -7.536002 0.721372 1.855076
H 5.407209 -5.806250 1.683075
H 4.187748 -0.363273 1.701248
H 3.283239 2.181086 1.801576
H 3.868994 -3.121465 1.191742
H -1.092102 6.288139 1.070231
H 2.270085 -3.854554 1.198898
H 6.147498 -1.923079 1.295249
H -1.743925 3.280437 0.956306
H -7.223617 -1.719725 1.561885
H 0.074730 5.018440 0.752232
H -6.585448 2.615181 1.093735
H 8.583538 -0.818909 1.422224
H 1.912376 -0.913104 0.743413
H -3.697529 7.568156 0.881430
H 9.828688 0.276688 0.825621
H -5.487567 -3.848345 1.156817
H 3.381521 3.900164 1.357644
H 5.964033 -4.632164 0.490380
H 3.229623 -6.025430 0.448237
H 8.189494 2.067487 0.478086
H -3.249171 -2.728679 0.214010
H 5.664224 -6.306995 0.010064

H 5.512621 4.919787 0.609519
H -1.229426 5.332126 -0.402725
H 0.970218 -5.216958 0.036622
H 7.010846 3.992769 0.518119
H 3.461349 2.650658 0.097642
H -8.488587 -0.060728 0.152280
H 8.561460 -0.301997 -0.260207
H -5.187286 -5.186921 0.045160
H -6.727261 -4.327172 -0.007243
H -1.863471 0.940473 -0.722805
H -5.949017 6.152639 -0.343953
H -3.094133 -4.182698 -0.794009
H -3.505796 5.965448 -0.807868
H -8.011570 -2.475101 -0.323042
H -6.177683 4.455830 -0.785432
H 5.743876 3.745586 -0.688147
H 1.628209 -3.189562 -1.287932
H -0.281549 -4.854364 -1.165495
H -2.463601 3.812693 -1.498896
H -3.079937 -2.575675 -1.551344
H 6.508989 -2.764201 -1.358909
H -6.034642 1.486282 -1.486919
H -4.032219 3.015021 -1.450204
H 3.415224 -7.567281 -1.298533
H 0.848147 -6.164895 -1.441683
H -4.042225 -0.069271 -1.693677
H -8.453281 0.285595 -1.573361
H 7.332454 1.543331 -1.452104
H -9.692541 -0.783834 -0.918903
H 1.641458 1.756874 -2.032103
H 0.079500 3.748322 -1.994691
H -5.281877 -3.591520 -1.895189
H -5.679033 5.625919 -2.006629
H 2.568448 4.045672 -1.706710
H 7.523495 -0.874671 -1.982446
H 6.191096 -4.171115 -2.341656
H 5.273420 -6.172701 -2.237833
H -0.447875 0.220348 -2.837004
H -3.717409 4.266161 -2.658574
H 5.938344 1.674816 -2.492422
H 2.446939 -7.233352 -2.738752
H -6.730275 0.741254 -2.929728
H 0.431902 -1.194635 -3.452665
H -5.212796 1.626371 -3.048211
H 3.709575 3.070064 -2.645399
H -0.592376 2.425348 -2.960631
H 3.947572 -8.143228 -2.880803
H -7.438590 -3.237533 -2.642303
H 0.293101 -3.678600 -3.347269
H -9.085874 -2.612180 -2.529218
H -4.136655 -1.979032 -3.339839
H -1.217645 -0.857816 -4.014556
H 1.982868 -3.381287 -3.787516
H 7.737239 0.163973 -3.402275
H -7.821133 -1.625702 -3.256346
H 1.347660 -5.027810 -3.736166
H 0.133344 3.822078 -3.744957
H 7.265218 -2.508267 -3.741117
H 2.690259 4.271412 -3.445374
H -5.655174 -1.354439 -3.973173
H -4.180840 -0.409656 -4.151472
H 5.588441 -2.749480 -4.261300

H 4.919958 -4.808190 -4.320604
H 3.364444 -5.596241 -4.563366
H 4.846455 -6.547547 -4.625681
H 5.698544 0.763108 -4.510682
H 3.795091 -1.580815 -4.895999
H -0.396427 1.245282 -5.096037
H 1.190305 -1.226941 -5.990352
H 6.109093 -0.706938 -5.411785
H 3.805545 -0.118122 -5.848189
H -0.504745 -0.920587 -6.376756
H 3.987052 3.709774 -5.192983
H 4.703256 2.181761 -5.697926
H 1.431405 4.146953 -5.687094
H 0.747933 0.147644 -7.009585
H 0.472875 2.889516 -6.476459
H 2.545952 1.501851 -6.752060
H 4.164911 3.347901 -6.908524
H 1.735942 3.737361 -7.371686
Energy: −3305.5761024 au

Supplementary Table 23 - Thorium terminal imido complex with toluene adduct
Th 5.745838 1.879422 -2.670731
Si 4.793711 0.001240 -5.882770
Si 4.407283 5.497705 -2.427829
Si 8.207837 0.538564 -0.035162
N 5.737216 4.421561 -2.710673
N 7.969289 1.280877 -1.582903
N 8.011774 2.949648 -4.073548
N 5.776593 1.220051 -5.130681
N 4.232726 1.052293 -1.752039
C 6.857270 -1.769118 -6.987518
C 3.845338 1.836623 -7.986792
C 1.580390 5.064254 -1.749786
C 3.856208 8.086512 -1.161482
C 3.235557 5.054081 -5.014977
C 5.823525 6.814765 -0.222169
C 4.989278 7.103082 -1.474910
C 4.530565 7.189208 -4.831259
C 3.629642 6.202284 -4.080496
C 9.788937 2.838607 0.874305
C 6.388330 2.142563 1.547252
C 2.143199 0.823618 -5.068921
C 3.531535 -0.618300 -8.448227
C 4.407501 0.430312 -7.752566
C 2.552754 -1.652596 -4.935705
C 3.198854 -0.265205 -4.843215
C 6.186769 -2.154277 -4.605539
C 5.692792 -1.731059 -5.991372
C 7.554056 -2.120025 -0.908342
C 6.364309 -0.309475 2.087511
C 6.587524 0.730201 0.982815
C 10.014334 -1.703834 -0.635313
C 8.617322 -1.370533 -0.098853
C 7.702837 4.367709 -4.266741
C 7.021226 5.015237 -3.052147
C 3.257620 4.421113 0.004571
C 3.006459 4.555716 -1.502722
C 9.992472 0.759968 2.286581
C 9.764387 1.306925 0.872767
C 9.129396 1.401257 -2.450251
C 9.176437 2.730366 -3.216846
C 8.080841 2.196484 -5.325135

C 6.712306 1.946373 -5.972918
C 2.127137 -0.482886 -0.401538
C -0.230997 0.399877 -0.595796
C 0.932294 0.172641 0.170578
C 0.912678 0.659013 1.495560
C -0.195015 1.316163 2.022162
C -1.339615 1.519371 1.246251
C -1.340253 1.056726 -0.072843
H 1.795895 0.512659 2.114351
H -0.166488 1.673938 3.050867
H -2.206525 2.033149 1.656487
H -2.217439 1.209479 -0.700758
H -0.249497 0.049983 -1.626130
H 1.855410 -1.203134 -1.181782
H 2.707198 -1.004391 0.368395
H 2.890473 0.249453 -0.914525
H 10.029930 -0.335530 2.306181
H 10.935861 1.128272 2.722494
H 9.188234 1.067684 2.966255
H 10.813898 -1.267406 -0.024970
H 8.568800 -1.730492 0.941780
H 10.185400 -2.792118 -0.666349
H 7.124199 -0.248802 2.879251
H 6.373395 -1.334751 1.703664
H 10.619784 0.977071 0.260937
H 7.720570 -3.209048 -0.891433
H 5.387452 -0.155746 2.568569
H 10.145877 -1.333318 -1.659154
H 10.756789 3.230345 1.227408
H 6.538826 -1.928214 -0.546559
H 7.073959 2.354970 2.379971
H 9.015094 3.250183 1.531053
H 7.570425 -1.800568 -1.955341
H 9.602014 3.242584 -0.125190
H 5.797077 0.590756 0.218505
H 5.365014 2.266550 1.926410
H 10.098233 1.349686 -1.914034
H 6.543795 2.912360 0.784244
H 9.189289 0.578802 -3.192299
H 5.383373 -2.174010 -3.861903
H 6.656239 -3.150574 -4.621191
H 6.934463 -1.442010 -4.238054
H 9.199843 3.536074 -2.475943
H 4.263210 4.044994 0.210556
H 3.248248 -2.456875 -4.673107
H 3.551498 -0.113585 -3.802975
H 1.701535 -1.726089 -4.243253
H 2.553730 3.704605 0.446221
H 10.116055 2.801218 -3.806637
H 5.216235 6.365429 0.570330
H 6.638324 6.116435 -0.435044
H 3.131512 5.379107 0.529616
H 7.735299 4.974159 -2.205341
H 4.938953 -2.461713 -6.326292
H 8.528567 1.221546 -5.100749
H 1.357010 0.761665 -4.304538
H 6.265746 7.736625 0.189831
H 2.165454 -1.871620 -5.941232
H 7.359025 -2.750337 -6.988630
H 3.078207 3.525646 -1.895592
H 7.620004 -1.024081 -6.727578
H 2.580158 1.824077 -4.994743

H 8.619906 4.934192 -4.538972
H 6.967091 6.093045 -3.300757
H 8.753255 2.703668 -6.051715
H 1.656850 0.734494 -6.051091
H 0.851020 4.416561 -1.244379
H 6.538361 -1.566059 -8.016787
H 3.144458 7.658087 -0.445487
H 7.009539 4.447232 -5.110467
H 5.659299 7.603765 -2.192471
H 6.949077 1.440214 -6.929428
H 1.431479 6.081965 -1.361926
H 6.311332 2.931933 -6.286164
H 3.933206 -1.632286 -8.339668
H 4.235326 9.020846 -0.715752
H 2.517279 -0.626006 -8.031127
H 1.316635 5.081120 -2.812504
H 5.394755 0.398957 -8.241091
H 4.116384 4.463519 -5.292135
H 2.529804 4.360609 -4.545786
H 3.287401 8.359769 -2.058081
H 4.463357 2.604756 -7.513792
H 2.835796 1.940660 -7.575903
H 5.476768 6.715361 -5.120079
H 2.711996 6.739849 -3.791664
H 3.431359 -0.417115 -9.527539
H 4.776922 8.075739 -4.234849
H 2.772151 5.421054 -5.944458
H 3.782762 2.070517 -9.062368
H 4.056596 7.543147 -5.761255
Energy: −1895.1717786 au

Supplementary Table 24 - TS of the Hydrogen transfer from the toluene to the terminal imido thorium complex
Th 5.745019 1.874722 -2.672064
Si 4.794664 -0.001996 -5.882553
Si 4.392105 5.482958 -2.419903
Si 8.200865 0.534389 -0.035445
N 5.726280 4.410615 -2.705467
N 7.961849 1.277476 -1.584014
N 8.004899 2.950203 -4.068419
N 5.776141 1.217437 -5.126999
N 4.229790 1.037322 -1.750739
C 6.863375 -1.764509 -6.989352
C 3.842015 1.837511 -7.980937
C 1.566102 5.040258 -1.747551
C 3.833294 8.063425 -1.143045
C 3.226270 5.045835 -5.010935
C 5.803479 6.793456 -0.207302
C 4.969838 7.084973 -1.459723
C 4.517033 7.182829 -4.818170
C 3.617348 6.191358 -4.071926
C 9.773434 2.839214 0.877284
C 6.374982 2.131214 1.546712
C 2.144593 0.816496 -5.063633
C 3.536187 -0.616884 -8.450112
C 4.408622 0.432266 -7.750725
C 2.555347 -1.660157 -4.941033
C 3.201019 -0.272817 -4.842970
C 6.190680 -2.157881 -4.609181
C 5.697753 -1.731416 -5.994372
C 7.557834 -2.124376 -0.915504
C 6.356323 -0.321958 2.082305
C 6.578456 0.720332 0.979731

C 10.016215 -1.701000 -0.635454
C 8.616726 -1.372676 -0.102348
C 7.692857 4.368141 -4.260093
C 7.008143 5.010887 -3.045020
C 3.242813 4.393896 0.006300
C 2.993024 4.533327 -1.501036
C 9.980575 0.759992 2.288151
C 9.753408 1.307455 0.874323
C 9.124706 1.402285 -2.447680
C 9.169989 2.732858 -3.211324
C 8.077781 2.199377 -5.321524
C 6.710328 1.946062 -5.969694
C 2.189185 -0.443182 -0.447582
C -0.155301 0.469584 -0.633735
C 1.012571 0.223179 0.126062
C 1.003029 0.718073 1.451776
C -0.090857 1.395251 1.979813
C -1.237567 1.615745 1.210131
C -1.249801 1.146708 -0.107655
H 1.885294 0.558753 2.069110
H -0.050897 1.755397 3.007730
H -2.093840 2.145506 1.622410
H -2.127715 1.310069 -0.732585
H -0.187282 0.114621 -1.662256
H 1.912978 -1.153108 -1.235869
H 2.774139 -0.969089 0.315820
H 2.994952 0.298018 -0.981453
H 10.021325 -0.335379 2.307018
H 10.922145 1.130829 2.725654
H 9.174388 1.064823 2.966745
H 10.812823 -1.262307 -0.022904
H 8.566447 -1.734118 0.937630
H 10.190763 -2.788663 -0.666472
H 7.115121 -0.260545 2.874988
H 6.368394 -1.346503 1.696691
H 10.610862 0.980483 0.263859
H 7.727715 -3.212802 -0.899439
H 5.378459 -0.171467 2.561906
H 10.149231 -1.329758 -1.658852
H 10.738958 3.233199 1.234056
H 6.541129 -1.936482 -0.555853
H 7.058341 2.342926 2.381401
H 8.996216 3.247938 1.531748
H 7.576142 -1.803762 -1.962132
H 9.589045 3.243856 -0.122415
H 5.789444 0.579888 0.214474
H 5.350722 2.251981 1.923948
H 10.091488 1.352100 -1.908081
H 6.530709 2.903524 0.786232
H 9.188832 0.581222 -3.190693
H 5.386129 -2.182521 -3.866980
H 6.662634 -3.152880 -4.627473
H 6.936227 -1.445152 -4.238229
H 9.190667 3.537361 -2.469059
H 4.249430 4.020529 0.212553
H 3.251350 -2.465392 -4.682802
H 3.553997 -0.125982 -3.802566
H 1.705054 -1.736814 -4.248142
H 2.540299 3.674307 0.444585
H 10.109504 2.807205 -3.800470
H 5.196793 6.337623 0.581866
H 6.621526 6.099625 -0.422484

H 3.113751 5.350166 0.533675
H 7.721639 4.971720 -2.197769
H 4.945545 -2.462367 -6.332183
H 8.528942 1.225713 -5.098539
H 1.361131 0.753408 -4.296841
H 6.241357 7.714989 0.209892
H 2.167516 -1.874286 -5.947350
H 7.366146 -2.745138 -6.993345
H 3.065848 3.505053 -1.897706
H 7.625064 -1.019611 -6.725979
H 2.581223 1.817170 -4.990326
H 8.608908 4.936939 -4.530123
H 6.948641 6.088641 -3.291681
H 8.748705 2.710361 -6.046493
H 1.655499 0.728089 -6.044434
H 0.838077 4.388526 -1.246059
H 6.545293 -1.558232 -8.018196
H 3.122654 7.629912 -0.429087
H 7.000513 4.447153 -5.104628
H 5.639061 7.591041 -2.174228
H 6.948102 1.439740 -6.925648
H 1.415399 6.055888 -1.355075
H 6.306756 2.930292 -6.283296
H 3.940674 -1.630033 -8.344431
H 4.209391 8.997103 -0.693571
H 2.521640 -0.628701 -8.033926
H 1.303628 5.061803 -2.810529
H 5.396063 0.405574 -8.239136
H 4.108343 4.457688 -5.289386
H 2.520771 4.349885 -4.545119
H 3.264048 8.338383 -2.038830
H 4.457140 2.606341 -7.505300
H 2.831837 1.936911 -7.570571
H 5.464012 6.711790 -5.109077
H 2.698307 6.726006 -3.782315
H 3.436434 -0.412516 -9.528782
H 4.761895 8.067163 -4.217945
H 2.763469 5.415417 -5.939618
H 3.779567 2.074298 -9.055814
H 4.042588 7.540024 -5.746629
Energy: -1895.171719 (Imag=-117 cm^{-1})

Supplementary Table 25 - Thorium terminal imido complex with benzene adduct
Th -4.034642 0.290351 3.982588
Si -6.551794 -0.085281 1.096202
Si -2.976212 3.882020 4.917184
Si -3.082610 -2.720062 6.171172
N -4.221324 2.675640 4.884382
N -4.082377 -1.323045 5.959073
N -6.325079 0.670920 5.723980
N -6.253584 -0.131355 2.799928
N -2.467156 0.061492 2.855357
C -4.569157 -1.950703 0.103996
C -7.866584 -2.716203 1.131197
C -1.585231 2.609032 7.077984
C -2.625063 6.778227 4.662134
C -0.209350 3.950884 3.918456
C -4.624297 5.836082 3.453373
C -3.698651 5.683481 4.665102
C -2.028739 3.815264 2.187465
C -1.625599 3.445388 3.619735
C -3.271699 -5.464270 7.197902

C -1.104979 -4.051264 4.448583
C -8.595761 2.006935 0.933137
C -8.248311 -1.331726 -0.940485
C -7.997971 -1.295463 0.571456
C -6.202523 2.757545 0.947931
C -7.171217 1.665977 0.483056
C -4.794080 0.154204 -1.251491
C -4.913974 -0.457529 0.148841
C -1.862222 -2.269707 8.800944
C -3.456111 -3.965767 3.577100
C -2.413123 -3.251285 4.445796
C -0.691764 -1.265904 6.821300
C -1.524895 -2.452744 7.316715
C -6.105342 1.962663 6.372930
C -5.518594 3.027638 5.435816
C -2.963657 4.584184 7.768571
C -2.087856 3.993001 6.658197
C -5.451552 -4.428031 6.475333
C -4.065499 -4.159286 7.069122
C -4.892027 -0.877888 7.079135
C -6.307932 -0.447228 6.666391
C -7.488423 0.657563 4.837382
C -7.372824 -0.366094 3.696844
C 1.313444 -0.099433 -0.967506
C 2.541414 -0.083065 -0.303243
C 2.566384 -0.047288 1.092488
C 1.370031 -0.028763 1.811793
C 0.132567 -0.045160 1.161181
C 0.123981 -0.080351 -0.236382
H -0.832946 -0.091671 -0.755404
H 1.287329 -0.126293 -2.056796
H 3.472162 -0.097067 -0.869044
H 3.521793 -0.033222 1.616690
H 1.387226 0.000077 2.899829
H -0.821934 -0.026283 1.750318
H -2.293263 -5.310237 7.667827
H -3.810127 -6.215508 7.799248
H -3.090028 -5.915704 6.214769
H -2.369929 -3.143257 9.227068
H -0.911691 -3.363952 7.224699
H -0.956257 -2.093363 9.403268
H -1.214091 -5.032344 4.932505
H -0.293545 -3.522822 4.959166
H -4.226388 -3.780270 8.091324
H 0.239967 -1.152338 7.398498
H -0.766793 -4.237246 3.418908
H -2.517742 -1.403551 8.953373
H -6.033780 -5.125699 7.099641
H -0.427847 -1.349541 5.762232
H -3.665145 -4.984172 3.935810
H -5.380446 -4.871210 5.476331
H -1.256603 -0.333451 6.920279
H -6.027896 -3.504369 6.370155
H -2.209633 -2.279763 3.952790
H -3.100925 -4.053848 2.541098
H -5.043927 -1.649653 7.860573
H -4.405944 -3.421777 3.547759
H -4.427899 -0.030665 7.625047
H -0.903871 2.171924 6.340344
H -1.058701 2.635525 8.045265
H -2.427358 1.914081 7.175695
H -6.781289 -1.303187 6.174070

H -4.622352 -2.402449 1.098520
H 0.157122 3.619745 4.896216
H -1.608869 2.338645 3.627025
H 0.497604 3.574616 3.165282
H -3.541529 -2.098200 -0.255465
H -6.915972 -0.210531 7.566822
H -7.015165 -3.244875 0.690672
H -7.713496 -2.710423 2.214126
H -5.236375 -2.510520 -0.567190
H -7.321453 -1.372281 4.159525
H -1.213497 4.651823 6.533714
H -5.387089 1.806645 7.185980
H -1.338221 3.359236 1.466089
H -8.766577 -3.317209 0.920452
H -0.142438 5.048226 3.901342
H -2.438516 4.597486 8.737604
H -4.136779 -0.003780 0.792344
H -3.873883 3.988297 7.910334
H -3.028146 3.438522 1.950778
H -8.421181 0.480206 5.416466
H -8.360809 -0.349792 3.196645
H -7.046603 2.336281 6.833553
H -2.016943 4.902134 2.019996
H -3.800595 -0.049187 -1.677844
H -3.276615 5.612913 7.553797
H -7.399613 -1.779928 -1.471155
H -7.575012 1.650441 4.382852
H -8.895644 -0.855339 1.035215
H -5.519232 3.960803 6.032880
H -5.532075 -0.259328 -1.953212
H -6.268359 3.219096 4.641001
H -1.982683 6.734125 5.549673
H -9.137549 -1.931726 -1.195218
H -1.971439 6.688369 3.785828
H -4.924326 1.241627 -1.246590
H -4.321784 5.845415 5.559703
H -6.160010 2.794223 2.042641
H -5.178308 2.577963 0.603962
H -8.398601 -0.330027 -1.359821
H -5.404738 5.070329 3.440935
H -4.073378 5.744716 2.511759
H -8.676258 1.990810 2.027268
H -7.162978 1.644766 -0.618482
H -3.068687 7.787138 4.627719
H -9.339637 1.303548 0.540071
H -6.503962 3.755016 0.591440
H -5.118082 6.821852 3.445917
H -8.895539 3.016234 0.607564
Energy: −1855.8660038 au

Supplementary Table 26 - TS of the Hydrogen transfer from the toluene to the imido thorium complex
Th 0.304040 0.419279 -0.137427
Si -1.534514 -0.108335 3.185230
Si 2.636616 -2.416791 -1.292950
Si 0.219639 3.733075 -1.999833
N 2.440706 -0.820662 -0.647550
N -1.287018 -0.203697 -1.134769
N 0.863963 2.817715 -0.673457
N 2.525952 1.437192 1.387088
N -0.035309 0.176898 2.349279
C -3.756308 -2.369412 -2.865480
C -4.956260 -2.834397 -3.415096

C -6.108127 -2.047324 -3.338644
C -6.032015 -0.802597 -2.708279
C -4.816127 -0.366821 -2.170129
C -3.630373 -1.123347 -2.223735
C -2.640742 -2.267991 1.602169
C -1.467898 2.071691 5.152324
C 1.852000 -3.693933 1.153821
C 0.736349 -4.426179 -2.281356
C 4.392520 -3.914537 -3.096282
C 0.896612 -2.292573 -3.602270
C 1.018604 -2.920660 -2.208732
C 4.313383 -1.423043 -3.486469
C 4.212722 -2.539894 -2.441838
C -2.452655 3.851451 -0.966108
C 2.916480 4.548039 -2.835175
C -0.520350 -2.576271 4.410683
C -2.439249 2.607743 2.904092
C -2.277307 1.523001 3.972458
C -2.562566 -1.580980 5.498279
C -1.271605 -1.278805 4.728172
C -4.303891 -0.509863 2.284829
C -2.834234 -0.784183 1.943529
C 0.922204 1.905437 -4.142681
C 1.022208 5.889758 -3.815265
C 1.501217 5.085811 -2.601467
C -1.324921 3.003376 -4.388789
C -0.275785 2.498345 -3.389729
C 3.606223 0.472705 1.162633
C 3.632818 -0.072714 -0.274160
C 4.328747 -3.666870 0.755547
C 2.951645 -3.764340 0.089757
C -1.119434 5.957893 -0.650567
C -1.378792 4.760692 -1.572469
C 1.582420 3.523045 0.374242
C 2.829601 2.765017 0.849855
C 2.059319 1.479795 2.773401
C 1.172080 0.287258 3.153412
H -2.874473 -3.013767 -2.942805
H -5.001495 -3.810886 -3.904627
H -7.049579 -2.398397 -3.762524
H -6.925738 -0.176766 -2.640296
H -4.791975 0.613968 -1.684581
H -2.419145 -0.653282 -1.681216
H 0.034764 6.336205 -3.651136
H 1.716763 6.708871 -4.063982
H 0.946129 5.255431 -4.706673
H -0.428954 6.687026 -1.091896
H -1.761376 5.151224 -2.529587
H -2.051934 6.493840 -0.412258
H -0.961000 3.851399 -4.985786
H -2.249110 3.320410 -3.895520
H 1.564523 5.790778 -1.756684
H -3.389161 4.399545 -0.777803
H -1.597924 2.205637 -5.093718
H -0.687191 5.633877 0.304211
H 3.634769 5.362670 -3.023655
H -2.683960 2.988853 -1.598328
H 1.393897 2.641498 -4.809503
H 2.953072 3.878731 -3.701364
H -2.112592 3.441036 -0.009693
H 3.274545 3.973771 -1.975641
H -0.740083 1.667633 -2.822627

H 0.606218 1.060443 -4.769876
H 1.947682 4.523254 0.069703
H 1.694182 1.534228 -3.460037
H 0.949747 3.725996 1.262216
H -3.062945 2.275004 2.067968
H -2.891017 3.525410 3.312625
H -1.464124 2.878915 2.482359
H 3.484486 2.631323 -0.017701
H 1.066787 -1.213311 -3.567902
H -4.508899 0.555292 2.437698
H -2.593902 -0.253329 1.004216
H -4.950925 -0.848806 1.464893
H -0.113983 -2.440631 -4.003556
H 3.391997 3.368663 1.593622
H 3.538509 -1.519362 -4.253894
H 4.191624 -0.435094 -3.033036
H 1.607433 -2.734663 -4.314570
H 3.796013 0.788784 -0.951112
H -3.279915 1.261554 4.347151
H 1.459427 2.389380 2.892587
H -3.234081 -2.530050 0.717962
H 5.286838 -1.441395 -4.002971
H -4.626129 -1.036685 3.194214
H -1.916119 2.991941 5.561128
H 0.217710 -2.448651 -1.612757
H -0.446826 2.328185 4.843545
H -1.597320 -2.487809 1.356790
H 4.589480 0.921154 1.420858
H 4.569115 -0.658299 -0.346229
H 2.918051 1.558954 3.474803
H -2.948984 -2.925865 2.428229
H -0.236788 -4.612869 -2.757092
H -1.389266 1.353944 5.977373
H 3.584897 -4.123298 -3.808240
H 3.451409 -0.374376 1.839596
H 5.058262 -2.401842 -1.748417
H 0.981292 0.406198 4.237328
H 1.491440 -4.960486 -2.874460
H 1.796546 -0.627362 3.092605
H -3.088544 -0.668407 5.801758
H 5.339163 -3.980219 -3.657026
H -3.259024 -2.169291 4.888811
H 0.706549 -4.897347 -1.293307
H -0.623052 -0.695737 5.402099
H 1.851520 -2.713360 1.643386
H 0.851994 -3.829883 0.728570
H 4.392523 -4.726834 -2.359997
H 0.412545 -2.383084 3.873643
H -1.119279 -3.241552 3.780791
H 4.467459 -2.690717 1.236607
H 2.888115 -4.748286 -0.401854
H -2.364837 -2.165437 6.411838
H 5.150198 -3.799182 0.041437
H 1.986222 -4.457690 1.935637
H -0.272500 -3.132818 5.329245
H 4.453476 -4.428041 1.542452
Energy: −1855.8574316 au (Imag=-1260 cm^{-1})

Supplementary Table 27 - 2nd toluene addition to hydroamido Thorium complex
Th -3.115493 2.528482 -3.039337
Si -5.073007 3.095017 0.185232
Si -1.912267 5.694072 -4.787275

Si -2.543054 -0.874953 -4.634892
N -2.950877 4.286812 -4.718497
N -3.608983 0.463922 -4.269071
N -5.486659 2.737951 -4.467094
N -4.970086 3.171178 -1.564149
N -1.388042 2.383715 -1.927500
C -2.615322 4.433995 0.921786
C -7.473995 1.440121 0.506176
C -2.624460 -1.957036 -1.981279
C -2.973738 -0.519009 -7.519033
C -3.446380 7.161989 -2.848778
C 0.185605 6.746060 -3.035003
C -0.350579 7.218851 -6.722422
C 0.657936 4.526355 -4.112703
C -0.439716 5.448944 -3.566388
C -0.779030 4.808782 -7.342312
C -1.324396 6.039042 -6.607847
C -5.764466 5.946859 0.393805
C -5.294992 0.234918 0.194040
C -5.966953 1.476774 0.787930
C -6.234159 4.541254 2.432060
C -6.146731 4.551149 0.900214
C -3.155379 2.395231 2.293003
C -3.292853 3.057511 0.915935
C -5.367471 3.963364 -5.271968
C -3.941674 4.173923 -5.791899
C -0.250823 0.653680 -5.549599
C -1.937843 -2.707087 -6.825890
C -2.894226 -1.579620 -6.415401
C 0.301976 -1.360912 -4.153486
C -0.733215 -0.257832 -4.413296
C -3.858924 7.864101 -5.223348
C -2.815576 7.326592 -4.235609
C -4.106084 -3.132607 -3.637565
C -2.779269 -2.388897 -3.443080
C -5.017788 0.292519 -4.618721
C -5.601326 1.532402 -5.301051
C -6.556604 2.814332 -3.462224
C -6.167272 3.658815 -2.248310
C 0.317225 -0.036911 0.549989
C 2.069139 0.706266 -1.113537
C 3.393436 0.797701 -1.543163
C 4.421728 0.251425 -0.777732
C 4.115036 -0.387299 0.424297
C 2.791980 -0.475954 0.850520
C 1.747796 0.067266 0.089741
H -0.544854 2.451738 -1.362978
H 1.269040 1.134689 -1.713757
H 3.618334 1.300517 -2.480395
H 5.453665 0.322936 -1.112578
H 4.908833 -0.816760 1.031530
H 2.561410 -0.975066 1.790215
H 0.195718 0.369136 1.560980
H -0.009905 -1.083131 0.584210
H -0.359570 0.505449 -0.117708
H -1.894262 -3.511286 -6.082922
H -2.239826 -3.160592 -7.781753
H -0.916358 -2.332549 -6.961075
H -4.218332 -3.528848 -4.653052
H -1.966231 -3.093737 -3.677558
H -4.193404 -3.982830 -2.944803
H 0.403569 -2.039131 -5.009838

H 0.054091 -1.971895 -3.280109
H -3.896610 -2.029646 -6.334895
H -2.707123 -2.812627 -1.294814
H 1.293813 -0.926880 -3.967861
H -4.963095 -2.476535 -3.444120
H -3.327206 -0.953386 -8.466095
H -1.663512 -1.468392 -1.791868
H -0.087146 0.091236 -6.477751
H -1.995422 -0.068925 -7.716834
H -3.402682 -1.237274 -1.705160
H -3.650759 0.296951 -7.250782
H -0.788608 0.367383 -3.504711
H 0.705783 1.127571 -5.290861
H -5.198872 -0.545534 -5.314483
H -0.961646 1.457008 -5.772242
H -5.641162 0.051499 -3.736446
H -4.222095 0.196067 0.411554
H -5.743267 -0.694723 0.573848
H -5.400726 0.227106 -0.897351
H -5.031225 1.707747 -6.218247
H 0.251365 3.573684 -4.461987
H -3.521163 1.363470 2.301940
H -2.725698 2.455649 0.186124
H -2.101009 2.367185 2.603042
H 1.391363 4.294185 -3.328549
H -6.655126 1.364357 -5.595438
H 0.168510 4.469069 -6.912476
H -1.474079 3.965317 -7.294608
H 1.206176 4.993315 -4.940891
H -3.698928 3.345586 -6.482564
H -5.833559 1.444734 1.880393
H -6.754275 1.794976 -3.112824
H -1.545680 4.339378 1.153077
H -0.592473 5.030234 -8.403850
H -3.700238 2.941103 3.073797
H -7.927634 0.507616 0.873998
H -0.882808 4.908604 -2.714141
H -7.680548 1.487367 -0.570300
H -2.690824 4.926978 -0.052074
H -6.093738 3.951034 -6.107449
H -3.985598 5.065450 -6.440397
H -7.491554 3.194474 -3.918127
H -3.052355 5.101242 1.675684
H 0.992140 6.520710 -2.323076
H -8.009635 2.270459 0.979741
H 0.609989 6.992020 -6.245128
H -5.621324 4.813566 -4.630886
H -2.246499 6.336387 -7.132691
H -7.064385 3.677345 -1.605806
H 0.629040 7.346536 -3.839257
H -6.049508 4.706846 -2.582117
H -6.558451 3.571720 2.826580
H -0.134677 7.461294 -7.773661
H -5.263230 4.770105 2.886977
H -0.536364 7.382816 -2.513991
H -7.160755 4.344837 0.522605
H -4.234609 6.401005 -2.868793
H -2.719824 6.843530 -2.093297
H -0.741237 8.128153 -6.251563
H -5.696102 5.982876 -0.696809
H -4.794522 6.267665 0.786541
H -4.689073 7.159714 -5.356785

H -2.022135 8.085969 -4.155641
H -6.944048 5.297851 2.798346
H -3.435833 8.063166 -6.214208
H -3.900382 8.098822 -2.494221
H -6.503829 6.698732 0.708113
H -4.300119 8.805346 -4.862841
Energy: −1895.9192642 au

Supplementary Table 28 - TS of the Hydrogen transfer from the 2nd toluene to the imido thorium complex
Th 1.888693 1.975537 -4.636652
Si 3.952016 5.097786 -5.494207
Si -1.863265 2.782842 -4.136489
Si 3.979535 -1.297393 -5.170539
N -0.429238 2.581092 -5.141521
N 1.375582 1.767646 -7.363974
N 3.197851 3.558524 -5.907943
N 2.701647 -0.163821 -5.566093
N 2.356460 2.549127 -2.660445
C 6.616335 3.951182 -5.941893
C 3.199193 6.793124 -7.808894
C 5.767572 -1.632058 -2.873793
C 2.785921 -3.903335 -5.879421
C -4.143006 4.585219 -3.831336
C -2.501022 2.468813 -1.306045
C -2.442939 -0.045790 -4.046453
C -0.743422 4.180736 -1.851156
C -1.373419 2.848112 -2.276591
C -3.808102 1.224648 -5.719385
C -3.108543 1.298708 -4.355660
C 6.210969 -2.681907 -6.510556
C 2.450655 -3.164625 -3.511233
C 3.389642 -3.098898 -4.719324
C 5.800082 -0.327322 -7.271699
C 5.146730 -1.592918 -6.706415
C -0.086752 1.834128 -7.498322
C -0.702398 2.862138 -6.555909
C 5.013324 4.623321 -2.834356
C 1.546390 6.616856 -5.926751
C 3.033183 6.618416 -6.294022
C 3.934930 6.867420 -3.165329
C 3.920220 5.391467 -3.588560
C 6.503824 6.471227 -5.856096
C 5.752499 5.185005 -6.224168
C -2.070703 5.639112 -4.801686
C -2.886854 4.347809 -4.680012
C 1.882027 -0.569440 -6.713007
C 1.901248 0.472837 -7.828983
C 2.054920 2.898142 -8.014361
C 3.379512 3.205647 -7.322040
C 5.736586 0.655381 -3.908750
C 4.911568 -0.607167 -3.630033
C 1.172853 0.077057 -2.225448
C 1.205048 0.005328 -0.754856
C 2.429861 -0.027137 -0.050797
C 2.477650 -0.042264 1.339432
C 1.303592 -0.017640 2.094926
C 0.080697 0.019068 1.423109
C 0.034003 0.028528 0.031969
H 2.630437 2.863037 -1.735220
H 3.357813 -0.040128 -0.618500
H 3.444115 -0.072040 1.839894
H 1.341292 -0.028625 3.181443

H -0.849671 0.031855 1.988525
H -0.931415 0.041309 -0.468740
H 0.178984 -0.155400 -2.621416
H 1.892739 -0.611406 -2.674415
H 1.732616 1.412406 -2.398067
H 5.785564 -3.637645 -6.187883
H 6.764404 -2.869018 -7.442696
H 6.951549 -2.383474 -5.758918
H 3.419501 -3.903945 -6.773335
H 4.327040 -3.603560 -4.434714
H 2.628826 -4.952953 -5.590278
H 6.608450 -1.986526 -3.482396
H 5.195224 -2.511185 -2.562099
H 4.450947 -1.961335 -7.478467
H 2.221314 -4.206475 -3.242823
H 6.198128 -1.185629 -1.966571
H 1.805967 -3.508006 -6.170147
H 6.230315 -0.508090 -8.267602
H 2.871555 -2.684931 -2.622112
H 6.645275 0.421676 -4.475799
H 6.616309 0.022245 -6.630761
H 1.499119 -2.664656 -3.719954
H 5.086727 0.497145 -7.358360
H 4.091278 -0.308912 -2.959911
H 6.054403 1.129304 -2.970875
H 2.203119 -1.518455 -7.172959
H 5.183299 1.408915 -4.479512
H 0.830368 -0.754254 -6.422059
H -2.048283 -0.090117 -3.026358
H -3.146596 -0.883209 -4.162051
H -1.600062 -0.226818 -4.724243
H 2.941408 0.615089 -8.134660
H 4.983574 3.555688 -3.063321
H -2.909717 1.472281 -1.502759
H -0.586139 2.088194 -2.175396
H -2.130026 2.467875 -0.272653
H 4.874548 4.723917 -1.748615
H 1.339457 0.128712 -8.717000
H 6.863718 3.864467 -4.879033
H 6.110193 3.025772 -6.230823
H 6.015372 5.006027 -3.064666
H 4.042806 2.331318 -7.436964
H -3.893151 1.465145 -3.600966
H -0.490438 0.849048 -7.237657
H -0.335699 4.102794 -0.834630
H 7.567632 3.998636 -6.492611
H -3.336931 3.177047 -1.348964
H -4.547477 0.410274 -5.739767
H 2.965394 4.951759 -3.265582
H -3.094313 1.019376 -6.526071
H 0.084036 4.466961 -2.506159
H 2.200181 2.694570 -9.091645
H 3.864156 3.998309 -7.914348
H -0.369763 2.040512 -8.548310
H -1.481380 4.992695 -1.842770
H 3.887325 6.950821 -2.070314
H -4.337707 2.149190 -5.974668
H 6.727797 6.512761 -4.783867
H 1.413557 3.780728 -7.929942
H 5.593002 5.215678 -7.313508
H -1.777319 2.878247 -6.798998
H 4.851936 7.379247 -3.482604

H -0.342754 3.864478 -6.851575
H -4.772235 3.691585 -3.753038
H 7.466011 6.536354 -6.385214
H -3.883498 4.892017 -2.811618
H 3.089389 7.433967 -3.567875
H -3.235502 4.098078 -5.694307
H 1.044514 5.742202 -6.355813
H 1.382657 6.571687 -4.844897
H 5.933383 7.373162 -6.105123
H -1.175919 5.500426 -5.413723
H -1.737326 5.998982 -3.823160
H 2.724858 5.976009 -8.365051
H 3.495481 7.497577 -5.818829
H -4.764966 5.387271 -4.255737
H 4.250228 6.832811 -8.116356
H 1.031101 7.512606 -6.302848
H -2.666525 6.444332 -5.256613
H 2.723620 7.723449 -8.153557
Energy: −1895.8780889 au (Imag=-1296 cm^{-1})

Supplementary Table 29 - Product of hydrogen transfer from 2nd toluene to the imido Thorium complex
Th -2.653315 1.809430 -2.578203
Si -4.523897 2.688332 0.674748
Si -2.222647 5.114674 -4.810552
Si -1.819637 -1.225249 -4.893485
N -2.826723 3.511177 -4.400684
N -2.888415 -0.155107 -4.003099
N -5.146586 1.675743 -3.824750
N -4.452029 2.512822 -1.079840
N -0.589476 2.648721 -2.118241
C -1.951762 3.957980 1.141730
C -7.033833 1.247722 1.276485
C -0.773180 -3.063855 -2.827671
C -2.639770 -0.387644 -7.590818
C -3.962218 6.461132 -2.970682
C -0.400214 7.038942 -3.537750
C -1.268161 6.652528 -7.097833
C 0.637937 4.915291 -4.372391
C -0.637722 5.543680 -3.791241
C -1.119715 4.148332 -7.370160
C -1.893808 5.301789 -6.724293
C -5.066487 5.575386 0.484695
C -4.945836 -0.133943 1.195962
C -5.520392 1.245906 1.527988
C -5.548129 4.509732 2.712428
C -5.504594 4.290887 1.194354
C -2.645778 2.280149 2.875793
C -2.741419 2.665818 1.393021
C -5.199731 2.803138 -4.768002
C -3.826641 3.058942 -5.383884
C -0.031690 0.966912 -5.633064
C -1.937492 -2.785115 -7.409195
C -2.567948 -1.603056 -6.659338
C 1.029784 -1.302047 -5.510472
C -0.078962 -0.402819 -4.946717
C -4.699774 6.630302 -5.366612
C -3.493087 6.539102 -4.424632
C -2.837494 -3.837237 -4.013875
C -1.554946 -3.004173 -4.144678
C -4.271640 -0.624428 -3.888896
C -5.236837 0.376621 -4.515798
C -6.144485 1.785660 -2.751984

C -5.739458 2.821747 -1.711504
C -1.634769 -0.042696 -0.900596
C 0.845020 0.112772 -0.501488
C 1.951448 0.142651 0.340850
C 1.806482 0.015285 1.723814
C 0.518599 -0.154505 2.236581
C -0.588099 -0.187352 1.394672
C -0.477482 -0.042245 -0.010403
H 0.075671 3.245024 -2.592716
H 0.991339 0.206161 -1.575346
H 2.943865 0.261303 -0.091826
H 2.671675 0.038699 2.381571
H 0.373011 -0.275436 3.309142
H -1.573845 -0.348339 1.825574
H -2.503394 -0.546715 -0.456494
H -1.393628 -0.558986 -1.838139
H -0.125611 2.269074 -1.294304
H -1.983448 -3.718864 -6.840883
H -2.448881 -2.961878 -8.367208
H -0.882862 -2.597967 -7.643946
H -3.424163 -3.866279 -4.939745
H -0.931144 -3.493314 -4.910456
H -2.606901 -4.877683 -3.741723
H 0.891268 -1.481819 -6.583656
H 1.077162 -2.277663 -5.016833
H -3.607911 -1.890796 -6.431591
H -0.531323 -4.104068 -2.563873
H 2.014188 -0.827722 -5.393599
H -3.485872 -3.440811 -3.224493
H -3.272270 -0.592648 -8.467011
H 0.171462 -2.511744 -2.867383
H -0.134951 0.870796 -6.719552
H -1.647949 -0.119530 -7.971737
H -1.348405 -2.643367 -1.997554
H -3.043008 0.496791 -7.088919
H 0.143765 -0.236609 -3.880538
H 0.931807 1.460583 -5.446455
H -4.453217 -1.601870 -4.364201
H -0.817234 1.649689 -5.292634
H -4.570150 -0.782759 -2.832739
H -3.885616 -0.214550 1.452532
H -5.479172 -0.932779 1.732035
H -5.028529 -0.346030 0.123660
H -4.942603 0.514430 -5.560083
H 0.514174 3.851785 -4.597692
H -3.129828 1.323004 3.095534
H -2.242291 1.870485 0.819381
H -1.594281 2.184477 3.175023
H 1.481991 5.013802 -3.674129
H -6.278813 0.011199 -4.509854
H -0.088247 4.096099 -7.007052
H -1.583564 3.180668 -7.160759
H 0.938922 5.412381 -5.302293
H -3.501227 2.126310 -5.873926
H -5.374809 1.414162 2.606513
H -6.208141 0.810336 -2.255007
H -0.898009 3.822137 1.418650
H -1.071209 4.266877 -8.463017
H -3.104263 3.034940 3.526283
H -7.529892 0.452042 1.852113
H -0.822960 5.070195 -2.817413
H -7.267172 1.062290 0.221078

H -1.969364 4.259751 0.090063
H -5.961748 2.614628 -5.546878
H -3.986645 3.770699 -6.209999
H -7.143628 2.010867 -3.171645
H -2.339419 4.789805 1.742963
H 0.504695 7.187283 -2.931144
H -7.509118 2.193814 1.557551
H -0.246078 6.745527 -6.712902
H -5.503966 3.699454 -4.220551
H -2.898768 5.295243 -7.173208
H -6.577791 2.871357 -0.997813
H -0.253573 7.597099 -4.470116
H -5.732124 3.816491 -2.191676
H -5.918868 3.632938 3.254847
H -1.208867 6.774946 -8.189514
H -4.553229 4.742457 3.108698
H -1.228290 7.511694 -3.001024
H -6.539508 4.093499 0.874259
H -4.475633 5.511449 -2.785901
H -3.130632 6.506543 -2.259273
H -1.841451 7.500245 -6.706037
H -5.051351 5.455241 -0.600589
H -4.060807 5.883842 0.787586
H -5.322132 5.729055 -5.319772
H -2.919763 7.471126 -4.545580
H -6.201410 5.355576 2.973186
H -4.404708 6.769347 -6.412464
H -4.658216 7.274229 -2.718078
H -5.744349 6.409302 0.720466
H -5.349632 7.476032 -5.096836
Energy: -1895.9075267 au

Supplementary Table 30 - Benzyl-thorium product
Th -0.121178 0.004521 -0.001985
Si 3.542686 -0.888692 -0.551536
Si -2.163424 0.899964 -3.016769
Si -1.860716 -0.881997 3.276364
N 1.798523 -0.958523 -0.902064
N -1.755150 -0.150317 -1.638940
N -0.860189 -1.204997 1.835187
N -0.604727 -2.634168 -0.684123
C 1.145410 4.340521 -0.265597
C 2.106036 5.343667 -0.172801
C 2.743763 5.607960 1.039565
C 2.393350 4.851485 2.157886
C 1.432597 3.848333 2.062523
C 0.782890 3.554733 0.846919
C -0.220053 2.469255 0.747230
C -4.234506 -2.498169 2.701171
C -3.480335 -2.508638 5.097771
C -3.020170 -2.397441 3.635603
C -3.657094 0.834843 1.713584
C -3.748476 1.076585 4.215625
C -2.850823 0.746152 3.013477
C 6.101575 -0.411710 -1.774386
C 4.328345 -1.079067 -3.430788
C 4.597621 -0.382502 -2.091158
C 4.021702 1.888223 0.225821
C 2.856688 0.424282 1.919478
C 3.875279 0.462840 0.773647
C -1.039248 3.213121 -4.316741
C 0.671574 1.667700 -3.325172

C -0.758965 2.204653 -3.191683
C -3.279126 0.521687 -5.697680
C -1.137665 -0.708505 -5.232793
C -2.433534 -0.166850 -4.614746
C 0.169490 -1.806334 5.129932
C 5.260176 -2.481654 1.219787
C -4.996948 0.977343 -2.422653
C -0.336651 -2.583414 1.750042
C -0.970506 -3.317419 0.570906
C 4.434476 -3.694028 -0.810252
C 4.106227 -2.593858 0.211043
C 1.445234 -2.001489 -1.890217
C 0.662529 -3.139700 -1.245258
C -3.679201 2.985929 -1.674194
C -3.799739 1.886174 -2.734794
C -2.563458 -1.376126 -1.495044
C -1.695567 -2.618543 -1.678408
C 0.030774 0.683322 4.809298
C -0.768179 -0.624543 4.846117
H 0.655375 0.789687 5.706314
H 0.708113 -1.663387 6.076110
H -1.477596 -0.556144 5.684550
H -2.642407 -2.572181 5.798311
H -3.198663 1.076386 5.162477
H -4.095475 -1.653976 5.400030
H -0.614859 1.566073 4.758713
H 2.353167 5.929764 -1.055223
H 3.490094 6.393864 1.113112
H -0.363329 -2.761511 5.201294
H -4.093080 -3.408731 5.241593
H 0.703440 0.714677 3.944348
H -4.201103 2.069970 4.101673
H 0.926784 -1.909307 4.344152
H -4.571526 0.359516 4.312104
H -2.081965 1.530774 2.970126
H 0.652093 4.164840 -1.219339
H -2.395590 -3.281524 3.438824
H 4.676112 -4.637260 -0.301472
H 2.864645 5.050413 3.117945
H -4.980386 -1.729006 2.930998
H 5.306379 -3.430250 -1.419008
H -4.736382 -3.468468 2.813402
H -0.508297 -3.167367 2.663813
H 3.610255 -3.904064 -1.498892
H 5.515639 -3.470287 1.624897
H -4.095501 1.834039 1.596553
H -4.482111 0.116335 1.696898
H -3.966605 -2.385752 1.645204
H 0.759603 -2.590422 1.623002
H 6.170490 -2.081743 0.758888
H -3.056945 0.644222 0.815169
H 4.538778 -2.153954 -3.392183
H 1.162395 3.282856 2.952241
H 3.221012 -2.925431 0.776328
H -1.048987 2.782912 0.087575
H 3.294376 -0.949337 -3.762630
H -2.056963 -3.274378 0.688761
H 5.013104 -1.839658 2.070813
H -0.680498 -4.380377 0.537556
H 4.973076 -0.658868 -4.214897
H -0.681849 2.308963 1.736656
H -3.417414 2.571431 -0.694987

H -3.046376 -1.420500 -0.505156
H 6.472074 -1.438042 -1.670540
H 1.263325 -3.549135 -0.427624
H 2.616508 -0.590496 2.257587
H -4.871875 0.476143 -1.456285
H -4.631013 3.520235 -1.552050
H 4.313476 0.671751 -2.226722
H -2.920719 3.732646 -1.929486
H 6.679438 0.054082 -2.583733
H 1.923800 0.937259 1.633605
H -2.290039 -3.545045 -1.621527
H -5.927484 1.557593 -2.364271
H 4.847207 0.179312 1.201211
H -3.392918 -1.431658 -2.213615
H -0.812463 2.762088 -2.243999
H 0.473947 -3.960609 -1.957208
H 6.352058 0.120757 -0.850428
H 0.960473 0.940551 -2.554244
H 2.324420 -2.433381 -2.384294
H 0.844540 -1.577517 -2.710957
H 3.208672 0.987524 2.792509
H -1.250081 -2.576597 -2.676834
H -5.147765 0.199947 -3.180818
H 1.400791 2.485963 -3.268263
H -4.001652 2.377801 -3.698717
H 3.125539 2.224679 -0.305938
H 4.867965 1.976143 -0.461883
H -2.033726 3.663979 -4.240539
H 0.823785 1.165194 -4.285275
H -3.013644 -1.036533 -4.269714
H 4.182812 2.606559 1.037794
H -0.307134 4.030806 -4.301067
H -0.483371 -1.181741 -4.493229
H -0.966183 2.739635 -5.302357
H -4.273611 0.802819 -5.338666
H -0.562347 0.090864 -5.712654
H -2.797643 1.431001 -6.074545
H -1.353275 -1.454354 -6.009420
H -3.420880 -0.144800 -6.559041
Energy: −1839.9071191 au

Supplementary Table 31 - Phenyl-thorium product
Th -0.135297 0.031037 2.529633
Si -1.120966 -3.636021 2.559607
Si -2.725197 2.784705 2.555217
Si 3.461655 0.962899 3.504668
N -1.171668 -1.950791 3.145101
N -1.408323 1.831805 3.274120
N -0.539694 -0.073814 5.273770
N 1.916002 0.088008 3.640752
C 1.311872 -0.814142 -0.163573
C 1.811172 -1.210034 -1.409883
C 1.109935 -0.867192 -2.561838
C -0.075798 -0.137369 -2.450700
C -0.550851 0.242602 -1.196198
C -5.130431 4.150140 3.518165
C -4.309262 2.656666 0.136664
C 5.036707 -1.482817 4.122953
C 2.138587 2.900480 1.818196
C 1.792257 -3.534471 2.133371
C 0.132504 -4.991694 4.834214
C 4.288293 1.408241 6.317819

C 3.932363 1.582553 0.644368
C 3.466821 2.156095 1.988572
C 4.448235 3.406125 4.824734
C 3.684264 2.097351 5.083251
C -2.570602 -3.330187 0.076313
C -1.718833 -6.260351 3.708201
C -1.188664 -4.863035 4.064168
C -3.978916 -3.725923 2.125483
C -2.641057 -4.030810 1.438552
C -1.200946 4.595697 0.879454
C -3.632826 0.427378 1.066769
C -3.233263 1.900130 0.929655
C -1.616083 5.388211 3.231562
C -2.176739 4.569373 2.061147
C 1.808183 -0.705911 4.887531
C 0.811529 -0.091589 5.866083
C 0.518945 -5.284847 0.862965
C 0.449572 -3.876923 1.475350
C -2.086115 -1.696895 4.275445
C -1.296496 -1.316150 5.524679
C -4.987792 1.701147 4.062633
C -4.179309 2.983811 3.826172
C -1.010692 2.248181 4.631973
C -1.299679 1.136454 5.635635
C 6.301402 0.515973 3.253950
C 4.958125 -0.230445 3.241342
C 0.120133 -0.077309 0.003285
H 1.905408 -1.104381 0.713380
H 2.737444 -1.776190 -1.479750
H 1.481073 -1.165118 -3.539295
H -0.629554 0.132412 -3.347744
H -1.482860 0.805677 -1.161620
H -0.927390 5.626601 0.617214
H -1.431173 6.428439 2.932009
H -3.105493 5.060386 1.732502
H -4.613970 5.113571 3.474441
H -4.043071 3.703155 -0.042751
H -5.647745 4.012217 2.562006
H -1.618812 4.132929 -0.020478
H -2.294795 5.414255 4.091982
H -5.906604 4.232878 4.290993
H -0.272661 4.066742 1.117808
H -4.474803 2.189075 -0.842725
H -0.659076 4.981702 3.577684
H -5.272099 2.646226 0.660260
H -2.306906 1.937516 0.336137
H -3.679145 3.227785 4.776463
H 4.307500 2.096182 7.174113
H -5.630757 1.472307 3.205423
H 5.323478 1.098726 6.137769
H -5.648565 1.806542 4.933488
H -1.524113 3.158235 4.971437
H 3.734614 0.519570 6.633989
H 4.529087 3.992156 5.750352
H -3.735712 -0.045571 0.082448
H -4.591884 0.319110 1.581884
H -4.352225 0.826359 4.235738
H 0.063070 2.496701 4.675158
H 5.470698 3.224238 4.474270
H -2.913897 -0.180395 1.632152
H 5.181558 -1.237232 5.181233
H 2.651864 2.381651 5.338923

H 4.137375 -2.099853 4.041010
H -2.367309 0.903227 5.584699
H 3.953610 4.042557 4.085080
H -1.076770 1.445945 6.670139
H 5.888217 -2.109423 3.824243
H -2.503571 -2.242334 0.185041
H -2.789571 -0.879589 4.043035
H 6.564240 0.854646 4.262379
H 1.114032 0.940375 6.065826
H 1.739779 3.296142 2.758750
H -4.087344 -2.653021 2.319776
H -3.467584 -3.542334 -0.521329
H 4.790881 -0.583379 2.211932
H -1.705104 -3.647406 -0.513520
H 7.116168 -0.138574 2.916549
H 1.374939 2.249613 1.365605
H -1.946439 -1.206067 6.407942
H -4.824697 -4.024539 1.491820
H 4.215496 2.904657 2.283672
H -2.729130 -2.554787 4.513790
H 0.309062 -3.172156 0.642460
H 0.808222 -0.625131 6.831178
H 6.303845 1.397940 2.604307
H 1.814198 -2.535890 2.585604
H 2.771012 -0.817282 5.401275
H 1.491792 -1.739607 4.671408
H 2.237897 3.743188 1.121716
H -0.587486 -2.121246 5.737338
H -4.091208 -4.247717 3.083092
H 2.603622 -3.566719 1.393872
H -2.600746 -5.115418 1.257718
H 3.247818 0.821862 0.259558
H 4.929032 1.135256 0.705631
H -0.398781 -5.562050 0.334389
H 2.051845 -4.246139 2.922885
H -1.919807 -4.420307 4.757015
H 3.979810 2.376455 -0.112870
H 1.343352 -5.353904 0.141336
H 0.581050 -4.021459 5.072701
H 0.701763 -6.046975 1.629356
H -2.724766 -6.229168 3.279900
H 0.872612 -5.559470 4.259532
H -1.071307 -6.771419 2.987079
H -0.014423 -5.529393 5.780465
H -1.765598 -6.896626 4.602428
Energy: −1800.6014177 au

Supplementary Table 32 - Toluene
C 0.004616 -0.000010 -0.025582
C 0.009579 0.000085 1.366602
C 1.213013 0.000070 2.083883
C 2.411697 -0.000060 1.363520
C 2.412622 -0.000164 -0.030948
C 1.208260 -0.000138 -0.730884
C 1.207620 0.000243 3.590448
H 3.356558 -0.000075 1.902681
H 3.356333 -0.000311 -0.570049
H 1.206013 -0.000212 -1.817445
H -0.940645 0.000043 -0.562083
H -0.934336 0.000143 1.907982
H 2.223722 0.000130 3.994480
H 0.691345 0.881865 3.987293

H 0.691061 -0.881115 3.987512
Energy: –271.4704158 au

Supplementary Table 33 - Benzyl potassium
C -0.060432 0.209589 0.042148
C -0.051068 0.190044 1.488645
C 1.276898 0.203437 2.062491
C 2.404413 -0.057822 1.303699
C 2.328617 -0.279035 -0.087928
C 1.077813 -0.051844 -0.700441
C -1.203851 -0.010659 2.250970
H -2.181226 0.125935 1.791961
H -1.162871 0.120603 3.330623
H 1.370879 0.342497 3.138711
H 3.376506 -0.084442 1.795908
H 3.222484 -0.448267 -0.680037
H 1.004495 -0.073766 -1.787681
H -1.013960 0.353642 -0.464288
K 0.338904 -2.520855 1.218345
Energy: –299.1797253 au

Supplementary Table 34 - Phenyl potassium
C -0.027901 -0.005105 -0.023183
C 0.041520 -0.002903 1.387150
C 1.243950 0.000573 2.103114
C 2.459696 0.002359 1.421091
C 2.448689 0.000712 0.027000
C 1.230891 -0.002891 -0.662470
H -0.879501 -0.004117 1.978448
H 1.233920 0.001821 3.193183
H 3.400802 0.004879 1.967223
H 3.390348 0.002117 -0.522211
H 1.287168 -0.004104 -1.755315
K -2.399451-0.029334 -1.359957
Energy: –259.8545859 au

Supplementary Table 35 - Benzene
C 0.004639 -0.003766 0.002658
C 0.004644 -0.002540 1.397371
C 1.212445 -0.000178 2.094751
C 2.420244 0.001023 1.397362
C 2.420240 -0.000179 0.002649
C 1.212438 -0.002544 -0.694730
H -0.936542 -0.003274 1.940740
H 1.212448 0.000732 3.181523
H 3.361434 0.002591 1.940724
H 3.361425 0.000737 -0.540722
H 1.212435 -0.003276 -1.781502
H -0.936552 -0.005787 -0.540699
Energy: –232.1641749 au

Supplementary Table 36 - Dinitrogen
N 0.000000 0.000000 1.102161
N 0.000000 0.000000 -0.002161
Energy: –109.4743689 au

Supplementary Table 37 - Potassium azide
K -0.051008 0.000238 2.409102
N 0.107577 -0.000176 -2.384210
N 0.105302 -0.000488 -1.219823
N 0.102939 -0.000275 -0.022787
Energy: –192.4924013 au

Supplementary Table 38 – Experimental and computed bond lengths (Å) comparison

Complex	Experimental solid state data			Computed data		
	[a]Th-N_X	[b]Th-N_{amide}	Th-N_{amine}	[a]Th-N_X	[b]Th-N_{amide}	Th-N_{amine}
**2**	2.365(3)	2.310(5)	2.673(3)	2.355	2.343	2.709
**4Li**	2.209(2)	2.369(3)	2.742(3)	2.237	2.344	2.767
**4Na**	2.158(5)	2.372(9)	2.739(5)	2.191	2.404	2.775
**4K**	2.147(4)	2.386(7)	2.749(5)	2.183	2.413	2.784
**4Rb**	2.149(6)	2.398(9)	2.739(6)	2.165	2.425	2.768
**4Cs**	2.105(13)	2.402(10)	2.755(13)	2.161	2.426	2.772
**5**	2.290(4)	2.331(6)	2.710(3)	2.311	2.359	2.735

[a] X = α-N of N_3, imido, or NH_2; [b] average value of three Tren^{TIPS} amides

Supplementary Methods

General Experimental Procedures

All manipulations were carried out under an inert atmosphere of dry nitrogen using Schlenk techniques, or an MBraun UniLab glovebox operating under an atmosphere of dry nitrogen. The solvents THF, diethyl ether, toluene and pentane were dried by passage through activated alumina towers and degassed before use. 1,2-dimethoxyethane (DME), hexanes and benzene were distilled from potassium. All solvents were stored over potassium mirrors except for ethers which were stored over activated 4 Å sieves. Deuterated NMR solvents (d_6-benzene, d_8-toluene, d_8-tetrahydrofuran) were purchased from Goss Scientific Ltd and were distilled from potassium, degassed by three freeze-pump-thaw cycles and stored under nitrogen prior to use. ^{n}BuLi (2.5 M in hexanes), NH_3 (0.4 M) in THF, and $(ClSiMe_2CH_2)_2$ were used as purchased. $HN(SiMe_2CH_2)_2$,[1] MCH_2Ph (M = Na, K, Rb, Cs),[2] MC_8 (M = K, Rb, Cs),[3] [Th(Tren^{TIPS})(Cl)] (**1**),[4] [Th^{cyclomet}(Tren^{TIPS})],[4] and [Th(Tren^{DMBS})(I)],[5] and $[Th(Cl)_4(THF)_{3.5}]$[6] were prepared using literature methods.

^{1}H, $^{13}C\{^{1}H\}$,$^{19}F\{^{1}H\}$, $^{29}Si\{^{1}H\}$ NMR spectra were recorded on a Bruker 400 spectrometer operating at 400.1, 100.6, 79.5, and 376 MHz, respectively; chemical shifts are quoted in ppm and are relative to TMS (^{1}H, ^{13}C ^{29}Si), and $CFCl_3$ (^{19}F), respectively. FTIR spectra were recorded on a Bruker Alpha spectrometer with a Pt-ATR module in the glovebox. CHN analyses were carried out by Martin Jennings and Anne Davies (University of Manchester). Crystals were examined on Bruker APEX CCD area detector (graphite-monochromated MoKα radiation, λ = 0.71073 Å) or on Oxford Diffraction SuperNova Atlas CCD (mirror-monochromated CuKα radiation, λ = 1.5418 Å) diffractometers.[7-10]

General Crystallographic Comments

The crystallographic refinements in this publication were largely routine, but here we provide details of restraints that were required. ***Complex 2:*** The azide is disordered over two orientations due to 'wagging' of this group. Restraints were applied to give a linear N_3 unit consistent with other An-N_3 linkages. Restraints were applied to the thermal parameters of the azide group atoms to give sensible ellipsoids. ***Complexes 4K, 4Rb and 4Cs:*** In each structure, one or more triisopropylsilyl group[s] is[are] disordered over two orientations. Chemically equivalent C-C and Si-C distances within isopropyl groups were restrained to be approximately similar to give chemically sensible and consistent metrical parameters within the isopropyl groups. This disorder in such heavy atom structures results in unusual ellipsoid parameters for several atoms in each case so restraints were applied to these parameters, again to provide a chemically sensible model. ***Complex 5:*** The N-H distances of the UNH_2 linkage were not constrained with the usual riding model for hydrogens as this is an unusual linkage where AFIX would not be appropriate. The distances were freely refined but restrained to be approximately 0.91 Å to aid the refinement. ***Complex 6:*** Akin to **4K**, **4Rb**, and **4Cs**, there is a disordered triisopropylsilyl group that was treated in the same way as the disordered groups in **4K**, **4Rb** and **4Cs**. Additionally in **6** the lattice toluene molecules are disordered, so to aid refinement the aromatic C-C distances were restrained to be approximately 1.39 Å and the $C\text{-}C_{methyl}$

distances to approximately 1.55 Å and other 1,3 and 1,4 C-C distances in the lattice solvents were restrained to be approximately equal. The disordered groups lead to unusual ellipsoid parameters for several atoms in each case so restraints were applied to these parameters. ***Complex 8:*** The CH_2CH_2 group is disordered over two orientations. Restraints were applied to give consistent Si-C and C-C distances in the disordered groups and restraints were applied to the thermal parameters to give chemically sensible ellipsoids. ***Complex 9:*** The lattice pentane was ill-defined and disordered. Restraints were applied to give sensible C-C distances in the pentane and to give a sensible geometry. Restraints were applied to the thermal parameters of the pentane to give chemically sensible ellipsoids.

General Computational Details

Geometry optimisations for **2**, **4M**, **5**, **11**, and **12** were performed using coordinates derived from crystal structures or the coordinates from **13** as the starting points. No constraints were imposed during these gas-phase optimisations, and the resulting atomic coordinates were used for single point energy calculations. Calculations were performed using ADF 2012.01.[11,12] The DFT optimisations employed Slater type orbital (STO) triple-ζ-plus polarisation all-electron basis sets (from the Dirac and ZORA/TZP databases of ADF).[11,12] Scalar relativistic approaches were used within the ZORA Hamiltonian[13-15] for the inclusion of relativistic effects and the local density approximation (LDA) with the correlation potential due to Vosko *et al* was used in all of the calculations.[16] Gradient corrections were performed using the functionals of Becke and Perdew.[17,18] For **11** and **12** two additional types of geometry optimisation followed by single point energy calculations were undertaken: (i) frozen-core up to and including 6p for U and Th, all other atoms all-electron; (ii) as for (i) but frozen-core up to and including 5d for U and Th. The computed orbital energies and thus σ-π energy gap for the all-electron vs type (ii) calculations differ by at most 0.01 eV. Frequency calculations were carried out with the analytical frequencies approach within ADF. Natural Bond Order (NBO) analyses were carried out with NBO 5.0.[19] MOLEKEL[20]

was used to prepare the three-dimensional plots of the electron density. The Atoms in Molecules analysis[21,22] was carried out with Xaim-1.0.[23] DFT reaction profile calculations were done with the B3PW91 hybrid functional.[24-26] Th, K, and Si atoms were described by the Stuttgart/Dresden group pseudopotentials with their adapted valence basis sets.[27-31] Small core RECPs were used for Th (30 valence electrons) and large core RECPS were used for K and Si. The other atomic centres were defined with the Pople basis set 6-31G**.[31] Structures were optimised without symmetry constraints. Optimised geometries were classified as transition states or minima by analytical calculations of their vibrational frequencies. Singular points of the Potential Electronic Surface were connected via intrinsic reaction coordinate calculations.[32] All the calculations were done with Gaussian09, modeled as if in benzene or toluene using the SMD model to describe the solvent.[33-34]

Supplementary References

1. B. Zhang, X. Zhou, F. Miao, K. Liu, *Huaxue Xuebao* **1987**, *5*, 456.
2. P. J. Bailey, R. A. Coxall, C. M. Dick, S. Fabre, L. C. Henderson, C. Herber, S. T. Liddle, D. Loroño-González, A. Parkin and S. Parsons, *Chem. Eur. J.* **2003**, *9*, 4820.
3. D. E. Bergbreiter, J. M. Killough, *J. Am. Chem. Soc.* **1978**, *100*, 2126.
4. B. M. Gardner, P. A. Cleaves, C. E. Kefalidis, J. Fang, L. Maron, W. Lewis, A. J. Blake, S. T. Liddle, *Chem. Sci.* **2014**, *5*, 2489.
5. B. M. Gardner, W. Lewis, A. J. Blake, S. T. Liddle, *Organometallics* **2015**, *34*, 2386.
6. T. Cantat, B. L. Scott, J. L. Kiplinger, *Chem. Commun.* **2010**, *46*, 919.
7. SAINT and SMART, Bruker AXS Inc.: Madison, WI, 2001.
8. CrysAlis PRO, Agilent Technologies: Yarnton, England, 2010.
9. G. Sheldrick, *Acta Cryst. Sect. A* **2008**, *64*, 112.
10. O. V. Dolomanov, L. J. Bourhis, R. J. Gildea, J. A. K. Howard, H. Puschmann, *J. App. Cryst.* **2009**, *42*, 339.

11. C. Fonseca Guerra, J. G. Snijders, G. Te Velde, E. J. Baerends, *Theor. Chem. Acc.* **1998**, *99*, 391.
12. G. Te Velde, F. M. Bickelhaupt, S. J. van Gisbergen, A. C. Fonseca Guerra, E. J. Baerends, J. G. Snijders, T. Ziegler, *J. Comput. Chem.* **2001**, *22*, 931.
13. E. van Lenthe, E. J. Baerends, J. G. Sniders, *J. Chem. Phys.* **1993**, *99*, 4597.
14. E. van Lenthe, E. J. Baerends, J. G. Sniders, *J. Chem. Phys.* **1994**, *101*, 9783.
15. E. van Lenthe, A. E. Ehlers, E. J. Baerends, *J. Chem. Phys.* **1999**, *110*, 8943.
16. S. H. Vosko, L. Wilk, M. Nusair, *Can. J. Phys.* **1980**, *58*, 1200.
17. A. D. Becke, *Phys. Rev. A.* **1988**, *38*, 3098.
18. J. P. Perdew, *Phys. Rev. B.* **1986**, *33*, 8822.
19. NBO 5.0: E. D. Glendening, J. K. Badenhoop, A. E. Reed, J. E. Carpenter, J. A. Bohmann, C. M. Morales, F. Weinhold, (Theoretical Chemistry Institute, University of Wisconsin, Madison, WI, 2001); http://www.chem.wisc.edu/~nbo5.
20. S. Portmann, H. P. Luthi, *Chimia* **2000**, *54*, 766.
21. R. F. W. Bader, *Atoms in Molecules: A Quantum Theory*, Oxford University Press, New York, 1990.
22. R. F. W. Bader, *J. Phys. Chem. A* **1998**, *102*, 7314.
23. http://www.quimica.urv.es/XAIM.
24. A. D. Becke, *J. Chem. Phys.* **1993**, *98*, 5648.
25. J. P. Perdew, Y. Wang, *Phys. Rev. B* **1992**, *45*, 13244.
26. K. Burke, J. P. Perdew, W. Yang, *Electronic Density Functional Theory: Recent Progress and New Directions*, ed. J. F. Dobson, G. Vignale, M. P. Das, Plenum, New York, **1998**.
27. A. Moritz, X. Cao, M. Dolg, *Theor. Chem. Acc.* **2007**, *118*, 845.
28. Moritz, X. Cao, M. Dolg, *Theor. Chem. Acc.* **2007**, *117*, 473.
29. Bergner, M. Dolg, W. Kuechle, H. Stoll, H. Preuss, *Mol. Phys.* **1993**, *80*, 1431.

30. T. Leininger, A. Nicklass, W. Kuechle, H. Stoll, M. Dolg, A. Bergner, *Chem. Phys. Lett.* **1996**, *255*, 274

31. P. C. Hariharan, J. A. Pople, *Theor. Chim. Acta* **1978**, *28*, 213.

32. K. Fukui, *Acc. Chem. Res*. **1981**, *14*, 363.

33. Gaussian 09, Revision A.02, M. J. Frisch, G. W. Trucks, H. B. Schlegel, G. E. Scuseria, M. A. Robb, J. R. Cheeseman, G. Scalmani, V. Barone, G. A. Petersson, H. Nakatsuji, X. Li, M. Caricato, A. Marenich, J. Bloino, B. G. Janesko, R. Gomperts, B. Mennucci, H. P. Hratchian, J. V. Ortiz, A. F. Izmaylov, J. L. Sonnenberg, D. Williams-Young, F. Ding, F. Lipparini, F. Egidi, J. Goings, B. Peng, A. Petrone, T. Henderson, D. Ranasinghe, V. G. Zakrzewski, J. Gao, N. Rega, G. Zheng, W. Liang, M. Hada, M. Ehara, K. Toyota, R. Fukuda, J. Hasegawa, M. Ishida, T. Nakajima, Y. Honda, O. Kitao, H. Nakai, T. Vreven, K. Throssell, J. A. Montgomery, Jr., J. E. Peralta, F. Ogliaro, M. Bearpark, J. J. Heyd, E. Brothers, K. N. Kudin, V. N. Staroverov, T. Keith, R. Kobayashi, J. Normand, K. Raghavachari, A. Rendell, J. C. Burant, S. S. Iyengar, J. Tomasi, M. Cossi, J. M. Millam, M. Klene, C. Adamo, R. Cammi, J. W. Ochterski, R. L. Martin, K. Morokuma, O. Farkas, J. B. Foresman, D. J. Fox, Gaussian, Inc., Wallingford CT, 2009.

34. Marenich, A. V.; Cramer, C. J.; Truhlar, D. G. *J. Phys. Chem. B* **2009**, *113*, 6378–6396.
